# Discovery of Galloyl–Flavonoid Conjugates as SARS-CoV-2 3CL^pro^ Inhibitors: Understanding Binding Interactions Through Computational Approaches

**DOI:** 10.3390/ijms26199742

**Published:** 2025-10-07

**Authors:** Nopawit Khamto, Panida Boontawee, Vachira Choommongkol, Kritsada Pruksaphon, Suwicha Patnin, Nuttee Suree, Panchika Prangkio, Puttinan Meepowpan

**Affiliations:** 1 Department of Biochemistry, Faculty of Medical Science, Naresuan University, Phitsanulok 65000, Thailand; 2 Center of Excellence in Medical Biotechnology (CEMB), Faculty of Medical Science, Naresuan University, Phitsanulok 65000, Thailand; 3 Cellular and Molecular Immunology Research Unit, Faculty of Allied Health Sciences, Naresuan University, Phitsanulok 65000, Thailand; 4 Department of Chemistry, Faculty of Science, Chiang Mai University, Chiang Mai 50200, Thailand; panida_boontawee@cmu.ac.th (P.B.); nuttee.suree@cmu.ac.th (N.S.); panchika.p@cmu.ac.th (P.P.); 5 Multidisciplinary and Interdisciplinary School, Chiang Mai University, Chiang Mai 50200, Thailand; 6 Department of Chemistry, Faculty of Science, Maejo University, Chiang Mai 50290, Thailand; vachira@mju.ac.th; 7 Center of Excellence Research for Melioidosis and Microorganisms (CERMM), Walailak University, Nakhon Si Thammarat 80160, Thailand; kritsada.pru@mail.wu.ac.th; 8 Laboratory of Organic Synthesis, Chulabhorn Research Institute, Bangkok 10210, Thailand; suwicha@cri.or.th; 9 Center of Excellence in Materials Science and Technology, Chiang Mai University, Chiang Mai 50200, Thailand; 10 Center of Excellence for Innovation in Chemistry (PERCH-CIC), Faculty of Science, Chiang Mai University, Chiang Mai 50200, Thailand

**Keywords:** SARS-CoV-2, flavonoids, structural modification, molecular dynamics, fragment molecular orbital

## Abstract

The emergence of SARS-CoV-2 in 2019 posed significant global public health challenges. One of the most promising targets for novel antiviral drug development is the SARS-CoV-2 main protease (3CL^pro^). In this study, fragment molecular orbital (FMO) calculations were conducted to provide guidance for the structural modification of natural flavonoids, identifying the pyrogallol moiety as a key candidate. Natural flavonoids were chemically modified to generate 33 semi-synthetic derivatives through the introduction of various functional groups. Our findings revealed that the incorporation of a galloyl moiety significantly enhances anti-proteolytic activity against SARS-CoV-2 3CL^pro^, achieving up to a 23-fold increase compared to the activity of the parent compounds. Notably, 7-*O*-galloyl-DMC (**40**) exhibited the highest anti-proteolytic activity in an enzymatic assay. Additionally, molecular dynamics simulations provided atomic-level insights into the interactions between the galloyl moiety and 3CL^pro^. All galloylated flavonoid derivatives positioned their galloyl groups within the S1′ sub-pocket, facilitating hydrogen bonding and π-interactions, particularly with Thr26 and Leu27. These findings underscore the potential of the galloyl moiety as a crucial structural element for enhancing the binding affinity of flavonoids with inhibitory activity against SARS-CoV-2 3CL^pro^.

## 1. Introduction

The COVID-19 pandemic has had one of the most severe impacts on humanity of any disease. First identified in late 2019 in Wuhan, Hubei Province, China [1,2], SARS-CoV-2 infection causes symptoms resembling pneumonia, known as severe acute respiratory syndrome (SARS), which later came to be recognized as coronavirus disease 2019 (COVID-19) [3]. Since its initial outbreak, the disease has led to a significant toll in terms of fatalities worldwide and has led to several damaging effects [4,5].

SARS-CoV-2 belongs to the order *Nidovirales*, within the *Coronaviridae* family and *Coronavirinae* subfamily, and is further classified under the *Betacoronavirus* genus [6]. Coronaviruses possess a single-stranded, positive-sense RNA genome, typically ranging from 26 to 32 kilobases in length [7,8,9]. SARS-CoV-2 shares substantial genetic similarity with other highly pathogenic coronaviruses, including severe acute respiratory syndrome coronavirus (SARS-CoV) and Middle East respiratory syndrome coronavirus (MERS-CoV), with genetic identities of 79% and 50%, respectively [10,11]. The structural proteins of SARS-CoV-2 include the membrane (M), nucleocapsid (N), spike (S), and envelope (E) proteins [12,13].

A key target for antiviral drug development against SARS-CoV-2 is the viral main protease (M^pro^), also known as 3-chymotrypsin-like protease (3CL^pro^) or non-structural protein 5 (nsp5), which plays an essential role in viral replication and assembly [14]. The 3CL^pro^ enzyme functions as a cysteine protease, cleaving polyproteins into non-structural proteins at 11 specific sites—7 on polyprotein 1a (pp1a) and 4 on polyprotein 1b (pp1b) [15,16]. The catalytic dyad in the active site of SARS-CoV-2 3CL^pro^, composed of His41 and Cys145, is critical for enzymatic catalysis, making it a prime target for inhibitor design [17].

Significant advancements have been made in the development of antiviral agents specifically designed to combat SARS-CoV-2. Several promising candidates have been identified as inhibitors of the viral main protease (3CL^pro^). The flavonoid quercetin, also known as 3,3′,4′,5,7 pentahydroxyflavone, has demonstrated inhibitory activity by interacting with the Gln189 residue of the enzyme [18,19]. Additionally, *Shuanghuanglian*, a traditional Chinese medicine used to treat respiratory infections and containing baicalin and baicalein, exhibited significant antiviral activity against SARS-CoV-2 in a Vero E6 cell model [20]. The organoselenium compound Ebselen, known for its anti-inflammatory, antioxidant, and cytoprotective properties [21,22], also strongly inhibits 3CL^pro^, with an IC_50_ value of 0.67 μM [23]. Among covalent inhibitors, Pfizer developed Paxlovid (PF-07321332) as an oral antiviral treatment for COVID-19. This medication combines nirmatrelvir, a protease inhibitor, with ritonavir, which enhances the levels of nirmatrelvir by inhibiting its metabolism [24,25]. Paxlovid has been widely administered worldwide, demonstrating broad antiviral activity against human coronaviruses in vitro and exhibiting a promising safety profile in animal models [26]. Despite the development of various SARS-CoV-2 3CL^pro^ inhibitors, many of these drugs are associated with notable side effects. Therefore, there remains a significant demand for novel inhibitors with improved efficacy and favorable pharmacokinetic properties [27].

In this study, we focused on natural flavonoids, including pinostrobin (**6**), pinocembrin (**7**), cardamonin (**8**), dimethylcardamonin (DMC, **9**), tectochrysin (**10**), and chrysin (**11**). Previous studies have demonstrated that these flavonoids exhibit diverse bioactivities, including antioxidant, anticancer, antiviral, anti-inflammatory, anti-aromatase, antibacterial, and anti-leukemic properties [28,29,30]. Fragment molecular orbital (FMO) calculations were employed to investigate the role of flavonoids containing a pyrogallol moiety with activity against 3CL^pro^. The study identified this moiety as a key structural feature for binding within the S1′ sub-pocket of SARS-CoV-2 3CL^pro^. Based on these findings, the flavonoids were structurally modified, yielding 33 semi-synthetic derivatives. Enzymatic assays were conducted to evaluate their inhibitory activity against SARS-CoV-2 3CL^pro^, highlighting the potential of semi-synthetic flavonoids. To gain atomic-level insights into their interactions with 3CL^pro^, we employed several computational approaches: molecular docking, molecular dynamics (MD) simulations, molecular mechanics with generalized Born surface area (MM-GBSA), and FMO calculations. The results demonstrate that the galloyl moiety plays a crucial role in enhancing binding affinity towards SARS-CoV-2 3CL^pro^.

## 2. Results and Discussion

### 2.1. FMO Analysis of SARS-CoV-2 3CL^pro^—Inhibitor Complex

To better understand the key molecular interactions stabilizing the known SARS-CoV-2 3CL^pro^ inhibitor, we performed fragment molecular orbital (FMO) calculations on four crystal structures of 3CL^pro^–inhibitor complexes, including baicalein (**1**, PDB ID: 6M2N) [20], myricetin (**2**, PDB ID: 7DPP) [31], 7-*O*-methylmyricetin (**3**, PDB ID: 7DPU) [32], and robinetin (**4**, PDB ID: 8HI9) [33] (as summarized in Table 1), possessing the pyrogallol moiety or 1,2,3-trihydroxybenzene in their structures. Analysis of the crystal structures of SARS-CoV-2 3CL^pro^ in complex with these compounds revealed that this moiety binds within the S1 sub-pocket in the case of baicalein (**1**), and within the S1′ sub-pocket in the case of myricetin (**2**), 7-*O*-methylmyricetin (**3**), and robinetin (**4**).

**Figure 1:**
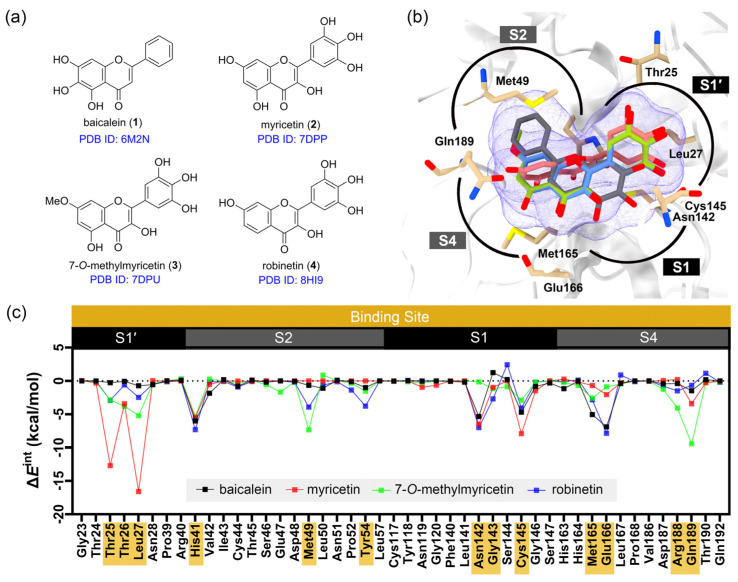
Details of the FMO calculations. (**a**) The chemical structures of the flavonoid inhibitors co-crystallized with SARS-CoV-2 3CL^pro^. (**b**) Superimposed binding poses of the flavonoid inhibitors within the substrate binding site, divided into four sub-pockets: S1, S1′, S2, and S4. Hydrogen atoms are omitted for clarity. (**c**) The pair interaction energy (PIE, Δ*E*^int^) between each co-crystallized ligand and residues within 7 Å. Residues with Δ*E*^int^ lower than −0.5 kcal/mol are highlighted in yellow.

**Figure 2:**
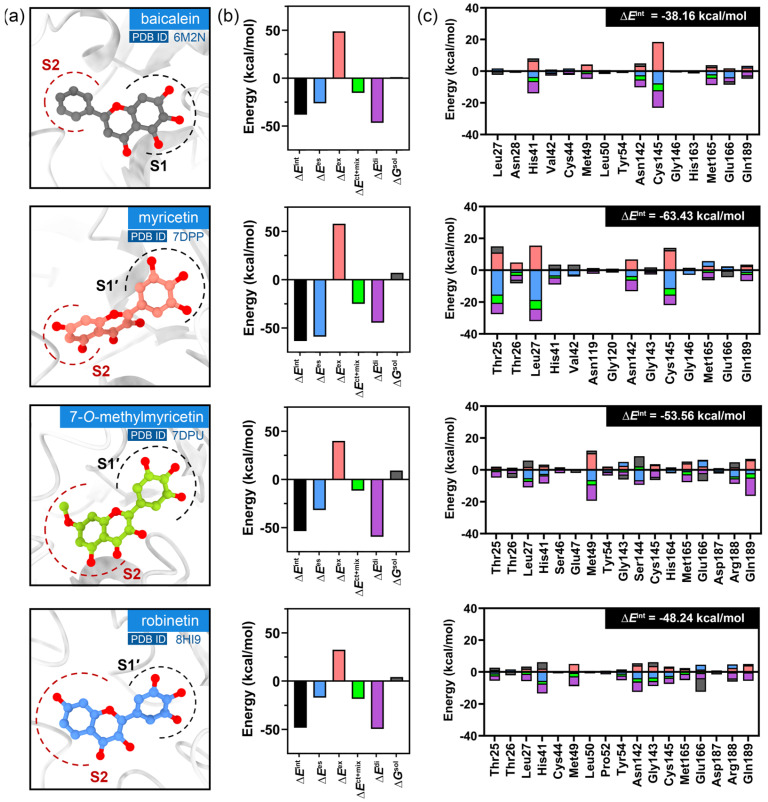
The results of the FMO calculations. (**a**) Binding poses at the substrate binding site of SARS-CoV-2 3CL^pro^. (**b**) Pair interaction (PIE, $∆E^{\mathrm{int}}$), electrostatic ($∆E^{\mathrm{es}}$), exchange repulsion ($∆E^{\mathrm{ex}}$), charge transfer ($∆E^{\mathrm{ct}+\mathrm{mix}}$), dispersion ($∆E^{\mathrm{di}}$), and solvation ($∆G^{\mathrm{sol}}$) energies. (**c**) The $∆E^{\mathrm{int}}$ contributions of the most significant residues with $∆E^{\mathrm{int}}$ lower than −0.5 kcal/mol.

**Figure 3:**
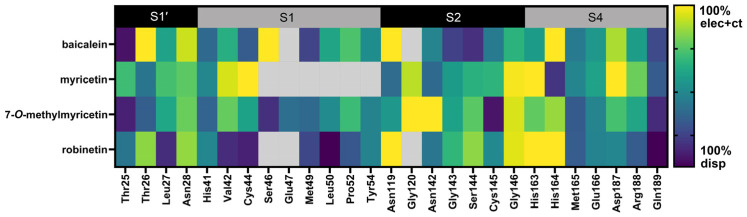
Comparison of the residues involved in the protein–ligand interactions. In the matrix, a colored box indicates the presence of contact between the ligand and the residue, while a gray box indicates the absence of contact. Boxes are otherwise colored according to their PIEDA type, ranging from violet (100% dispersion contribution) to yellow (100% sum of electrostatics + charge transfer). A mixed contribution is represented by green, as shown in the spectrum on the right-hand side.

In this study, FMO calculations were employed to analyze the role of the pyrogallol portion in binding to the 3CL^pro^ receptor. Pair interaction energy decomposition analysis (PIEDA) allowed for the quantification of residue-level interactions and validation of critical binding motifs. The results of PIEDA revealed similar protein–ligand interaction profiles (Figure 1 and Figure 2). All flavonoids interacted with the S1′, S2, and S4 sub-pockets, with partial interactions observed with the S1 sub-pocket. The covalent inhibitor flavonoids, including myricetin (**2**), 7-*O*-methylmyricetin (**3**), and robinetin (**4**), demonstrated interactions with residues in the S1′ sub-pocket, particularly Thr25, Thr26, and Leu27. In contrast, baicalein (**1**), a non-covalent inhibitor, exhibited a distinct interaction profile, lacking contributions from these particular residues, consistent with its different binding mode as observed in its crystal structure. Additionally, all the compounds showed strong interactions with the catalytic dyad residues His41 and Cys145, located in the S1 and S2 sub-pockets. Most of the flavonoid inhibitors interacted with the catalytic residues His41 and Cys145 through electrostatic and dispersion interactions. They all exhibited greater dispersion energy than electrostatic interactions, except for myricetin. The residue Leu27 showed strong electrostatic interactions with the pyrogallol moiety of all of the flavonoid inhibitors, except for baicalein. To gain further insight into the specific interactions within the protein–ligand complex, PIEDA was conducted to investigate electrostatic and charge transfer energies, as well as dispersion energy (Figure 3). The residues Thr26 and Leu27 in the S1′ sub-pocket exhibited significant electrostatic and charge transfer interactions, highlighting the contribution of the hydroxy group from the pyrogallol portion. Moreover, the residues Gly146, His163, and His164 within the S2 and S4 sub-pockets showed stronger contributions of electrostatic and charge transfer interactions than dispersion interactions, driven by the chromone ring and its hydroxy groups. These interactions were less pronounced for baicalein (**1**), as the sub-pocket was occupied by an unsubstituted phenyl group. Furthermore, residues within the S4 sub-pocket exhibited stronger electrostatic and charge transfer interactions compared to dispersion energy, except for Gln189.

**Figure 4:**
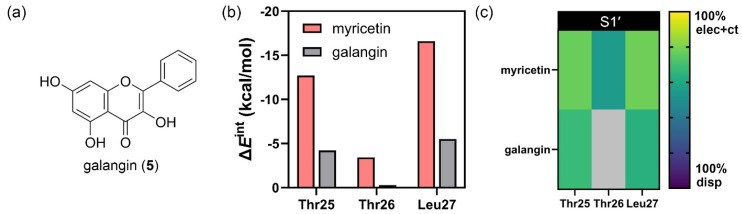
PIEDA of amino acid residues within the S1′ sub-pocket of SARS-CoV-2 3CL^pro^ and of the flavonoids myricetin (**2**) and galangin (**5**). (**a**) The chemical structure of galangin (**5**). (**b**) PIEDA of crucial amino acid residues in the S1′ sub-pocket. (**c**) Interaction contributions, including electrostatic, charge transfer, and dispersion interactions.

To investigate the role of the trihydroxy groups on the pyrogallol portion, the hydroxy groups on myricetin (**2**) were replaced with hydrogen atoms or galangin (**5**) (Figure 4a). Galangin (**5**), a flavonol analog of myricetin (**2**) which lacks the trihydroxy group on the B-ring, was modeled in the same pose as myricetin to evaluate how pyrogallol substitution affects interaction energy via FMO calculation. The PIEDA revealed a significant increase in the total interaction energies from myricetin (**2**, $∆E^{\mathrm{int}}$ = −63.43 kcal/mol) to galangin (**5**, $∆E^{\mathrm{int}}$ = −39.48 kcal/mol), particularly for electrostatic and charge transfer interactions with the residues Thr25, Thr26, and Leu27 (Figure 4b). These findings suggest that the presence of trihydroxy groups plays a crucial role in binding to the S1′ sub-pocket of 3CL^pro^, especially through hydrogen bonding. Further analysis of the interaction contributions indicated that the absence of hydroxy groups (as in the case of **5**) resulted in lower electrostatic and charge transfer interactions (Figure 4c). Additionally, the interaction with residue Thr26 was absent when the trihydroxy portion was removed. These observations suggest that the hydroxy groups on the pyrogallol portion contribute significantly to hydrogen bonding interactions with residues within the S1′ sub-pocket, marking them as one of the key pharmacophores for the S1′ sub-pocket of SARS-CoV-2 3CL^pro^.

Based on the calculations, the pyrogallol moiety was utilized as a pharmacophore to enhance the bioactivity of flavonoids against SARS-CoV-2 3CL^pro^. In this study, gallic acid, which is structurally similar to pyrogallol, was conjugated to the flavonoids. X-ray crystallography studies of myricetin (**2**), 7-*O*-methylmyricetin (**3**), and robinetin (**4**) revealed that these compounds covalently bound to the catalytic residue Cys145. The pyrogallol group served as an electrophilic warhead, reacting with the thiolate nucleophile of Cys145. Building on this rationale, an acryloyl group, a potent electrophilic moiety, was also incorporated into the flavonoids to facilitate covalent binding with 3CL^pro^, and its efficacy was compared with that of the galloyl group.

### 2.2. Isolation of Natural Flavonoids and Synthesis of Flavone Scaffolds

Four flavonoid substrates, namely pinostrobin (**6**), pinocembrin (**7**), cardamonin (**8**), and DMC (**9**), were isolated from *Boesenbergia rotunda* and *Syzygium nervosum*. The flavones tectochrysin (**10**) and chrysin (**11**) were synthesized from **6** and **7** via an iodine oxidation reaction, following a method described in a previous publication [34]. These compounds were successfully characterized using spectroscopic techniques, including ^1^H- and ^13^C-NMR, FTIR, and HRMS. HPLC analysis revealed that these compounds exhibited purity levels exceeding 95%.

### 2.3. Syntheses of Flavonoid Derivatives

**Chart 1:**
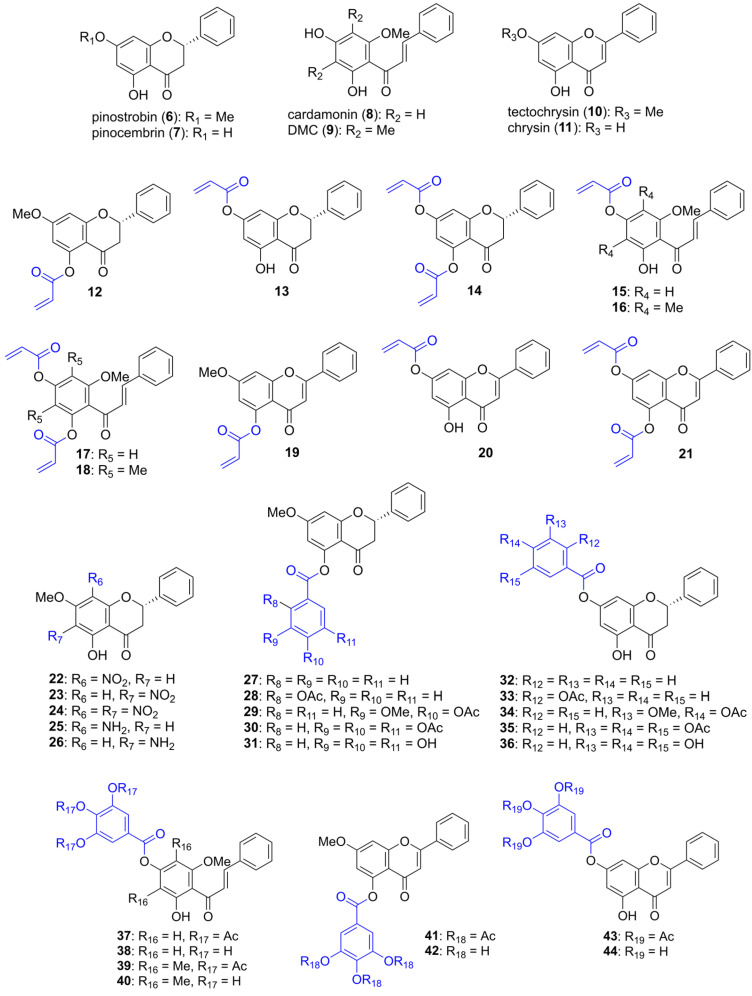
The chemical structures of natural flavonoids **6**–**9** and their semi-synthetic derivatives **10**–**44**.

To assess the functional groups that enhance the inhibitory activity of flavonoid derivatives, several functional groups were introduced into the flavonoid structure through conventional organic reactions, such as nitration, reduction, and esterification, as shown in Chart 1 or Supplementary Material Section I. The selection of these functional groups was based on the previous results of the FMO calculations.

A Michael acceptor is an electron-deficient alkene that can undergo Michael addition with a nucleophile, such as the thiolate of cysteine, to form a covalent bond. Firstly, the acryloyl group, a strong Michael acceptor and α,β-unsaturated group, was incorporated into various parent compounds to yield acryloyl–flavonoid derivatives **12**–**21**. The acryloyl group acts as an electrophile, reacting with the thiolate nucleophile of the catalytic residue Cys145 to form a covalent 3CL^pro^—inhibitor adduct, thereby causing permanent inhibition of enzyme activity. The synthesis of these acryloyl-flavonoid derivatives was achieved by reacting flavonoids with acryloyl chloride under basic conditions, and all the acryloylated products were obtained in high yields.

Secondly, the aromatic ring of the flavonoids was functionalized by introducing nitro and amine groups to explore the effects of electron-withdrawing and electron-donating groups on bioactivity. The nitro group was introduced into **6** through conventional nitration using acetic acid and nitric acid, yielding compounds **22**–**24** in moderate yields. These nitrated products were subsequently reduced using zinc dust under acidic conditions to obtain compounds **25** and **26**. However, the diaminated product was not observed due to decomposition.

Finally, we explored a focused series of flavonoid derivatives bearing the pyrogallol moiety, which has been generally identified in natural inhibitors such as baicalein and myricetin. Our aim was to assess how small substituent modifications, specifically to the hydroxy, methoxy, and acetoxy groups, would influence the flavonoids’ inhibitory activity against SARS-CoV-2 3CL^pro^. Thus, various functionalized aromatic acyl groups, including benzoyl, salicyloyl, vanilloyl, and galloyl groups, were attached to the flavonoids through esterification with their acyl chlorides. The incorporation of these groups was intended to enhance binding through π-interactions and hydrogen bonding with 3CL^pro^. Salicylic acid, vanillic acid, and gallic acid were first protected with acetyl groups using acetic anhydride and a catalytic amount of H_2_SO_4_. The acetylated products were then converted into their acyl chloride using SOCl_2_ and esterified with flavonoids under basic conditions, yielding compounds **27**, **28**, **29**, **30**, **32**, **33**, **34**, **35**, **37**, **39**, **41**, and **43** in high yields. Additionally, the triacetylgalloyl–flavonoids **30**, **35**, **37**, **39**, **41**, and **43** were deprotected under basic conditions using NaHCO_3_ in MeOH or a MeOH/THF mixture to yield compounds **31**, **36**, **38**, **40**, **42**, and **44** in high yields.

Characterization of these semi-synthetic flavonoids was performed using spectroscopic techniques, including ^1^H- and ^13^C-NMR, FTIR, and HRMS, as detailed in Supplementary Material Section I (Figures S1−S72). Additionally, HPLC analysis determined that the purity of these semi-synthetic compounds exceeded 95% (Figures S73−S105).

### 2.4. Enzymatic Assay

**Figure 5:**
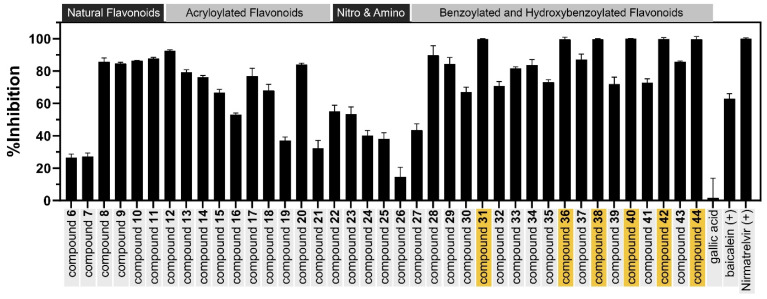
The inhibitory activity of the natural flavonoids and their semi-synthetic derivatives against SARS-CoV-2 3CL^pro^ at a concentration of 100 µM. Compounds with strong inhibitory activity are highlighted with yellow boxes.

All the flavonoid derivatives were evaluated for their inhibitory activity against SARS-CoV-2 3CL^pro^ using an enzymatic assay, as depicted in Table 2 and Figure 5.

The attachment of an acryloyl group to **6** at 5-OH to yield **12** markedly enhanced the flavonoid’s inhibitory activity. Furthermore, other acryloylated flavonoid derivatives, **13**–**21**, were synthesized by attaching an acryloyl group at various positions, such as the 5-OH and 7-OH positions of flavanones **6** and **7** and flavones **10** and **11**, as well as the 2′-OH and 4′-OH positions of chalcones **8** and **9**. These compounds were also evaluated to confirm that the attachment of the acryloyl group affected 3CL^pro^ inhibitory activity. Although compounds **12**–**21** were designed to introduce α,β-unsaturated carbonyl groups as potential Michael acceptors, a lack of proper orientation and anchoring interactions likely prevented effective covalent bond formation with Cys145. This highlights the importance of using covalent docking tools or reactivity-guided design to pre-screen such warheads before synthesis, ensuring that both chemical reactivity and spatial positioning are favorable for covalent inhibition.

The incorporation of electron-withdrawing groups, such as the incorporation of a nitro group to replace H-6, H-8, and H-6,8 of pinostrobin (**6**) to yield compounds **22**–**24**, did not significantly enhance their anti-proteolytic activity compared to that of the parent compound **6**. The reduction of the nitro group to an electron-donating group, such as an amino group, in compounds **25** and **26** also did not improve these compounds’ antiviral activity.

The benzoyl, salicylyl, vanillyl, *p*-hydroxybenzoyl, and galloyl moieties were attached to **6** and **7** by esterification with 5-OH and 7-OH, respectively. Among the products, the inhibitory activity against 3CL^pro^ was improved in compounds **28**, **29**, **30**, **32**, **33**, **34**, and **35**, with inhibition percentages ranging from 67.09% to 89.73% at 100 µM. It can be concluded that the presence of an aromatic ester on the flavonoid inhibitors enhanced their anti-proteolytic activity. Among these esters, the galloyl portion was selected for attachment to other flavonoids due to its previously reported 3CL^pro^ inhibitory activity and our FMO calculations [35]. The galloyl group was then attached to flavonoids at various positions, such as the 5-OH and 7-OH positions of flavones **10** and **11**, as well as the 2′-OH and 4′-OH positions of chalcones **8** and **9**. The acetylated galloyl–flavonoid derivatives **30**, **35**, **37**, **39**, **41**, and **43** exhibited moderate inhibitory activity at 100 µM. Surprisingly, the deprotected products **31**, **36**, **38**, **40**, **42**, and **44** showed excellent anti-proteolytic activity, with inhibition percentages greater than 99% at 100 µM and IC_50_ values of 7.42, 7.53, 13.34, 6.85, 20.58, and 9.26 µM, respectively. Dose-dependent curves of the galloyl–flavonoid derivatives are presented in Supplementary Material Figure S118. Among them, compound **40** exhibited the highest SARS-CoV-2 3CL^pro^ inhibitory activity. All the galloyl–flavonoid derivatives showed greater 3CL^pro^ inhibitory activity than their parent compounds, with a maximum increase of over 23-fold in the case of **31**. These results suggest that the galloyl groups significantly enhanced the SARS-CoV-2 3CL^pro^ inhibitory activity of the flavonoids. Although the introduction of the pyrogallol group is not novel, it was used here as a key pharmacophore to enhance interactions with the 3CL^pro^ active site. The modest improvements in activity observed in our series suggest that such modifications alone may not be sufficient to achieve high potency.

Additionally, all the galloylated flavonoids were validated as not being bad actors using the Python script RDKit. PAINS filters, as defined by Baell and Holloway [36], and toxicity filters, as proposed by Brenk et al. [37], were applied. All candidates successfully passed the PAINS and toxicity screening, suggesting that they are unlikely to exhibit common assay interference and toxicity behavior.

### 2.5. Structure–Activity Relationships (SARs)

**Figure 6:**
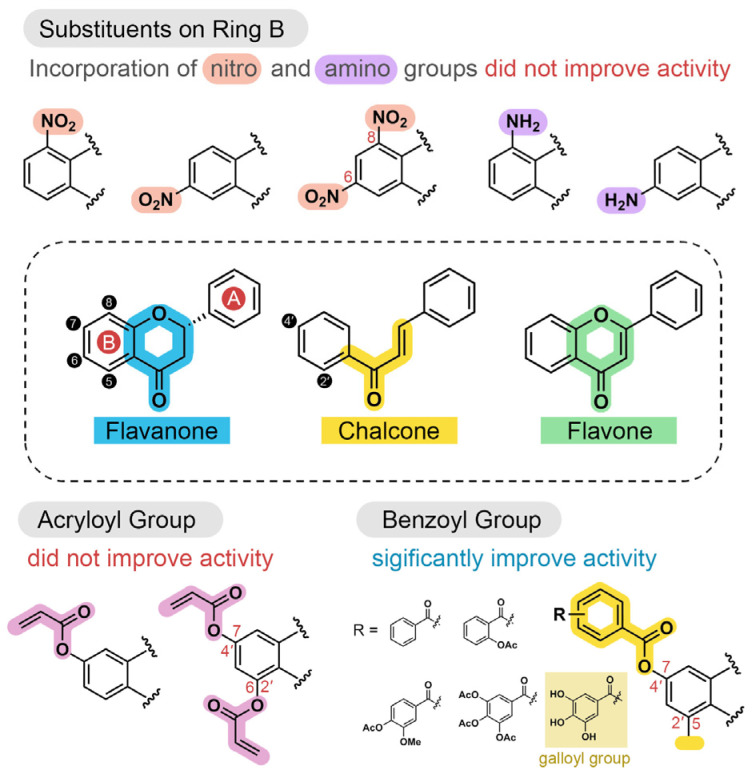
Structure–activity relationships (SARs) of modified flavonoids with inhibitory activity against SARS-CoV-2 3CL^pro^.

Based on the bioactivity results from the enzymatic assay, structure–activity relationships (SARs) were established (Figure 6).

The attachment of acryloyl groups to flavonoids at ring B (compounds **12**−**21**) markedly increase the flavonoids’ anti-proteolytic activity. Similarly, introducing nitro or amine groups at C-5 or C-7, or at both positions (compounds **22**−**26**), did not enhance antiviral activity. In contrast, benzoyl groups and their derivatives−such as salicylyl, vanillyl, and galloyl substituents−improved the antiviral activity of the flavonoids. The acetyl deprotection of galloyl groups in compounds **31**, **36**, **38**, **40**, **42**, and **44** significantly enhanced their antiviral activity compared to their protected derivatives, highlighting the role of hydroxy groups in binding to the 3CL^pro^ binding site. The galloylated flavones **44** showed greater inhibitory activity than the galloylated chalcone **38**. This suggests that flavones possess stronger inhibitory activity than chalcone, consistent with the parent compounds **8** and **11**. Moreover, the presence of methyl groups at C-2′ and C-4′ in compound **40** resulted in stronger bioactivity compared to that of **38**, likely due to enhanced hydrophobic interactions. These findings are consistent with our previous publication [34].

### 2.6. Molecular Docking

To gain insight into the atomistic interactions between the semi-synthetic inhibitors and the 3CL^pro^ receptor, the possible binding orientations of the galloyl–flavonoids were predicted using molecular docking techniques. To validate the docking protocol, the co-crystallized inhibitor myricetin was docked into its original crystal structure (PDB ID: 7B3E). The covalent bond between the ligand and Cys145 was removed prior to docking to simulate the pre-reactive, non-covalent binding state. However, non-covalent docking cannot fully recapitulate the covalent bond pose due to the absence of bond formation, steric hinderance, and electronic redistribution. The closest pose was located within a RMSD of 1.975 Å of the experimental structure (Supplementary Material Figure S106). This indicates both the approximate validity and the limitations of applying non-covalent docking to covalent ligands. Additionally, the known flavonoid inhibitor baicalein was used for validation by docking it to the substrate binding site. The docked pose showed close alignment with the experimental binding mode observed in the crystal structure PDB ID: 6M2N [20]. However, the superimposition between the docked and co-crystalized structures was not fully consistent, as the selected 3CL^pro^ crystal structure is covalently bound to a macromolecule, which may be distorted due to covalent modification. In the docking model, baicalein aligned its chromone ring within the S1 sub-pocket and its monosubstituted benzene ring within the S2 sub-pocket, closely resembling the orientation observed in the crystal structure, supporting the reliability of the docking protocol for modeling non-covalent interactions. Galloyl–flavonoids **31**, **36**, **40**, **42**, and **44**, along with their parent compounds **7** and **9** and the low-potency compound **35**, were subjected to docking studies. In this study, the crystal structure of 3CL^pro^ covalently bound with the myricetin was used as the receptor, due to its pyrogallol group being similar to that of our compounds. The results revealed various possible binding poses with different orientations of the galloyl group in sub-pockets. The binding affinity of the galloyl derivatives ranged from −7.3 to −8.7 kcal/mol (Table S2), which were lower than those of the parent compounds pinocembrin (**7**) and DMC (**9**), with energies of −7.0 and −6.5 kcal/mol, respectively. The failure of docking to distinguish the activity difference between compounds **7** and **9** likely reflects a limitation in the current docking protocol. The use of a rigid receptor conformation derived from a covalently bound complex may not represent the optimal geometry for accommodating a non-covalently inhibitor, particularly in the case of larger substituents or alternative binding orientations. Since covalent bond formation can constrain and bias receptor geometry, docking non-covalent ligands into such a structure may yield misleading prediction. Additionally, AutoDock Vina has a typical error of ±2 kcal/mol. Thus, molecular docking alone cannot reliably distinguish between active and inactive compounds. Therefore, three possible binding poses that allow for induced-fit effects in key active site residues were selected as initial structures for MD simulations to address these limitations.

### 2.7. Molecular Dynamics Simulation

To explore the dynamics and stability of the protein–ligand complexes, three classical MD simulations were performed using distinct binding poses to enhance sampling efficiency. As a result, most of the ligands remained stable during the simulation timescale. The analysis of the protein–ligand complexes, including the RMSD of Cα, the RMSD of the ligand, the number of hydrogen bonds between the protein and ligand, the evolution of interaction free energy, principal component analysis (PCA), and the free energy landscape (FEL), is presented in Supplementary Material Section III.

**Figure 7:**
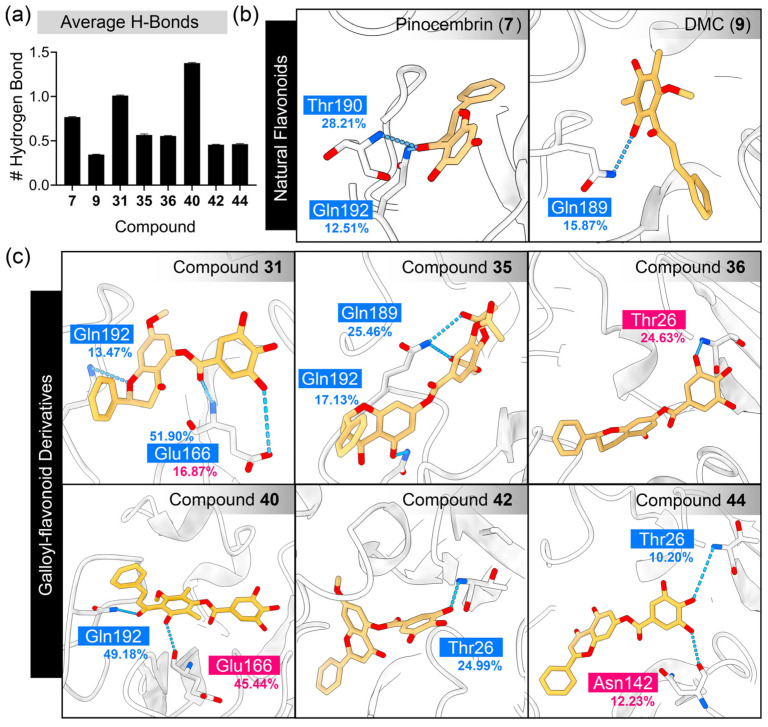
Analysis of hydrogen bond interactions between SARS-CoV-2 3CL^pro^ and flavonoid derivatives. (**a**) The average number of hydrogen bonds, plotted as the mean ± SEM, calculated from the last 150 ns of the simulation trajectory. Three-dimensional plots demonstrate hydrogen bonding at the atomistic level for (**b**) natural flavonoids and (**c**) galloyl–flavonoid derivatives. The protein is represented as a cartoon, while the interacting residues and ligands are shown using a stick model. The boxes highlight the residues contributing to hydrogen bonds, with cyan and magenta indicating ligands acting as hydrogen bond acceptors and donors, respectively.

In the MD simulation, three independent runs showed distinct protein trajectory paths caused by different initial ligand poses according to PCA. The trajectory with the strongest protein–ligand interaction free energy was selected for visualization. The most populated binding poses were obtained from clustering, and are depicted in Figure 7. Pinocembrin (**7**) aligned its chromanone ring with the S4 sub-pocket, while its monosubstituted phenyl ring aligned with the hydrophobic site in the S2 sub-pocket. DMC (**9**) favored alignment of its fully substituted aromatic ring to the S2 sub-pocket and of its monosubstituted phenyl ring to the S1 sub-pocket. The conformation with the fully substituted aromatic ring aligned to the S4 sub-pocket and the monosubstituted phenyl ring aligned to the S2 sub-pocket was less favored, as evidenced by the relative interaction free energy (Supplementary Material Figure S109). The presence of dimethyl groups at C-3′,5′ increased hydrophobic properties, enabling binding within the S2 sub-pocket. Bioactivity was significantly decreased when these methyl groups were removed, as observed in the case of cardamonin in our previous publication [34].

For the galloyl–flavonoid derivatives, the attachment of the galloyl group allowed the flavonoid inhibitors to bind within the S1′ sub-pocket. MM-GBSA calculations revealed that all compounds aligning the galloyl portion to the S1′ sub-pocket exhibited the lowest relative interaction free energies (Figures S108−S115). These results support the hypothesis that the galloyl group, being a strong hydrophilic portion, favors binding with the hydrophilic S1′ sub-pocket, consistent with previously reported crystal structures [31,32,33]. However, gallic acid alone (Supplementary Material Figure S116) failed to maintain stable binding within the binding site during the simulation timescale. This may be due to its small molecular size, which minimizes protein–ligand interactions. The potential of the galloyl moiety as a covalent inhibitor was assessed by measuring the distance between the thiolate nucleophile of Cys145 and the reactive Michael acceptor carbon of the galloyl group (Supplementary Material Figure S117). The results showed that all the galloyl–flavonoids exhibited a minimum distance of approximately 3.5 Å between these atoms, suggesting a plausible geometry for nucleophilic attack. While covalent bond formation was not modeled explicitly in this study, the maintained proximity supports the potential for covalent inhibition, consistent with previous findings for compounds **2**, **3**, and **4**.

Based on the structure of the candidate compounds, galloyl–flavonoids possess trihydroxy groups, which facilitate the formation of numerous hydrogen bonds with SARS-CoV-2 3CL^pro^. The number of hydrogen bonds and their occupancy percentage between 3CL^pro^ and the candidate compounds were monitored during the MD simulation, as depicted in Figure 7.

As a result, for the parent compounds, the inactive compound pinocembrin (**7**) exhibited a greater number of hydrogen bonds compared to the active compound DMC (**9**). The galloyl–flavonoid derivatives **31** and **40** displayed a high average number of hydrogen bonds, with values of 1.01 and 1.38, respectively. In contrast, other derivatives, such as **35**, **36**, **42**, and **44** exhibited relatively fewer hydrogen bonds. The residues within the S1′ and S4 sub-pockets were the primary contributors, particularly Thr26, Glu166, Gln189, Thr190, and Gln192. The galloyl group of galloyl–flavonoids **36**, **42**, and **44** interacted with Thr26 in the S1′ sub-pocket. However, compound **31** interacted with Glu166 in the S1 sub-pocket, while **40** did not form any hydrogen bonds with any residues. These findings suggest that the galloyl group did not directly enhance bioactivity by increasing hydrogen bonding interactions.

**Figure 8:**
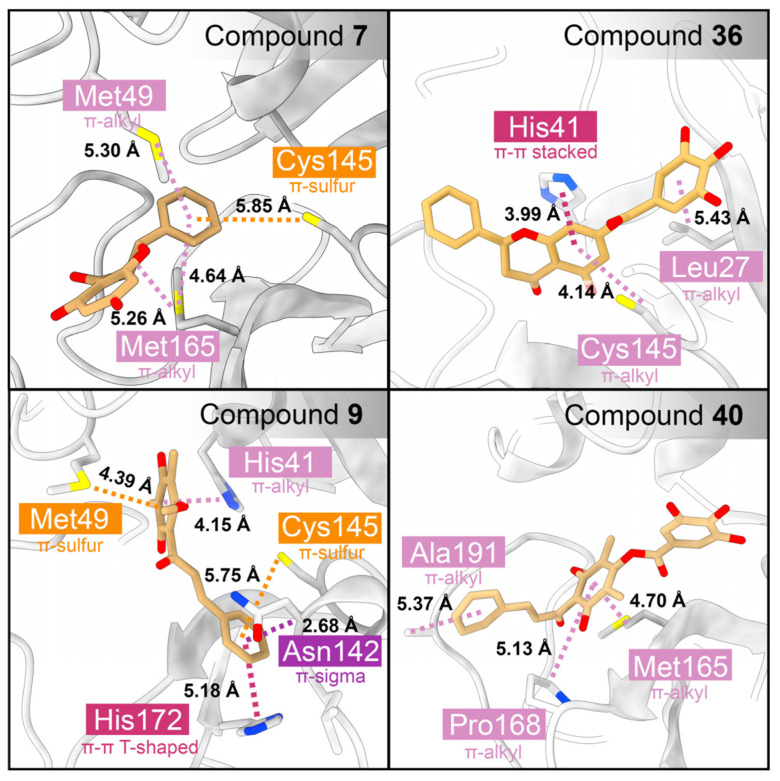
Analysis of π-interactions between SARS-CoV-2 3CL^pro^ and the flavonoid derivatives. The protein is represented by a cartoon, while the interacting residues and ligands are shown using a stick model. The boxes highlight the residues contributing to π-interactions, with orange, pink, and violet indicating residues involved in π-sulfur, π-alkyl, and π-sigma interactions, respectively.

In addition to hydrogen bonding, π-interactions caused by the aromatic ring of the galloyl moiety were observed (Figure 8). The contribution of the galloyl portion to both hydrogen bonds and π-interactions depended on the chemical structure of the parent compound. For compound **7**, the galloyl group did not increase the number of hydrogen bonds (Figure 7) but formed a π-alkyl interaction with Leu27. In contrast, the attachment of the galloyl group in **40** did not form any π-interactions, but did increase the number of hydrogen bonds. The predominant residues contributing to π-interactions in flavonoids included Leu27, His41, Met49, Cys145, and Met165.

To gain a deeper understanding of the thermodynamics of the binding interaction between flavonoids, their derivatives, and the SARS-CoV-2 3CL^pro^ enzyme, the relative interaction free energy was calculated using MM-GBSA, and the NMODE entropy was evaluated (Table 3). Pinocembrin (**7**), an inactive compound, exhibited the highest Δ*G*_bind_ compared to other active compounds. This compound demonstrated significantly lower van der Waals interactions, while other compounds showed similar values. Electrostatic interactions were stronger in compound **40**, corresponding to its strong hydrogen bond interactions. The −*T*Δ*S* term reflects the loss of freedom of the ligand upon binding with the receptor. Compound **7**, a smaller, more rigid compound, exhibited the lowest entropic energy. Although MM-GBSA interaction energies provided a general indication of binding strength, we note that the experimentally measured IC_50_ values of the studied compounds fall within a narrow range, and no clear quantitative correlation was observed. These calculations are thus intended to support qualitative trends in binding interactions, rather than to rank-order compounds by activity.

**Figure 9:**
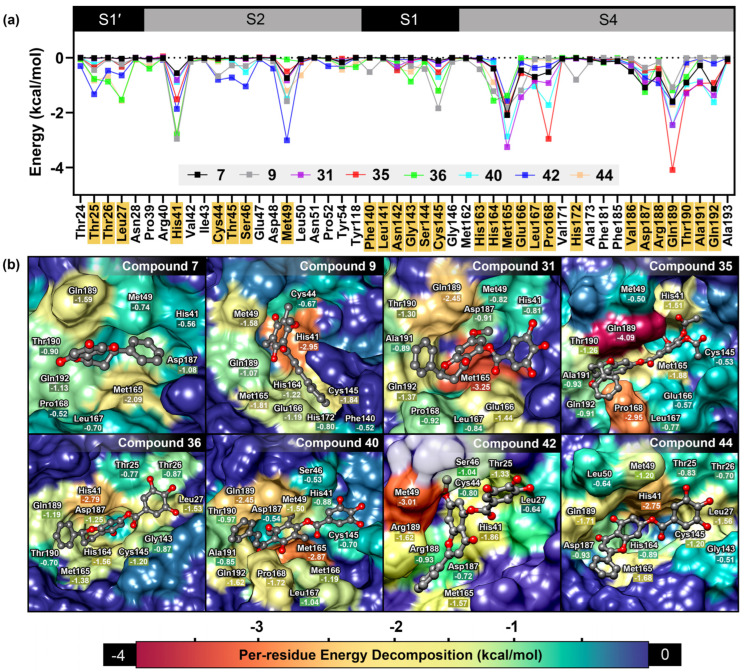
Analysis of MM-GBSA calculations. (**a**) Per-residue energy decomposition based on MM-GBSA calculations. The scatter plot illustrates the energy in each residue contributing to the binding affinity. Residues with an interaction energy lower than −0.5 kcal/mol are highlighted in yellow. (**b**) Three-dimensional structures of semi-synthetic 3CL^pro^ inhibitors at the substrate binding site of SARS-CoV-2 3CL^pro^. The surfaces are colored based on interaction energy values.

To gain deeper insights into protein–ligand interactions, per-residue energy decomposition analysis was performed (Figure 9). The binding profiles of the natural flavonoids and their derivatives exhibited similar trends. Key interaction hotspots were identified at the residues Thr25, Leu27, His41, Met49, Cys145, Met165, Pro168, Asp187, Gln189, and Gln192. The inactive natural flavonoid **7** showed no interactions with the S1 and S1′ sub-pockets, but exhibited strong interactions with the S4 sub-pocket, with slight support from the S2 sub-pocket. In comparison to other biologically active compounds, DMC (**9**) and galloyl–flavonoid derivatives **26**, **31**, **35**, **37**, and **39** displayed varying degrees of interaction with the S1, S1′, S2, and S4 sub-pockets. Notably, the profiles of interaction with the S4 sub-pocket remained largely unchanged. The attachment of the galloyl group facilitated interactions with the S1 sub-pocket, particularly with Thr25, Thr26, and Leu27, while also enhancing interactions with the key catalytic dyad His41 and Cys145 in the S1 and S2 sub-pockets.

Per-residue analysis further confirmed the role of the galloyl moiety in flavonoid scaffolds. In the case of **7**, no interactions were observed with the S1′ sub-pocket, only slight interactions with the S2 sub-pocket (His41 and Met49), and no interaction with the catalytic site Cys145 in the S1 sub-pocket. Additionally, this compound’s pattern of interaction with the S4 sub-pocket was similar to that of its galloylated derivative **36**.

To further investigate the π-interactions introduced by the galloyl moiety, FMO calculations were performed. Quantum mechanical calculations performed using the MP2 method provide a more accurate estimation of dispersion energy compared to molecular mechanics-based MM-GBSA calculations. The results (Table 4) showed good correlation with previous MM-GBSA results.

The calculated Δ*E*^int^ values effectively distinguished between inactive and active compounds, particularly between pinocembrin (**7**) and its galloylated derivative **36**. Compound **36** exhibited significantly stronger dispersion energy than pinocembrin (**7**) (ΔΔ*E*^di^ = 21.45 kcal/mol), which supported the presence of π-interactions, including π-alkyl, π-π stacked, and π-π T-shaped interactions. Furthermore, the decrease in electrostatic energy (Δ*E*^es^) and charge transfer and exchange energy (Δ*E*^ct+mix^) was attributed to the attachment of the galloyl group. In the case of **40**, the presence of the galloyl group resulted in a slight decrease in Δ*E*^di^ and Δ*E*^ct+mix^ energies, while leading to an increase in electrostatic energy (Δ*E*^es^).

## 3. Materials and Methods

### 3.1. Chemistry

Commercial solvents were purchased from local suppliers and distilled before use. Melting points were determined using a SANYO Gallenkamp electrical apparatus (SANYO Gallenkamp, Leicestershire, UK). Column chromatography was performed using Merck silica gel 60 (Merck KGaA, Darmstadt, Germany), while thin-layer chromatography (TLC) was conducted on Merck silica gel 60 PF_254_ aluminum plates (Merck KGaA, Darmstadt, Germany). The purity of the compounds was assessed using a PerkinElmer LC 300 UHPLC system (PerkinElmer, Waltham, MA, USA) equipped with a Brownlee Analytical C18 column (150 × 3 mm, 2.6 µm). An isocratic mobile phase of ACN/MeOH (90:10, *v*/*v*) was used at a flow rate of 0.2 mL/min. The column temperature was maintained at 25 °C, and detection was carried out using a UV detector set at 280 nm.

### 3.2. Spectroscopy

Fourier-transform infrared (FTIR) spectra were recorded on a Bruker INVENIO FTIR spectrometer (Bruker, Karlsruhe, Germany) using the attenuated total reflectance (ATR) technique, with absorption bands reported in reciprocal centimeters (cm^−1^). High-resolution mass spectra (HRMS) were obtained using an Agilent 6540 UHD QTOF mass spectrometer (Agilent Technologies, Santa Clara, CA, USA). Nuclear magnetic resonance (NMR) spectra were recorded on a Bruker Avance 500 NMR spectrometer (Bruker, Karlsruhe, Germany) using CDCl_3_, acetone-*d*_6_, and DMSO-*d*_6_ as solvents. Chemical shifts were calibrated using the residual solvent signals according to standard values [38]. Splitting patterns were reported as follows: s (singlet), d (doublet), t (triplet), dd (doublet of doublets), and m (multiplet). The coupling constants (*J*) were reported in hertz (Hz).

### 3.3. Isolation of Flavonoids

The flavonoid substrates, including pinostrobin (**6**), pinocembrin (**7**), cardamonin (**8**), and dimethylcardamonin (DMC, **9**), were isolated from natural sources, including *Syzygium nervosum* and *Boesenbergia rotunda*. Tectochrysin (**10**) and chrysin (**11**) were synthesized via iodine oxidation, following the procedure described in our previous publication [34].

### 3.4. Synthesis of Flavonoid Derivatives

The flavonoids were chemically modified through nitration and reduction, together with esterification with acrylic acid, benzoic acid, salicylic acid, vanillic acid, and gallic acid. Details of the synthesis procedures and spectroscopic characterization can be found in Supplementary Material Section I (Figures S1−S72).

### 3.5. Enzymatic Assay

A fluorescent resonance energy transfer (FRET) assay was employed to evaluate the enzymatic inhibition of the semi-synthetic derivatives, following the procedure described in our previous publication [34]. The untagged SARS-CoV-2 3CL^pro^ wild-type enzyme was purchased from Sigma-Aldrich (Merck KGaA, Darmstadt, Germany). Briefly, 10 µL of diluted SARS-CoV-2 3CL^pro^ (300 nM) solution in assay buffer (containing 20 mM Tris-HCL, 1 mM EDTA, 150 mM NaCl, and 1 mM DTT, pH 7.3) was pipetted into each well, which already contained 77 µL of assay buffer. Then, 1 µL of the tested compounds dissolved in DMSO, at various concentrations, was added to the wells. The mixture was incubated at 37 °C for 15 min. Subsequently, 12 µL of fluorogenic substrate (250 µM), Ac-Abu-Tle-Leu-Gln-MCA, was added to initiate the enzymatic reaction. In all the assays, the final concentrations of SARS-CoV-2 3CL^pro^ and the fluorogenic substrate were 30 nM and 30 µM, respectively. Fluorescence intensity was measured kinetically for 15 min using a SpectraMax i3x multi-mode microplate reader (Molecular Devices, San Jose, CA, USA), with excitation and emission wavelengths set to 380 nm and 455 nm, respectively. The changes in fluorescence intensity over time for each inhibitor concentration were directly related to the enzymatic activity, which was calculated using the following equation:

(1) $$\%\mathrm{inhibition}\;=\;\left[\frac{\left(\mathrm{control}\;-\;\mathrm{negative}\right)\;-\;(\mathrm{tested}\;\mathrm{inhibitor}\;-\;\mathrm{negative})}{\left(\mathrm{control}\;-\;\mathrm{negative}\right)}\right]\times 100\%$$

The percentage of enzymatic inhibition was calculated using GraphPad Prism version 9 software (GraphPad Software, Inc., La Jolla, CA, USA), employing nonlinear regression analysis. The inhibitory activity was expressed as the half-maximal inhibitory concentration (IC_50_).

### 3.6. Molecular Docking

The crystal structure of SARS-CoV-2 3CL^pro^ covalently bound to the flavonoid inhibitor myricetin (PDB ID: 7B3E) [31] was retrieved from the Protein Data Bank (RCSB) and used as the macromolecule for modeling. The protein structure was prepared using the Protein Preparation Tool in BIOVIA Discovery Studio 2021 (Dassault Systèmes: San Diego, CA, USA). Chain A was selected for modeling, and water molecules and ions were removed. The alternative conformer at the residue Cys128 was cleaned, and missing residues at the C-terminus were added. Hydrogen atoms were subsequently added, and the standard protonation state of titratable amino acid residues was corrected using the online PDB2PQR server [39], available at https://server.poissonboltzmann.org/pdb2pqr (accessed on 8 December 2024). The prepared protein structure was then exported in PDB format. The 3D structure of the ligand was constructed using Chem3D software 16 (PerkinElmer, Waltham, MA, USA), followed by preliminary energy minimization using molecular mechanics calculations. The protonation state of the ligand was corrected using MarvinSketch software (Chemaxon, Budapest, Hungary), calculated at pH 7.3. The structure was fully optimized using density functional theory (DFT) with the B3LYP functional and the 6-311++G(d,p) basis set, as implemented in the Gaussian16 package [40]. The optimized structure was exported in MOL2 format.

Charges were added to both the protein and ligand structures, and these were then converted to PDBQT format using AutoDock Tools. All single bonds in the ligand were set to allow free rotation. Molecular docking experiments were performed using AutoDock Vina [41]. The docking protocol was validated by redocking a co-crystallized ligand into the binding site (Figure S106). The center coordinates were defined as 23.239, 3.404, −27.169 for the x, y, and z axes. The grid size was set to 30 × 30 × 30 for the x, y, and z dimensions. The exhaustiveness was set to 300 for all predictions. The predicted binding poses were analyzed using BIOVIA Discovery studio [42].

### 3.7. Molecular Dynamics Simulation

The protein–ligand complex was prepared using the AmberTools23 package [43], employing three distinct binding poses obtained from molecular docking (Table S2). The protein structure was parameterized using the AMBER ff14SB force field [44]. The ligand charge was calculated based on electrostatic potential (ESP) using DFT calculations at the B3LYP/6-311++G(d,p) level of theory. The antechamber module was used for charge fitting with the restricted ESP model, followed by ligand parameterization using the general AMBER force field 2 (GAFF2). Using the tLEaP tool, the protein–ligand complex was placed into an octahedral box with a 12 Å distance between the protein surface and the box surface. The system was solvated with the TIP3P water model. The system was first neutralized by adding four sodium (Na^+^) ions. Then, 66 sodium (Na^+^) and 66 chloride (Cl^−^) ions were added to achieve a salt concentration of 0.15 M. The AMBER topology of the protein–ligand complex was converted into GROMACS topology using the ParmEd script.

Three independent molecular dynamics (MD) simulations were performed on GROMACS version 2022.5 [45], using NVIDIA A100 SXM4 GPUs for acceleration. The simulations were conducted on the HPE Cray EX235n supercomputer in the LANTA cluster. The system was energy-minimized using the steepest descent algorithm with energy tolerances of 10 kJ/mol/nm, followed by conjugate gradient minimization. The initial velocity was generated randomly in each simulation using random seed values, as depicted in Table S3. Equilibration was conducted in the NVT ensemble for 1000 ps, followed by the NPT ensemble for 1000 ps. The ligand position was restrained during equilibration with a force constant of 1000 kJ/mol/nm^2^ in the x, y, and z directions. The LINCS algorithm was applied to constrain bond lengths involving hydrogen atoms. The modified Berendsen thermostat and Parrinello–Rahman barostat were used for temperature and pressure coupling, with time constants of 0.1 and 0.2 ps, respectively. Electrostatic interactions were calculated using the Particle-Mesh Ewald (PME) method with a cutoff of 12 Å and with van der Waals interactions cut off at 12 Å, along with a force-switch modifier. Production runs were carried out with an unrestrained system for 300 ns at 310 K and 1 atm pressure, with a timestep of 2 fs. The trajectory was saved every 10 ps.

The dynamics of the protein–ligand complex were monitored using the RMSD of the Cα atoms and ligand with the gmx_rms command. The number of hydrogen bonds was assessed using the gmx_hbond module and hydrogen bond analysis in VMD software, with the following criteria: a distance between donor and acceptor atoms of ≤3.5 Å and an angle cutoff of 20°. Principal component analysis (PCA) was performed based on the eigenvectors of the covariance matrix of Cα atoms using the gmx_covar command. The two highest eigenvectors were analyzed with the gmx_anaeig module, projected onto the PC1 and PC2 axes. The free energy landscape (FEL) was calculated using the gmx_sham command. Clustering analysis was performed on the Cα atoms using the gmx_cluster module with the GROMOS method and a cutoff value of 1.5 Å. All graphs were generated using custom Python scripts based on Matplotlib version 3.9.0 and Seaborn version 0.13.0.

### 3.8. Interaction Free Energy Calculation

Molecular mechanics with generalized Born surface area (MM-GBSA) and normal-mode entropy calculations were performed using the gmx_MMPBSA package [46]. The calculations were performed using the single-trajectory approach, with all snapshots extracted from the protein–ligand trajectory. For all protein–ligand complexes, the trajectory of 30 ns extracted from the equilibrium state was selected for analysis. To reduce computational cost, only the last 2.5 ns was used for calculating the normal-mode entropy. The Modified GB model 2 (*igb* = 5), developed by A. Onufriev et al. [47], was employed as the polar solvation model. The internal (*intdiel*) and external (*extdiel*) dielectric constants were set to 2 and 78.5, respectively. The salt concentration was defined as 0.15 M, and the solvent prob radius was set to 1.4 Å. The relative interaction free energy of the protein–ligand complexes was calculated using the following equation:

(2) $${∆G}_{\mathrm{int}}={∆E}_{\mathrm{MM}}-T∆S+{∆G}_{\mathrm{sol}}$$

(3) $${∆E}_{\mathrm{MM}}={∆E}_{\mathrm{vdw}\;}+{∆E}_{\mathrm{elec}\;}$$

(4) $${∆G}_{\mathrm{sol}}={∆G}_{\mathrm{polar}\;}+{∆G}_{\mathrm{nonpolar}\;}$$

### 3.9. Fragment Molecular Orbital (FMO) Calculation

The relative interaction free energy based on quantum mechanics (QM) calculations was determined using the FMO code version 5.5, embedded within the General Atomic and Molecular Electronic Structure System (GAMESS) software version 2024R2 [48]. The structures of protein–ligand complexes obtained from clustering molecular dynamics trajectories were used for the calculations. Clustering was performed using the GROMOS algorithm based on the RMSD of the Cα atoms of the protein structure, with a cutoff value of 1.5 Å. The most populated cluster was selected for each system. The centroid structure of this cluster was extracted and used as the representative protein–ligand conformation for calculation. The complex underwent energy minimization using the AMBER10:EHT force field in the Molecular Operating Environment (MOE) 2015 package, with an energy tolerance of 0.001 kcal/mol. The amino acids within 7 Å of the ligand were selected for calculating the pair interaction energy (PIE, ${∆E}^{\mathrm{int}}$) using the MP2 method (second-order Møller–Plesset perturbation theory), with a 6-31G* basis set and the polarizable continuum model (PCM) for solvation. The interaction between specific residues and the ligand was decomposed into interaction energy decomposition analysis (PIEDA). The PIE (${∆E}_{ij}^{\mathrm{int}}$) between fragments *i* and *j* consists of five energy components: electrostatics (${∆E}^{\mathrm{es}}$), exchange repulsion (${∆E}^{\mathrm{ex}}$), charge transfer (${∆E}^{\mathrm{ct}+\mathrm{mix}}$), dispersion interaction (${∆E}^{\mathrm{di}}$), and solvation (${∆G}^{\mathrm{sol}}$) energies.

(5) $${∆E}_{ij}^{\mathrm{int}}={∆E}_{ij}^{\mathrm{es}}+{∆E}_{ij}^{\mathrm{ex}}+{∆E}_{ij}^{\mathrm{ct}+\mathrm{mix}}+{∆E}_{ij}^{\mathrm{di}}+{∆G}^{\mathrm{sol}}$$

## 4. Conclusions

In summary, the conjugation of flavonoids with a galloyl moiety via ester linkers substantially improved their inhibitory activity against SARS-CoV-2 3CL^pro^, with up to a 23-fold increase in potency observed for pinostrobin (**6**). Among these, 4′-*O*-galloyl-DMC (**40**) emerged as the most potent inhibitor, highlighting the galloyl group as a critical pharmacophoric element for effective binding within the S1′ sub-pocket. Molecular dynamics simulations further corroborated these findings, demonstrating stable interactions mediated by hydrogen bonding and π-interactions with Thr25, Thr26, and Leu27. These results provide a rational framework for the design of galloyl-based flavonoid derivatives as promising antiviral candidates targeting SARS-CoV-2 3CL^pro^.

## Figures and Tables

**Table 1:** Summary of crystal structures of SARS-CoV-2 3CL^pro^–ligand complexes analyzed by FMO calculation.

No.	PDB ID	Ligand	Binding Pocket	Resolution (Å)	Δ*E*^int^ (kcal/mol) ^1^
1	6M2N	baicalein (**1**)	S1	2.20	−38.16
2	7DPP	myricetin (**2**)	S1′	2.10	−63.43
3	7DPU	7-*O*-methylmyricetin (**3**)	S1′	1.75	−53.56
4	8HI9	robinetin (**4**)	S1′	2.28	−48.24

^1^ Pair interaction energy was calculated at FMO2-RIMP2-6-31G (d) level of theory with PCM solvation model.

**Table 2:** Enzymatic inhibitory activity against SARS-CoV-2 3CL^pro^.

Cpd. No	Name	SARS-CoV-2 3CL^pro^ Inhibition	
		100 µM (%)	IC_50_ (µM)
**6**	pinostrobin	26.44 ± 2.32 ^3^	>200 ^3^
**7**	pinocembrin	27.18 ± 2.20 ^3^	>200 ^3^
**8**	cardamonin	85.60 ± 2.44 ^3^	57.42 ^3^
**9**	DMC	84.59 ± 0.84 ^3^	27.54 ^3^
**10**	tectochrysin	86.39 ± 0.19 ^3^	24.00 ^3^
**11**	chrysin	87.73 ± 0.66 ^3^	32.84 ^3^
**12**	5-*O*-acryloylpinostrobin	92.57 ± 0.52 ^3^	ND
**13**	7-*O*-acryloylpinocembrin	79.25 ± 1.52	ND
**14**	5,7-*O*-diacryloylpinocembrin	76.24 ± 0.97	ND
**15**	4′-*O*-acryloylcardamonin	66.57 ± 2.05	ND
**16**	4′-*O*-acryloyl-DMC	52.90 ± 1.17	ND
**17**	2′,4′-*O*-diacryloylcardamonin	76.85 ± 4.85	ND
**18**	2′,4′-*O*-diacryloyl-DMC	68.00 ± 3.77	ND
**19**	5-*O*-acryloyltectochrysin	37.21 ± 2.09	>100
**20**	7-*O*-acryloylchrysin	84.01 ± 0.82	ND
**21**	5,7-*O*-diacryloylchrysin	32.32 ± 4.83	>100
**22**	8-nitropinostrobin	54.95 ± 3.96	ND
**23**	6-nitropinostrobin	53.45 ± 4.31	ND
**24**	6,8-dinitropinostrobin	40.01 ± 3.27	>100
**25**	8-aminopinostrobin	38.17 ± 3.74	>100
**26**	6-aminopinostrobin	14.70 ± 5.85	>100
**27**	5-*O*-benzoylpinostrobin	43.37 ± 3.99	>100
**28**	5-*O*-(*O*-acetylsalicyloyl)pinostrobin	89.73 ± 5.92	ND
**29**	5-*O*-(*O*-acetylvanilloyl)pinostrobin	84.38 ± 3.91	ND
**30**	5-*O*-(tri-*O*-acetylgalloyl)pinostrobin	67.09 ± 2.93	ND
**31**	5-*O*-galloylpinostrobin	99.82 ± 0.15	7.42
**32**	7-*O*-benzoylpinocembrin	70.77 ± 2.72	ND
**33**	7-*O*-(*O*-acetylsalicyloyl)pinocembrin	81.70 ± 0.89	ND
**34**	7-*O*-(*O*-acetylvanilloyl)pinocembrin	83.70 ± 3.37	ND
**35**	7-*O*-(tri-*O*-acetylgalloyl)pinocembrin	72.97 ± 1.66	ND
**36**	7-*O*-galloylpinocembrin	99.54 ± 1.34	7.53
**37**	4′-*O*-(tri-*O*-acetylgalloyl)cardamonin	87.17 ± 3.25	ND
**38**	4′-*O*-galloylcardamonin	99.59 ± 0.41	13.34
**39**	4′-*O*-(tri-*O*-acetylgalloyl)-DMC	71.95 ± 4.27	ND
**40**	4′-*O*-galloyl-DMC	99.92 ± 0.11	6.85
**41**	5-*O*-(tri-*O*-acetylgalloyl)tectochrysin	72.81 ± 2.43	ND
**42**	5-*O*-galloyltectochrysin	99.79 ± 0.80	20.58
**43**	7-*O*-(tri-*O*-acetylgalloyl)chrysin	85.75 ± 0.48	ND
**44**	7-*O*-galloylchrysin	99.46 ± 1.79	9.26
	gallic acid	1.72 ± 12.05	>100
	baicalein (+) ^1^	62.95 ± 3.10 ^3^	86.57 ^3^
	nirmatrelvir (+) ^2^	99.92 ± 0.62	ND

ND = not determined. ^1^ Baicalein was used as a positive control flavonoid with 3CL^pro^ inhibitory activity. ^2^ Nirmatrelvir was used as a positive medication for COVID-19 treatment targeting 3CL^pro^. ^3^ The IC_50_ values reported are in accordance with one of our previous publications [34].

**Table 3:** The relative interaction free energy between flavonoids and SARS-CoV-2 3CL^pro^, calculated using MM-GBSA and NMODE entropy calculations.

Cpd.	Free Energy (kcal/mol) ^1^					
	Δ*E*_vdw_	Δ*E*_elec_	Δ*G*_polar_	Δ*G*_nonpolar_	−*T*Δ*S*	Δ*G*_int_
**7**	−26.06 ± 3.63	−7.88 ± 3.41	11.39 ± 2.37	−3.61 ± 0.38	19.40 ± 3.58	−6.75 ± 5.14
**9**	−40.74 ± 3.14	−3.33 ± 1.68	9.36 ± 1.14	−4.95 ± 0.25	25.21 ± 5.68	−14.45 ± 6.48
**31**	−39.62 ± 2.81	−4.22 ± 3.11	10.35 ± 2.60	−4.51 ± 0.27	23.02 ± 3.14	−14.98 ± 4.16
**35**	−43.62 ± 3.18	−7.17 ± 3.06	17.23 ± 2.37	−5.59 ± 0.35	23.25 ± 4.12	−15.90 ± 5.04
**36**	−40.01 ± 2.57	−4.46 ± 2.65	13.84 ± 1.78	−5.17 ± 0.27	23.12 ± 3.76	−12.67 ± 4.65
**40**	−39.92 ± 3.41	−13.30 ± 2.90	17.97 ± 1.88	−5.78 ± 0.39	22.74 ± 3.73	−18.30 ± 5.05
**42**	−42.74 ± 3.59	−2.43 ± 1.96	10.84 ± 1.68	−5.42 ± 0.37	23.66 ± 4.37	−16.09 ± 5.68
**44**	−39.22 ± 3.30	−2.67 ± 2.01	11.70 ± 1.53	−5.05 ± 0.32	20.37 ± 4.92	−14.87 ± 5.97

^1^ All energies are reported as the mean ± SD.

**Table 4:** The relative interaction free energy between flavonoids and SARS-CoV-2 3CL^pro^, obtained using FMO calculations.

Cpd.	Energy (kcal/mol) ^1^					
	Δ*E*^es^	Δ*E*^ex^	Δ*E*^ct+mix^	Δ*E*^di^	Δ*G*^sol^	Δ*E*^int^
**7**	−12.97	28.04	−13.39	−41.51	3.81	−36.02
**9**	−33.70	44.30	−14.83	−63.85	7.28	−60.80
**36**	−18.19	43.80	−20.71	−62.96	7.41	−50.64
**40**	−25.00	48.59	−20.66	−69.59	5.11	−61.56

^1^ Energy was calculated at FMO2-RIMP2-6-31G (d) level of theory with PCM solvation model.

## Data Availability

All data are provided within the article.

## Supplementary Materials

The following supporting information can be downloaded at https://www.mdpi.com/article/10.3390/ijms26199742/s1.
