# Supplementary material for: Discovery of Galloyl–Flavonoid Conjugates as SARS-CoV-2 3CLpro Inhibitors: Understanding Binding Interactions Through Computational Approaches
Source: Int J Mol Sci. 2025 Oct 7;26(19):9742. doi: 10.3390/ijms26199742 (PMC12524796; doi:10.3390/ijms26199742)
Supplement: Supplementary file 1 [file ijms-26-09742-s001.zip › ijms-3901135-supplementary.pdf]

Supplementary Material

for

Discovery of Galloyl-Flavonoid Conjugates as SARS-CoV-2 3CL^{pro} Inhibitors: Understanding Binding Interactions through Computational Approaches

Nopawit Khamto [1,2,3]*, Panida Boontawee [4,5], Vachira Choommongkol [6], Kritsada Pruksaphon [7], Suwicha Patnin [8], Nuttee Suree [4,9], Panchika Prangkio [4,9] and Puttinan Meepowpan [4,9,10]*

[1]*Department of Biochemistry, Faculty of Medical Science, Naresuan University, Phitsanulok 65000, Thailand*

[2]*Center of Excellence in Medical Biotechnology (CEMB), Faculty of Medical Science, Naresuan University, Phitsanulok 65000, Thailand*

[3]*Cellular and Molecular Immunology Research Unit, Faculty of Allied Health Sciences, Naresuan University, Phitsanulok 65000, Thailand*

[4]*Department of Chemistry, Faculty of Science, Chiang Mai University, Chiang Mai 50200, Thailand*

[5]*Multidisciplinary and Interdisciplinary School, Chiang Mai University, Chiang Mai 50200, Thailand*

[6]*Department of Chemistry, Faculty of Science, Maejo University, Chiang Mai 50290, Thailand*

[7]*Center of Excellence Research for Melioidosis and Microorganisms (CERMM), Walailak University, Nakhon Si Thammarat 80160, Thailand*

[8]*Laboratory of Organic Synthesis, Chulabhorn Research Institute, Bangkok 10210, Thailand*

[9]*Center of Excellence in Materials Science and Technology, Chiang Mai University, Chiang Mai 50200, Thailand*

[10]*Center of Excellence for Innovation in Chemistry (PERCH-CIC), Faculty of Science, Chiang Mai University, Chiang Mai 50200, Thailand*

**Correspondence: nopawitk@nu.ac.th (N.K.) and puttinan.m@cmu.ac.th (P.M.)*

Table of Content

Contents		Page
**Section I.** Synthesis and Characterization of Inhibitors		
**Figure S1**	^{1}H-NMR spectrum (500 MHz, $CDCl_3$) of acetylsalicylic acid	S27
**Figure S2**	^{13}C-NMR, DEPT90, and DEPT135 spectra (125 MHz, $CDCl_3$) of acetylsalicylic acid	S27
**Figure S3**	^{1}H-NMR spectrum (500 MHz, $CDCl_3$) of acetylvanilic acid	S28
**Figure S4**	^{13}C-NMR, DEPT90, and DEPT135 spectra (125 MHz, $CDCl_3$) of acetylvanilic acid	S28
**Figure S5**	^{1}H-NMR spectrum (500 MHz, $CDCl_3$) of triacetylgallic acid	S29
**Figure S6**	^{13}C-NMR, DEPT90, and DEPT135 spectra (125 MHz, $CDCl_3$) of triacetyl gallic acid	S29
**Figure S7**	^{1}H-NMR spectrum (500 MHz, $CDCl_3$) of 5-*O*-acryloylpinostrobin (**12**)	S30
**Figure S8**	^{13}C-NMR, DEPT90, and DEPT135 spectra (125 MHz, $CDCl_3$) of 5-*O*-acryloylpinostrobin (**12**)	S30
**Figure S9**	^{1}H-NMR spectrum (500 MHz, $CDCl_3$) of 7-*O*-acryloylpinocembrin (**13**)	S31
**Figure S10**	^{13}C-NMR, DEPT90, and DEPT135 spectra (125 MHz, $CDCl_3$) of 7-*O*-acryloylpinocembrin (**13**)	S31
**Figure S11**	^{1}H-NMR spectrum (500 MHz, $CDCl_3$) of 5,7-*O*-diacryloylpinocembrin (**14**)	S32
**Figure S12**	^{13}C-NMR, DEPT90, and DEPT135 spectra (125 MHz, $CDCl_3$) of 5,7-*O*-diacryloylpinocembrin (**14**)	S32
**Figure S13**	^{1}H-NMR spectrum (500 MHz, $CDCl_3$) of 4′-*O*-acryloylcardamonin (**15**)	S33
**Figure S14**	^{13}C-NMR, DEPT90, and DEPT135 spectra (125 MHz, $CDCl_3$) of 4′-*O*-acryloylcardamonin (**15**)	S33
**Figure S15**	^{1}H-NMR spectrum (500 MHz, $CDCl_3$) of 4′-*O*-acryloyl-DMC (**16**)	S34
**Figure S16**	^{13}C-NMR, DEPT90, and DEPT135 spectra (125 MHz, $CDCl_3$) of 4′-*O*-acryloyl-DMC (**16**)	S34
**Figure S17**	^{1}H-NMR spectrum (500 MHz, $CDCl_3$) of 2′,4′-*O*-diacryloylcardamonin (**17**)	S35
**Figure S18**	^{13}C-NMR, DEPT90, and DEPT135 spectra (125 MHz, $CDCl_3$) of 2′,4′-*O*-diacryloylcardamonin (**17**)	S35
**Figure S19**	^{1}H-NMR spectrum (500 MHz, $CDCl_3$) of 2′,4′-*O*-diacryloyl-DMC (**18**)	S36
**Figure S20**	^{13}C-NMR, DEPT90, and DEPT135 spectra (125 MHz, $CDCl_3$) of 2′,4′-*O*-diacryloyl-DMC (**18**)	S36
**Figure S21**	^{1}H-NMR spectrum (500 MHz, $CDCl_3$) of 5-*O*-acryloyltectochrysin (**19**)	S37
**Figure S22**	^{13}C-NMR, DEPT90, and DEPT135 spectra (125 MHz, $CDCl_3$) of 5-*O*-acryloyltectochrysin (**19**)	S37

Table of Content

Contents		Page
**Figure S23**	^{1}H-NMR spectrum (500 MHz, $CDCl_3$) of 7-*O*-acryloylchrysin (**20**)	S38
**Figure S24**	^{13}C-NMR, DEPT90, and DEPT135 spectra (125 MHz, $CDCl_3$) of 7-*O*-acryloylchrysin (**20**)	S38
**Figure S25**	^{1}H-NMR spectrum (500 MHz, $CDCl_3$) of 5,7-*O*-diacryloylchrysin (**21**)	S39
**Figure S26**	^{13}C-NMR, DEPT90, and DEPT135 spectra (125 MHz, $CDCl_3$) of 5,7-*O*-diacryloylchrysin (**21**)	S39
**Figure S27**	^{1}H-NMR spectrum (500 MHz, $CDCl_3$) of 8-nitropinostrobin (**22**)	S40
**Figure S28**	^{13}C-NMR, DEPT90, and DEPT135 spectra (125 MHz, $CDCl_3$) of 8-nitropinostrobin (**22**)	S40
**Figure S29**	^{1}H-NMR spectrum (500 MHz, $CDCl_3$) of 6-nitropinostrobin (**23**)	S41
**Figure S30**	^{13}C-NMR, DEPT90, and DEPT135 spectra (125 MHz, $CDCl_3$) of 6-nitropinostrobin (**23**)	S41
**Figure S31**	^{1}H-NMR spectrum (500 MHz, $CDCl_3$) of 6,8-dinitropinostrobin (**24**)	S42
**Figure S32**	^{13}C-NMR, DEPT90, and DEPT135 spectra (125 MHz, $CDCl_3$) of 6,8-dinitropinostrobin (**24**)	S42
**Figure S33**	^{1}H-NMR spectrum (500 MHz, $CDCl_3$) of 8-aminopinostrobin (**25**)	S43
**Figure S34**	^{13}C-NMR, DEPT90, and DEPT135 spectra (125 MHz, $CDCl_3$) of 8-aminopinostrobin (**25**)	S43
**Figure S35**	^{1}H-NMR spectrum (500 MHz, $CDCl_3$) of 6-aminopinostrobin (**26**)	S44
**Figure S36**	^{13}C-NMR, DEPT90, and DEPT135 spectra (125 MHz, $CDCl_3$) of 6-aminopinostrobin (**26**)	S44
**Figure S37**	^{1}H-NMR spectrum (500 MHz, $CDCl_3$) of 5-*O*-benzoylpinostrobin (**27**)	S45
**Figure S38**	^{13}C-NMR, DEPT90, and DEPT135 spectra (125 MHz, $CDCl_3$) of 5-*O*-benzoylpinostrobin (**27**)	S45
**Figure S39**	^{1}H-NMR spectrum (500 MHz, $CDCl_3$) of 5-*O*-(*O*-acetylsalicyloyl)pinostrobin (**28**)	S46
**Figure S40**	^{13}C-NMR, DEPT90, and DEPT135 spectra (125 MHz, $CDCl_3$) of 5-*O*-(*O*-acetylsalicyloyl)pinostrobin (**28**)	S46
**Figure S41**	^{1}H-NMR spectrum (500 MHz, $CDCl_3$) of 5-*O*-(*O*-acetylvanilloyl)pinostrobin (**29**)	S47
**Figure S42**	^{13}C-NMR, DEPT90, and DEPT135 spectra (125 MHz, $CDCl_3$) of 5-*O*-(*O*-acetylvanilloyl)pinostrobin (**29**)	S47
**Figure S43**	^{1}H-NMR spectrum (500 MHz, $CDCl_3$) of 5-*O*-(tri-*O*-acetylgalloyl)pinostrobin (**30**)	S48
**Figure S44**	^{13}C-NMR, DEPT90, and DEPT135 spectra (125 MHz, $CDCl_3$) of 5-*O*-(tri-*O*-acetylgalloyl)pinostrobin (**30**)	S48
**Figure S45**	^{1}H-NMR spectrum (500 MHz, acetone-d_6) of 5-*O*-galloylpinostrobin (**31**)	S49

Table of Content

Contents		Page
**Figure S46**	^{13}C-NMR, DEPT90, and DEPT135 spectra (125 MHz, acetone-d_6) of 5-*O*-galloylpinostrobin (**31**)	S49
**Figure S47**	^{1}H-NMR spectrum (500 MHz, $CDCl_3$) of 7-*O*-benzoylpinocembrin (**32**)	S50
**Figure S48**	^{13}C-NMR, DEPT90, and DEPT135 spectra (125 MHz, $CDCl_3$) of 7-*O*-benzoylpinocembrin (**32**)	S50
**Figure S49**	^{1}H-NMR spectrum (500 MHz, $CDCl_3$) of 7-*O*-(*O*-acetylsalicyloyl)pinocembrin (**33**)	S51
**Figure S50**	^{13}C-NMR, DEPT90, and DEPT135 spectra (125 MHz, $CDCl_3$) of 7-*O*-(*O*-acetylsalicyloyl)pinocembrin (**33**)	S51
**Figure S51**	^{1}H-NMR spectrum (500 MHz, $CDCl_3$) of 7-*O*-(*O*-acetylvanilloyl)pinocembrin (**34**)	S52
**Figure S52**	^{13}C-NMR, DEPT90, and DEPT135 spectra (125 MHz, $CDCl_3$) of 7-*O*-(*O*-acetylvanilloyl)pinocembrin (**34**)	S52
**Figure S53**	^{1}H-NMR spectrum (500 MHz, $CDCl_3$) of 7-*O*-(tri-*O*-acetylgalloyl)pinocembrin (**35**)	S53
**Figure S54**	^{13}C-NMR, DEPT90, and DEPT135 spectra (125 MHz, $CDCl_3$) of 7-*O*-(tri-*O*-acetylgalloyl)pinocembrin (**35**)	S53
**Figure S55**	^{1}H-NMR spectrum (500 MHz, acetone-d_6) of 7-*O*-galloylpinocembrin (**36**)	S54
**Figure S56**	^{13}C-NMR, DEPT90, and DEPT135 spectra (125 MHz, acetone-d_6) of 7-*O*-galloylpinocembrin (**36**)	S54
**Figure S57**	^{1}H-NMR spectrum (500 MHz, $CDCl_3$) of 4′-*O*-(tri-*O*-acetylgalloyl)cardamonin (**37**)	S55
**Figure S58**	^{13}C-NMR, DEPT90, and DEPT135 spectra (125 MHz, $CDCl_3$) of 4′-*O*-(tri-*O*-acetylgalloyl)cardamonin (**37**)	S55
**Figure S59**	^{1}H-NMR spectrum (500 MHz, acetone-d_6) of 4′-*O*-galloylcardamonin (**38**)	S56
**Figure S60**	^{13}C-NMR, DEPT90, and DEPT135 spectra (125 MHz, $CDCl_3$) of 4′-*O*-galloylcardamonin (**38**)	S56
**Figure S61**	^{1}H-NMR spectrum (500 MHz, $CDCl_3$) of 4′-*O*-(tri-*O*-acetylgalloyl)-DMC (**39**)	S57
**Figure S62**	^{13}C-NMR, DEPT90, and DEPT135 spectra (125 MHz, $CDCl_3$) of 4′-*O*-(tri-*O*-acetylgalloyl)-DMC (**39**)	S57
**Figure S63**	^{1}H-NMR spectrum (500 MHz, $CDCl_3$) of 4′-*O*-galloyl-DMC (**40**)	S58
**Figure S64**	^{13}C-NMR, DEPT90, and DEPT135 spectra (125 MHz, $CDCl_3$) of 4′-*O*-galloyl-DMC (**40**)	S58
**Figure S65**	^{1}H-NMR spectrum (500 MHz, $CDCl_3$) of 5-*O*-(tri-*O*-acetylgalloyl)tectochrysin (**41**)	S59
**Figure S66**	^{13}C-NMR, DEPT90, and DEPT135 spectra (125 MHz, $CDCl_3$) of 5-*O*-(tri-*O*-acetylgalloyl)tectochrysin (**41**)	S59

Table of Content

Contents		Page
**Figure S67**	^{1}H-NMR spectrum (500 MHz, DMSO-d_6) of 5-*O*-galloyltectochrysin (**42**)	S60
**Figure S68**	^{13}C-NMR, DEPT90, and DEPT135 spectra (125 MHz, DMSO-d_6) of 5-*O*-galloyltectochrysin (**42**)	S60
**Figure S69**	^{1}H-NMR spectrum (500 MHz, $CDCl_3$) of **7**-*O*-(tri-*O*-acetylgalloyl)chrysin (**43**)	S61
**Figure S70**	^{13}C-NMR, DEPT90, and DEPT135 spectra (125 MHz, DMSO-d_6) of **7**-*O*-(tri-*O*-acetylgalloyl)chrysin (**43**)	S61
**Figure S71**	^{1}H-NMR spectrum (500 MHz, DMSO-d_6) of 7-*O*-galloylchrysin (**44**)	S62
**Figure S72**	^{13}C-NMR, DEPT90, and DEPT135 spectra (125 MHz, DMSO-d_6) of 7-*O*-galloylchrysin (**44**)	S62
**Figure S73**	HPLC chromatogram of **12** (Purity > 99.9%)	S63
**Figure S74**	HPLC chromatogram of **13** (Purity > 99.9%)	S63
**Figure S75**	HPLC chromatogram of **14** (Purity 99.8%)	S63
**Figure S76**	HPLC chromatogram of **15** (Purity 95.0%)	S63
**Figure S77**	HPLC chromatogram of **16** (Purity 97.5%)	S63
**Figure S78**	HPLC chromatogram of **17** (Purity >99.9%)	S64
**Figure S79**	HPLC chromatogram of **18** (Purity >99.9%)	S64
**Figure S80**	HPLC chromatogram of **19** (Purity >95.0%)	S64
**Figure S81**	HPLC chromatogram of **20** (Purity >99.5%)	S64
**Figure S82**	HPLC chromatogram of **21** (Purity >99.9%)	S64
**Figure S83**	HPLC chromatogram of **22** (Purity > 99.9%)	S65
**Figure S84**	HPLC chromatogram of **23** (Purity > 99.9%)	S65
**Figure S85**	HPLC chromatogram of **24** (Purity > 99.9%)	S65
**Figure S86**	HPLC chromatogram of **25** (Purity > 99.9%)	S65
**Figure S87**	HPLC chromatogram of **26** (Purity 97.8%)	S65
**Figure S88**	HPLC chromatogram of **27** (Purity 99.2%)	S66
**Figure S89**	HPLC chromatogram of **28** (Purity 99.2%)	S66
**Figure S90**	HPLC chromatogram of **29** (Purity 99.8%)	S66
**Figure S91**	HPLC chromatogram of **30** (Purity > 99.9%)	S66
**Figure S92**	HPLC chromatogram of **31** (Purity > 99.9%)	S66
**Figure S93**	HPLC chromatogram of **32** (Purity > 99.9%)	S67
**Figure S94**	HPLC chromatogram of **33** (Purity > 99.9%)	S67
**Figure S95**	HPLC chromatogram of **34** (Purity > 99.9%)	S67
**Figure S96**	HPLC chromatogram of **35** (Purity > 99.9%)	S67
**Figure S97**	HPLC chromatogram of **35** (Purity > 99.9%)	S67
**Figure S98**	HPLC chromatogram of **37** (Purity 99.2%)	S68
**Figure S99**	HPLC chromatogram of **38** (Purity 98.3%)	S68
**Figure S100**	HPLC chromatogram of **39** (Purity 95.7%)	S68
**Figure S101**	HPLC chromatogram of **40** (Purity 95.0%)	S68

Table of Content

Contents		Page
**Figure S102**	HPLC chromatogram of **41** (Purity 98.2%)	S68
**Figure S103**	HPLC chromatogram of **42** (Purity 97.7%)	S69
**Figure S104**	HPLC chromatogram of **43** (Purity > 99.9%)	S69
**Figure S105**	HPLC chromatogram of **44** (Purity 99.3%)	S69
**Section II.** SMILES format of compounds		
**Table S1**	Chemical structures and their SMILES	S71
**Section III.** Computational Simulations		
**Table S2**	The molecular docking results and binding poses for MD simulations at the binding site of SARS-CoV-2 3CL^{pro}	S78
**Table S3**	Seed values for molecular dynamics simulation	S82
**Figure S106**	Validation of molecular docking protocol	S78
**Figure S107**	Analysis of three independent simulations of 300 ns molecular dynamics simulations of SARS-CoV-2 3CL^{pro} in apo form. The analysis includes RMSD of protein Cα, principal component analysis (PCA), and free energy landscape (FEL)	S82
**Figure S108**	Analysis of three independent simulations of 300 ns molecular dynamics simulations of pinocembrin (**7**)	S83
**Figure S109**	Analysis of three independent simulations of 300 ns molecular dynamics simulations of DMC (**9**)	S84
**Figure S110**	Analysis of three independent simulations of 300 ns molecular dynamics simulations of 5-*O*-galloylpinostrobin (**31**)	S85
**Figure S111**	Analysis of three independent simulations of 300 ns molecular dynamics simulations of 7-*O*-(tri-*O*-acetylgalloyl)pinocembrin (**35**)	S86
**Figure S112**	Analysis of three independent simulations of 300 ns molecular dynamics simulations of 7-*O*-galloylpinocembrin (**36**).	S87
**Figure S113**	Analysis of three independent simulations of 300 ns molecular dynamics simulations of 4′-*O*-galloyl-DMC (**40**)	S88
**Figure S114**	Analysis of three independent simulations of 300 ns molecular dynamics simulations of 7-*O*-galloyltectochrysin (**42**)	S89
**Figure S115**	Analysis of three independent simulations of 300 ns molecular dynamics simulations of 5-*O*-galloylchrysin (**44**)	S90
**Figure S116**	Analysis of three independent simulations of 300 ns molecular dynamics simulations of gallic acid.	S91
**Figure S117**	Distance between thiolate nucleophile of Cys145 and reactive Michael acceptor carbon of galloyl group	S92
**Section VI.** Enzymatic assay		
**Figure S118**	Dose-dependent curves of galloylated flavonoids against SARS-CoV-2 3CL^{pro}	S94

Section I

Synthesis and Characterization of Inhibitors

^{1}H-NMR, ^{13}C-NMR, DEPT90, DEPT135, FTIR, and HRMS Data

1. Synthesis of acryloylated flavonoids

1.1 *General procedure A* for the synthesis of acryloylated flavonoids 12–21. (Example Given for 12)

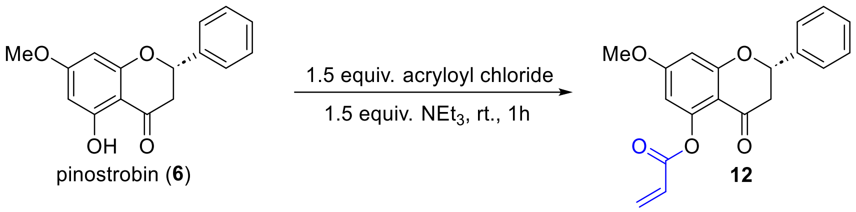

Pinostrobin (**6**) (200.0 mg, 0.7399 mmol) was dissolved in 5 mL of CH_2Cl_2. Then, acryloyl chloride (89 µL, 1.1098 mmol) was added to the solution, followed by NEt_3 (155 µL, 1.1098 mmol). The reaction was continuously stirred for 1 h at room temperature. Until the completion of reaction, the solution was neutralized by adding saturated $NaHCO_3$ solution, followed by extracting several times with CH_2Cl_2. The combined organic layer was dried over anhydrous Na_2SO_4. The crude product was purified by column chromatography on silica gel using isocratic elution of EtOAc:*n*hexane = 2:8 to provide 5-*O*-acryloylpinostrobin (**12**) in 92 %yield. Light-yellow solids; mp. 142.3–144.5 °C; R_f (30%EtOAc/hexane) 0.42; ν_{max} (ATR): 2975 (CH_3), 1744 (C=O), 1673 (C=O), 1622 (C=C), 1568 (C=C), 1276 (C-O), 1200 (C-O), 1151 (C-O), 1076 (C-O), 1062 (C-O); ^{1}H-NMR (500 MHz, $CDCl_3$): δ 2.73 (dd, J = 16.7, 2.8 Hz, 1H), 3.01 (dd, J = 16.7, 13.5 Hz, 1H), 5.46 (dd, J = 13.5, 2.8 Hz, 1H), 6.04 (d, J = 1.3 Hz, 1H), 6.06 (d, J = 1.3 Hz, 1H), 6.33 (d, J = 2.5 Hz, 1H), 6.41 (dd, J = 17.3, 10.5 Hz, 1H), 6.46 (d, J = 2.5 Hz, 1H), 6.61 (d, J = 1.3 Hz, 1H), 6.65 (d, J = 1.3 Hz, 1H), 7.36-7.47 (m, 5H); ^{13}C-NMR (125 MHz, $CDCl_3$): δ 45.2, 56.0, 79.7, 99.8, 104.9, 108.1, 126.3, 128.0, 129.0, 132.7, 138.5, 151.8, 164.3, 164.5, 165.6, 188.8; HRMS (ESI) calcd for $C_{19}H_{16}O_5$ $[M+H]^+$: *m*/*z* 325.1076 Found 325.1076. LC R_t: 3.727 min. Purity >99.9%.

1.2 Synthesis of acryloylated pinocembrins 13 and 14

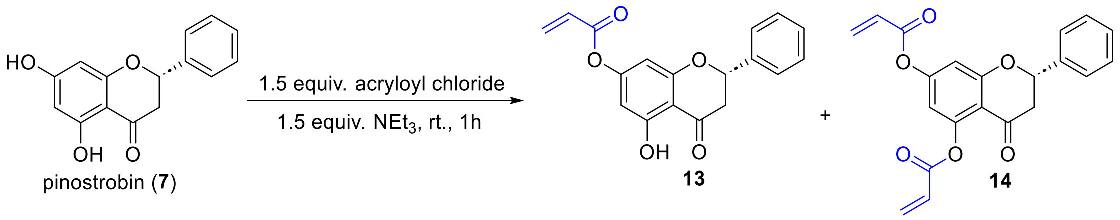

7-*O*-acryloylpinocembrin (**13**) and 5,7-diacryloylpinocembrin (**14**) was synthesized from pinocembrin (**7**) as the substrate following the general procedure A. Shortly, pinocembrin (**7**) (200.0 mg, 0.7804 mmol) was dissolved in CH_2Cl_2 then reacted with acryloyl chloride (95 µL, 1.1707 mmol) and NEt_3 (165 µL, 1.1707 mmol). Until the completion of reaction, the

product was purified by column chromatography using EtOAc:*n*hexane = 3:7 to yield compounds **13** and **14** in 36 and 42 %yield.

7-*O*-Acryloylpinocembrin (**13**): light-yellow viscous liquid; R_f (30%EtOAc/hexane) 0.58; ν_{max} (ATR): 3099 (C-H), 3073 (C-H), 3045 (C-H), 2976 (CH_2), 2917 (CH_2), 1751 (C=O), 1650 (C=O), 1630 (C=C), 1583 (C=C), 1502 (C=C), 1295 (C-O), 1267 (C-O), 1235 (C-O), 1186 (C-O), 1126 (C-O), 1090 (C-O), 1064 (C-O); ^{1}H-NMR (500 MHz, $CDCl_3$): δ 2.89 (dd, J = 17.2, 3.0 Hz, 1H), 3.14 (dd, J = 17.2, 13.2 Hz, 1H), 5.48 (dd, J = 13.2, 3.0 Hz, 1H), 6.05 (dd, J = 10.5, 1.1 Hz, 1H), 6.28 (dd, J = 17.3, 10.5 Hz, 1H), 6.36-6.39 (m, 2H), 6.61 (dd, J = 17.3, 1.1 Hz, 1H), 7.37-7.48 (m, 5H), 11.86 (s, 1H); ^{13}C-NMR (125 MHz, $CDCl_3$): δ 43.7, 79.5, 101.9, 103.4, 106.4, 126.3, 127.5, 129.1, 129.2, 133.7, 138.1, 158.5, 162.5, 163.5, 197.1; HRMS (ESI) calcd for $C_{18}H_{15}O_5$ $[M+H]^+$: *m*/*z* 311.0919 Found 311.0915. LC R_t: 3.740 min. Purity >99.9%.

5,7-*O*-Diacryloylpinocembrin (**14**): light-yellow viscous liquid; R_f (30%EtOAc/hexane) 0.48; ν_{max} (ATR): 2962 (CH_2), 2924 (CH_2), 2855 (CH), 1747 (C=O), 1688 (C=O), 1618 (C=C), 1577 (C=C), 1294 (C-O), 1266 (C-O), 1240 (C-O), 1130 (C-O), 1073 (C-O), 1057 (C-O); ^{1}H-NMR (500 MHz, $CDCl_3$): δ 2.79 (dd, J = 16.7, 2.8 Hz, 1H), 3.07 (dd, J = 16.7, 13.6 Hz, 1H), 5.52 (dd, J = 13.6, 2.8 Hz, 1H), 6.06 (dd, J = 2.5, 1.1 Hz, 1H), 6.29 (dd, J = 17.3, 10.5 Hz, 1H), 6.40 (dd, J = 17.3, 10.5 Hz, 1H), 6.62 (dd, J = 8.9, 1.1 Hz, 1H), 6.64 (d, J = 2.2 Hz, 1H), 6.65 (dd, J = 8.9, 1.1 Hz, 1H), 6.88 (dd, J = 2.2 Hz, 1H), 7.35-7.49 (m, 5H); ^{13}C-NMR (125 MHz, $CDCl_3$): δ 45.3, 79.8, 109.4, 110.7, 112.0, 126.3, 127.4, 127.8, 129.0, 129.1, 133.1, 134.0, 138.2, 151.2, 156.0, 163.3, 163.4, 164.3, 189.2; HRMS (ESI) calcd for $C_{21}H_{17}O_6$ $[M+H]^+$: *m*/*z* 365.1025 Found 365.1025. LC R_t: 3.620 min. Purity 99.8%.

1.3 Synthesis of acryloylated cardamonins 15 and 17

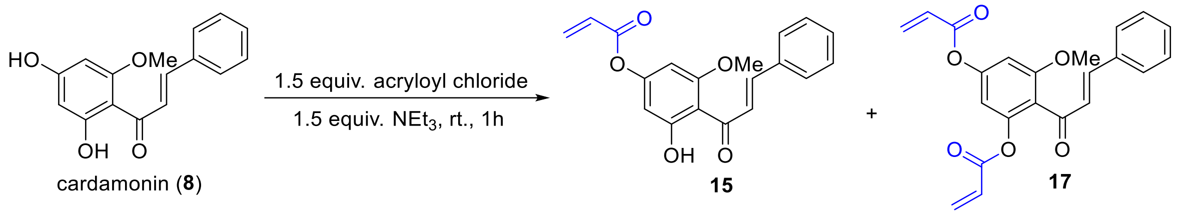

Following general procedure A, cardamonin (**8**) (200.0 mg, 0.7399 mmol) was dissolved in CH_2Cl_2 in the presence of reagents acryloyl chloride (89 µL, 1.1099 mmol) and NEt_3 (155 µL, 1.1099 mmol). The products were purified by column chromatography on silica gel using EtOAc:*n*hexane = 3:7 as eluents to provide 4′-*O*-acryloylcardamonin (**15**) and 2′,4′-diacryloylcardamonin (**16**) in 39 and 41 %yields.

4′-*O*-Acryloylcardamonin (**15**): yellow solids; mp. 148.9–150.4 °C; R_f (30%EtOAc/hexane) 0.48; ν_{max} (ATR): 2977 (CH_3), 1736 (C=O), 1631 (C=O), 1591 (C=C), 1563 (C=C), 1201 (C-O), 1187 (C-O), 1149 (C-O), 1136 (C-O), 1113 (C-O), 1073 (C-O), 1030 (C-O); ^{1}H-NMR (500 MHz, $CDCl_3$): δ 3.95 (s, 3H), 6.06 (dd, J = 10.5, 1.1 Hz, 1H), 6.28 (d, J = 2.2 Hz, 1H), 6.31 (dd, J = 17.3, 10.5 Hz, 1H), 6.45 (d, J = 2.2 Hz, 1H), 6.63 (dd, J = 17.3, 1.1 Hz, 1H), 7.38-7.46 (m, 3H), 7.59-7.64 (m, 2H), 7.82 (d, J = 15.6 Hz, 1H), 7.86 (d, J = 15.6 Hz, 1H), 13.54 (s, 1H); ^{13}C-NMR (125 MHz, $CDCl_3$): δ 56.3, 96.4, 104.1, 110.0, 127.3, 127.7, 128.7, 129.1, 130.5, 133.6, 135.4, 143.5, 156.7, 162.2, 163.7, 166.5, 193.8; HRMS (ESI) calcd for $C_{19}H_{17}O_5$ $[M+H]^+$: m/z 325.1076 Found 325.1075. LC R_t: 3.995 min. Purity 95.0%.

2′,4′-*O*-Diacryloylcardamonin (**17**): yellow-viscous liquid; R_f (30%EtOAc/hexane) 0.32; ν_{max} (ATR): 1747 (C=O), 1655 (C=O), 1608 (C=C), 1579 (C=C), 1237 (C-O), 1212 (C-O), 1131 (C-O), 1091 (C-O), 1067 (C-O), 1030 (C-O); ^{1}H-NMR (500 MHz, $CDCl_3$): δ 3.82 (s, 3H), 5.92 (dd, J = 10.5, 1.1 Hz, 1H), 6.06 (dd, J = 10.5, 1.1 Hz, 1H), 6.15 (dd, J = 17.3, 10.5 Hz, 1H), 6.32 (dd, J = 17.3, 10.5 Hz, 1H), 6.47 (dd, J = 17.3, 1.1 Hz, 1H), 6.64 (dd, J = 17.3, 1.1 Hz, 1H), 6.74 (d, J = 1.9 Hz, 1H), 6.76 (d, J = 1.9 Hz, 1H), 6.94 (d, J = 16.2 Hz, 1H), 7.36-7.39 (m, 3H), 7.41 (d, J = 16.2 Hz, 1H), 7.50-7.56 (m, 2H, 1H); ^{13}C-NMR (125 MHz, $CDCl_3$): δ 56.4, 103.2, 109.1, 120.5, 127.2, 127.6, 127.8, 128.7, 129.0, 130.8, 133.5, 133.6, 134.6, 146.1, 148.8, 152.4, 158.4, 163.8, 163.9, 192.0; HRMS (ESI) calcd for $C_{22}H_{18}O_6$ $[M+H]^+$: m/z 379.1182 Found 379.1181. LC R_t: 4.594 min. Purity 97.5%.

1.4 Synthesis of acryloylated DMCs 16 and 18

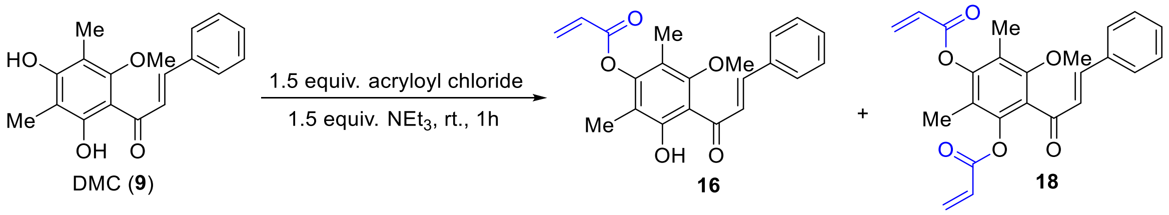

Following general procedure A, DMC (**9**) (200.0 mg, 0.6704 mmol) was dissolved in CH_2Cl_2 followed by adding acryloyl chloride (81 µL, 1.0056 mmol) and NEt_3 (140 µL, 1.0056 mmol). Until the completion of reaction, the product was purified through column chromatography using EtOAc:*n*hexane = 3:7 to yield 4′-*O*-acryloyl-DMC (**17**) and 2′,4′-diacryloyl-DMC (**18**) in 47 and 29 %yields.

4′-*O*-Acryloyl-DMC (**16**): orange solids; mp. 87.7–91.1 °C; R_f (5%EtOAc/hexane) 0.60; ν_{max} (ATR): 3029 (C-H), 2969 (CH_3), 2933 (CH_3), 1739 (C=O), 1633 (C=O), 1604 (C=C), 1561 (C=C), 1287 (C-O), 1235 (C-O), 1134 (C-O), 1110 (C-O), 1087 (C-O); ^{1}H-NMR (500 MHz, $CDCl_3$): δ 2.04 (s, 3H), 2.06 (s, 3H), 3.68 (s, 3H), 6.10 (dd, J = 10.5, 1.1 Hz, 1H),

6.38 (dd, J = 17.3, 10.5 Hz), 6.68 (dd, J = 17.3, 1.1 Hz, 1H), 7.37-7.47 (m, 3H), 7.64-7.68 (m, 2H), 7.89 (d, J = 15.7 Hz, 1H), 7.96 (d, J = 15.7 Hz, 1H), 12.95 (s, 1H); ^{13}C-NMR (125 MHz, $CDCl_3$): δ 9.1, 9.3, 62.8, 113.5, 115.4, 115.9, 126.5, 127.2, 128.7, 129.1, 130.7, 133.6, 135.2, 144.0, 154.0, 158.3, 160.9, 163.2, 194.5; HRMS (ESI) calcd for $C_{21}H_{21}O_5$ $[M+H]^+$: *m*/*z* 353.1389 Found 353.1388. LC R_t: 3.560 min. Purity >99.9%.

2′,4′-*O*-Diacryloyl-DMC (**18**): white solids; mp. 113.2–115.8 °C; R_f (30%EtOAc/hexane) 0.45; ν_{max} (ATR): 3067 (C-H), 2973 (CH_3), 2932 (CH_3), 1741 (C=O), 1673 (C=O), 1603 (C=C), 1577 (C=C), 1236 (C-O), 1143 (C-O), 1119 (C-O), 1101 (C-O), 1077 (C-O), 1065 (C-O), 1038 (C-O); ^{1}H-NMR (500 MHz, $CDCl_3$): δ 1.94 (s, 3H), 2.12 (s, 3H), 3.71 (s, 3H), 5.93 (dd, J = 10.5, 1.1 Hz, 1H), 6.10 (dd, J = 10.5, 1.1 Hz, 1H), 6.19 (dd, J = 17.3, 10.5 Hz, 1H), 6.39 (dd, J = 17.3, 10.5 Hz, 1H), 6.48 (dd, J = 17.3, 1.1 Hz, 1H), 6.69 (dd, J = 17.3, 1.1 Hz, 1H), 6.99 (d, J = 16.1 Hz, 1H), 7.35-7.41 (m, 3H), 7.45 (d, J = 16.2 Hz, 1H), 7.51-7.57 (m, 2H); ^{13}C-NMR (125 MHz, $CDCl_3$): δ 9.9, 10.3, 62.7, 121.0, 123.2, 126.0, 127.0, 127.2, 127.4, 128.8, 129.0, 130.8, 133.5, 133.6, 134.6, 144.7, 146.6, 149.9, 154.7, 163.3, 163.6, 192.7; HRMS (ESI) calcd for $C_{24}H_{23}O_6$ $[M+H]^+$: *m*/*z* 407.1495 Found 407.1497. LC R_t: 3.808 min. Purity >99.9%.

1.5 Synthesis of acryloylated tectochrysin 19

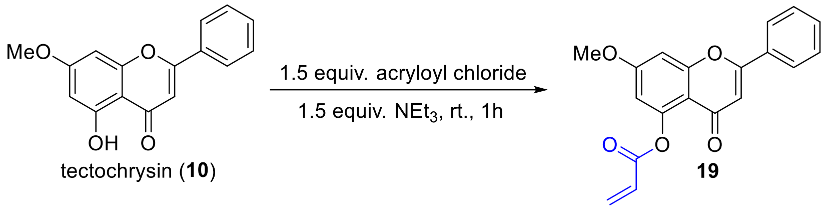

Following general procedure A, tectochrysin (**10**) (200.0 mg, 0.7455 mmol) was dissolved in CH_2Cl_2. Then, the reagents acryloyl chloride (90 µL, 1.1183 mmol) and NEt_3 (155 µL, 1.1183 mmol) was added to the solution. The product was purified by column chromatography on silica gel using CH_2Cl_2 as an eluent to yield 7-*O*-acryloyltectochrysin (**19**) in 86 %yield. Light-yellow solids; mp. 149.0–150.5 °C; R_f (30%EtOAc/hexane) 0.25; ν_{max} (ATR): 1745 (C=O), 1649 (C=O), 1631 (C=C), 1617 (C=C), 1244 (C-O), 1138 (C-O), 1101 (C-O), 1087 (C-O), 1076 (C-O), 1031 (C-O); ^{1}H-NMR (500 MHz, $CDCl_3$): δ 3.92 (s, 3H), 6.07 (dd, J = 10.5, 1.3 Hz, 1H), 6.46 (dd, J = 17.3, 10.5 Hz, 1H), 6.66 (d, J = 2.5 Hz, 1H), 6.67 (dd, J = 17.3, 1.3 Hz, 1H), 6.90 (d, J = 2.5 Hz, 1H), 7.46-7.56 (m, 3H), 7.84-7.87 (m, 2H); ^{13}C-NMR (125 MHz, $CDCl_3$): δ 56.1, 99.3, 108.5, 108.6, 111.3, 126.2, 128.0, 129.1, 131.5, 131.6, 132.8, 150.5, 159.0, 162.1, 163.6, 164.7, 176.5; HRMS (ESI) calcd for $C_{19}H_{15}O_5$ $[M+H]^+$: *m*/*z* 323.0919 Found 323.0919. LC R_t: 3.716 min. Purity 95.0%.

1.6 Synthesis of acryloylated chrysins 20 and 21

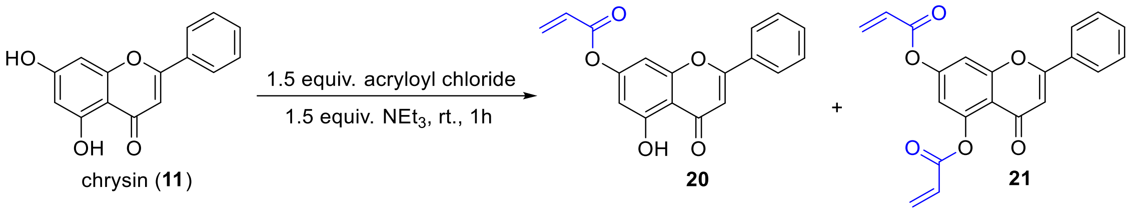

Following general procedure A, chrysin (**11**) (200.0 mg, 0.7866 mmol) was dissolved in CH_2Cl_2, followed by adding acryloyl chloride (95 μL, 1.1780 mmol) and NEt_3 (164 μL, 1.1780 mmol). Until the completion of reaction, the products were purified through column chromatography on silica gel using CH_2Cl_2 as eluent to yield 7-*O*-acryloylchrysin (**20**) and 5,7-diacryloylchrysin (**21**) in 42 and 20 %yields.

7-*O*-Acryloylchrysin (**20**): light-yellow solids; mp. 145.6–147.9 °C; R_f (30%EtOAc/hexane) 0.60; ν_{max} (ATR): 1748 (C=O), 1666 (C=O), 1620 (C=C), 1597 (C=C), 1573 (C=C), 1564 (C=C), 1554 (C=C), 1272 (C-O), 1257 (C-O), 1238 (C-O), 1194 (C-O), 1150 (C-O), 1133 (C-O), 1111 (C-O), 1099 (C-O), 1067 (C-O); ^{1}H-NMR (500 MHz, $CDCl_3$): δ 6.09 (dd, J = 10.5,1.0 Hz, 1H), 6.34 (dd, J = 17.3, 10.5 Hz, 1H), 6.63 (d, J = 2.1 Hz, 1H), 6.66 (dd, J = 17.3, 1.0 Hz, 1H), 6.92 (d, J = 2.1 Hz, 1H), 7.49-7.61 (m, 3H), 7.87-7.92 (m, 2H), 12.74 (s, 1H); ^{13}C-NMR (125 MHz, $CDCl_3$): δ 101.1, 105.6, 106.3, 109.1, 126.6, 127.5, 129.3, 131.1, 132.3, 133.9, 156.0, 156.9, 162.1, 163.6, 164.9, 183.0; HRMS (ESI) calcd for $C_{18}H_{13}O_5$ $[M+H]^+$: *m/z* 309.0763 Found 309.0763. LC R_t: 4.468 min. Purity 99.5%.

5,7-*O*-Diacryloylchrysin (**21**): clear needle-shaped crystals; R_f (30%EtOAc/hexane) 0.38; mp. 148.2–149.5 °C; ν_{max} (ATR): 1744 (C=O), 1649 (C=O), 1630 (C=C), 1617 (C=C), 1580 (C=C), 1293 (C-O), 1242 (C-O), 1136 (C-O), 1102 (C-O), 1085 (C-O), 1075 (C-O); ^{1}H-NMR (500 MHz, $CDCl_3$): δ 6.08-6.10 (*m*, 2H), 6.34 (*dd*, J = 17.3, 10.5 Hz, 1H), 6.46 (*dd*, J = 17.3, 10.5 Hz, 1H), 6.65 (*s*, 1H), 6.67 (*dd*, J = 17.3, 3.2 Hz, 2H), 6.95 (*dd*, J = 2.2 Hz, 1H), 7.44 (*d*, J = 2.2 Hz, 1H), 7.48-7.62 (m, 3H), 7.84-7.88 (m, 2H); ^{13}C-NMR (125 MHz, $CDCl_3$): δ 108.8, 109.3, 113.8, 115.2, 126.4, 127.3, 127.8, 129.2, 131.2, 131.9, 133.1, 134.2, 150.2, 154.0, 157.9, 162.7, 163.3, 164.4, 176.4; HRMS (ESI) calcd for $C_{21}H_{15}O_6$ $[M+H]^+$: *m/z* 363.0869 Found 363.0869. LC R_t: 4.204 min. Purity >99.9%.

2. Synthesis of nitropinostrobins 22–24.

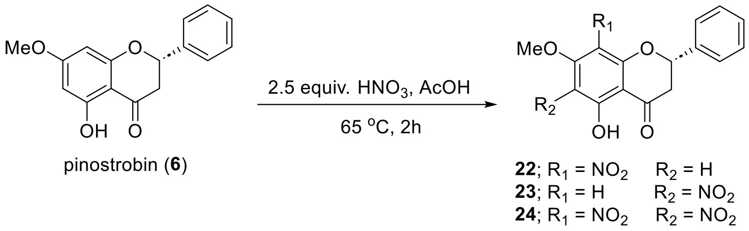

The introduction of nitro group to pinostrobin (**6**) was conducted through nitration. Pinostrobin (**6**) (400.0 mg, 1.4799 mmol) was dissolved in 10 mL of glacial CH_3COOH. Then, 154.2 μL of HNO_3 was subsequently added to the solution. The mixture was then heated to 65 °C for 2 h. Until the completion of reaction, the reaction was quenched by basified with saturated $NaHCO_3$ to pH 4-5. The reaction was then extracted several times with CH_2Cl_2. The combined organic layer was dried over anhydrous Na_2SO_4. The products were purified through column chromatography on silica gel using isocratic elution of EtOAc:*n*hexane = 2:98 to yield 8-nitropinostrobin (**22**), 6-nitropinostrobin (**23**) and 6,8-dinitropinostrobin (**24**) in 34, 21, and 12% yields.

8-Nitropinostrobin (**22**): white solids; ν_{max} (ATR): 1662 (C=O), 1634 (C=C), 1613 (C=C), 1576 (C=C), 1530 (N-O), 1503 (C=C), 1360 (C-O), 1347 (C-O), 1289 (C-O), 1205 (C-O), 1178 (C-O), 1125 (C-O), 1061 (C-O); ^{1}H-NMR (500 MHz, $CDCl_3$): δ 3.00 (dd, J = 17.4, 3.4 Hz, 1H), 3.16 (dd, J = 17.4, 12.0 Hz, 1H), 3.93 (s, 3H), 5.61 (dd, J = 12.0, 3.4 Hz, 1H), 6.15 (s, 1H), 7.34-7.50 (m, 5H), 12.29 (s, 1H); ^{13}C-NMR (125 MHz, $CDCl_3$): δ 42.8, 57.1, 80.2, 93.3, 102.1, 126.0, 129.1, 129.3, 137.0, 154.4, 159.4, 165.1, 195.5; HRMS (ESI) calcd for $C_{16}H_{14}NO_6$ $[M+H]^+$: *m/z* 316.0821 Found 316.0808. LC R_t: 4.038 min. Purity >99.9%.

6-Nitropinostrobin (**23**): white solids; ν_{max} (ATR): 1657 (C=O), 1632 (C=C), 1574 (C=C), 1533 (N-O), 1356 (C-O), 1341 (C-O), 1298 (C-O), 1265 (C-O), 1145 (C-O), 1103 (C-O); ^{1}H-NMR (500 MHz, $CDCl_3$): δ 2.92 (dd, J = 17.4, 3.2 Hz, 1H), 3.16 (dd, J = 17.4, 13.0 Hz, 1H), 3.92 (s, 3H), 5.51 (dd, J = 13.0, 3.2 Hz, 1H), 6.15 (s, 1H), 7.35-7.55 (m, 5H), 12.55 (s, 1H); ^{13}C-NMR (125 MHz, $CDCl_3$): δ 43.0, 57.1, 80.1, 91.8, 102.3, 126.3, 129.2, 129.5, 137.3, 156.0, 159.5, 163.9, 196.2; HRMS (ESI) calcd for $C_{16}H_{14}NO_6$ $[M+H]^+$: *m/z* 316.0821 Found 316.0813. LC R_t: 4.090 min. Purity >99.9%.

6,8-Dinitropinostrobin (**24**): white solids; ν_{max} (ATR): 1660 (C=O), 1630 (C=C), 1577 (C=C), 1329 (C-O), 1299 (C-O), 1262 (C-O), 1141 (C-O), 1100 (C-O); ^{1}H-NMR (500 MHz, $CDCl_3$): δ 3.12 (dd, J = 17.6, 3.5 Hz, 1H), 3.27 (dd, J = 17.6, 11.9 Hz, 1H), 4.05 (s, 3H), 5.70 (dd, J = 11.9, 3.5 Hz, 1H), 7.33-7.53 (m, 5H), 12.74 (s, 1H); ^{13}C-NMR (125 MHz, $CDCl_3$): δ

42.5, 63.0, 81.1, 103.3, 126.1, 129.4, 129.8, 135.7, 152.8, 155.2, 156.8, 195.8; HRMS (ESI) calcd for $C_{16}H_{13}N_2O_8$ $[M+H]^+$: *m*/*z* 361.0672 Found 361.0675. LC R_t: 4.021 min. Purity > 99.9%. LC R_t: 4.021 min. Purity >99.9%.

3. *General procedure B* for the synthesis of aminopinostrobins 25 and 26. (Example Given for 25).

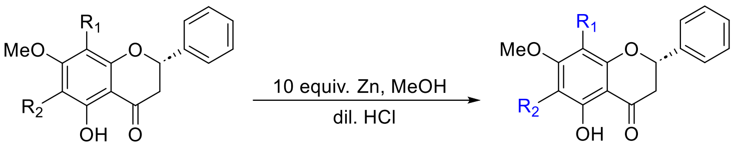

8-Nitropinostrobin (**22**) (100.0 mg, 0.3172 mmol) was dissolved in 5 mL of MeOH, followed by adding Zn dust (207.3 mg, 3.1722 mmol) to the solution. Then, the solution was slowly cooled down in ice-bath followed by adding 3 drops of 1 M HCl solution. The reaction was continuously stirred for 10 min. Until the completion of reaction, the reaction was quenched by adding saturated $NaHCO_3$ until reached to pH 8. The solution was extracted several times with EtOAc. The product was purified by column chromatography on silica gel using EtOAc:*n*hexane = 6:94 as an eluent to yield 8-aminopinostrobin (**25**) in 76 %yield. Light-yellow solids; ν_{max} (ATR): 3450 (N-H), 3364 (N-H), 1631 (C=O), 1602 (C=C), 1584 (C=C), 1318 (C-O), 1284 (C-O), 1229 (C-O), 1202 (C-O), 1185 (C-O), 1158 (C-O), 1092 (C-O), 1073 (C-O); ^{1}H-NMR (500 MHz, $CDCl_3$): δ 2.84 (dd, J = 17.1, 3.0 Hz, 1H), 3.10 (dd, J = 17.1, 12.8 Hz, 1H), 3.90 (s, 3H), 5.44 (dd, J = 12.8, 3.0 Hz, 1H), 6.14 (s, 1H), 7.34-7.56 (m, 5H), 11.60 (s, 1H); ^{13}C-NMR (125 MHz, $CDCl_3$): δ 43.9, 56.0, 79.3, 92.4, 102.8, 116.5, 126.2, 128.8, 138.7, 146.8, 156.0, 156.3, 196.0. HRMS (ESI) calcd for $C_{16}H_{16}NO_4$ $[M+H]^+$: *m*/*z* 286.1079 Found 286.1077. LC R_t: 4.092 min. Purity >99.9%.

6-Aminopinostrobin (**26**) was prepared following the *general procedure B*. Shortly, 6-nitropinostrobin (**23**) was reacted with Zn dust in the presence of HCl solution. Until the completion of reaction, 6-aminopinostrobin (**26**) was obtained in 72 %yield. Light-yellow solids; ν_{max} (ATR): 3465 (N-H), 3376 (N-H), 1654 (C=O), 1626 (C=C), 1601 (C=C), 1578 (C=C), 1513 (C=C), 1303 (C-O), 1291 (C-O), 1200 (C-O), 1174 (C-O), 1160 (C-O), 1084 (C-O), 1055 (C-O); ^{1}H-NMR (500 MHz, $CDCl_3$): δ 2.80 (dd, J = 17.2, 3.0 Hz, 1H), 3.09 (dd, J = 17.2, 13.2 Hz, 1H), 3.53 (br s, 2H), 3.88 (s, 3H), 5.39 (dd, J = 13.2, 3.0 Hz, 1H), 6.14 (s, 1H), 7.31-7.60 (m, 5H), 11.68 (s, 1H); ^{13}C-NMR (125 MHz, $CDCl_3$): δ 43.9, 56.0, 79.3, 92.4, 102.8, 116.5, 126.2, 128.8, 128.8, 138.7, 146.8, 156.0, 156.3, 196.0. HRMS (ESI) calcd for $C_{16}H_{16}NO_4$ $[M+H]^+$: *m*/*z* 286.1079 Found 286.1115. LC R_t: 4.214 min. Purity 97.8%.

4. Synthesis of protected hydroxybenzoic acid derivatives

4.1 *General procedure C* for the synthesis of acetylsalicylic acid

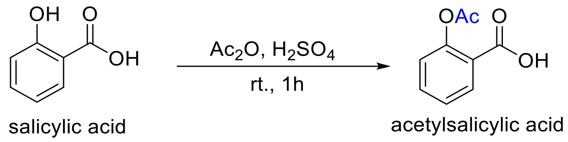

Salicylic acid (3.0 g, 21.7199 mmol) was dissolved in 5 mL of acetic anhydride with strong stirring. Then, 2 drops of conc. sulfuric acid were dropped to reaction. The reaction was continuously stirred for 1 hour at room temperature. Until the completion of reaction monitored by TLC, the acetic anhydride residue was eliminated by adding 20 mL of deionized water. The white precipitate was obtained, which was collected by filtration and washed several times with cold water. The product was purified by precipitation with dichloromethane/*n*-hexane to yield acetylsalicylic acid in 87 %yield.

Acetylsalicylic acid. White solids; ν_{max} (ATR): 2972 (CH_3), 1750 (C=O), 1679 (C=O), 1603 (C=C), 1216 (C-O), 1182 (C-O), 1134 (C-O), 1092 (C-O), 1038 (C-O), 1011 (C-O); ^{1}H-NMR (500 MHz, $CDCl_3$): 2.35 (s, 3H), 7.14 (d, *J* = 8.0 Hz), 7.36 (td, *J* = 8.0, 0.8 Hz), 7.63 (td, *J* = 8.0, 1.5 Hz), 8.12 (dd, *J* = 8.0, 1.5 Hz); ^{13}C-NMR (125 MHz, $CDCl_3$): 21.2, 122.3, 124.2, 126.3, 132.7, 135.0, 151.4, 169.9, 170.0; HRMS (ESI) calcd for $C_9H_9O_4$ $[M+H]^+$: m/z 181.0501 Found 181.0506.

4.2 Synthesis of acetylvanilic acid

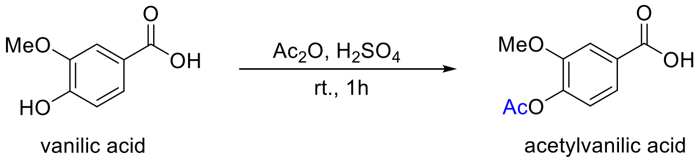

Acetylvanilic acid was synthesized following the general procedure C for the synthesis of acetylsalicylic acid. Shortly, vanillic acid (3.0 g, 17.8414 mmol) was reacted with acetic anhydride in the presence of H_2SO_4 to provide acetylvanilic acid in 71 %yield. White solids; ν_{max} (ATR): 2972 (CH_3), 1758 (C=O), 1683 (C=O), 1595 (C=C), 1506 (C=C), 1297 (C-O), 1263 (C-O), 1168 (C-O), 1111 (C-O), 1028 (C-O); ^{1}H-NMR (500 MHz, $CDCl_3$): 2.35 (s, 3H), 3.91 (s, 3H), 7.14 (d, *J* = 8.2 Hz, 1H), 7.71 (d, *J* = 1.8 Hz, 1H), 7.76 (dd, *J* = 8.2, 1.8 Hz, 1H); ^{13}C-NMR (125 MHz, $CDCl_3$): 20.8, 56.2, 114.0, 123.1, 123.6, 128.0, 144.5, 151.3, 168.6, 171.3; HRMS (ESI) calcd for $C_{10}H_{11}O_5$ $[M+H]^+$: *m/z* 211.0606 Found 211.0610.

4.3 Synthesis of triacetylgallic acid

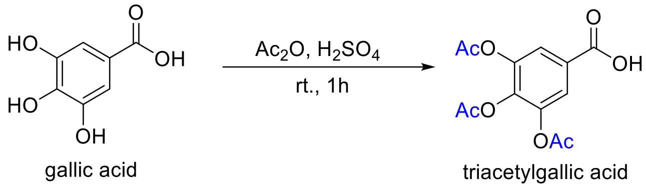

3,4,5-*O*-triacetylgallic acid was synthesized following the general procedure C. In brief, gallic acid (3.0 g, 17.6346 mmol) was reacted with acetic anhydride with H_2SO_4 as catalyst. After precipitation, acetylated gallic acid was obtained in 89 %yield. White solids; ν_{max} (ATR): 2987 (CH_3), 1786 (C=O), 1767 (C=O), 1691 (C=O), 1261 (C-O), 1161 (C-O), 1093 (C-O), 1048 (C-O); ^{1}H-NMR (500 MHz, $CDCl_3$): 2.31 (s, 6H), 2.32 (s, 3H), 7.86 (s, 2H); ^{13}C-NMR (125 MHz, $CDCl_3$): 20.3, 20.7, 123.0, 127.5, 139.5, 143.7, 166.5, 167.7, 169.6; HRMS (ESI) calcd for $C_{13}H_{13}O_8$ $[M+H]^+$: *m*/*z* 297.0610 Found 297.0607.

5. Synthesis of benzoylated flavonoids

5.1 *General procedure D* for the synthesis of 5-*O*-benzoylpinostrobin (27)

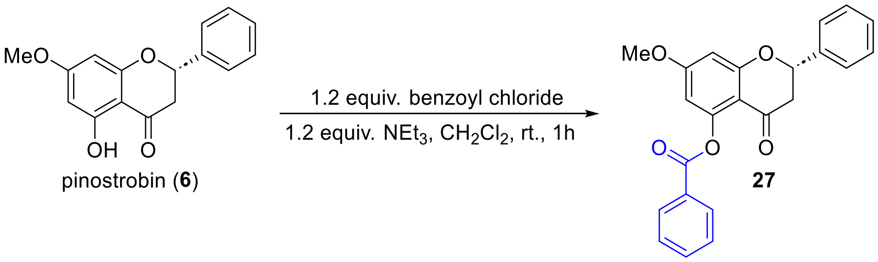

Pinostrobin (**6**) (200.0 mg, 0.7399 mmol) was dissolved in CH_2Cl_2. Then, benzoyl chloride (103 μL, 0.8879 mmol) and NEt_3 (123 μL, 0.8879 mmol) was slowly added to the solution. The reaction was stirred for 1 h at room temperature. Until the completion of reaction, the reaction was neutralized by adding saturated $NaHCO_3$. The product was obtained after purification through silica gel column chromatography using isocratic elution of EtOAc:*n*hexane = 1:9 to yield 5-*O*-benzoylpinostrobin (**27**) in 92 %yield. White solids; mp. 113.2–115.8 °C; R_f (30%EtOAc/hexane) 0.45; ν_{max} (ATR): 1742 (C=O), 1683 (C=O), 1615 (C=C), 1568 (C=C), 1261 (C-O), 1250 (C-O), 1198 (C-O), 1150 (C-O), 1090 (C-O), 1067 (C-O); ^{1}H-NMR (500 MHz, $CDCl_3$): δ 2.71 (dd, J = 16.7, 2.8 Hz, 1H), 3.01 (dd, J = 16.7, 13.5 Hz, 1H), 3.84 (s, 3H), 5.48 (dd, J = 13.5, 2.8 Hz, 1H), 6.43 (d, J = 2.5 Hz, 1H), 6.49 (d, J = 2.5 Hz, 1H), 7.35-7.54 (m, 7H), 7.57-7.67 (m, 1H), 8.20-8.25 (m, 2H); ^{13}C-NMR (125 MHz, $CDCl_3$): δ 45.1, 56.0, 79.8, 99.8, 105.1, 108.3, 126.3, 128.6, 129.0, 130.4, 133.5, 138.6, 152.2, 164.4, 165.3, 165.6, 188.8; HRMS (ESI) calcd for $C_{23}H_{19}O_5$ $[M+H]^+$: *m*/*z* 375.1232 Found 375.1221. LC R_t: 4.483 min. Purity 99.2%.

5.2 *General procedure E* for the synthesis of 5-*O*-(acetylsalicyloyl)pinostrobin (28)

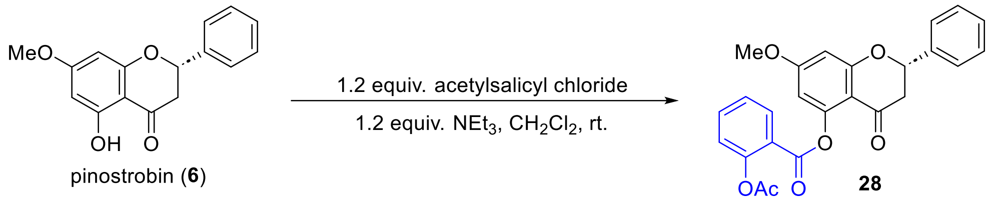

Acetylsalicylic acid (159.9 mg, 0.8879 mmol) was dissolved in 5 mL of CH_2Cl_2. Then, $SOCl_2$ (644 µL, 8.8788 mmol) was added to the reaction. The reaction was refluxed for 1 h, followed by solvent removal by reduced pressure to yield crude acetylsalicyloyl chloride, which was redissolved in 5 mL of CH_2Cl_2. Then, pinostrobin (**6**) (200.0 mg, 0.7399 mmol) dissolved in 5 mL of CH_2Cl_2 was added to the reaction, followed by adding NEt_3 (123 µL, 0.8879 mmol). The reaction was stirred for 1 h at room temperature. Until the completion of reaction, the solution was neutralized by adding saturated $NaHCO_3$, followed by extracting several times with CH_2Cl_2. The combined organic layer was dried over anhydrous Na_2SO_4. The crude product was purified through column chromatography on silica gel using EtOAc:*n*hexane = 8:2 to yield compound **28** in 84 %yield. White solids; ν_{max} (ATR): 1766 (C=O), 1740 (C=O), 1687 (C=O), 1615 (C=C), 1568 (C=C), 1509 (C=C), 1370 (C-O), 1271 (C-O), 1249 (C-O), 1220 (C-O), 1195 (C-O), 1161 (C-O), 1127 (C-O), 1085 (C-O), 1050 (C-O), 1032 (C-O); ^{1}H-NMR (500 MHz, $CDCl_3$): 2.31 (s, 6H), 2.31 (s, 3H), 2.73 (dd, J = 16.7, 2.8 Hz, 1H), 3.02 (dd, J = 16.7, 13.5 Hz, 1H), 3.85 (s, 3H), 5.48 (dd, J = 13.5, 2.8 Hz, 1H), 6.40 (d, J = 2.5 Hz, 1H), 6.48 (d, J = 2.5 Hz, 1H), 7.18 (dd, J = 8.1, 1.0 Hz, 1H), 7.36-7.48 (m, 6H), 7.63 (ddd, J = 8.1, 7.6, 1.7 Hz, 1H), 8.33 (dd, J = 7.9, 1.7 Hz, 1H); ^{13}C-NMR (125 MHz, $CDCl_3$): 21.2, 45.1, 56.0, 79.8, 100.0, 105.2, 108.1, 122.9, 123.9, 126.3, 129.0, 132.8, 134.5, 138.6, 151.3, 151.8, 162.8, 164.4, 165.6, 169.8, 188.7; HRMS (ESI) calcd for $C_{25}H_{21}O_7$ $[M+H]^+$: m/z 433.1287 Found 433.1272. LC R_t: 4.382 min. Purity 99.2%.

5.3 Synthesis of 7-*O*-benzoylpinocembrin (32)

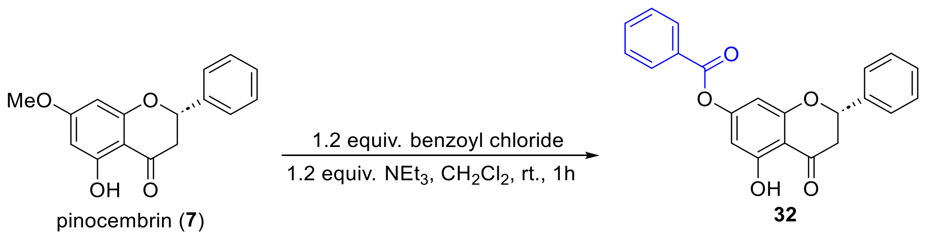

Following general procedure D, pinocembrin (**7**) (200.0 mg, 0.7805 mmol) was dissolved in CH_2Cl_2 in the presence of benzoyl chloride (108 µL, 0.9366 mmol) and NEt_3 (130 µL, 0.9366 mmol). The product was purified by column chromatography on silica gel to yield

7-*O*-benzoylpinocembrin (**32**) in 93 %yield. White solids; ν_{max} (ATR): 1726 (C=O), 1645 (C=O), 1634 (C=C), 1579 (C=C), 1337 (C-O), 1292 (C-O), 1245 (C-O), 1180 (C-O), 1133 (C-O), 1089 (C-O), 1053 (C-O); ^{1}H-NMR (500 MHz, $CDCl_3$): δ 2.90 (dd, J = 17.2, 3.0 Hz, 1H), 3.15 (dd, J = 17.2, 13.2 Hz, 1H), 5.49 (dd, J = 13.2, 3.0 Hz, 1H), 6.46 (m, 2H), 7.36-7.56 (m, 7H), 7.62-7.69 (m, 1H), 8.13-8.21 (m, 2H), 11.89 (s, 1H); ^{13}C-NMR (125 MHz, $CDCl_3$): δ 43.7, 79.4, 102.1, 103.6, 106.4, 126.2, 128.8, 129.0, 129.1, 129.2, 130.4, 134.1, 138.1, 158.9, 162.5, 163.5, 164.2, 197.2; HRMS (ESI) calcd for $C_{22}H_{17}O_5$ $[M+H]^+$: *m*/*z* 361.1076 Found 361.1069. LC R_t: 4.827 min. Purity >99.9%.

5.4 Synthesis of 5-*O*-(*O*-acetylvanilloyl)pinostrobin (29)

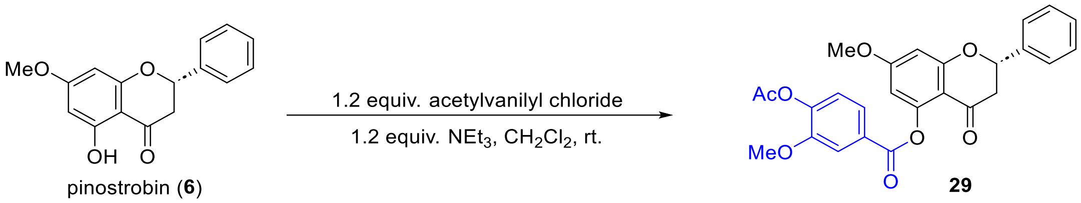

Compound **29** was prepared following the general procedure E, *O*-acetylvanillic acid (186.6 mg, 0.8879 mmol) was reacted with $SOCl_2$ (644 µL, 8.8788 mmol). Then, the *O*-acetylvanilloyl chloride was reacted with pinostrobin (**6**) (200.0 mg, 0.7399 mmol), NEt_3 (123 µL, 0.8879 mmol). Until the completion of reaction, the product was purified through column chromatography using EtOAc:*n*hexane = 2:8 to yield compound **29** in 89 %yield. White solids; ν_{max} (ATR): 1769 (C=O), 1745 (C=O), 1687 (C=O), 1617 (C=C), 1573 (C=C), 1509 (C=C), 1372 (C-O), 1286 (C-O), 1267 (C-O), 1238 (C-O), 1195 (C-O), 1167 (C-O), 1151 (C-O), 1124 (C-O), 1087 (C-O), 1063 (C-O), 1033 (C-O); ^{1}H-NMR (500 MHz, $CDCl_3$): 2.35 (s, 3H), 2.72 (dd, J = 16.8, 2.8 Hz, 1H), 3.02 (dd, J = 16.8, 13.5 Hz, 1H), 3.86 (s, 3H), 3.92 (s, 3H), 5.49 (dd, J = 13.5, 2.8 Hz, 1H), 6.42 (d, J = 2.5 Hz, 1H), 6.50 (d, J = 2.5 Hz, 1H), 7.18 (d, J = 1.9 Hz, 1H), 7.35-7.49 (m, 5H), 7.81 (d, J = 1.9 Hz, 1H), 7.86 (dd, J = 8.2, 1.9 Hz, 1H); ^{13}C-NMR (125 MHz, $CDCl_3$): 20.8, 45.2, 56.0, 56.3, 79.8, 99.9, 105.1, 108.3, 114.2, 123.1, 123.6, 126.3, 128.5, 129.0, 138.6, 144.2, 151.3, 152.1, 164.4, 164.6, 165.6, 168.6, 188.8; HRMS (ESI) calcd for $C_{26}H_{23}O_8$ $[M+H]^+$: *m*/*z* 463.1393 Found 463.1376. LC R_t: 4.355 min. Purity 99.8%.

5.5 Synthesis of 5-*O*-(tri-*O*-acetylgalloyl)pinostrobin (30)

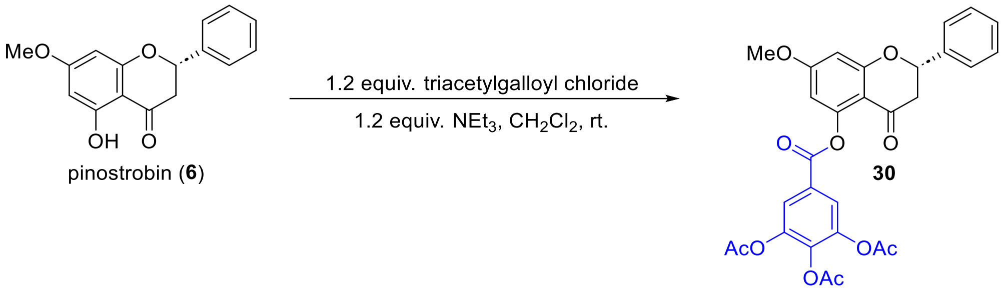

Following general procedure E, *O*-triacetylgallic acid (263.3 mg, 0.8879 mmol) was refluxed with $SOCl_2$ (644 µL, 8.8788 mmol). The crude *O*-triacetylgalloyl chloride was redissolved with 5 mL of CH_2Cl_2. Then, the solution of pinostrobin (**6**) (200.0 mg, 0.7399 mmol) dissolved in 5 mL of CH_2Cl_2 was added to the reaction, followed by NEt_3 (123 µL, 0.8879 mmol). Until the completion of reaction, the product was purified through column chromatography using EtOAc:*n*hexane = 3:7 as eluent to yield compound **30** in 75 %yield. white solids; ν_{max} (ATR): 1775 (C=O), 1746 (C=O), 1685 (C=O), 1618 (C=C), 1570 (C=C), 1501 (C=C), 1327 (C-O), 1268 (C-O), 1239 (C-O), 1191 (C-O), 1151 (C-O), 1090 (C-O), 1048 (C-O), 1034 (C-O), 1015 (C-O); ^{1}H-NMR (500 MHz, $CDCl_3$): 2.31 (s, 6H), 2.32 (s, 3H), 2.72 (dd, J = 16.8, 2.8 Hz, 1H), 3.01 (dd, J = 16.8, 13.5 Hz, 1H), 3.85 (s, 3H), 5.48 (dd, J = 13.5, 2.8 Hz, 1H), 6.39 (d, J = 2.5 Hz, 1H), 6.49 (d, J = 2.5 Hz, 1H), 7.36-7.48 (m, 5H), 7.98 (s, 2H); ^{13}C-NMR (125 MHz, $CDCl_3$): 20.4, 20.8, 45.1, 56.0, 79.8, 100.1, 105.0, 108.1, 123.1, 126.3, 128.1, 129.0, 138.5, 139.2, 143.6, 151.8, 163.2, 164.3, 165.6, 166.5, 167.7, 188.7; HRMS (ESI) calcd for $C_{29}H_{25}O_{11}$ $[M+H]^+$: m/z 549.1397 Found 549.1388. LC R_t: 3.511 min. Purity >99.9%.

5.6 Synthesis of 7-*O*-(*O*-acetylsalicyloyl)pinocembrin (33)

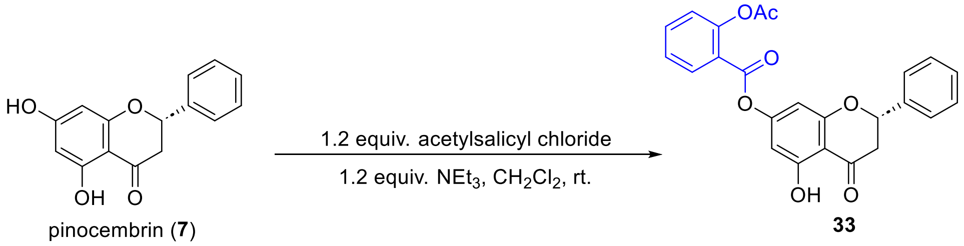

Following general procedure E, *O*-acetylsalicylic acid (168.7 mg, 0.9365 mmol) was reacted with $SOCl_2$ (679.3 µL, 9.3648 mmol). The generating *O*-acetylsalicyloyl chloride was redissolved in CH_2Cl_2 followed by adding pinocembrin (**7**) (200.0 mg, 0.7804 mmol) and NEt_3 (130.5 µL, 0.9365 mmol). The product was obtained after purification through column chromatography using EtOAc:*n*hexane = 2:8 to yield compound **33** in 88 %yield. white solids; ν_{max} (ATR): 1771 (C=O), 1748 (C=O), 1652 (C=O), 1633 (C=C), 1610 (C=C), 1585 (C=C),

1502 (C=C), 1281 (C-O), 1240 (C-O), 1218 (C-O), 1184 (C-O), 1163 (C-O), 1125 (C-O), 1091 (C-O), 1062 (C-O), 1036 (C-O); ^{1}H-NMR (500 MHz, $CDCl_3$): 2.32 (s, 3H), 2.90 (dd, *J* = 17.2, 3.0 Hz, 1H), 3.15 (dd, *J* = 17.2, 13.3 Hz, 1H), 5.49 (dd, *J* = 13.3, 3.0 Hz, 1H), 6.42 (s, 2H), 7.18 (dd, *J* = 8.1, 1.0 Hz, 1H), 7.36-7.49 (m, 6H), 7.65 (ddd, *J* = 8.1, 7.5, 1.7 Hz, 1H), 8.17 (dd, *J* = 7.9, 1.7 Hz, 1H), 11.89 (s, 1H); ^{13}C-NMR (125 MHz, $CDCl_3$): 21.1, 43.7, 79.5, 102.0, 103.6, 106.6, 122.1, 124.3, 126.3, 126.4, 129.1, 129.2, 132.4, 135.1, 138.1, 151.5, 158.4, 161.9, 162.5, 163.6, 169.7, 197.2; HRMS (ESI) calcd for $C_{24}H_{19}O_7$ $[M+H]^+$: *m*/*z* 419.1131 Found 419.1135. LC R_t: 4.522 min. Purity >99.9%.

5.7 Synthesis of 7-*O*-(*O*-acetylvanilloyl)pinocembrin (34)

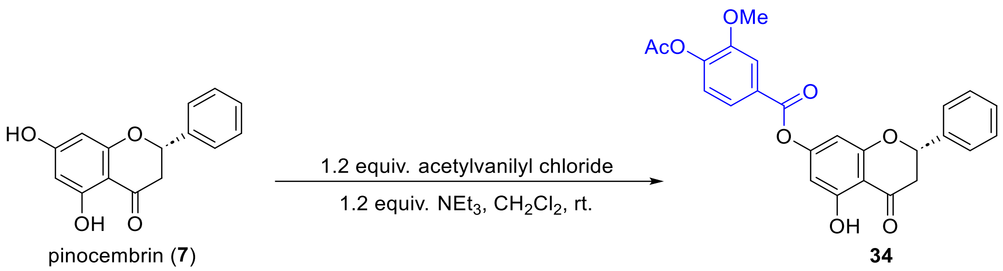

Following general procedure E, *O*-acetylvanilic acid (196.8 mg, 0.9365 mmol) was dissolved in CH_2Cl_2, then $SOCl_2$ (679.3 µL, 9.3648 mmol) was added to the solution, followed by refluxing for 1 h. The crude acyl chloride was dissolved in 5 mL of CH_2Cl_2, followed by adding pinocembrin (**7**) (200.0 mg, 0.7804 mmol) solution dissolved in 5 mL of CH_2Cl_2. The compound **34** was obtained in 92 %yield after purification through column chromatography using EtOAc:*n*hexane = 2:8. White solids; ν_{max} (ATR): 1743 (C=O), 1659 (C=O), 1631 (C=C), 1608 (C=C), 1507 (C=C), 1284 (C-O), 1241 (C-O), 1218 (C-O), 1187 (C-O), 1175 (C-O), 1124 (C-O), 1084 (C-O), 1063 (C-O), 1030 (C-O); ^{1}H-NMR (500 MHz, $CDCl_3$): 2.35 (s, 3H), 2.92 (dd, *J* = 17.2, 3.0 Hz, 1H), 3.17 (dd, *J* = 17.2, 13.2 Hz, 1H), 3.92 (s, 3H), 5.51 (dd, *J* = 13.2, 3.0 Hz, 1H), 6.44 (s, 2H), 7.17 (dd, *J* = 8.3, 1.0 Hz, 1H), 7.38-7.53 (m, 5H), 7.73 (d, *J* = 1.9 Hz, 1H), 7.81 (dd, *J* = 8.3, 1.9 Hz, 1H), 11.90 (s, 1H); ^{13}C-NMR (125 MHz, $CDCl_3$): 20.8, 43.8, 56.3, 79.5, 102.0, 103.6, 106.5, 114.1, 123.3, 123.6, 126.3, 127.6, 129.1, 129.2, 138.1, 144.7, 151.5, 158.8, 162.5, 163.5, 163.6, 168.5, 197.2; HRMS (ESI) calcd for $C_{25}H_{21}O_8$ $[M+H]^+$: *m*/*z* 449.1236 Found 449.1239. LC R_t: 4.519 min. Purity >99.9%.

5.8 Synthesis of 7-*O*-(tri-*O*-acetylgalloyl)pinocembrin (35)

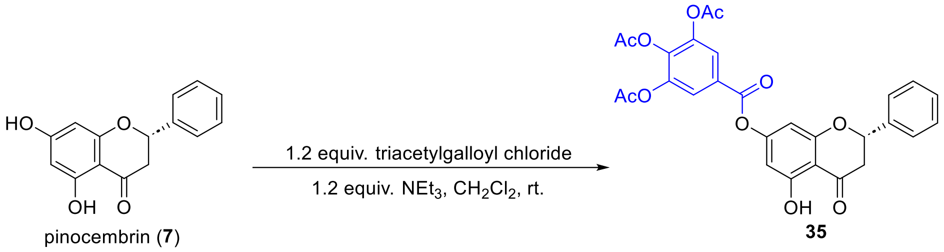

Following general procedure E, 3,4,5-triacetylgallic acid (277.4 mg, 0.9365 mmol) in 5 mL of CH_2Cl_2 was reacted with $SOCl_2$ (679 µL, 9.3648 mmol). The crude acyl chloride was redissolved in 5 mL of CH_2Cl_2. Then, the solution of pinocembrin (**7**) (200.0 mg, 0.7804 mmol) in CH_2Cl_2 and NEt_3 (130.5 mg, 0.9365 mmol) was added to the reaction. Until the completion of reaction, compound **35** was obtained after purified through column chromatography using EtOAc:*n*hexane = 4:6 in 90 %yield. white solids; ν_{max} (ATR): 2993 (CH_3), 2976 (CH_2), 2907 (CH_3), 1785 (C=O), 1755 (C=O), 1640 (C=O), 1587 (C=C), 1327 (C-O), 1178 (C-O), 1151 (C-O), 1127 (C-O), 1097 (C-O), 1052 (C-O), 1015 (C-O); ^{1}H-NMR (500 MHz, $CDCl_3$): δ 2.32 (s, 6H), 2.33 (s, 3H), 2.91 (dd, J = 17.2, 3.0 Hz, 1H), 3.16 (dd, J = 17.2, 13.2 Hz, 1H), 5.50 (dd, J = 13.2, 3.0 Hz, 1H), 6.41 (m, 2H), 7.36-7.53 (m, 5H), 7.92 (s, 2H), 11.89 (s, 1H); ^{13}C-NMR (125 MHz, $CDCl_3$): δ 20.3, 20.7, 43.7, 79.5, 101.9, 103.5, 106.6, 123.1, 126.3, 127.1, 129.1, 129.2, 138.1, 139.7, 143.8, 158.3, 162.1, 162.5, 163.5, 163.6, 166.5, 167.7, 197.2; HRMS (ESI) calcd for $C_{28}H_{23}O_{11}$ $[M+H]^+$: m/z 535.1240 Found 535.1227. LC R_t: 3.530 min. Purity >99.9%.

5.9 Synthesis of 4′-*O*-(tri-*O*-acetylgalloyl)cardamonin (37)

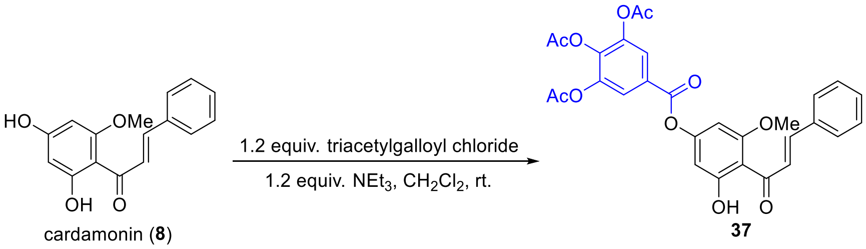

Compound **37** was synthesized following general procedure E, 3,4,5-triacetylgallic acid (263.0 mg, 0.8879 mmol) was reacted with $SOCl_2$ (644 µL, 8.8795 mmol). Until the completion of reaction, the crude acyl chloride was reacted with cardamonin (**8**) (200.0 mg, 0.7399 mmol) and NEt_3 (124 µL, 0.8879 mmol). The product was obtained in 81 %yield after purification through precipitation using EtOAc:*n*hexane. Orange solids; ν_{max} (ATR): 1780 (C=O), 1747 (C=O), 1638 (C=O), 1614 (C=C), 1587 (C=C), 1567 (C=C), 1325 (C-O), 1185 (C-O), 1166 (C-O), 1134 (C-O), 1115 (C-O), 1056 (C-O); ^{1}H-NMR (500 MHz, $CDCl_3$): δ 2.33 (s, 6H), 2.34 (s, 3H), 3.96 (s, 3H), 6.31 (d, J = 2.2 Hz, 1H), 6.49 (d, J = 2.2 Hz, 1H), 7.38-7.47

(m, 3H), 7.59-7.65 (m, 2H), 7.85 (m, 2H), 7.95 (s, 2H), 13.56 (s, 1H); ^{13}C-NMR (125 MHz, $CDCl_3$): *δ* 20.3, 20.7, 56.4, 96.4, 104.2, 110.2, 123.0, 127.3 ,128.7, 129.1, 130.6, 135.4, 139.6, 143.6, 143.8, 156.7, 162.3, 162.4, 166.5, 166.6, 167.8, 193.8; HRMS (ESI) calcd for $C_{29}H_{25}O_{11}$ $[M+H]^+$: *m*/*z* 549.1397 Found 549.1396. LC R_t: 3.940 min. Purity 99.2%.

5.10 Synthesis of 4′-*O*-(tri-*O*-acetylgalloyl)-DMC (39)

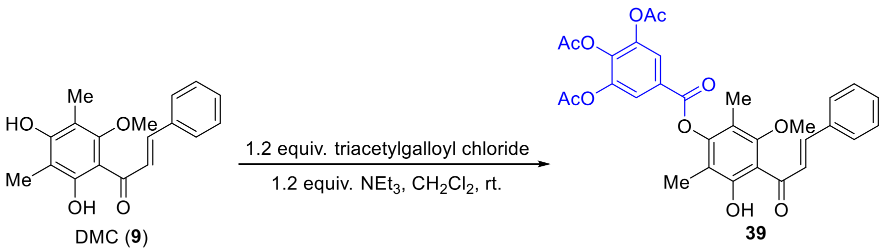

Compound **39** was synthesized following general procedure E, 3,4,5-triacetylgallic acid (238.3 mg, 0.8044 mmol) was reacted with $SOCl_2$ (486 µL, 8.0445 mmol). The acyl chloride product was then reacted with DMC (**9**) (200.0 mg, 0.6704 mmol) and NEt_3 (112 µL, 0.8044 mmol). The product was purified through precipitation using EtOAc:*n*hexane to yield compound **39** in 86 %yield. orange solids; ν_{max} (ATR): 1779 (C=O), 1742 (C=O), 1633 (C=O), 1606 (C=C), 1562 (C=C), 1330 (C-O), 1284 (C-O), 1181 (C-O), 1158 (C-O), 1133 (C-O), 1113 (C-O), 1055 (C-O); ^{1}H-NMR (400 MHz, $CDCl_3$): *δ* 2.07 (s, 3H), 2.08 (s, 3H), 2.34 (s, 6H), 2.35 (s, 3H), 3.69 (s, 3H), 7.40-7.45 (m, 3H), 7.64-7.69 (m, 2H), 7.90 (d, *J* = 15.7 Hz, 1H), 7.97 (d, *J* = 15.7 Hz, 1H), 12.96 (s, 1H); ^{13}C-NMR (100 MHz, $CDCl_3$): *δ* 9.3, 9.4, 20.4, 20.7, 62.8, 113.7, 115.4, 116.0, 123.1, 126.5, 126.9, 128.8, 129.2, 130.7, 135.2, 139.7, 143.9, 144.1, 153.9, 158.4, 161.0, 161.8, 166.5, 167.8, 194.6; HRMS (ESI) calcd for $C_{31}H_{29}O_{11}$ $[M+H]^+$: *m*/*z* 577.1710 Found 577.1710. LC R_t: 4.071 min. Purity 95.7%.

5.11 Synthesis of 5-*O*-(tri-*O*-acetylgalloyl)tectochrysin (41)

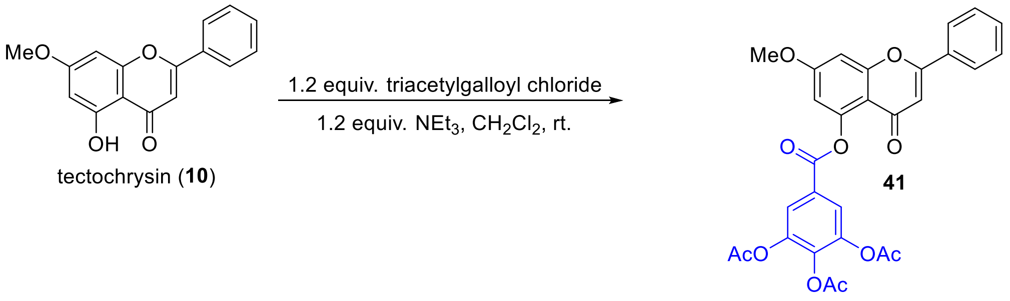

Following general procedure E, 3,4,5-triacetylgallic acid (265.0 mg, 0.8946 mmol) was reacted with $SOCl_2$ (649 µL, 8.9460 mmol). The acyl chloride product was dissolved in CH_2Cl_2 followed by adding tectochrysin (200.0 mg, 0.7455 mmol) and NEt_3 (125 µL, 0.8946 mmol). The product **41** was obtained in 94 %yield after precipitation using EtOAc:*n*hexane. Light-

yellow solids; ν_{max} (ATR): 1778 (C=O), 1741 (C=O), 1642 (C=O), 1610 (C=C), 1331 (C-O), 1187 (C-O), 1157 (C-O), 1110 (C-O), 1060 (C-O); ^{1}H-NMR (500 MHz, $CDCl_3$): δ 2.30 (s, 6H), 2.32 (s, 3H), 3.94 (s, 3H), 6.58 (s, 1H), 6.73 (d, J = 2.4 Hz, 1H), 6.94 (d, J = 2.4 Hz, 1H), 7.46-7.57 (m, 3H), 7.82-7.89 (m, 2H), 8.02 (s, 2H); ^{13}C-NMR (125 MHz, $CDCl_3$): δ 20.4, 20.7, 56.2 ,99.6, 108.5, 108.6, 111.2, 123.1, 126.3, 128.1, 129.2, 131.5, 131.7, 139.1, 143.6, 150.5, 159.0, 162.2, 163.4, 163.7, 166.5, 167.7, 176.4; HRMS (ESI) calcd for $C_{29}H_{23}O_{11}$ $[M+H]^+$: *m/z* 547.1240 Found 547.1231. LC R_t: 3.961 min. Purity 98.2%.

5.12 Synthesis of 7-*O*-(tri-*O*-acetylgalloyl)chrysin (43)

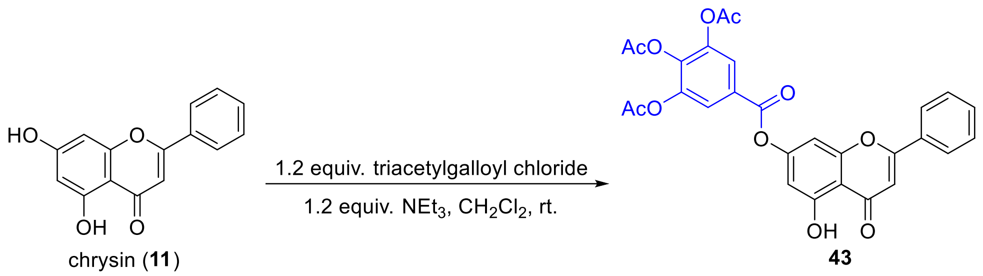

Compound **43** was synthesized following general procedure E. Shortly, 3,4,5-triacetylgallic acid (198.4 mg, 0.9439 mmol) was reacted with $SOCl_2$ (685 µL, 9.4390 mmol). The crude acyl chloride was then reacted with chrysin (**11**) (200.0 mg, 0.7866 mmol) and NEt_3 (132 µL, 0.9365 mmol). The product **43** was obtained in 91 %yield after precipitation using EtOAc:*n*hexane. Light-yellow solids; ν_{max} (ATR): 2993 (CH_3), 2978 (CH_2), 2906 (CH_3), 1776 (C=O), 1752 (C=O), 1655 (C=O), 1595 (C=C), 1327 (C-O), 1242 (C-O), 1184 (C-O), 1131 (C-O), 1060 (C-O), 1027 (C-O); ^{1}H-NMR (400 MHz, $CDCl_3$): δ 2.34 (s, 6H), 2.35 (s, 3H), 6.68 (d, J = 2.1 Hz, 1H), 6.76 (s, 1H), 6.95 (d, J = 2.1 Hz, 1H), 7.49-7.62 (m, 3H), 7.86-7.94 (m, 2H), 7.96 (s, 2H), 12.78 (s, 1H); ^{13}C-NMR (100 MHz, $CDCl_3$): δ 20.3, 20.7, 101.2, 105.6, 106.3, 109.3, 123.1, 126.6, 127.0, 129.3, 131.1, 132.4, 139.8, 143.9, 155.9, 156.9, 162.2, 162.3, 165.0, 166.5, 167.8, 183.0; HRMS (ESI) calcd for $C_{28}H_{21}O_{11}$ $[M+H]^+$: *m/z* 533.1084 Found 533.1075. LC R_t: 3.843 min. Purity >99.9%.

5.13 *General procedure F* for the synthesis of 5-*O*-galloylpinostrobin (31)

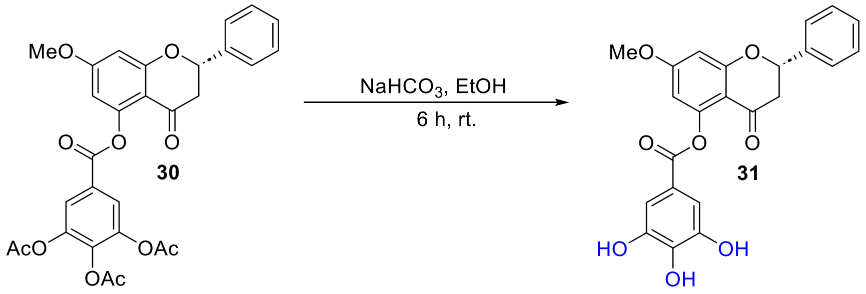

The deprotection of acetyl groups was conducted under basic conditions. In briefly, 70.0 mg of compound **30** was dissolved in 1 mL of EtOH in the presence of 50 mg of $NaHCO_3$. The reaction was stirred at room temperature for 6 h. Until the completion of reaction, the reaction was acidified using 6 M HCl to pH 3-4, followed by extracted several times with EtOAc. The combined organic layer was dried over anhydrous Na_2SO_4. The crude product was purified through size exclusion column chromatography on Sephadex LH-20 using MeOH as an eluent to yield **31** in 80 %yield. White solids; ν_{max} (ATR): 3475 (OH), 3420 (OH), 2993 (CH_2), 2978 (CH_3), 2907 (CH_2), 1719 (C=O), 1671 (C=O), 1614 (C=C), 1565 (C=C), 1336 (C-O), 1286 (C-O), 1235 (C-O), 1195 (C-O), 1158 (C-O), 1090 (C-O), 1083 (C-O), 1069 (C-O), 1048 (C-O), 1031 (C-O); ^{1}H-NMR (500 MHz, acetone-d_6): δ 2.66 (dd, J = 16.6, 2.9 Hz, 1H), 3.35 (dd, J = 16.6, 13.1 Hz, 1H), 3.91 (s, 3H), 5.59 (dd, J = 13.1, 2.9 Hz, 1H), 6.47 (d, J = 2.5 Hz, 1H), 6.55 (d, J = 2.5 Hz, 1H), 7.27 (s, 2H), 7.38-7.42 (m, 1H), 7.43-7.49 (m, 3H), 7.55-7.68 (m, 2H), 8.16 (br s, 1H), 8.29 (br s, 2H); ^{13}C-NMR (125 MHz, acetone-d_6): δ 44.7, 55.6, 79.4, 99.2, 104.7, 108.5, 109.8, 120.7, 126.4, 128.5, 128.6, 138.3, 139.3, 145.2, 152.6, 164.1, 164.2, 165.2, 187.6; HRMS (ESI) calcd for $C_{23}H_{19}O_8$ [M+H]$^+$: m/z 423.1080 Found 423.1071. LC R_t: 3.516 min. Purity >99.9%.

5.14 Synthesis of 7-*O*-galloylpinocembrin (36)

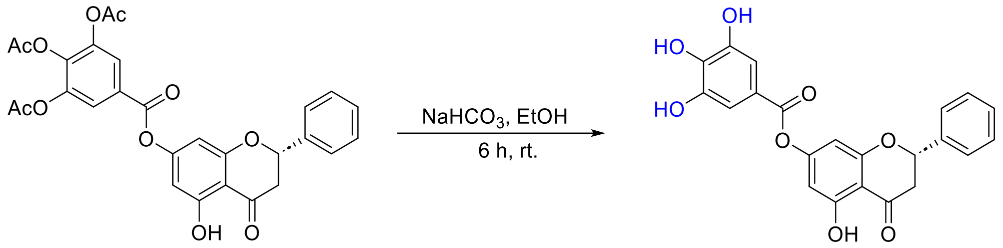

35

36

Compound **36** was synthesized from substrate **35** following general procedure F. White solids; ν_{max} (ATR): 3488 (OH), 3444 (OH), 2993 (CH_3), 2979 (CH_2), 2906 (CH_3), 1742 (C=O), 1638 (C=O), 1616 (C=C), 1578 (C=C), 1537 (C=C), 1375 (C-O), 1321 (C-O), 1298 (C-O), 1191 (C-O), 1174 (C-O), 1128 (C-O), 1089 (C-O), 1082 (C-O), 1069 (C-O), 1028 (C-O); ^{1}H-NMR (500 MHz, acetone-d_6): δ 2.95 (dd, J = 17.2, 3.0 Hz, 1H), 3.35 (dd, J = 17.2, 13.0 Hz, 1H), 5.73 (dd, J = 13.0, 3.0 Hz, 1H), 6.44 (d, J = 2.1 Hz, 1H), 6.48 (d, J = 2.1 Hz, 1H), 7.24 (s, 2H), 7.39-7.44 (m, 1H), 7.45-7.52 (m, 3H), 7.57-7.66 (m, 2H), 8.40 (br s, 3H), 12.03 (s, 1H); ^{13}C-NMR (125 MHz, acetone-d_6): δ 43.8, 80.2, 102.6, 103.8, 106.8, 110.6, 120.2, 127.4, 129.6, 139.7, 140.0, 146.3, 160.1, 163.4, 164.1, 164.4, 198.6; HRMS (ESI) calcd for $C_{22}H_{17}O_8$ [M+H]$^+$: m/z 409.0923 Found 409.0965. LC R_t: 3.668 min. Purity >99.9%.

5.15 Synthesis of 4′-*O*-galloylcardamonin (38)

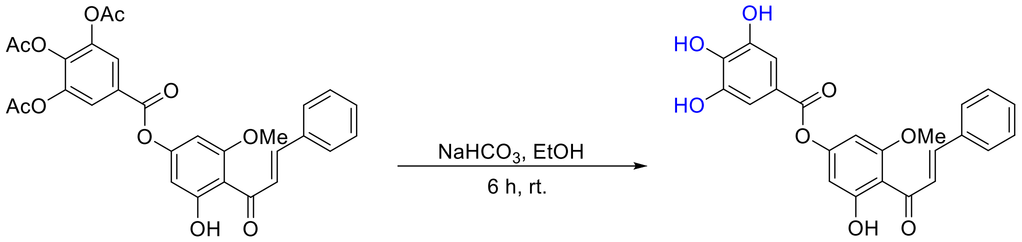

37 **38**

Following general procedure F, compound **38** was prepared from substrate **37**. Orange solids; ν_{max} (ATR): 3289 (OH), 2993 (CH_3), 2976 (CH_2), 2928 (CH_3), 2907 (CH_2), 1725 (C=O), 1634 (C=O), 1595 (C=C), 1568 (C=C), 1211 (C-O), 1189 (C-O), 1149 (C-O), 1115 (C-O), 1081 (C-O), 1070 (C-O), 1040 (C-O) cm^{-1}; ^{1}H-NMR (500 MHz, acetone-d_6): δ 4.05 (s, 3H), 6.50 (d, J = 2.1 Hz, 1H), 6.59 (d, J = 2.1 Hz, 1H), 7.26 (s, 2H), 7.43-7.51 (m, 3H), 7.75-7.79 (m, 2H), 7.83 (d, J = 15.7 Hz, 1H), 8.01 (d, J = 15.7 Hz, 1H), 8.45 (br s, 3H), 13.41 (s, 1H); ^{13}C-NMR (125 MHz, acetone-d_6): δ 57.0, 98.0, 104.5, 110.5, 120.3, 128.2, 129.5, 131.4, 136.2, 139.8, 144.0, 146.3, 158.7, 163.2, 164.5, 166.7, 194.6; HRMS (ESI) calcd for $C_{23}H_{19}O_8$ $[M+H]^+$: *m*/*z* 423.1080 Found 423.1075. LC R_t: 3.838 min. Purity 98.3%.

5.16 Synthesis of 4′-*O*-galloyl-DMC (40)

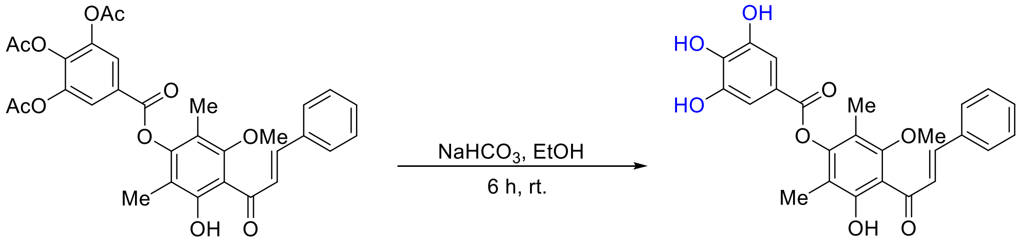

39 **40**

Following general procedure F, compound **40** was synthesized from starting material **39**. Orange solids; ν_{max} (ATR): 3409 (OH), 3219 (OH), 2994 (CH_3), 2977 (CH_2), 2907 (CH_3), 1730 (C=O), 1611 (C=O), 1538 (C=C), 1273 (C-O), 1237 (C-O), 1194 (C-O), 1137 (C-O), 1109 (C-O), 1084 (C-O); ^{1}H-NMR (500 MHz, $CDCl_3$): δ 2.01 (s, 3H), 2.03 (s, 3H), 3.66 (s, 3H), 6.41 (br s, 3H), 7.38 (s, 2H), 7.39-7.44 (m, 3H), 7.61-7.65 (m, 2H), 7.87 (d, J = 15.7 Hz, 1H), 7.93 (d, J = 15.7 Hz, 1H), 13.02 (s, 1H); ^{13}C-NMR (125 MHz, $CDCl_3$): δ 9.1, 9.3, 62.7, 110.7, 113.5, 115.8, 116.2, 119.8, 126.4, 128.8, 129.2, 130.8, 135.1, 137.8, 144.0, 144.3, 154.4, 158.3, 160.8, 164.5, 194.7; HRMS (ESI) calcd for $C_{25}H_{23}O_8$ $[M+H]^+$: *m*/*z* 451.1393 Found 451.1388. LC R_t: 3.960 min. Purity 95.0%.

5.17 Synthesis of 5-*O*-galloyltectochrysin (42)

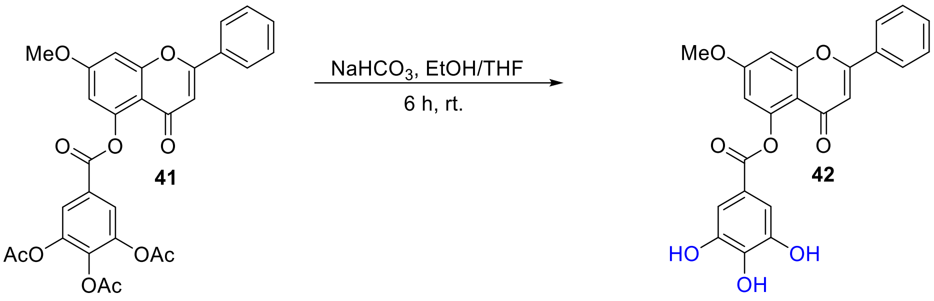

Following general procedure F, compound **41** was synthesized from starting material **42**. Light-yellow solids; ν_{max} (ATR): 3332 (OH), 2972 (CH_3), 1736 (C=O), 1634 (C=O), 1615 (C=C), 1572 (C=C), 1273 (C-O), 1162 (C-O), 1116 (C-O), 1098 (C-O), 1067 (C-O), 1035 (C-O); ^{1}H-NMR (500 MHz, DMSO-d_6): δ 3.94 (s, 3H), 6.76 (s, 1H), 6.89 (d, J = 2.5 Hz, 1H), 7.09 (s, 2H), 7.31 (d, J = 2.5 Hz, 1H), 7.54-7.66 (m, 3H), 8.06-8.11 (m, 2H), 9.24 (br s, 1H); ^{13}C-NMR (125 MHz, DMSO-d_6): δ 56.4, 99.5, 107.7, 108.7, 109.4, 111.1, 118.8, 126.2, 129.1, 130.8, 131.8, 139.0, 145.6, 150.4, 158.3, 161.0, 163.2, 164.4, 175.2; HRMS (ESI) calcd for $C_{23}H_{17}O_8$ [M+H]$^+$: m/z 421.0923 Found 421.0920. LC R_t: 3.897 min. Purity 97.7%.

5.18 Synthesis of 7-*O*-galloylchrysin (44)

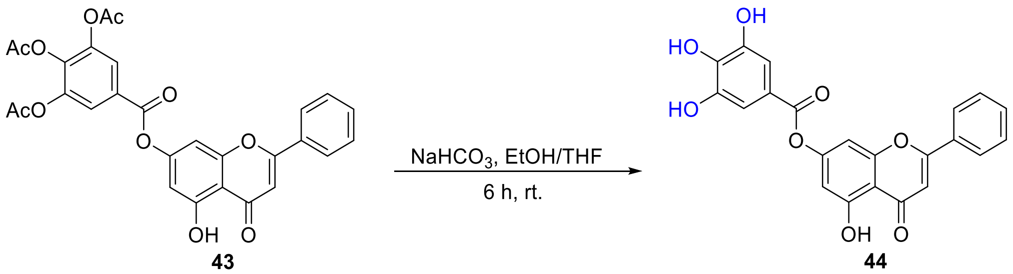

Following general procedure F, compound **43** was synthesized from starting material **44**. Light-yellow solids; ν_{max} (ATR): 1738 (C=O), 1651 (C=O), 1609 (C=C), 1585 (C=C), 1240 (C-O), 1198 (C-O), 1176 (C-O), 1139 (C-O), 1066 (C-O), 1033 (C-O); ^{1}H-NMR (500 MHz, DMSO-d_6): δ 6.78 (d, J = 2.0 Hz, 1H), 6.93 (s, 1H), 7.12 (s, 2H), 7.17 (s, 1H), 7.22 (d, J = 2.0 Hz, 1H), 7.58-7.67 (m, 3H), 8.11-8.16 (m, 2H), 9.22-9.55 (m, 3H), 12.87 (s, 1H); ^{13}C-NMR (125 MHz, DMSO-d_6): δ 101.9, 105.6, 105.8, 108.2, 108.5, 109.3, 117.5, 119.3, 126.7, 129.2, 130.4, 132.4, 138.4, 139.7, 145.6, 145.8, 156.4, 160.8, 163.8, 164.2, 166.3, 182.7; HRMS (ESI) calcd for $C_{22}H_{15}O_8$ [M+H]$^+$: m/z 407.0767 Found 407.0770. LC R_t: 3.855 min. Purity 99.3%.

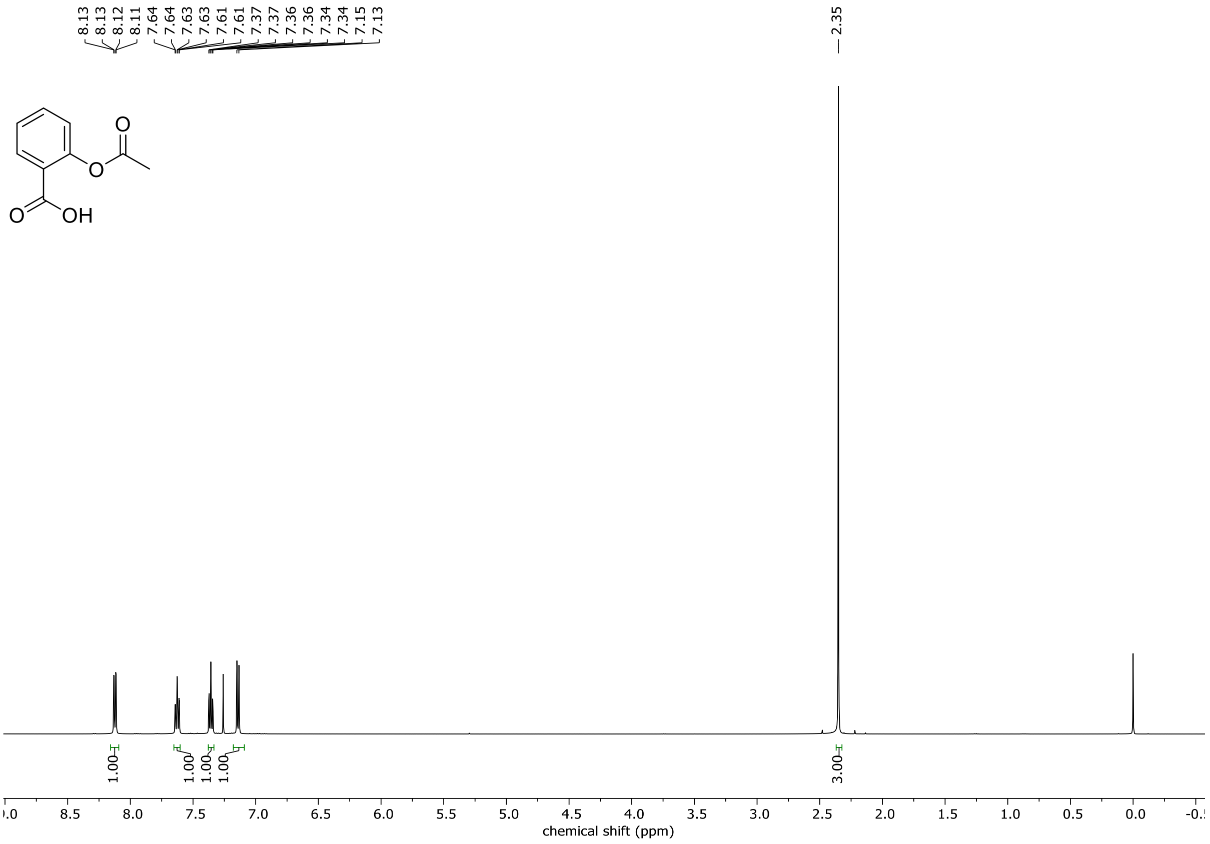

Figure S1. ^{1}H-NMR spectrum (500 MHz, $CDCl_3$) of acetylsalicylic acid.

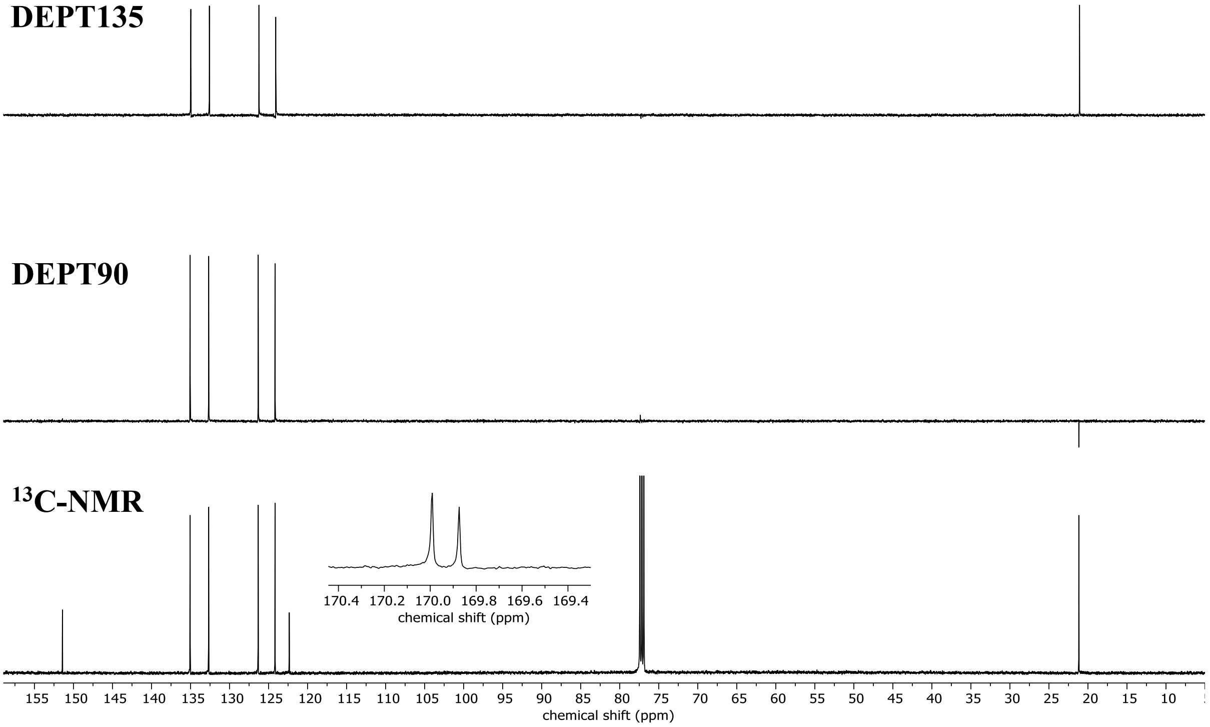

Figure S2. ^{13}C-NMR, DEPT90, and DEPT135 spectra (125 MHz, $CDCl_3$) of acetylsalicylic acid.

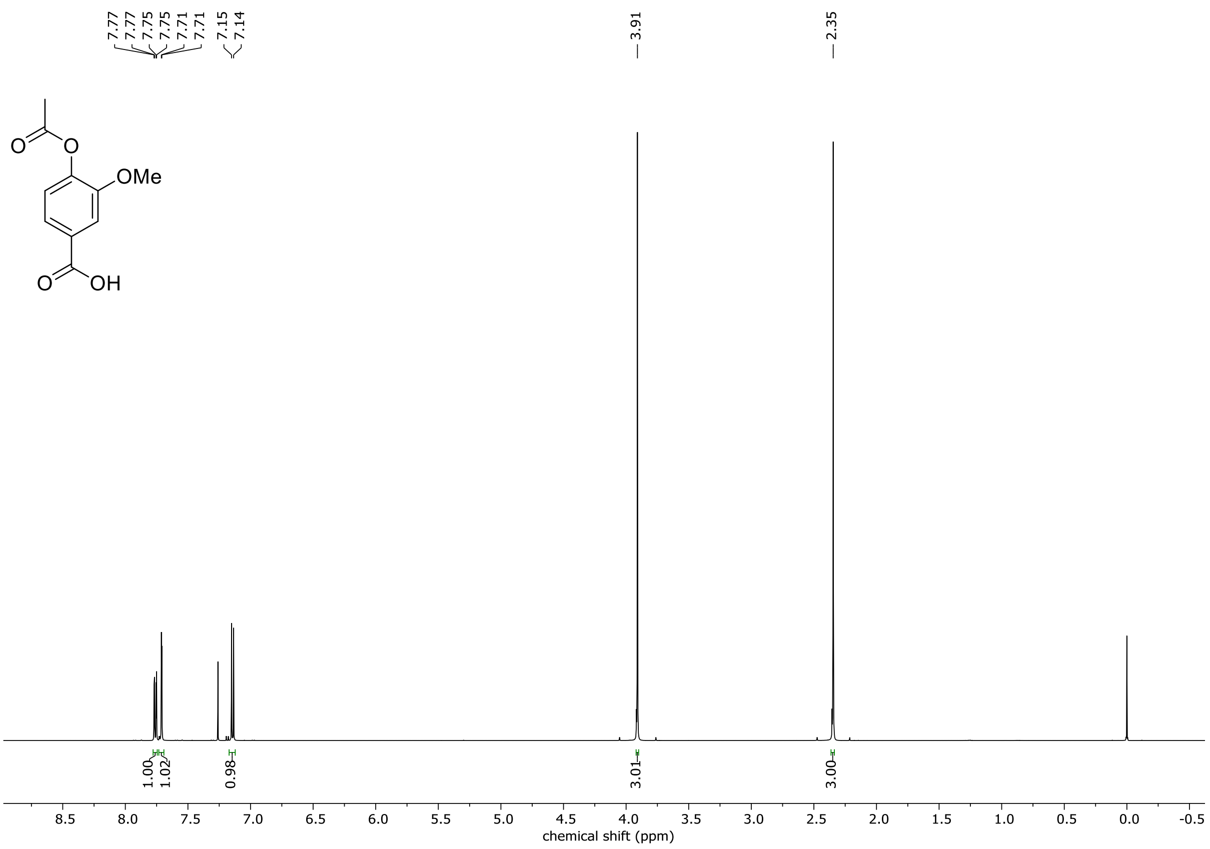

Figure S3. ^{1}H-NMR spectrum (500 MHz, $CDCl_3$) of acetylvanilic acid.

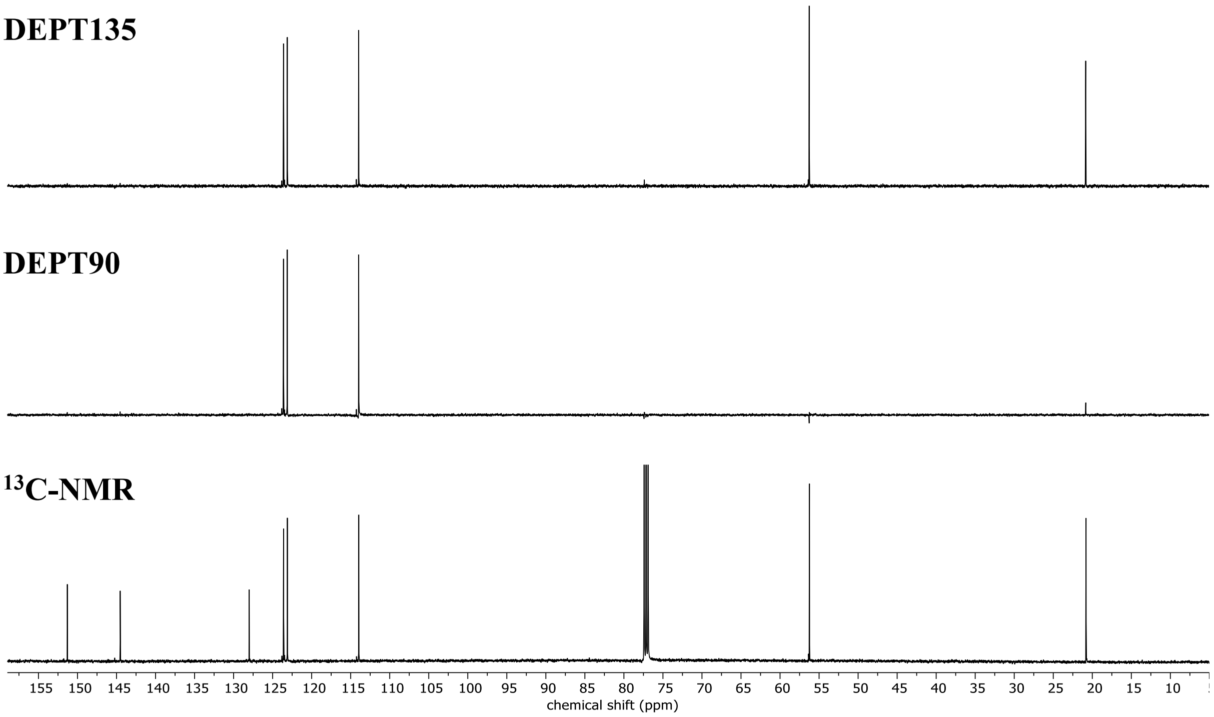

Figure S4. ^{13}C-NMR, DEPT90, and DEPT135 spectra (125 MHz, $CDCl_3$) of acetylvanilic acid.

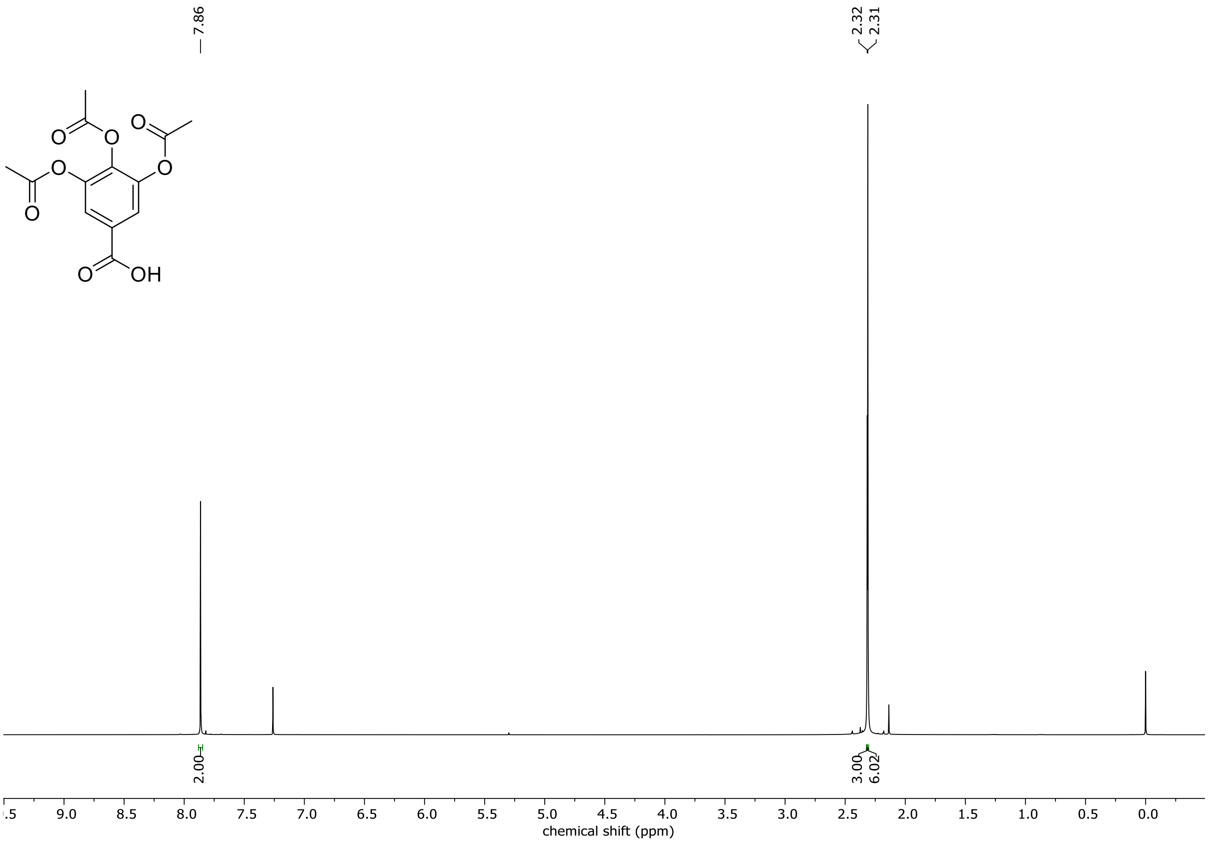

Figure S5. ^{1}H-NMR spectrum (500 MHz, $CDCl_3$) of triacetylgallic acid.

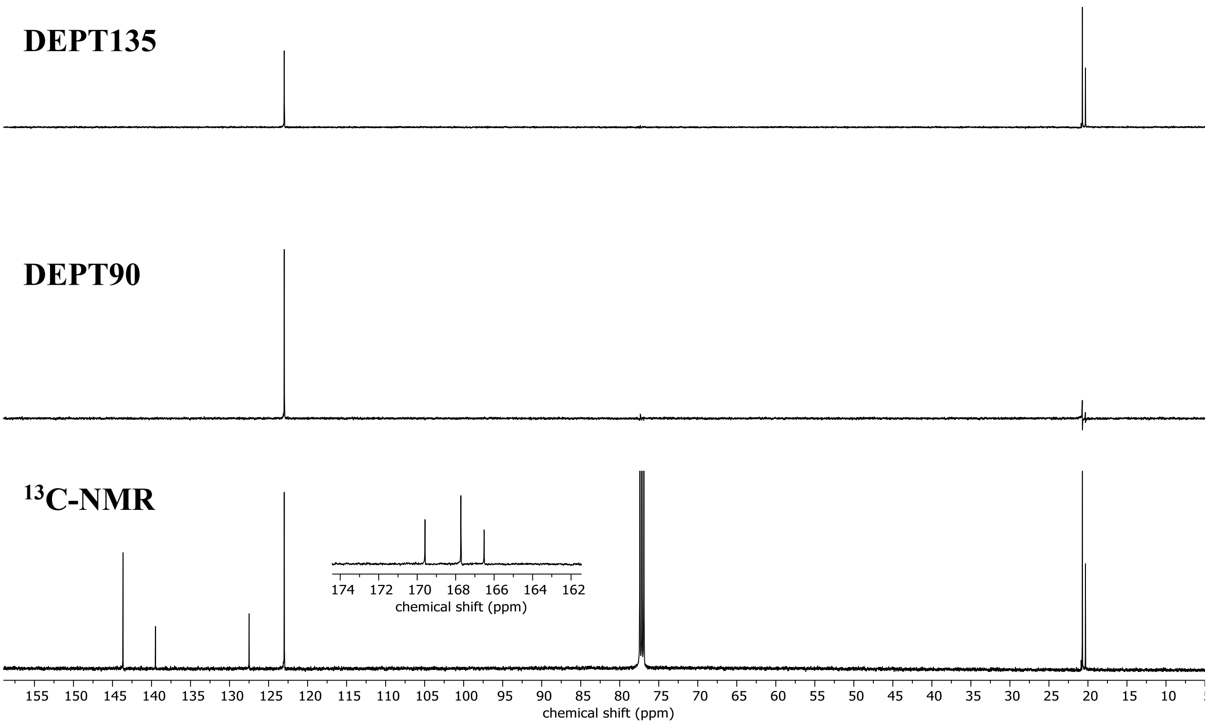

Figure S6. ^{13}C-NMR, DEPT90, and DEPT135 spectra (125 MHz, $CDCl_3$) of triacetylgallic acid.

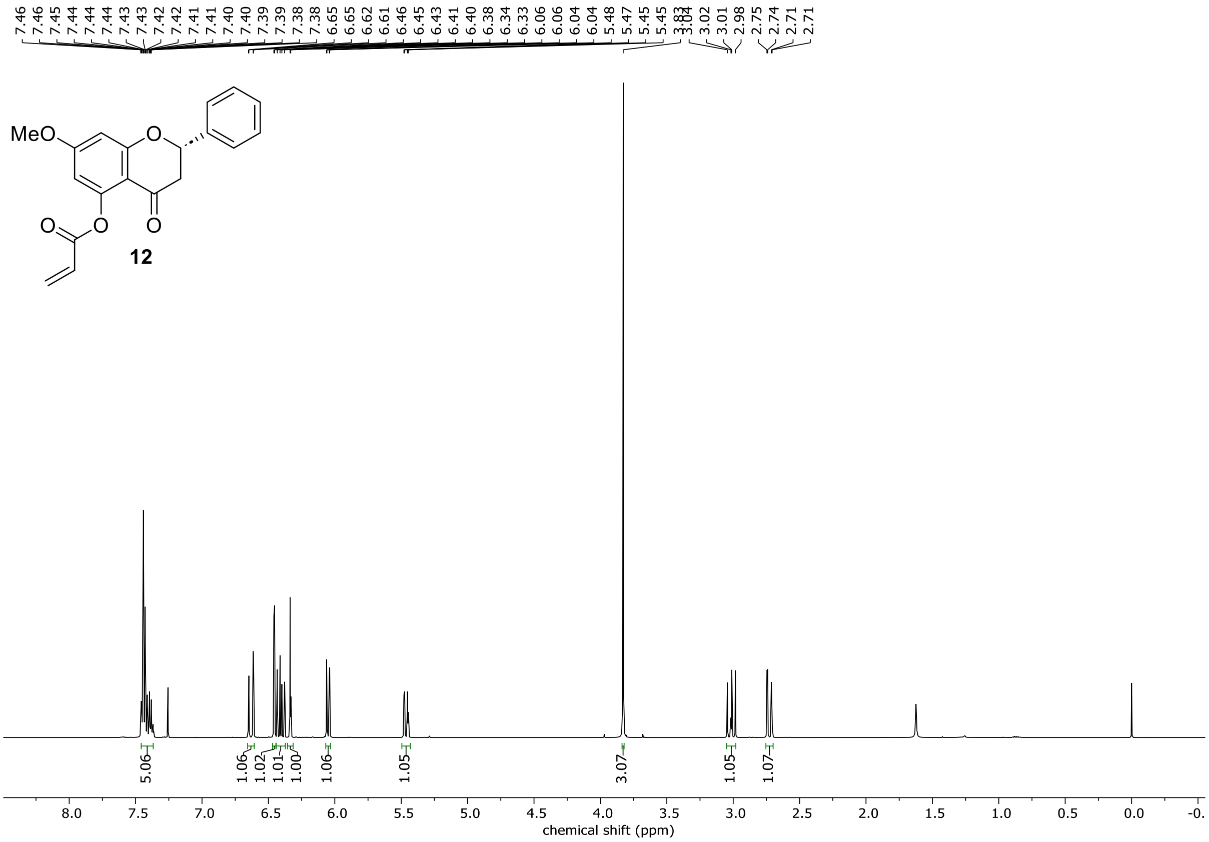

Figure S7. ^{1}H-NMR spectrum (500 MHz, $CDCl_3$) of 5-*O*-acryloylpinostrobin (**12**).

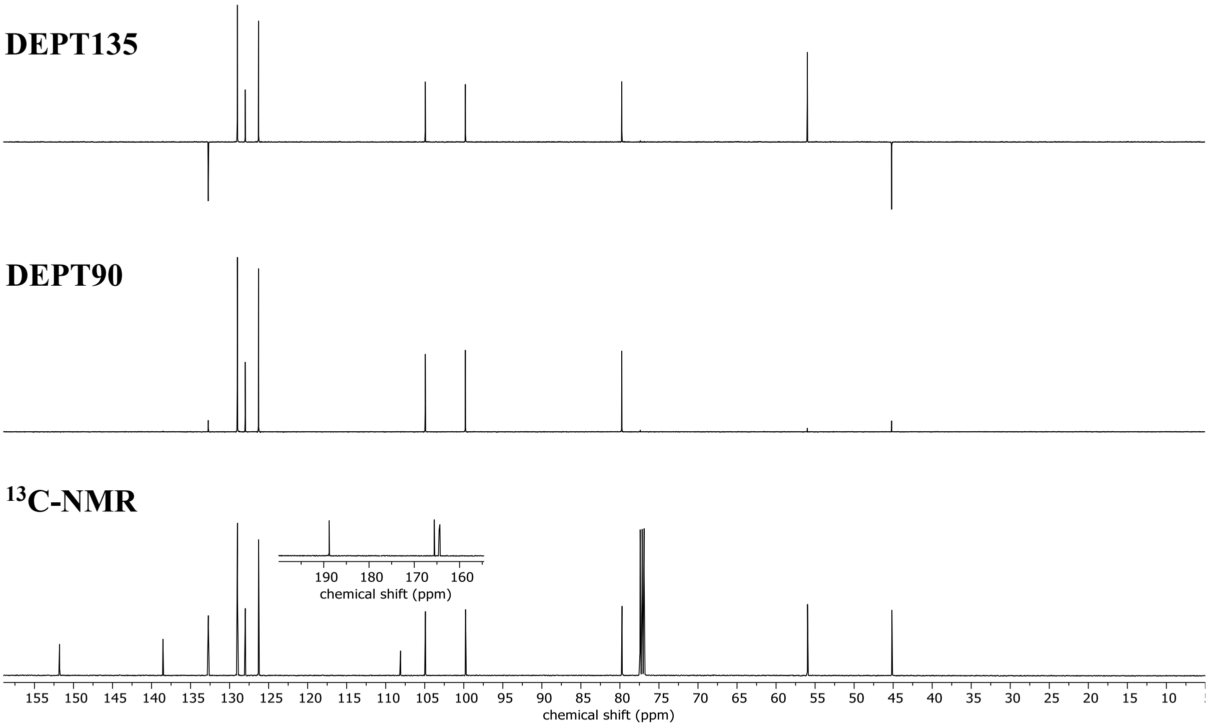

Figure S8. ^{13}C-NMR, DEPT90, and DEPT135 spectra (125 MHz, $CDCl_3$) of 5-*O*-acryloylpinostrobin (**12**).

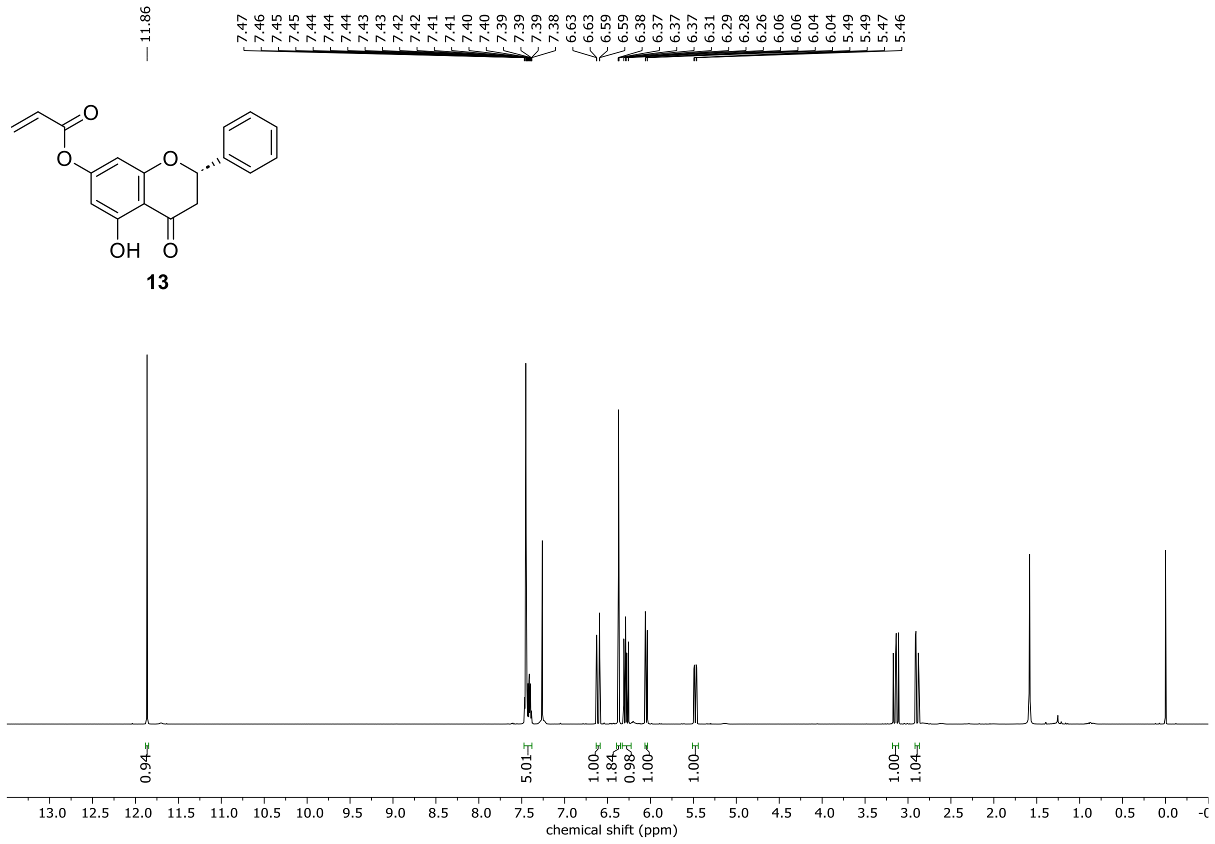

Figure S9. ^{1}H-NMR spectrum (500 MHz, $CDCl_3$) of 7-*O*-acryloylpinocembrin (**13**).

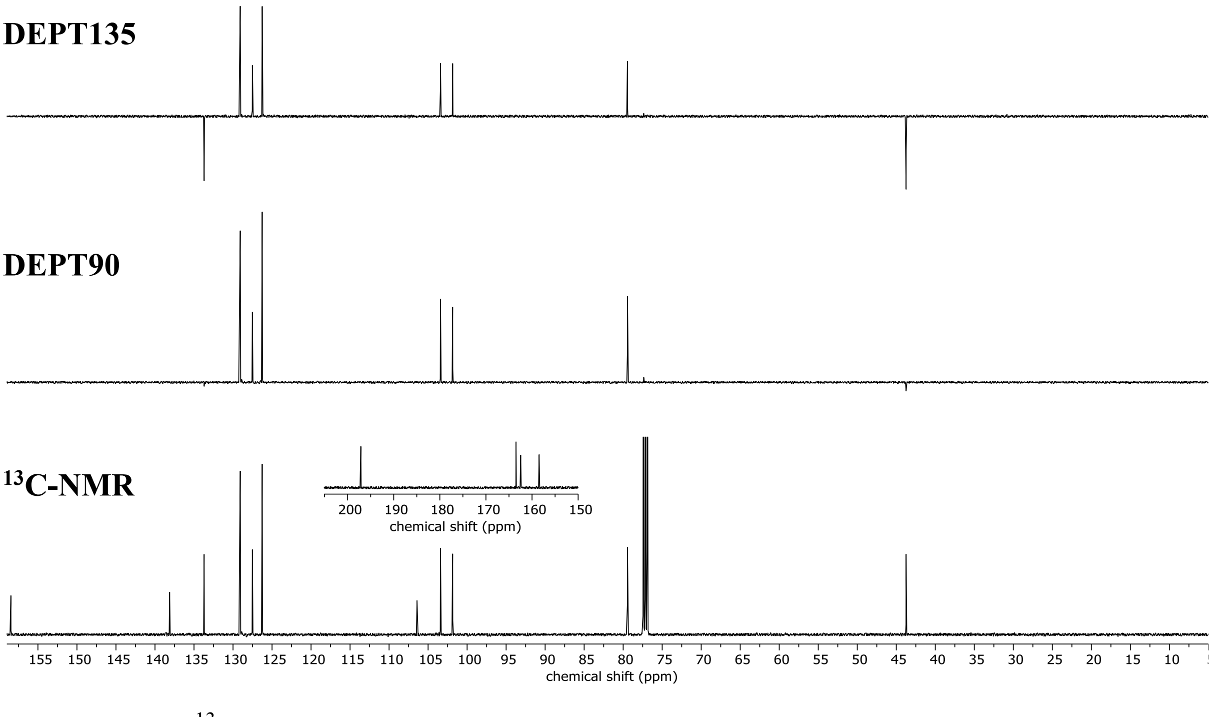

Figure S10. ^{13}C-NMR, DEPT90, and DEPT135 spectra (125 MHz, $CDCl_3$) of 7-*O*-acryloylpinocembrin (**13**).

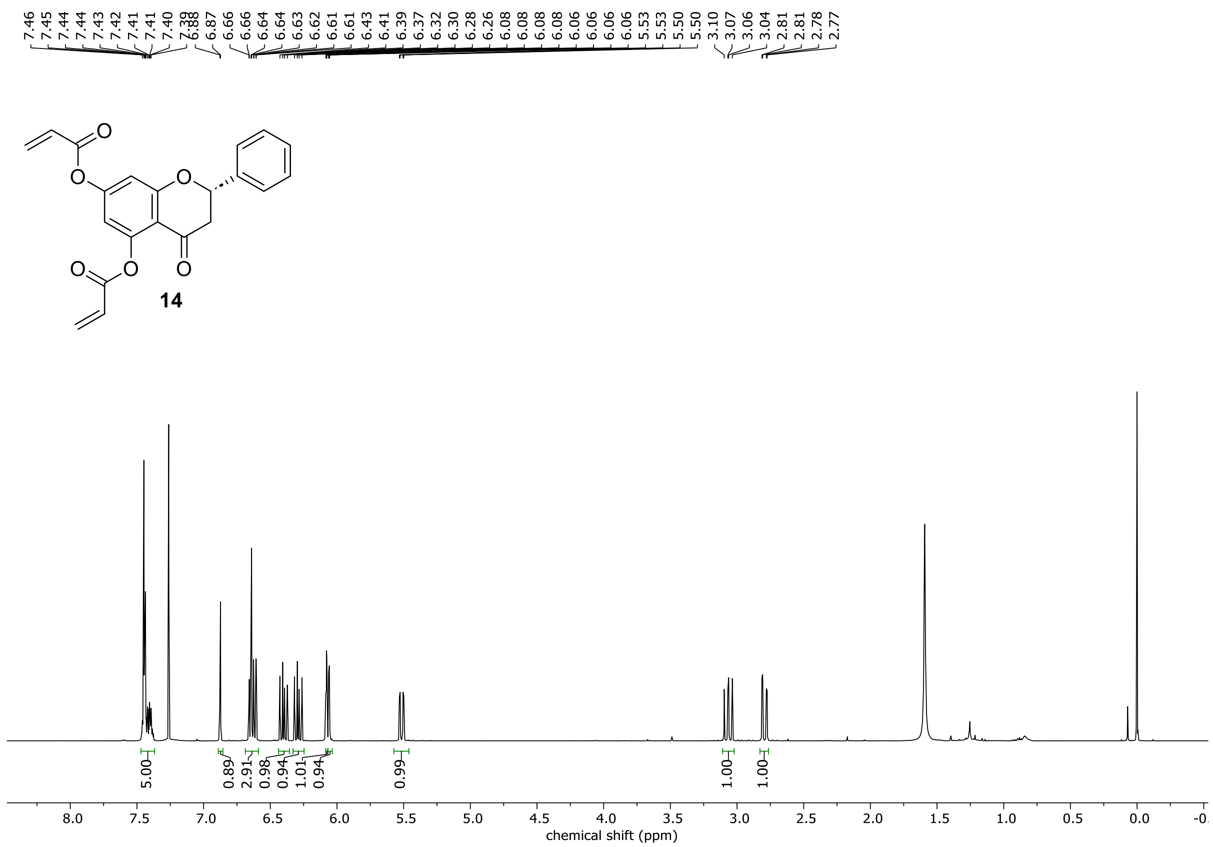

Figure S11. ^{1}H-NMR spectrum (500 MHz, $CDCl_3$) of 5,7-*O*-diacryloylpinocembrin (**14**).

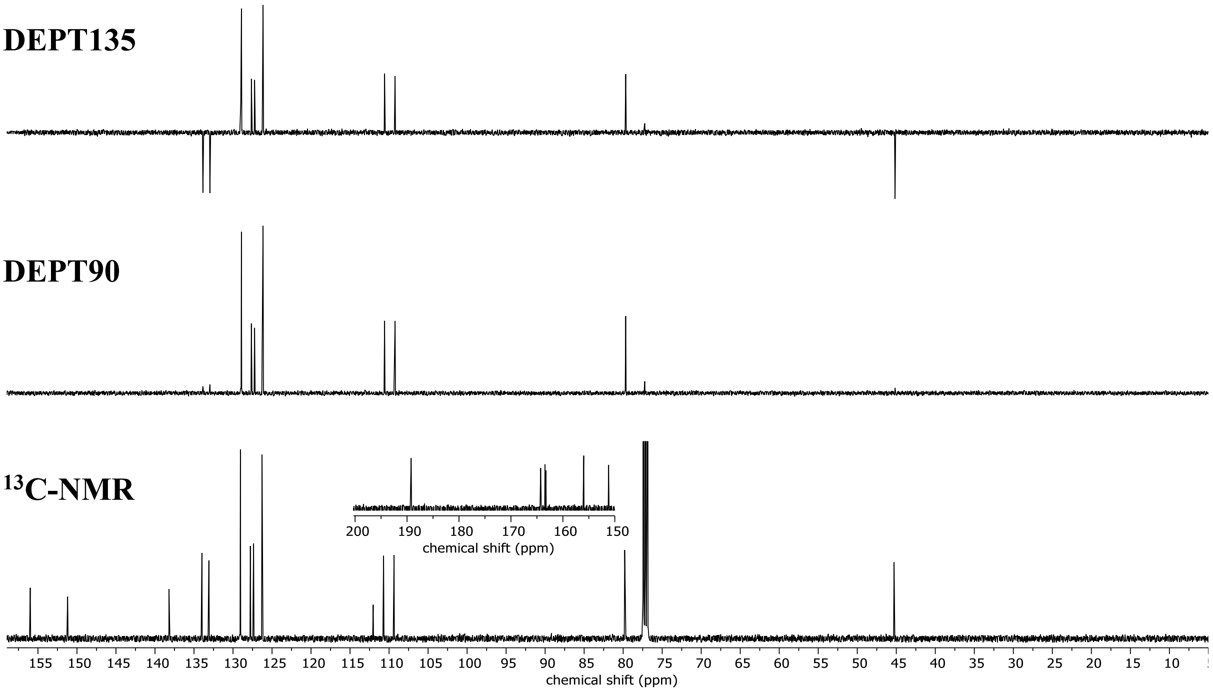

Figure S12. ^{13}C-NMR, DEPT90, and DEPT135 spectra (125 MHz, $CDCl_3$) of 5,7-*O*-diacryloylpinocembrin (**14**).

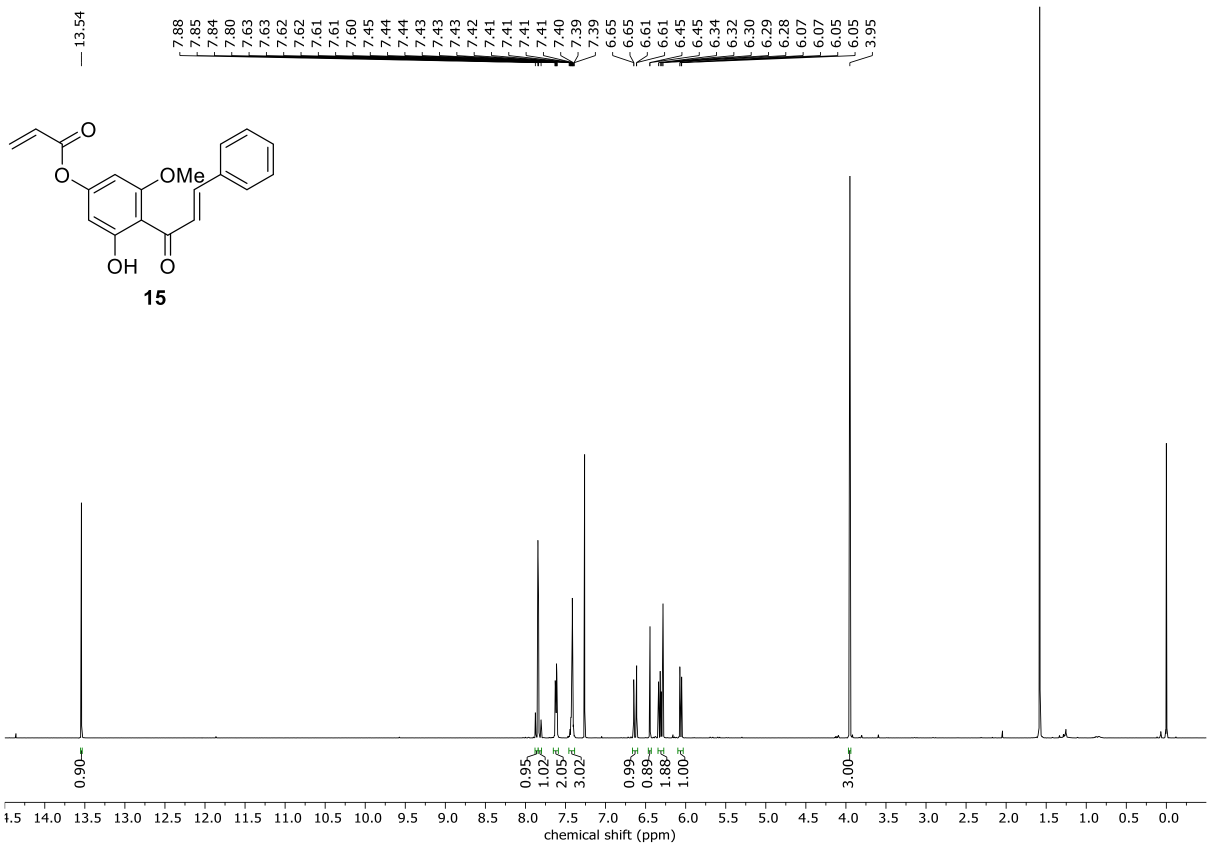

Figure S13. ^{1}H-NMR spectrum (500 MHz, $CDCl_3$) of 4′-*O*-acryloylcardamonin (**15**).

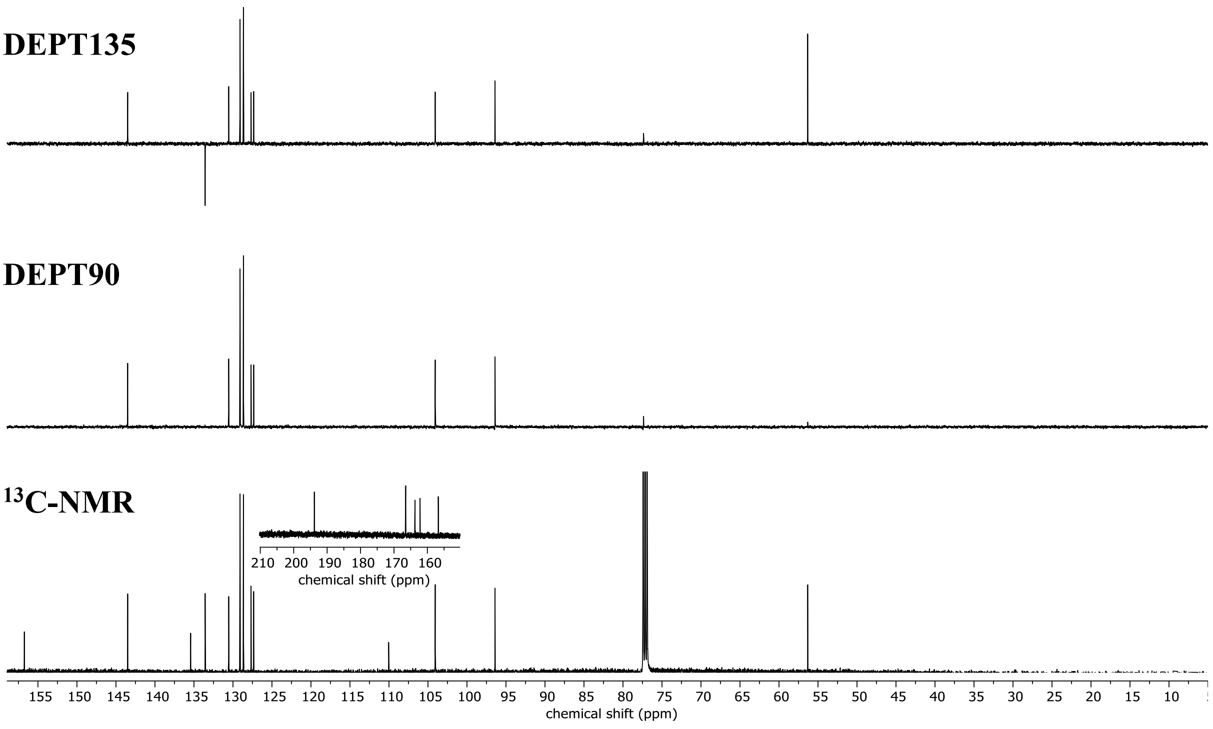

Figure S14. ^{13}C-NMR, DEPT90, and DEPT135 spectra (125 MHz, $CDCl_3$) of 4′-*O*-acryloylcardamonin (**15**).

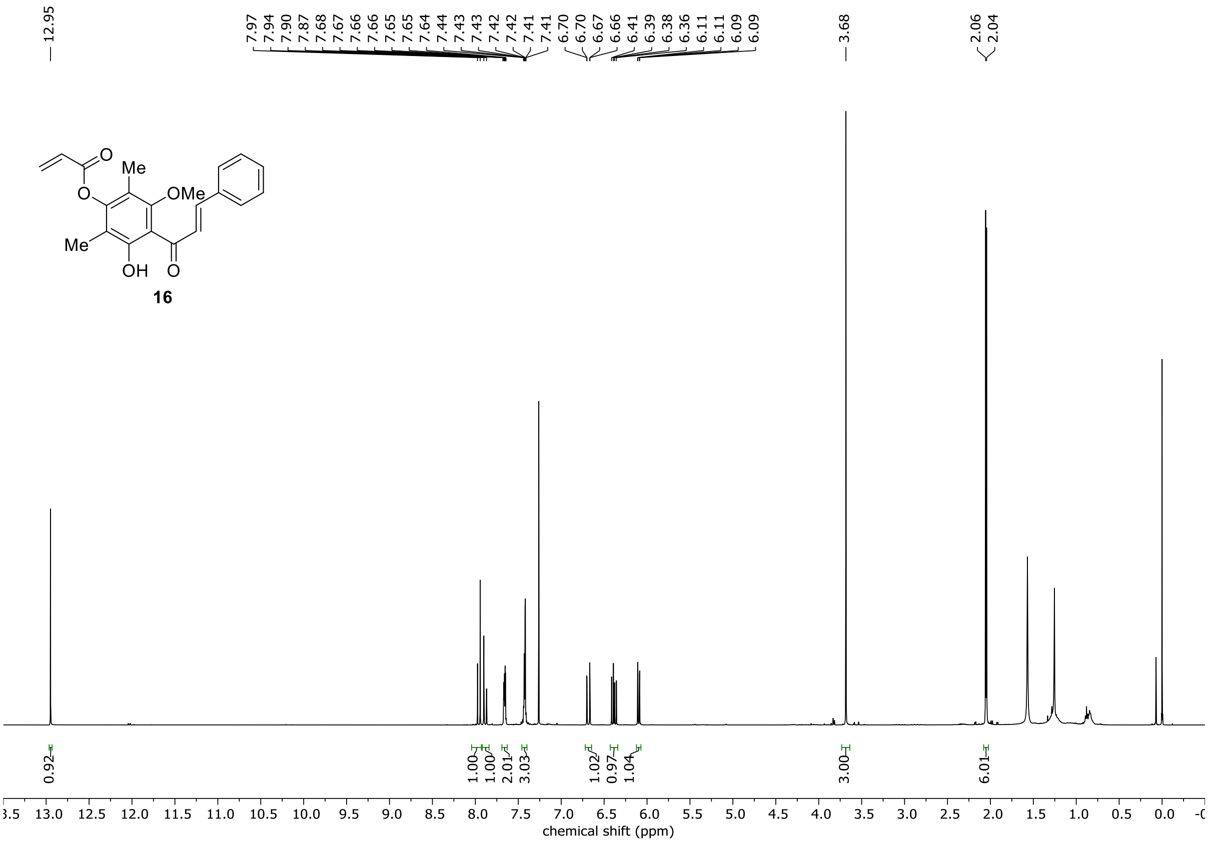

Figure S15. ¹H-NMR spectrum (500 MHz, $CDCl_3$) of 4′-*O*-acryloyl-DMC (**16**).

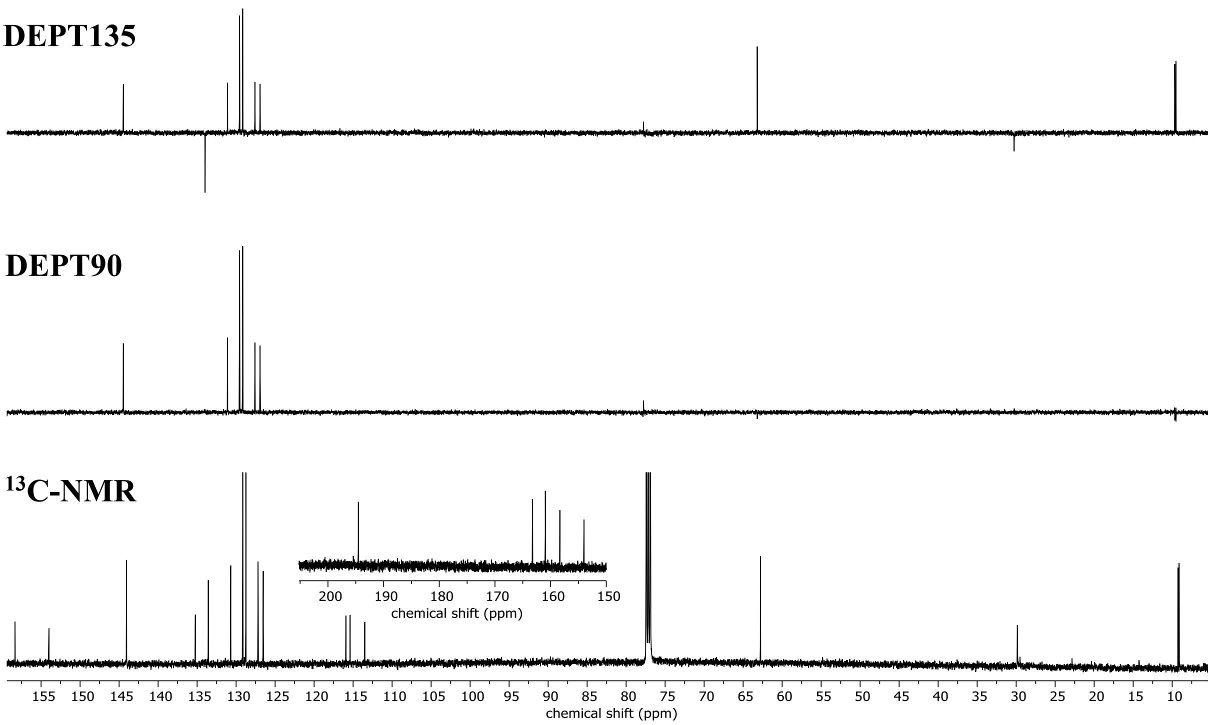

Figure S16. ¹³C-NMR, DEPT90, and DEPT135 spectra (125 MHz, $CDCl_3$) of 4′-*O*-acryloyl-DMC (**16**).

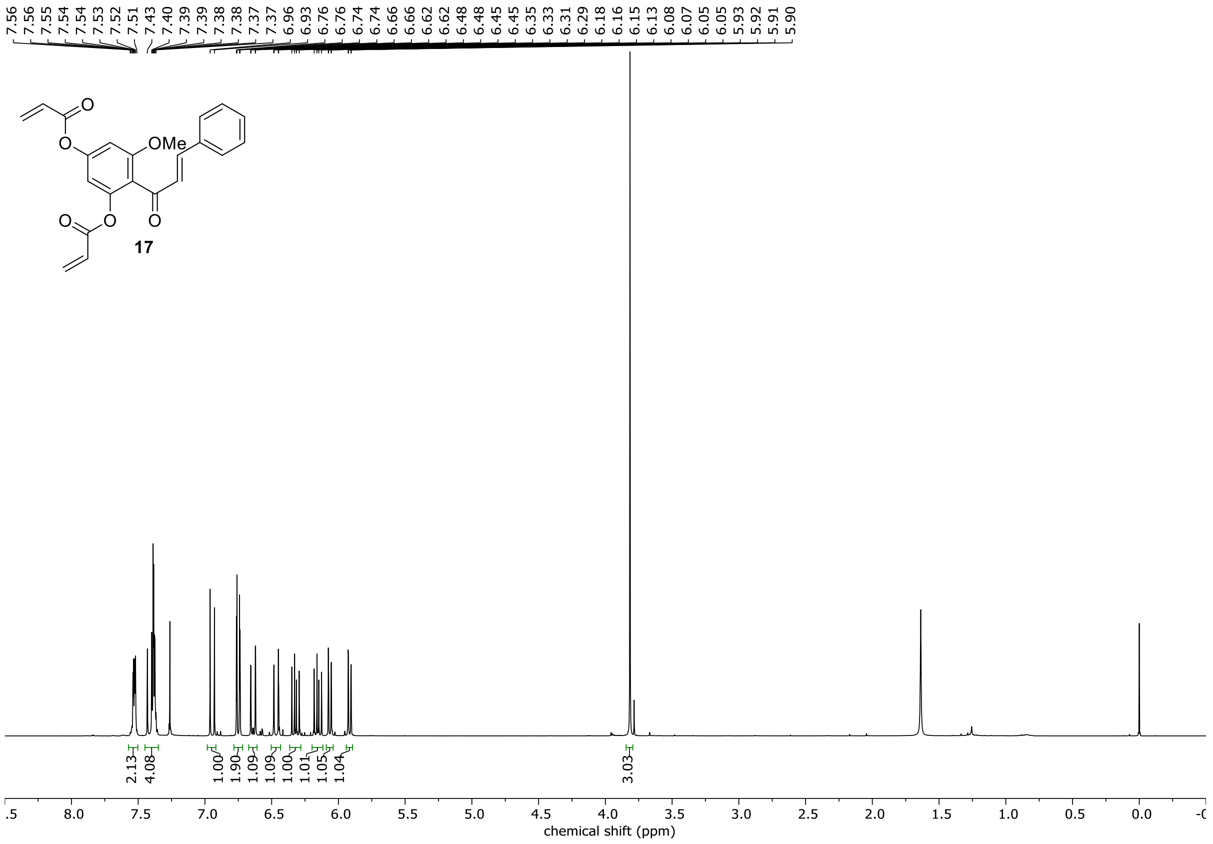

Figure S17. ^{1}H-NMR spectrum (500 MHz, $CDCl_3$) of 2′,4′-*O*-diacryloylcardamonin (**17**).

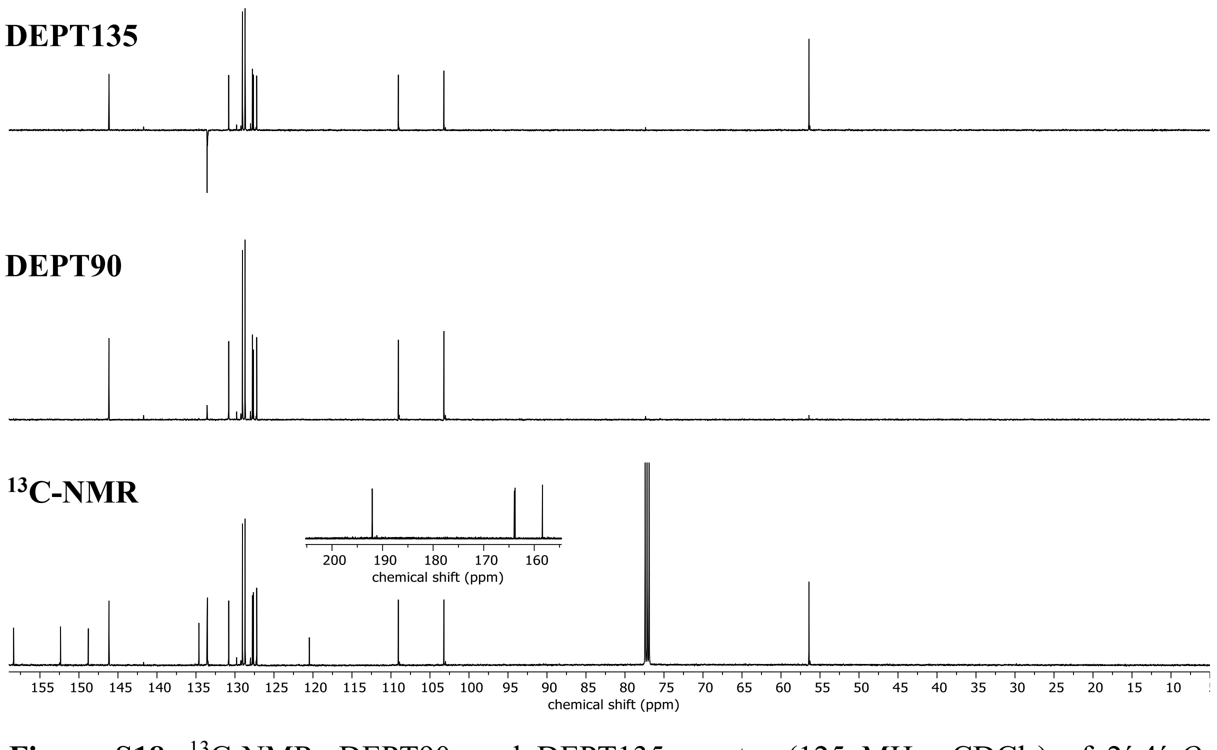

Figure S18. ^{13}C-NMR, DEPT90, and DEPT135 spectra (125 MHz, $CDCl_3$) of 2′,4′-*O*-diacryloylcardamonin (**17**).

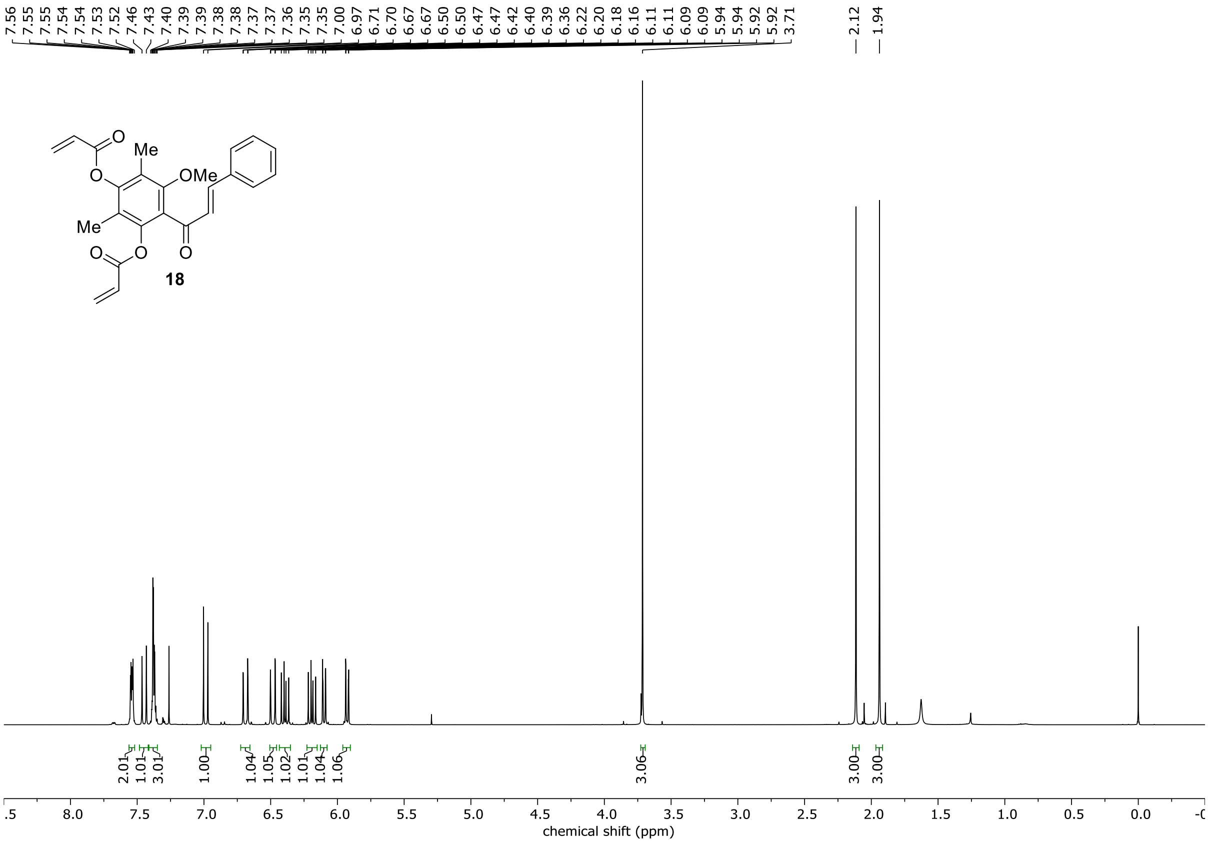

Figure S19. ^{1}H-NMR spectrum (500 MHz, $CDCl_3$) of 2′,4′-*O*-diacryloyl-DMC (**18**).

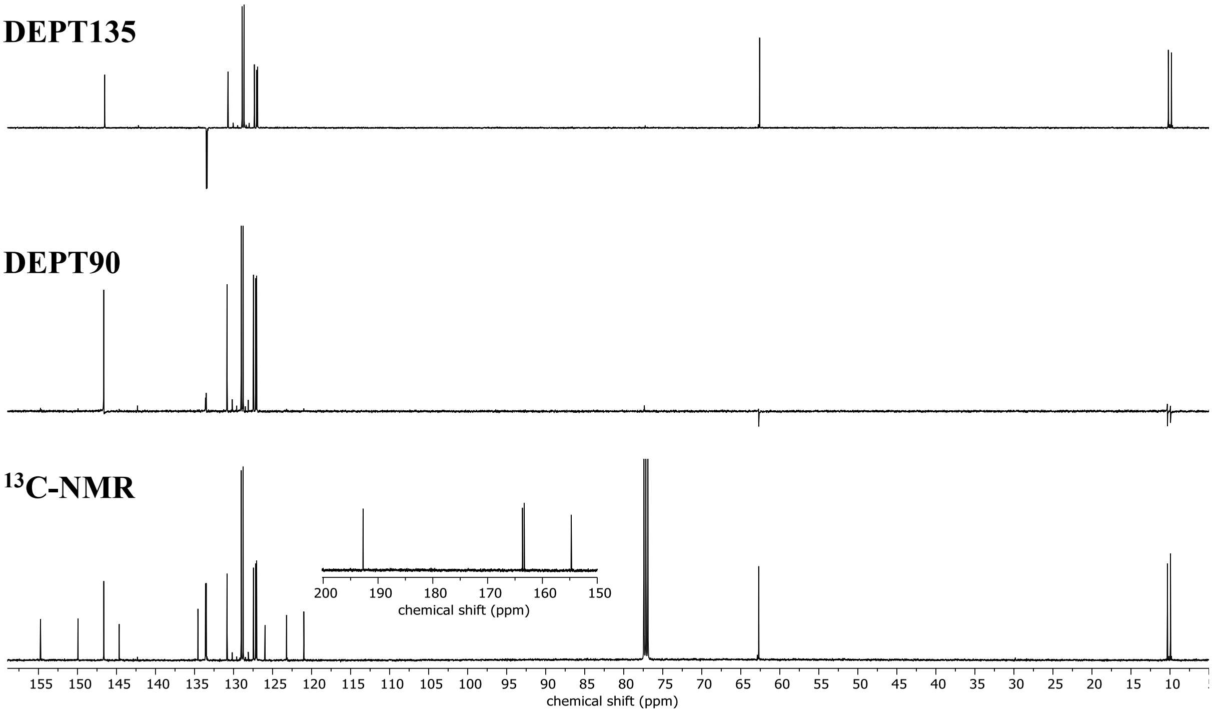

Figure S20. ^{13}C-NMR, DEPT90, and DEPT135 spectra (125 MHz, $CDCl_3$) of 2′,4′-*O*-diacryloyl-DMC (**18**).

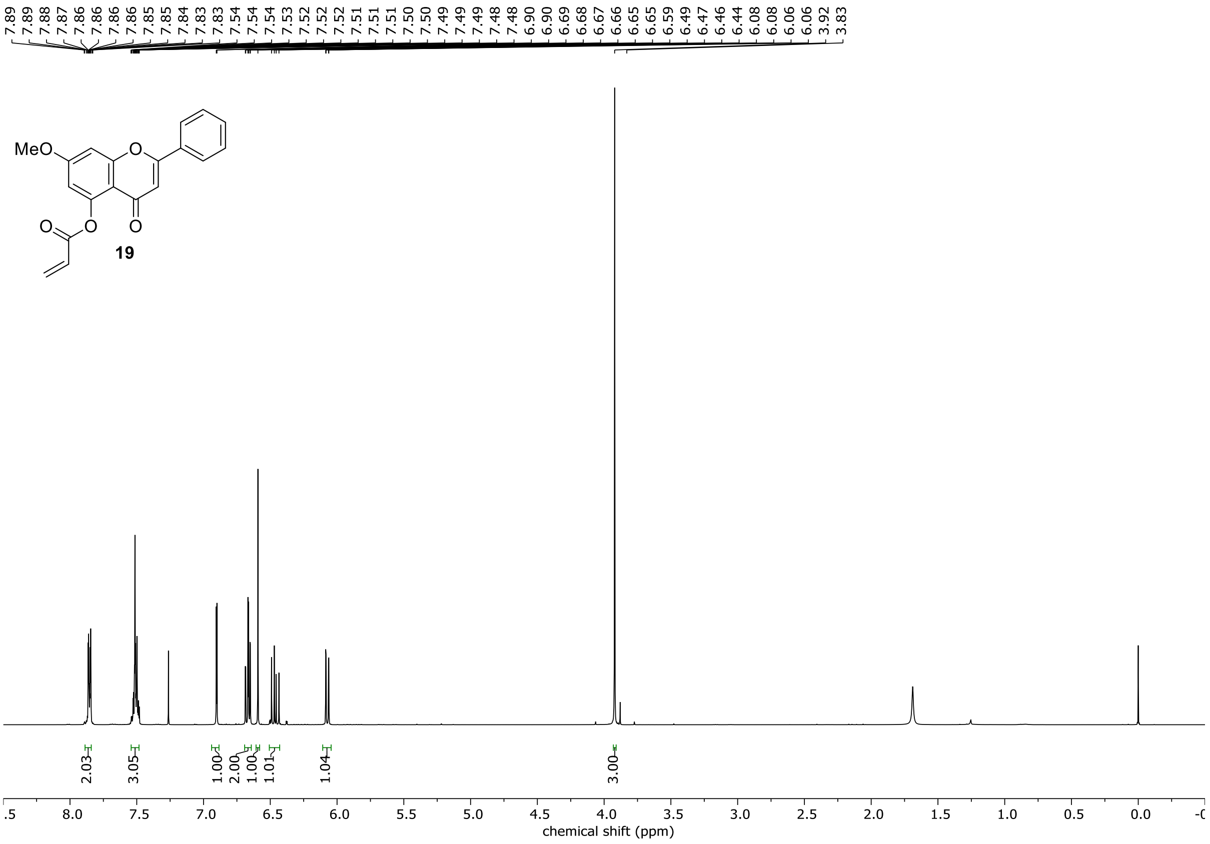

Figure S21. ^{1}H-NMR spectrum (500 MHz, $CDCl_3$) of 5-*O*-acryloyltectochrysin (**19**).

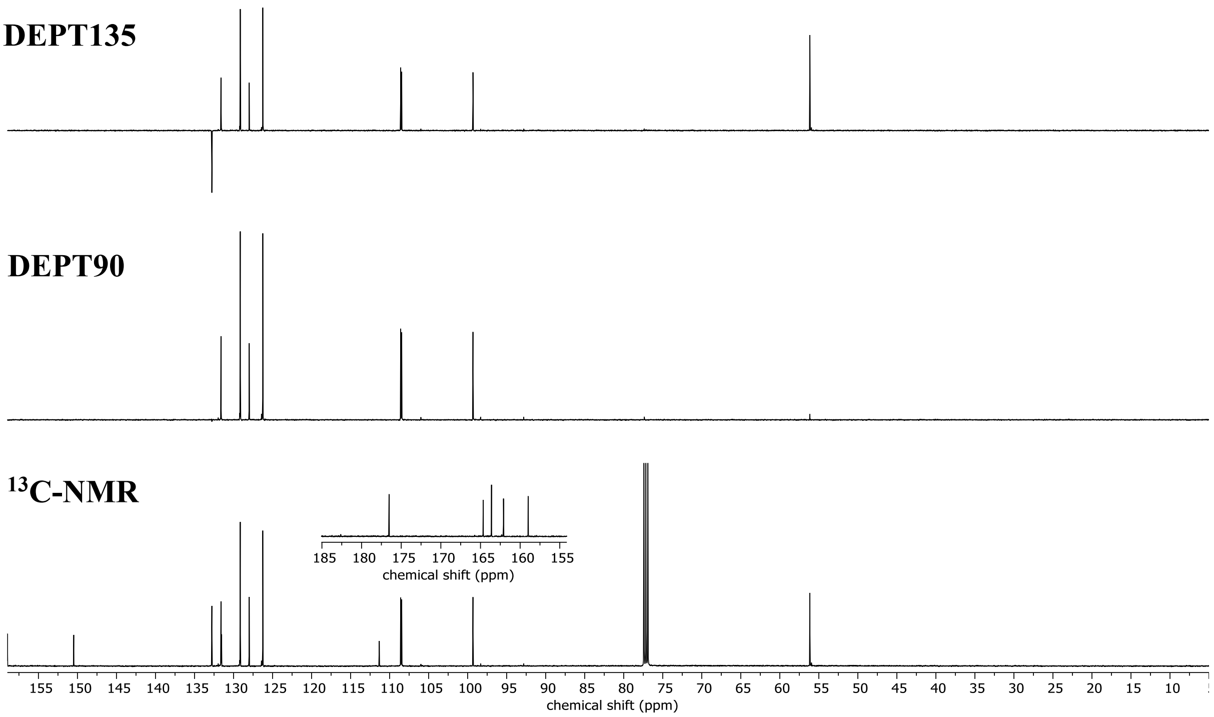

Figure S22. ^{13}C-NMR, DEPT90, and DEPT135 spectra (125 MHz, $CDCl_3$) of 5-*O*-acryloyltectochrysin (**19**).

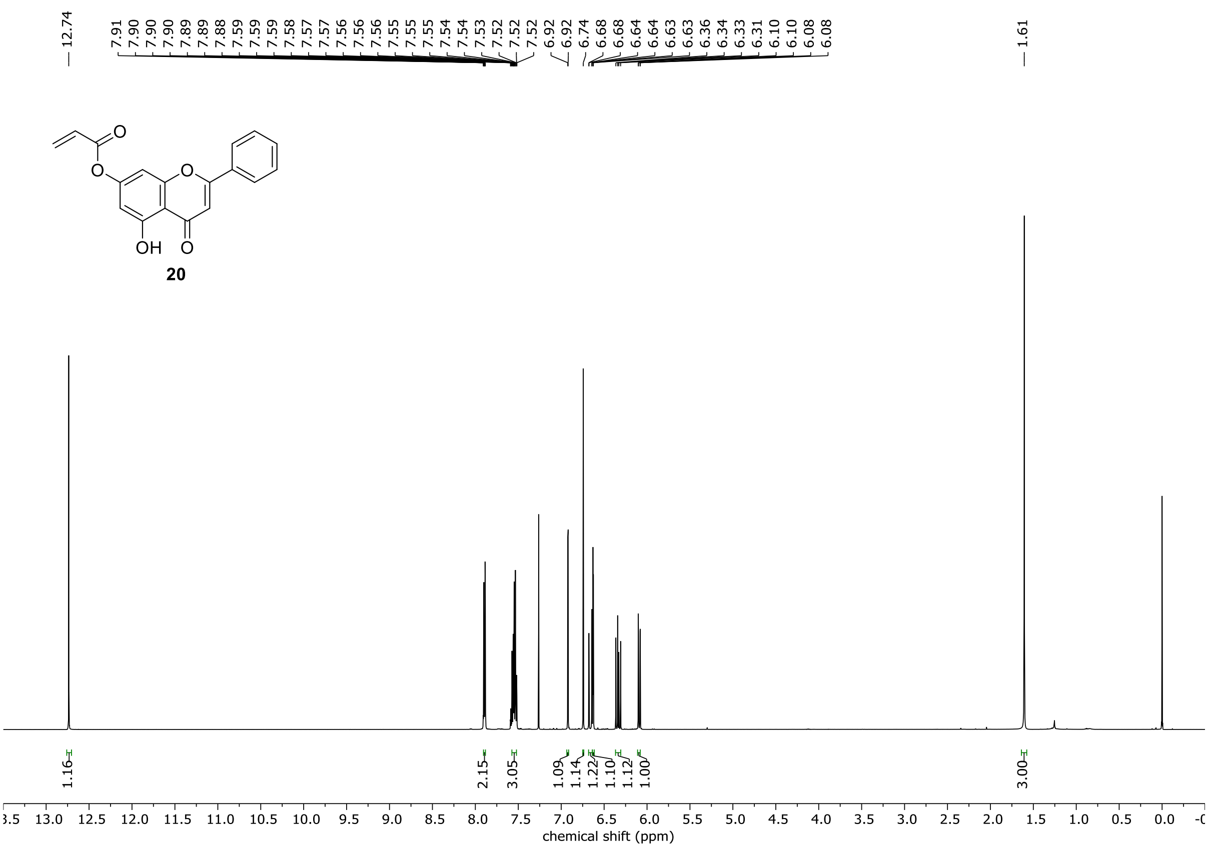

Figure S23. ^{1}H-NMR spectrum (500 MHz, $CDCl_3$) of 7-*O*-acryloylchrysin (**20**).

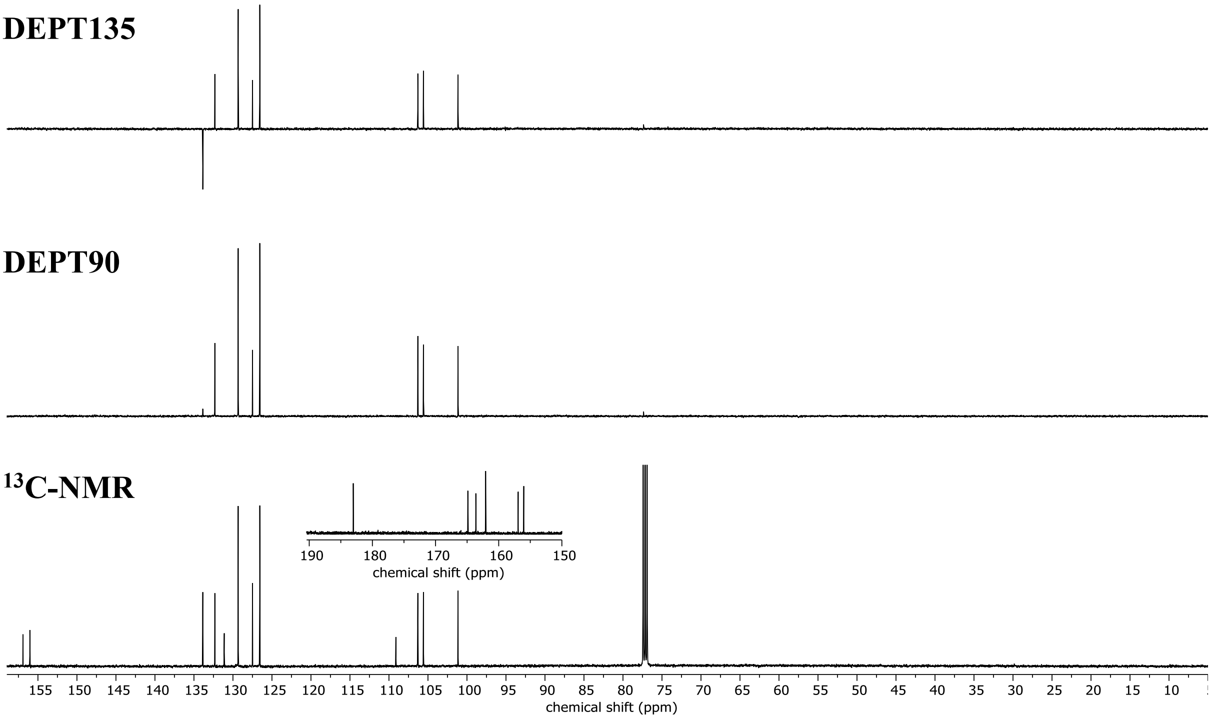

Figure S24. ^{13}C-NMR, DEPT90, and DEPT135 spectra (125 MHz, $CDCl_3$) of 7-*O*-acryloylchrysin (**20**).

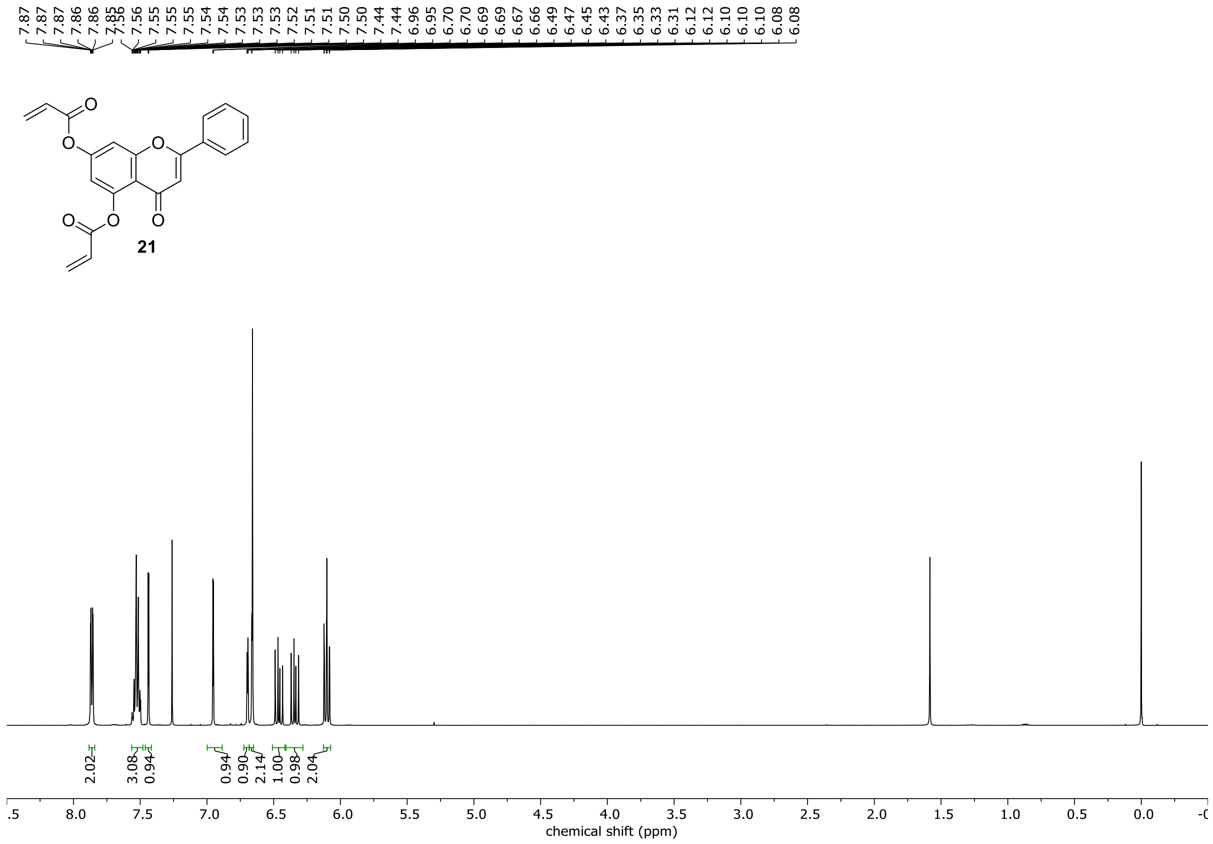

Figure S25. ^{1}H-NMR spectrum (500 MHz, $CDCl_3$) of 5,7-*O*-diacryloylchrysin (**21**).

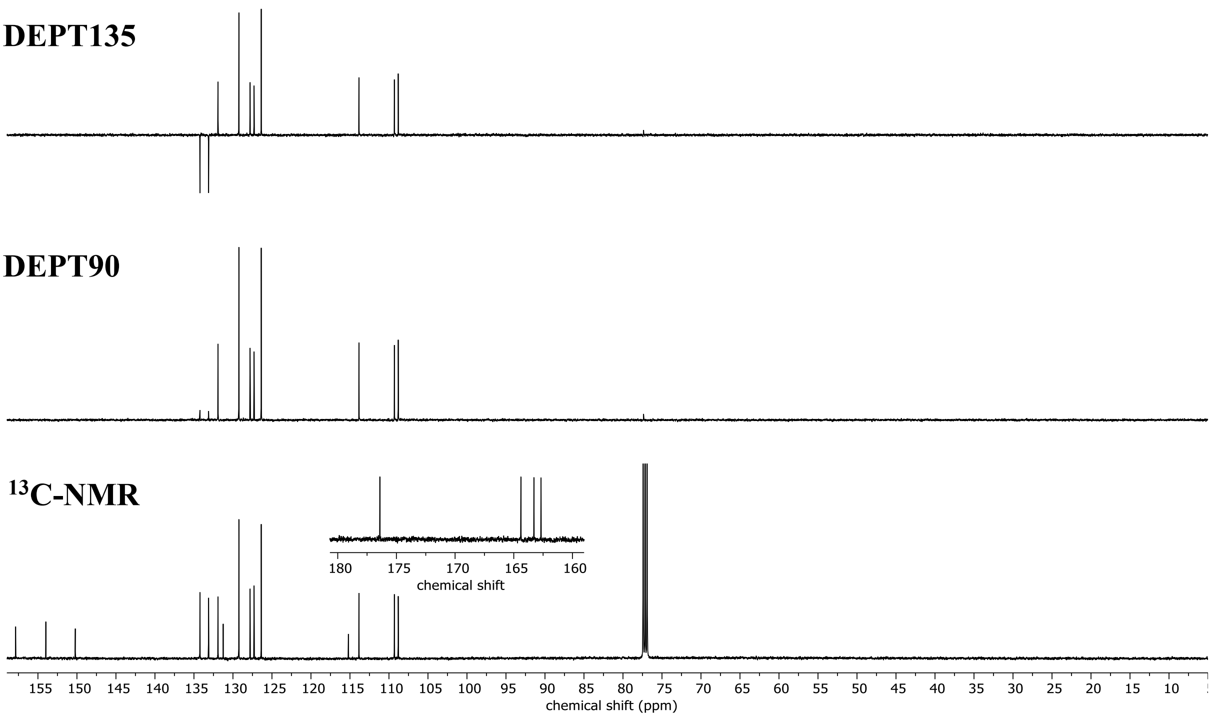

Figure S26. ^{13}C-NMR, DEPT90, and DEPT135 spectra (125 MHz, $CDCl_3$) of 5,7-*O*-diacryloylchrysin (**21**).

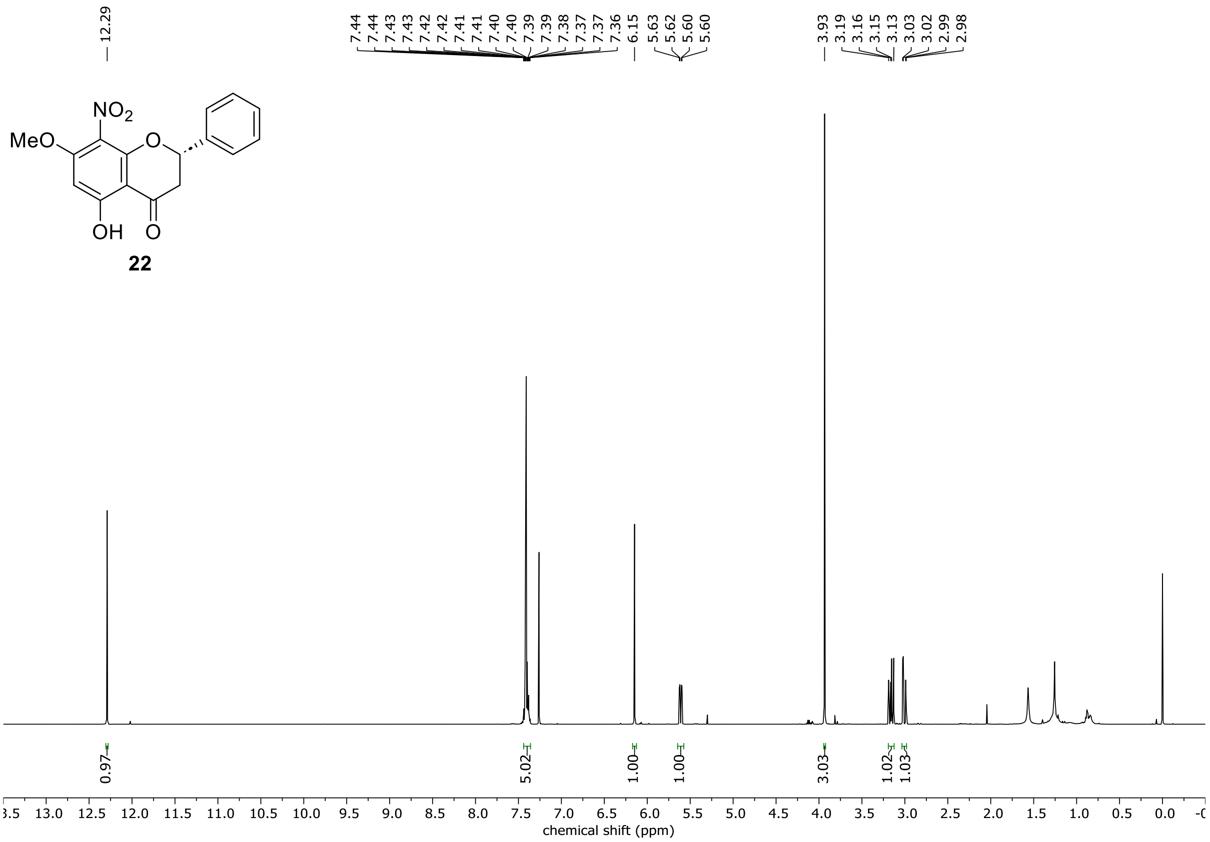

Figure S27. ^{1}H-NMR spectrum (500 MHz, $CDCl_3$) of 8-nitropinostrobin (**22**).

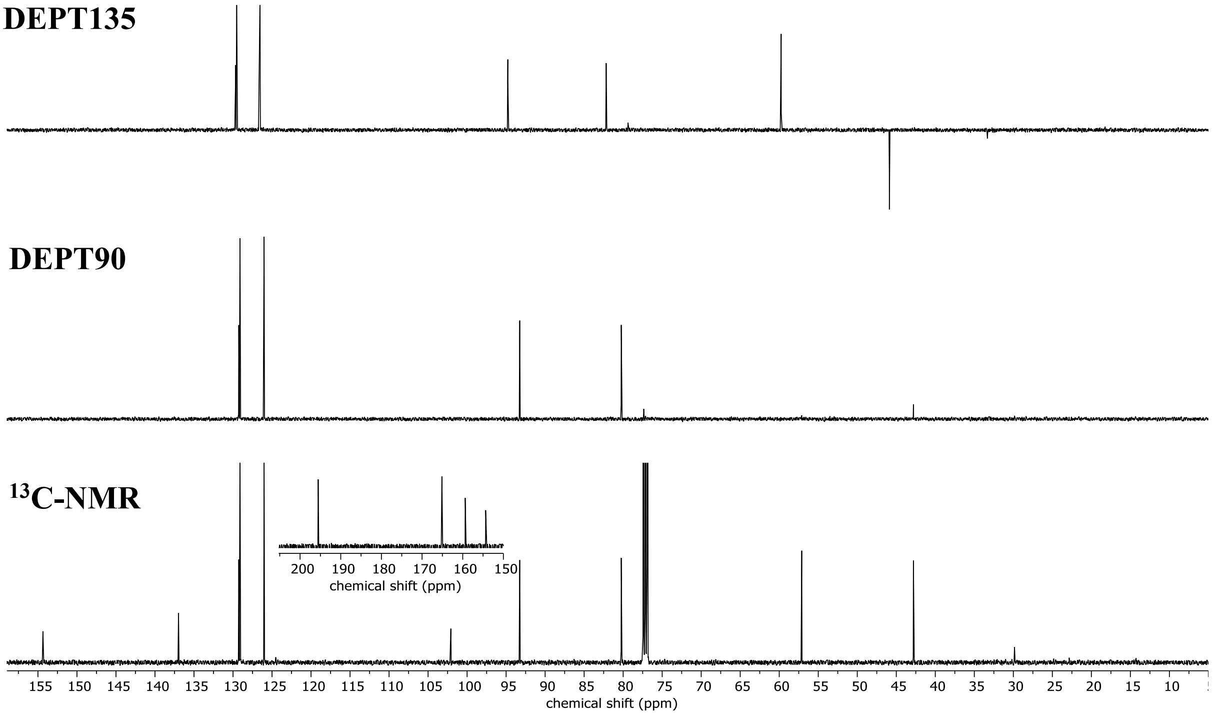

Figure S28. ^{13}C-NMR, DEPT90, and DEPT135 spectra (125 MHz, $CDCl_3$) of 8-nitropinostrobin (**22**).

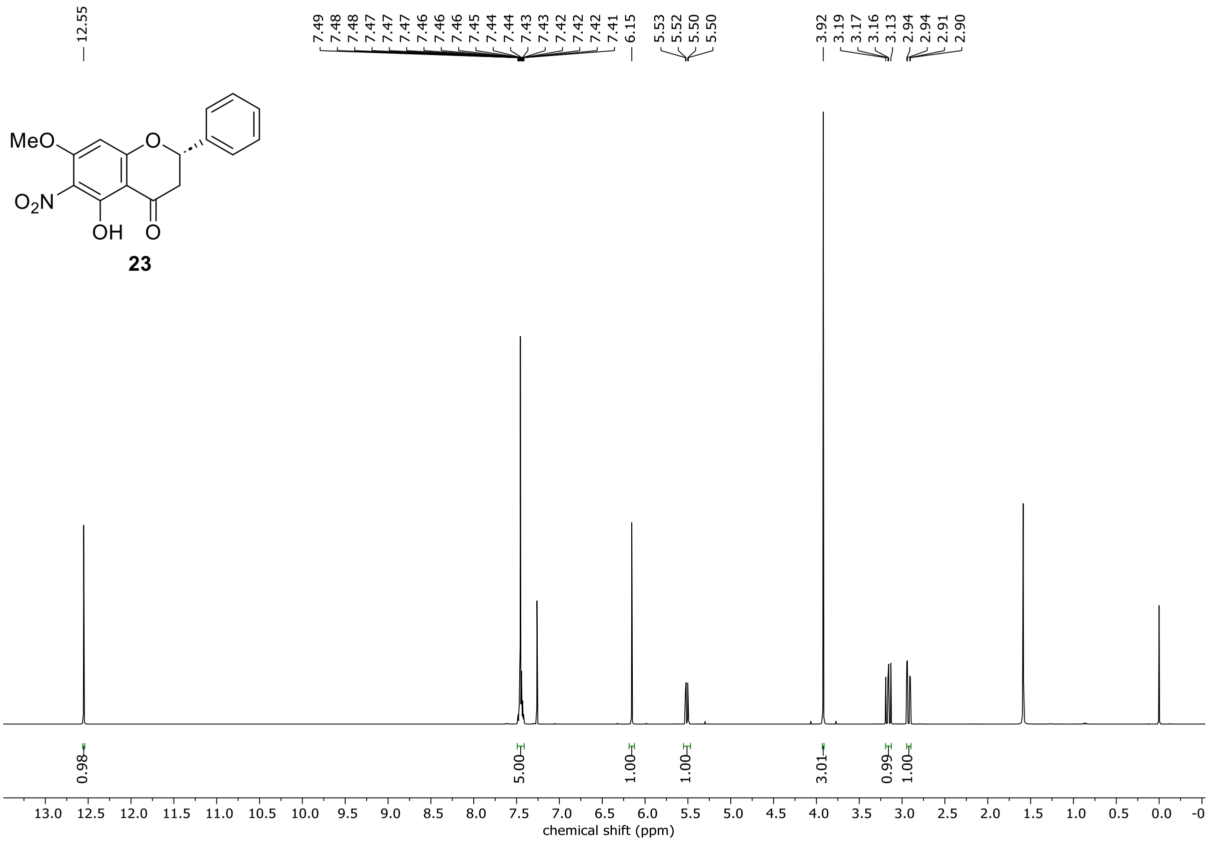

Figure S29. ^{1}H-NMR spectrum (500 MHz, $CDCl_3$) of 6-nitropinostrobin (**23**).

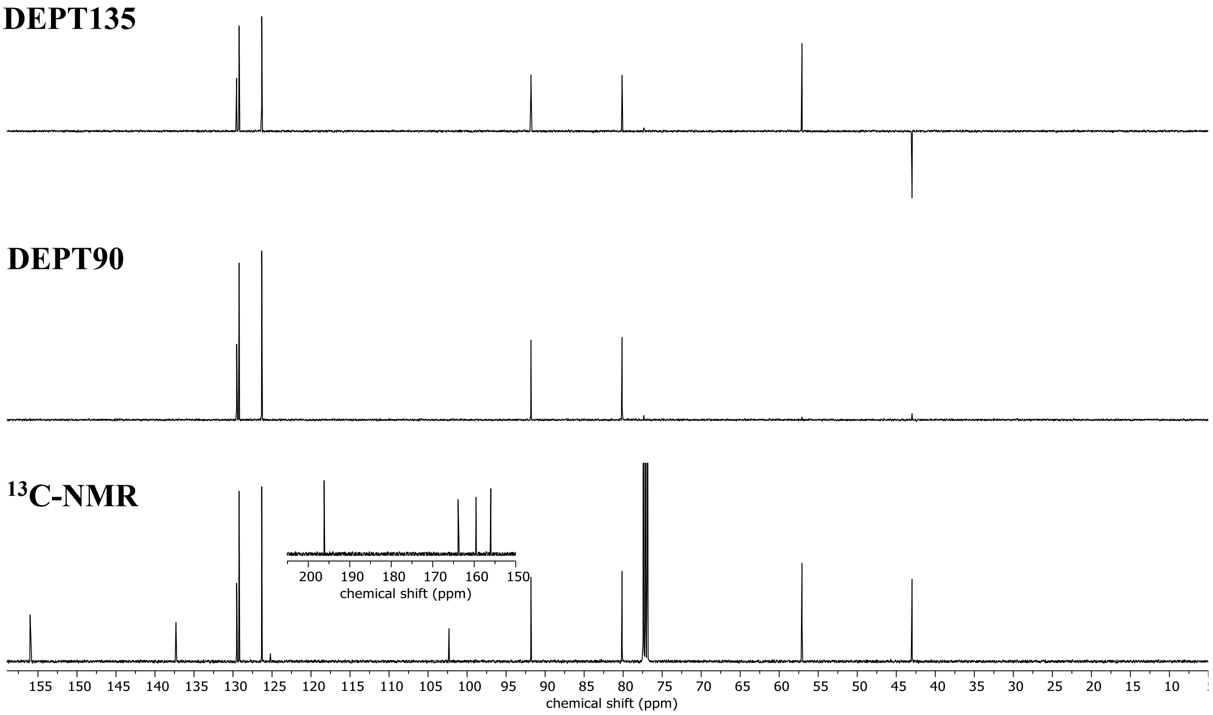

Figure S30. ^{13}C-NMR, DEPT90, and DEPT135 spectra (125 MHz, $CDCl_3$) of 6-nitropinostrobin (**23**).

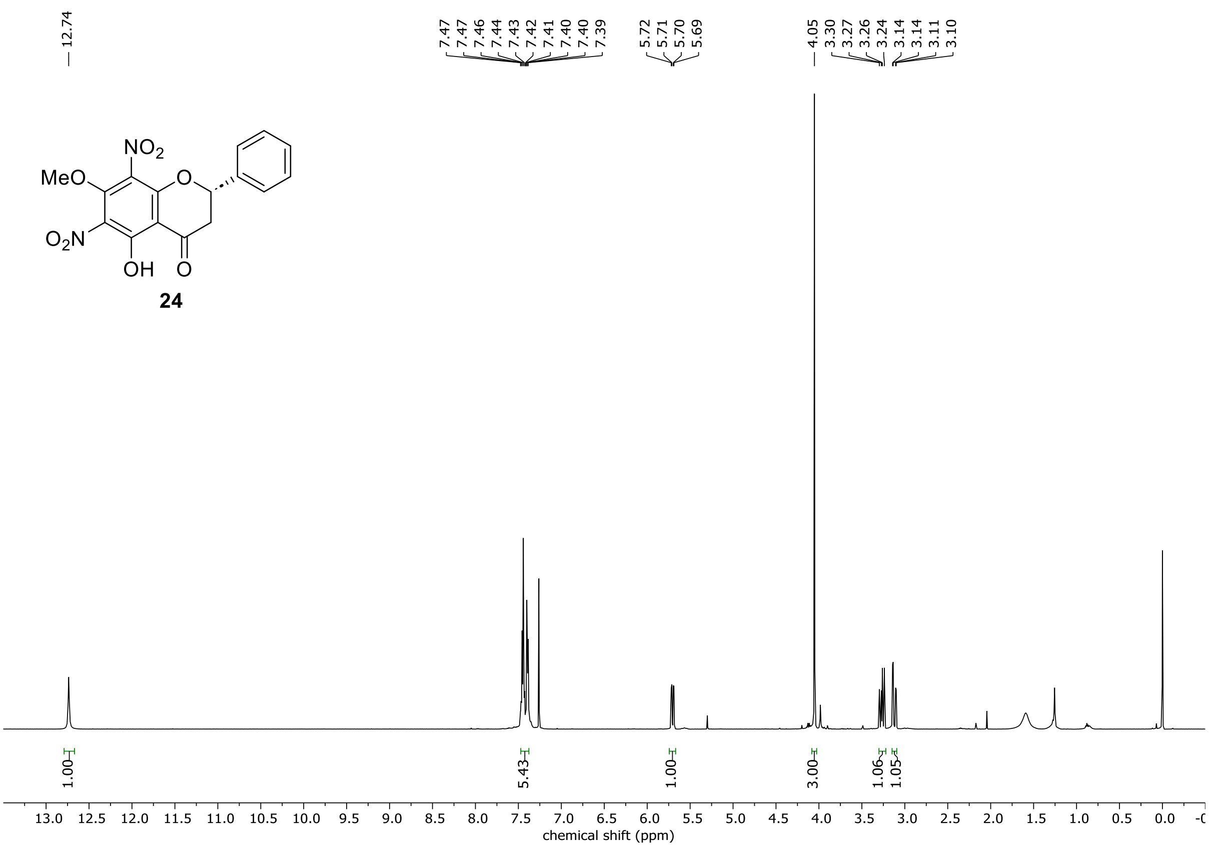

Figure S31. ^{1}H-NMR spectrum (500 MHz, $CDCl_3$) of 6,8-dinitropinostrobin (**24**).

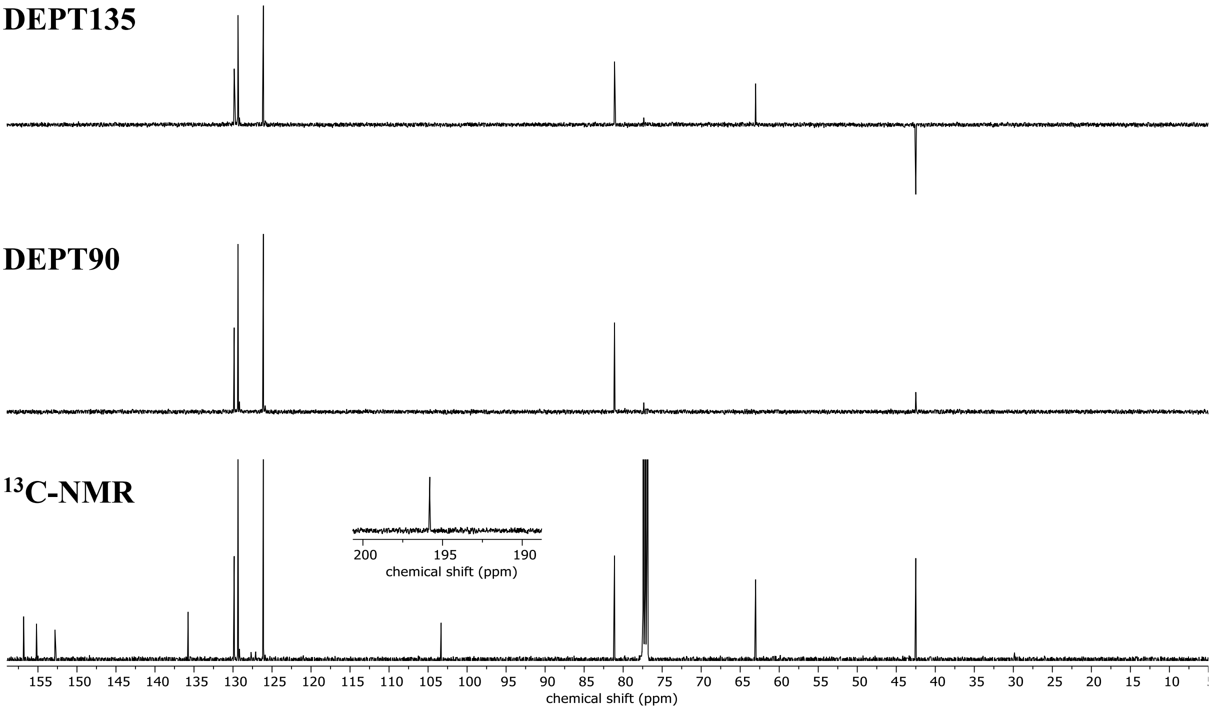

Figure S32. ^{13}C-NMR, DEPT90, and DEPT135 spectra (125 MHz, $CDCl_3$) of 6,8-dinitropinostrobin (**24**).

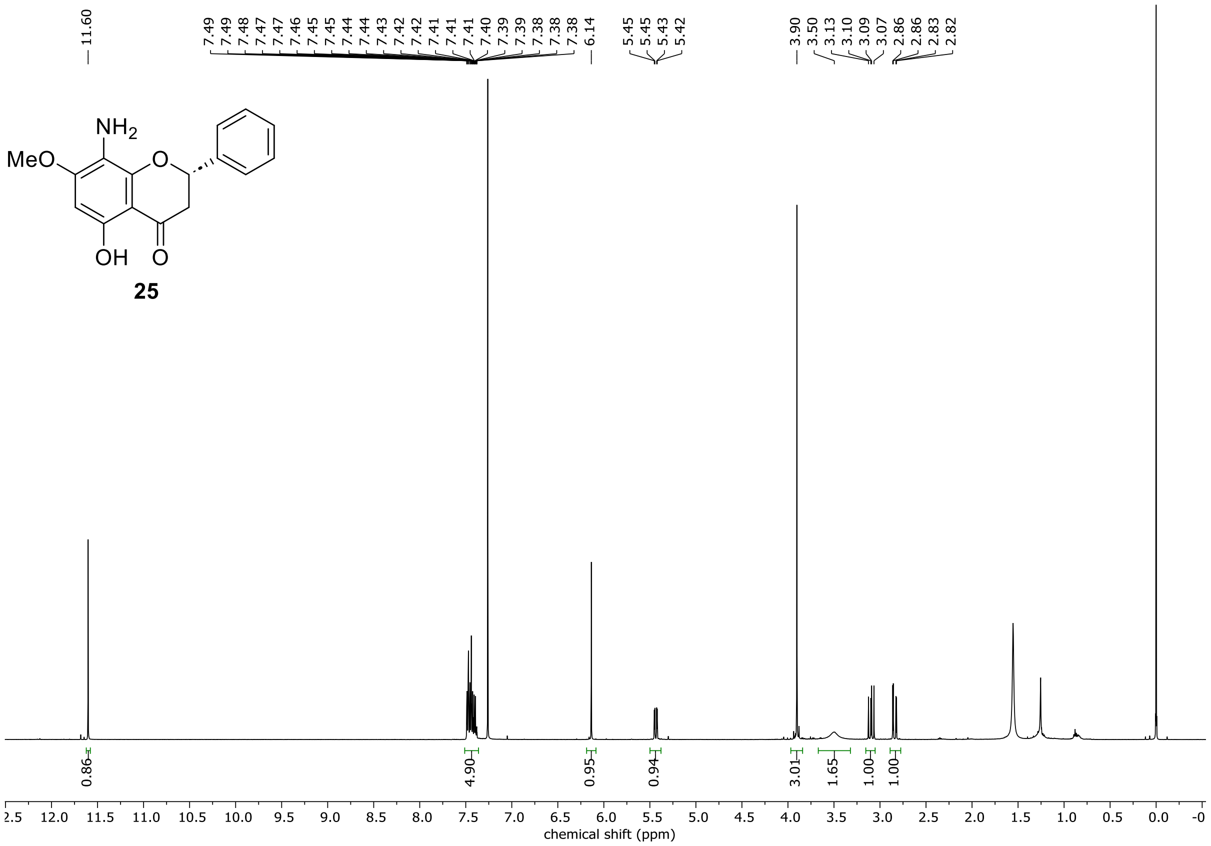

Figure S33. ^{1}H-NMR spectrum (500 MHz, $CDCl_3$) of 8-aminopinostrobin (**25**).

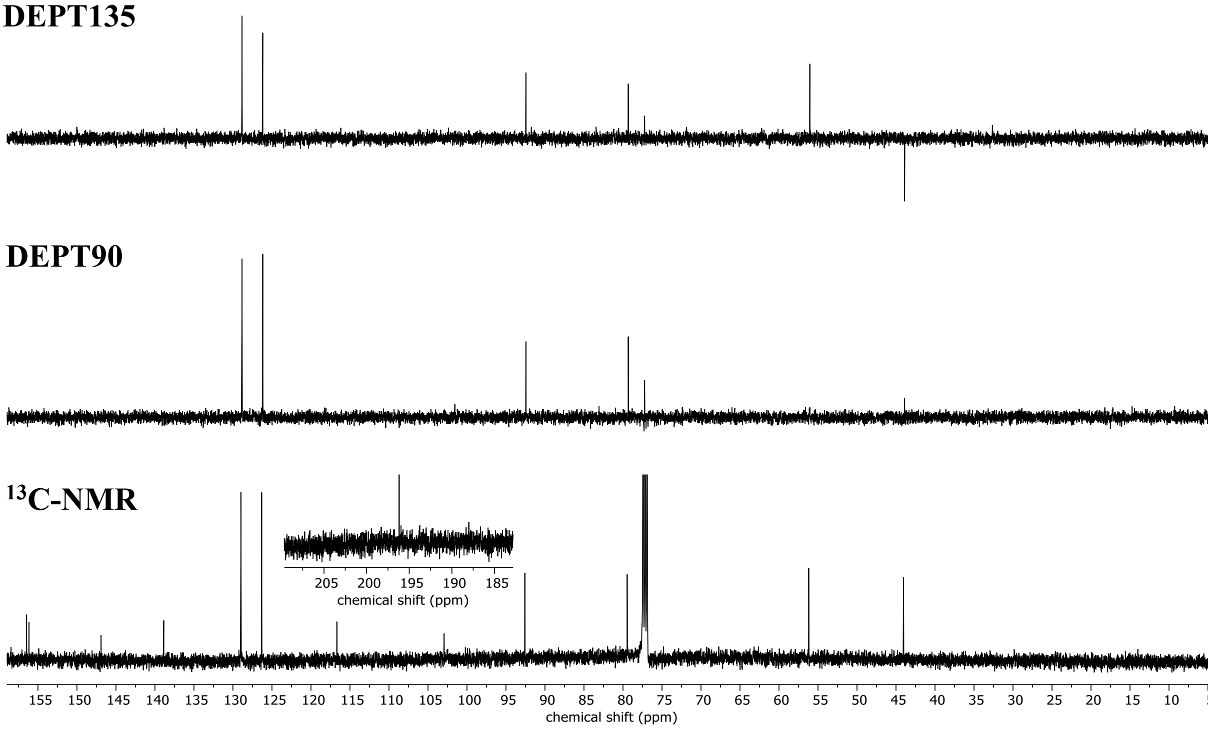

Figure S34. ^{13}C-NMR, DEPT90, and DEPT135 spectra (125 MHz, $CDCl_3$) of 8-aminopinostrobin (**25**).

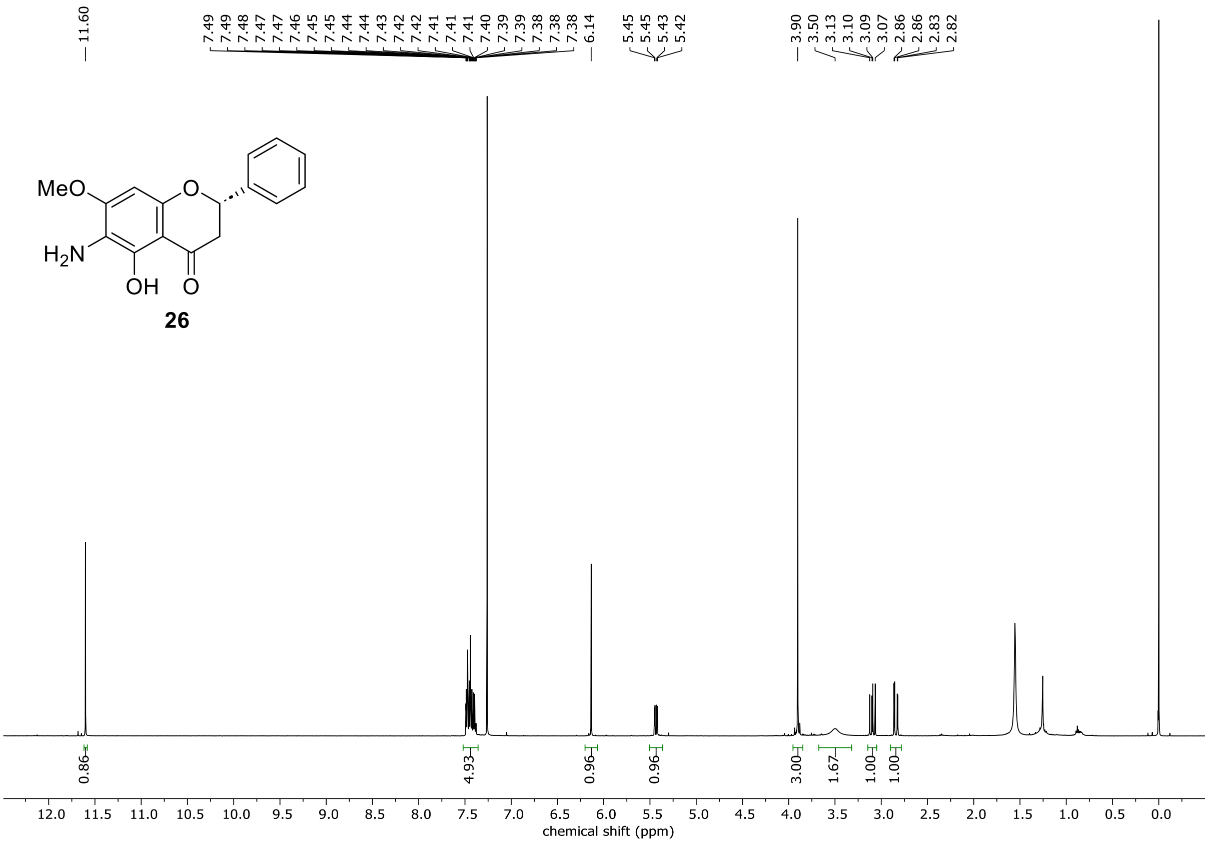

Figure S35. ^{1}H-NMR spectrum (500 MHz, $CDCl_3$) of 6-aminopinostrobin (**26**).

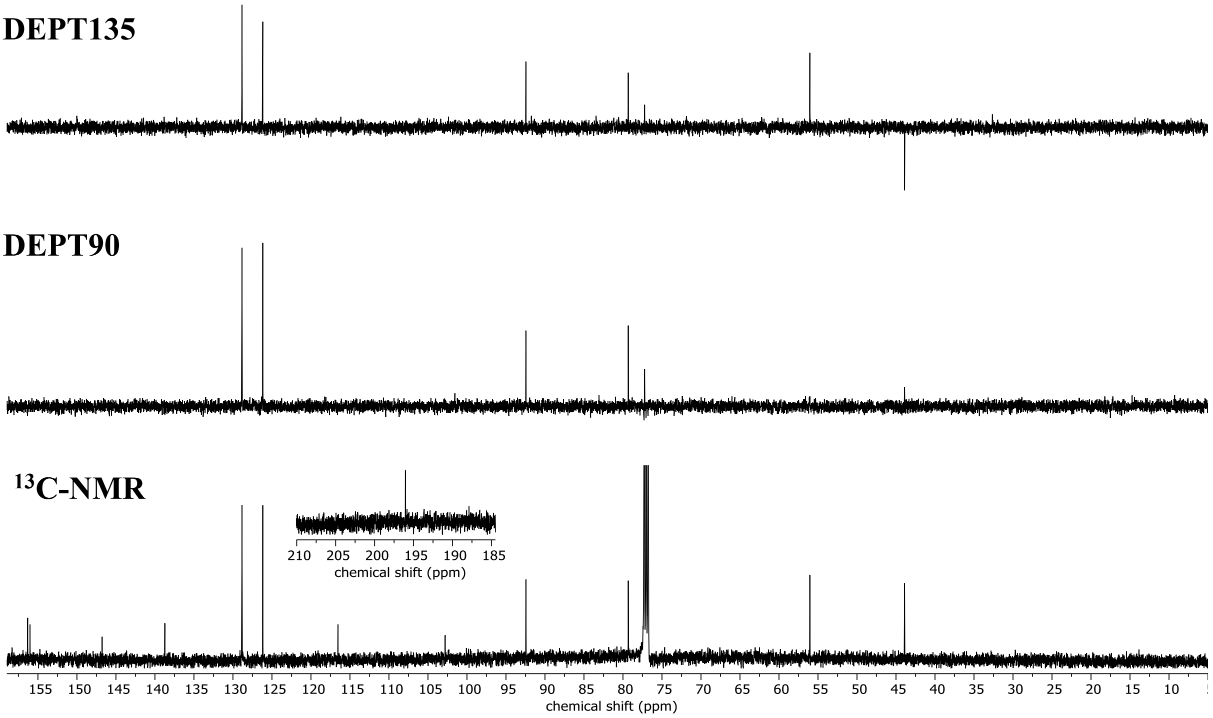

Figure S36. ^{13}C-NMR, DEPT90, and DEPT135 spectra (125 MHz, $CDCl_3$) of 6-aminopinostrobin (**26**).

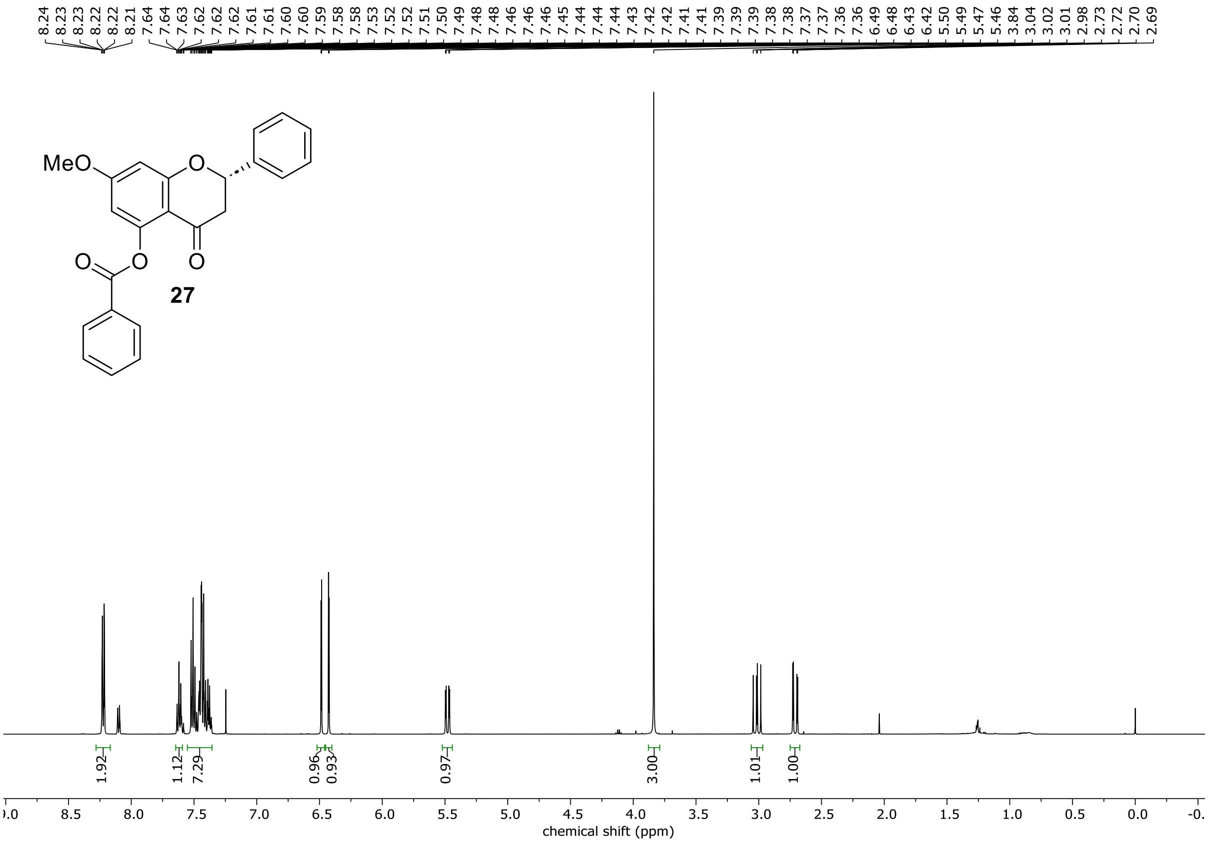

Figure S37. ^{1}H-NMR spectrum (500 MHz, $CDCl_3$) of 5-*O*-benzoylpinostrobin (**27**).

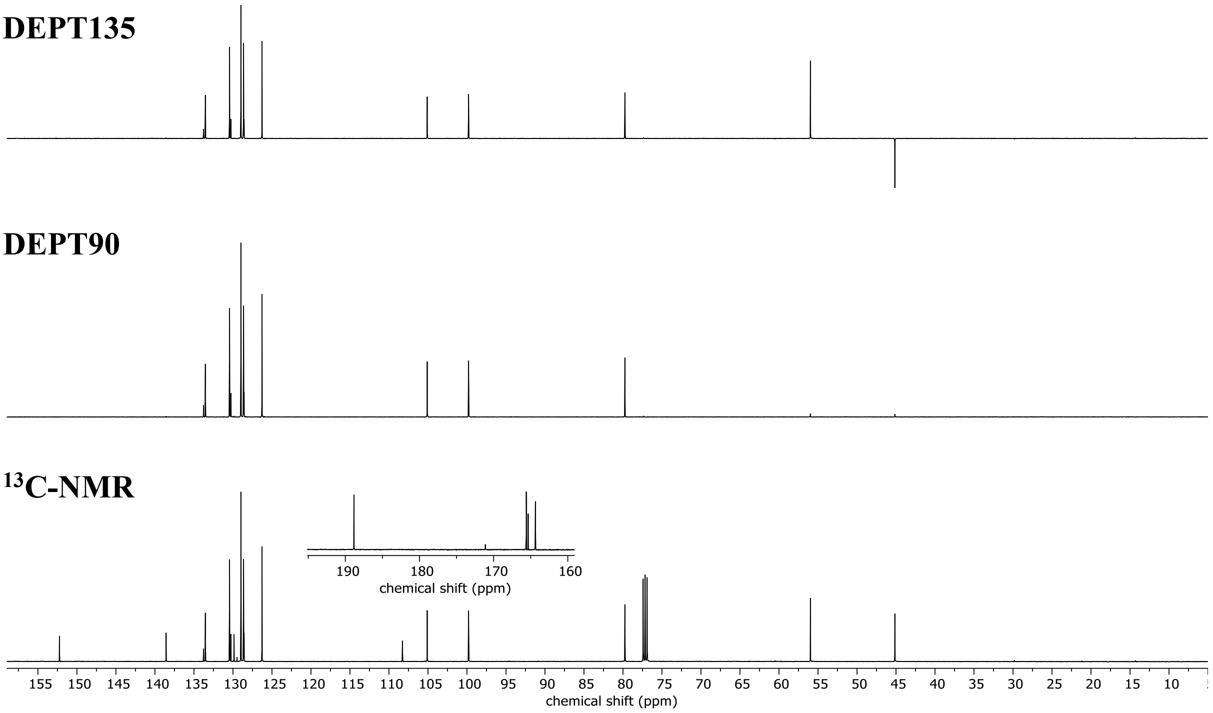

Figure S38. ^{13}C-NMR, DEPT90, and DEPT135 spectra (125 MHz, $CDCl_3$) of 5-*O*-benzoylpinostrobin (**27**).

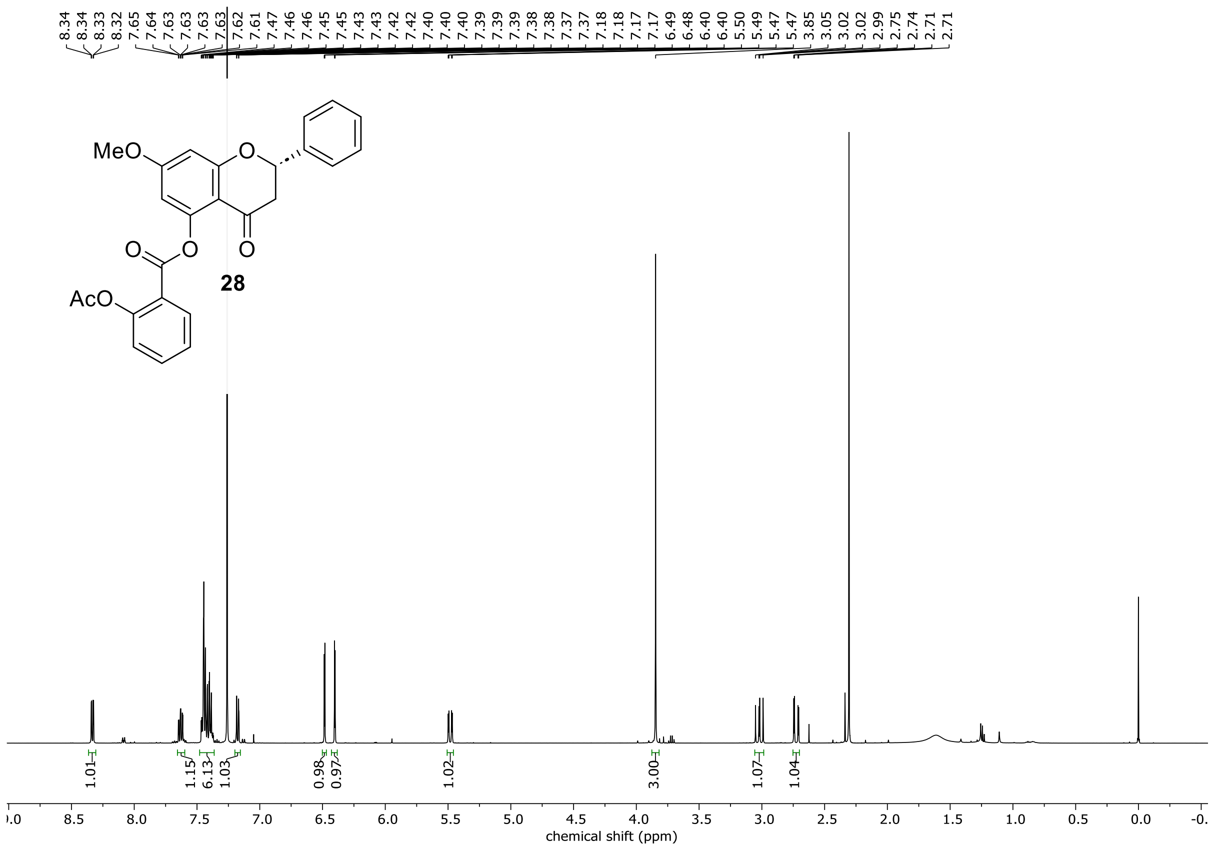

Figure S39. ^{1}H-NMR spectrum (500 MHz, $CDCl_3$) of 5-*O*-(*O*-acetylsalicyloyl)pinostrobin (**28**).

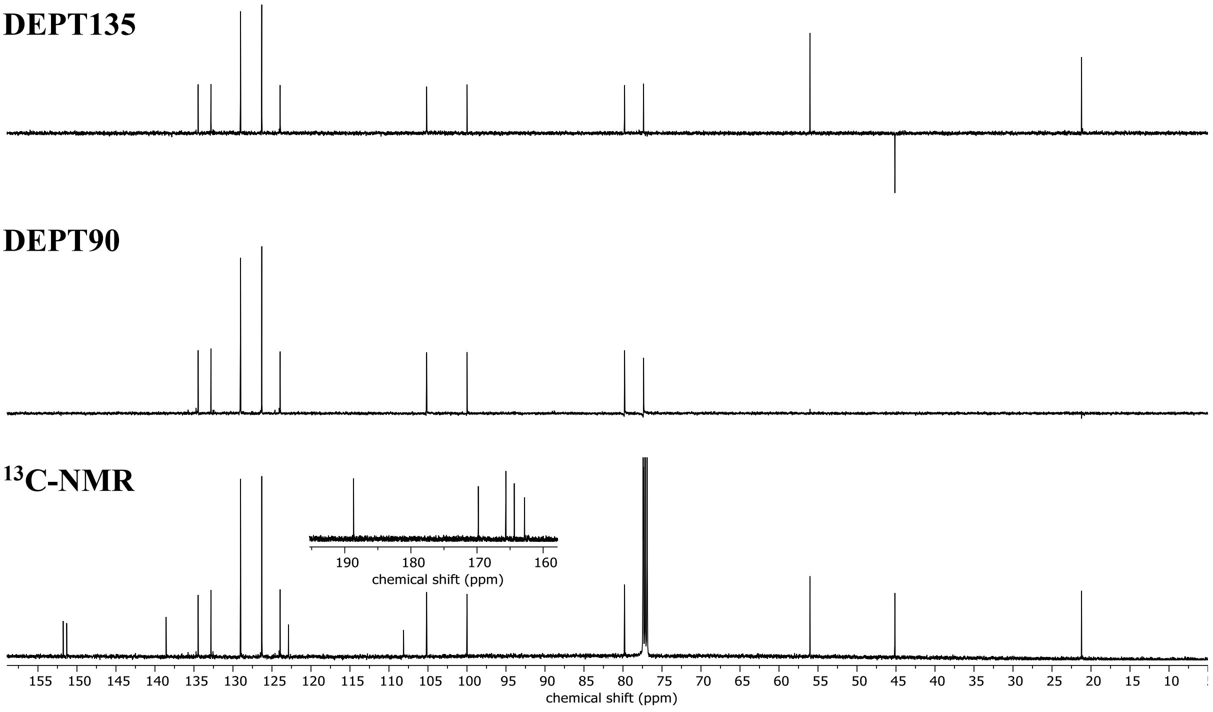

Figure S40. ^{13}C-NMR, DEPT90, and DEPT135 spectra (125 MHz, $CDCl_3$) of 5-*O*-(*O*-acetylsalicyloyl)pinostrobin (**28**).

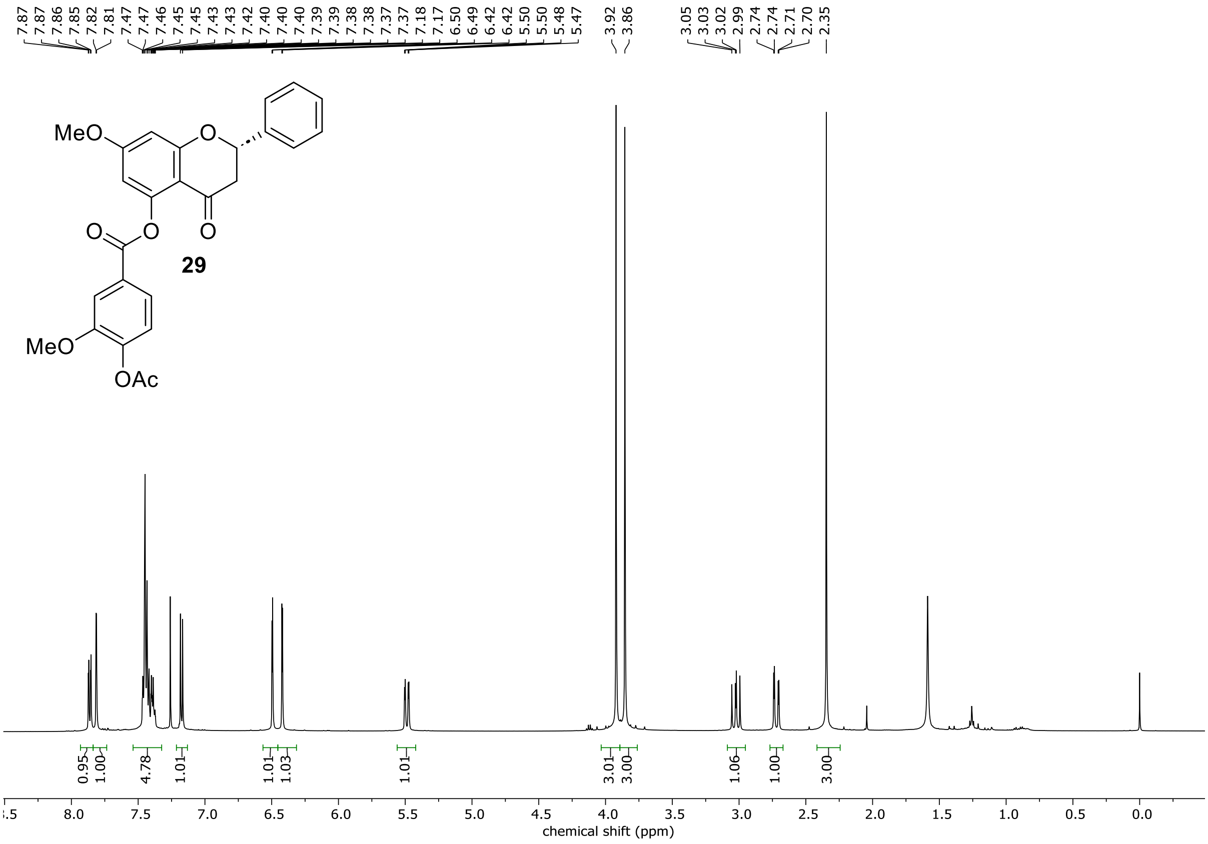

Figure S41. ^{1}H-NMR spectrum (500 MHz, $CDCl_3$) of 5-*O*-(*O*-acetylvanilloyl)pinostrobin (**29**).

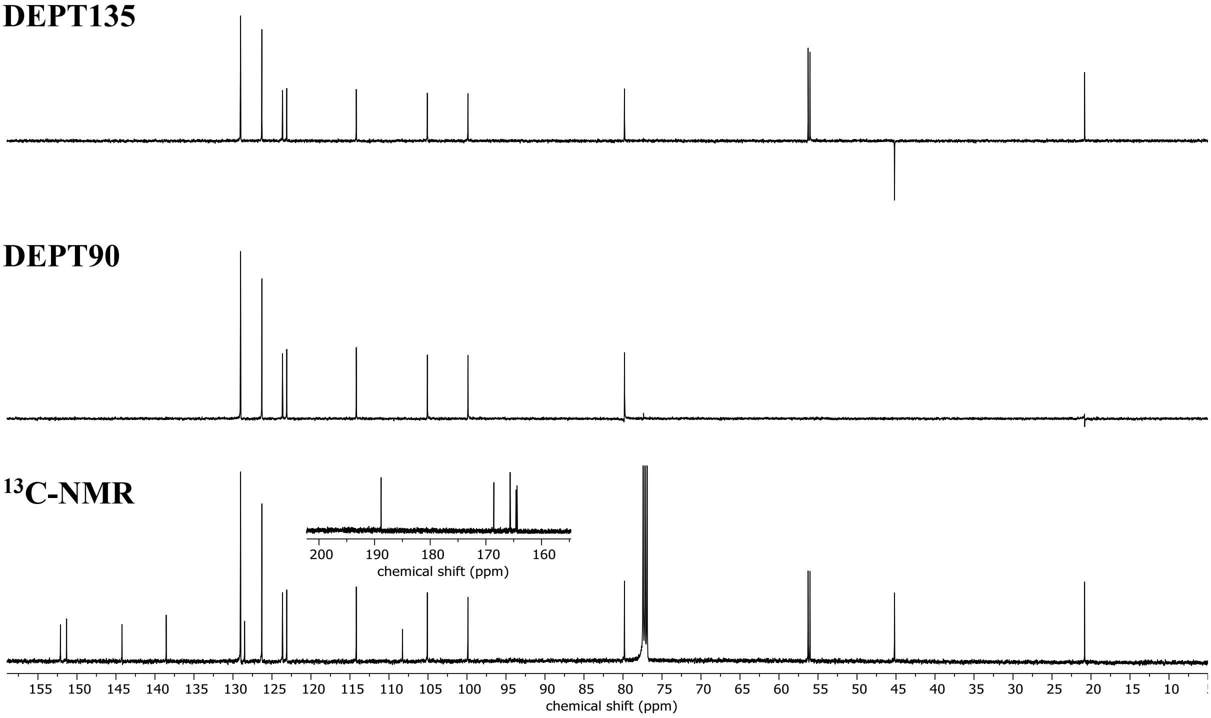

Figure S42. ^{13}C-NMR, DEPT90, and DEPT135 spectra (125 MHz, $CDCl_3$) of 5-*O*-(*O*-acetylvanilloyl)pinostrobin (**29**).

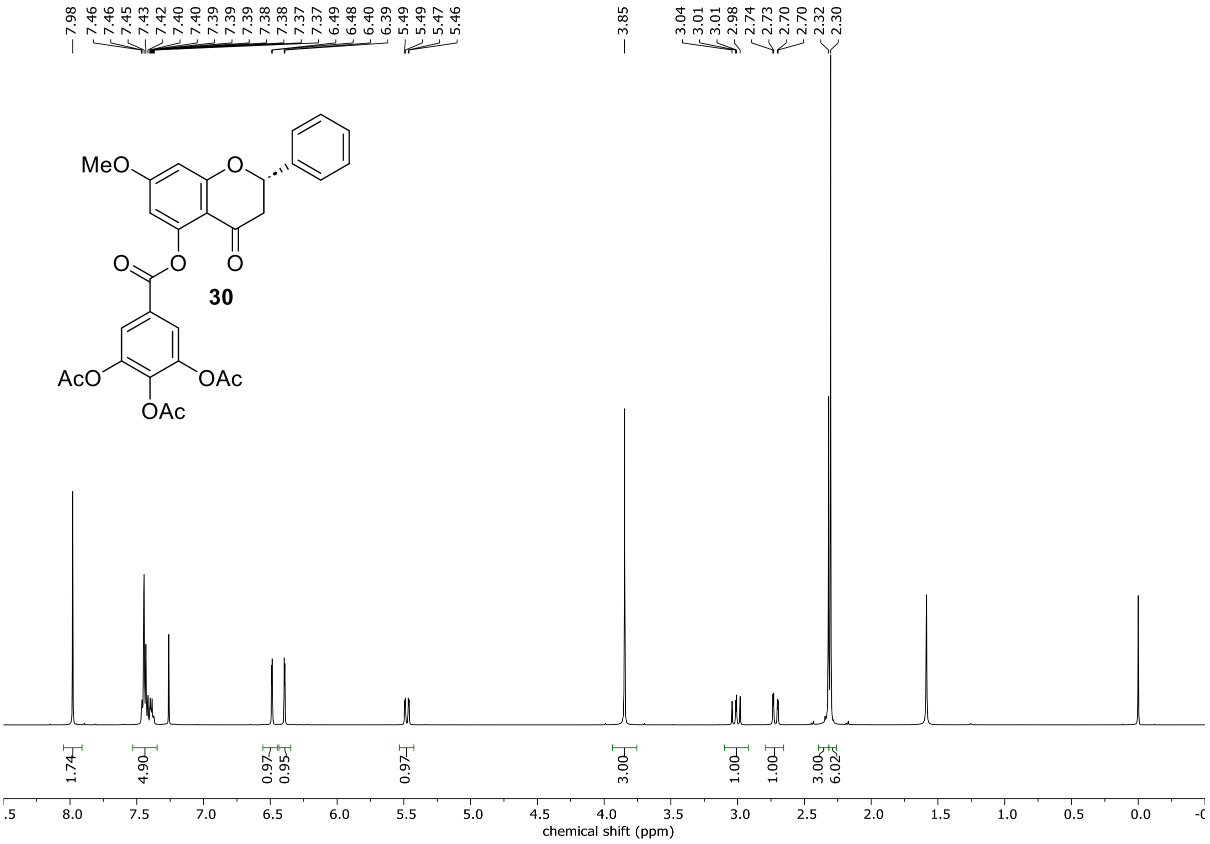

Figure S43. ^{1}H-NMR spectrum (500 MHz, $CDCl_3$) of 5-*O*-(tri-*O*-acetylgalloyl)pinostrobin (**30**).

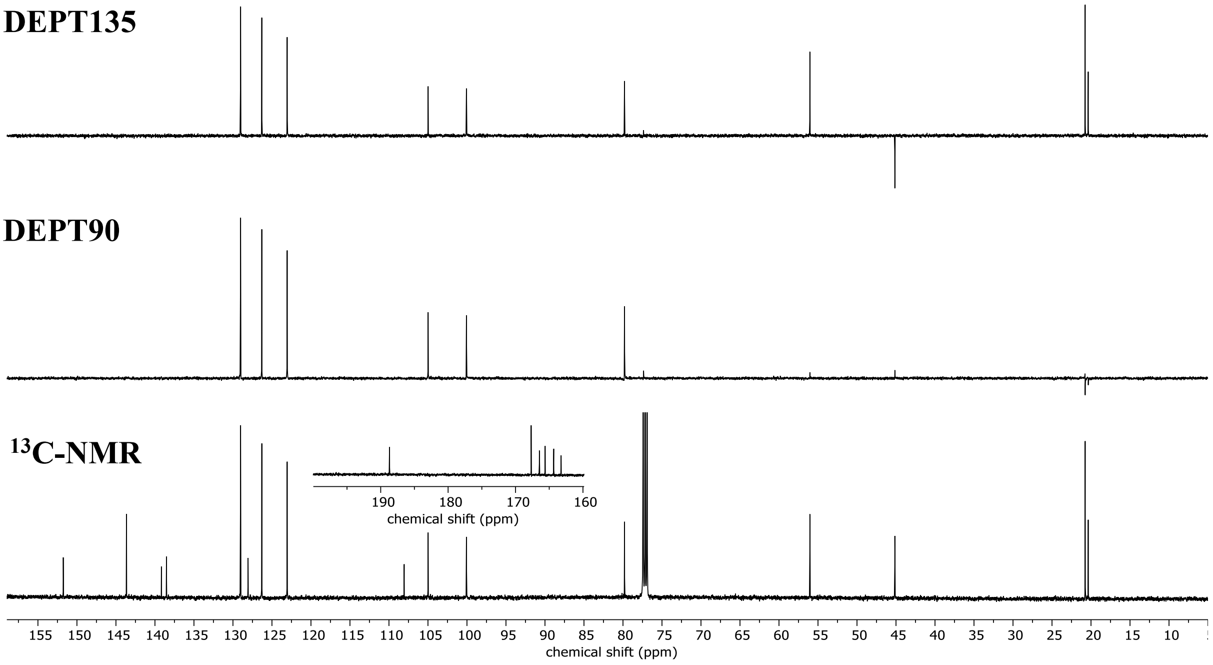

Figure S44. ^{13}C-NMR, DEPT90, and DEPT135 spectra (125 MHz, $CDCl_3$) of 5-*O*-(tri-*O*-acetylgalloyl)pinostrobin (**30**).

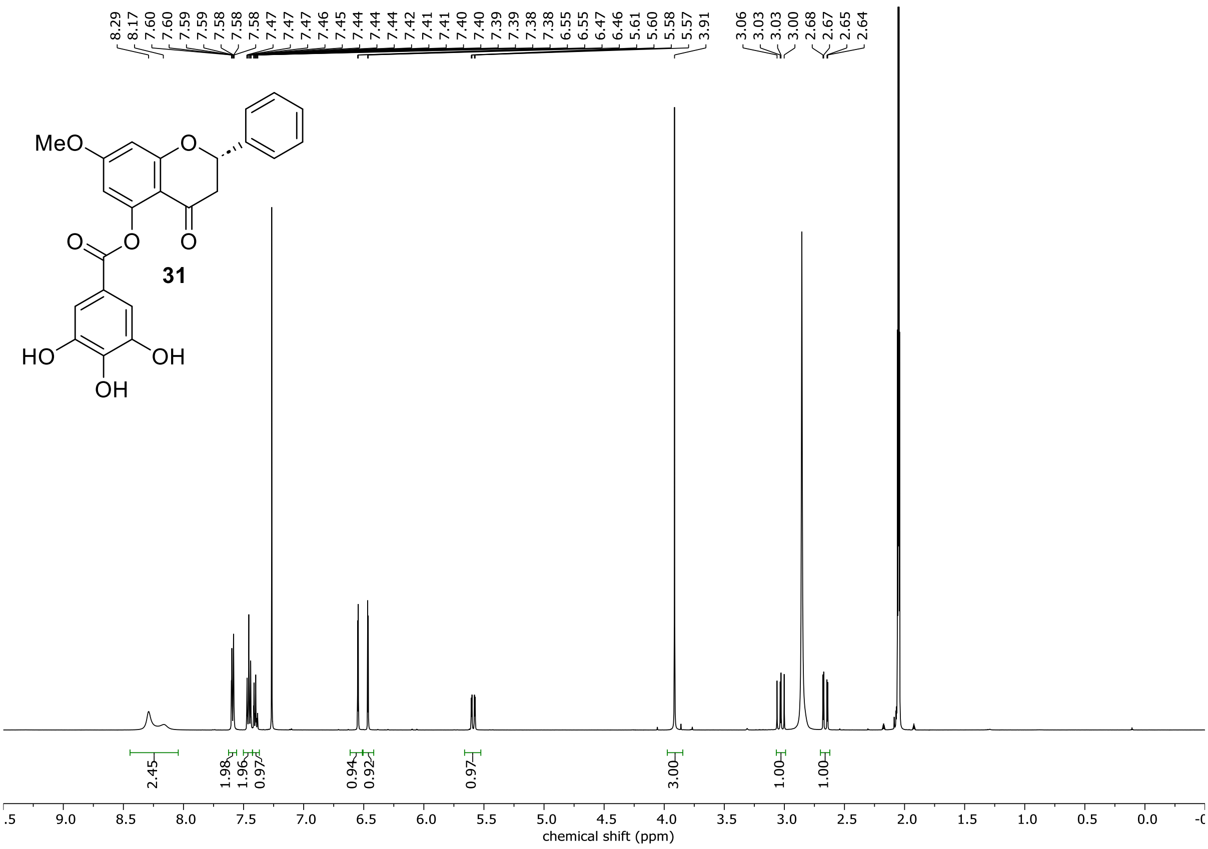

Figure S45. ^{1}H-NMR spectrum (500 MHz, acetone-d_6) of 5-*O*-galloylpinostrobin (**31**).

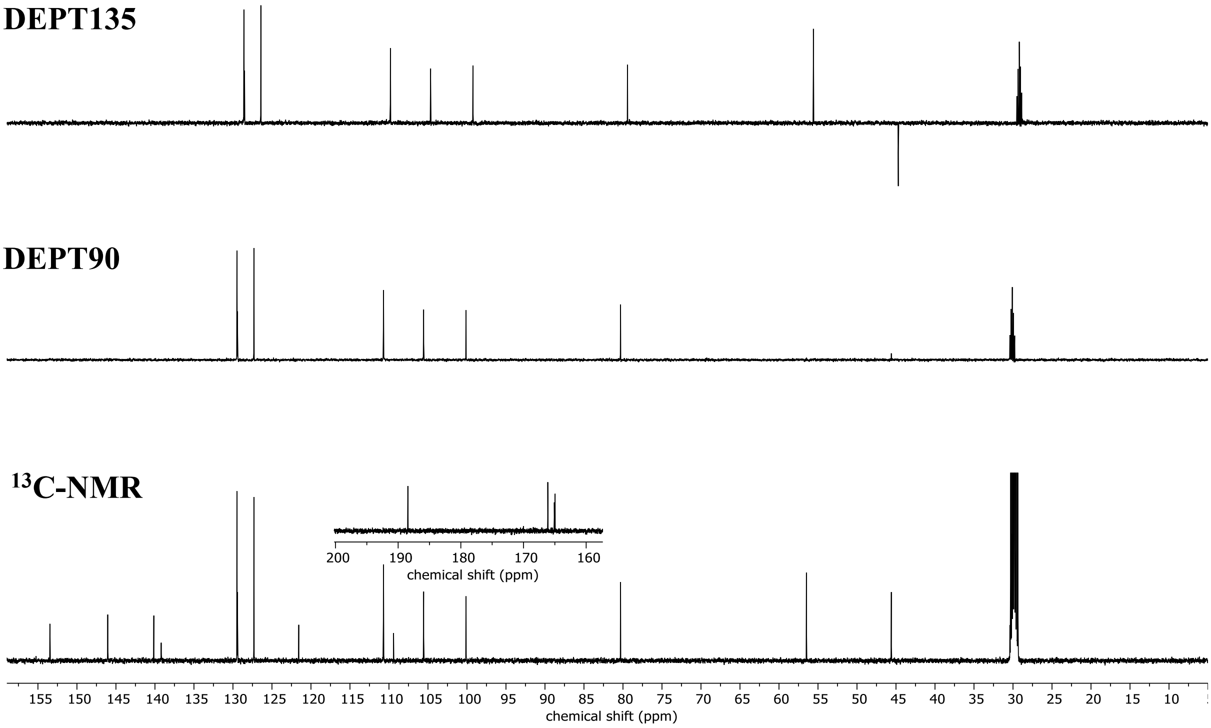

Figure S46. ^{13}C-NMR, DEPT90, and DEPT135 spectra (125 MHz, acetone-d_6) of 5-*O*-galloylpinostrobin (**31**).

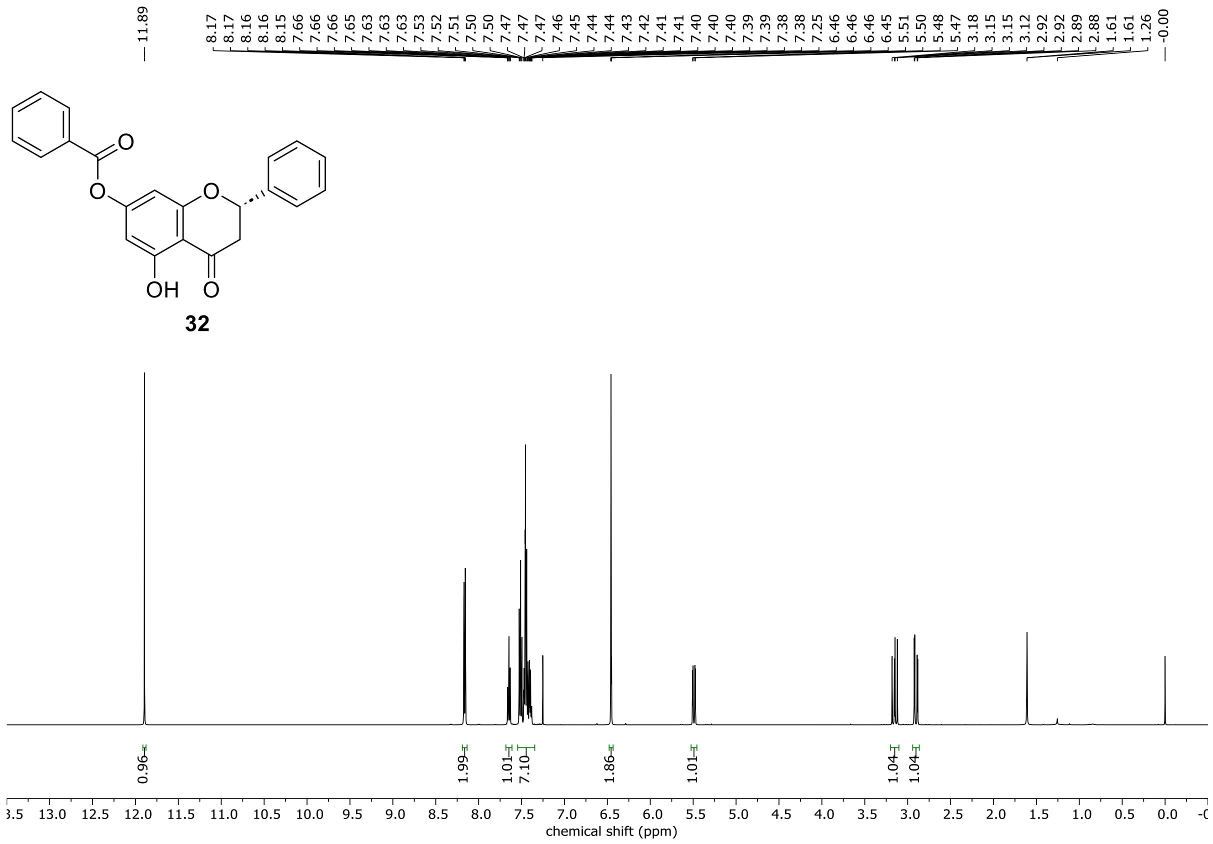

Figure S47. ^{1}H-NMR spectrum (500 MHz, $CDCl_3$) of 7-*O*-benzoylpinocembrin (**32**).

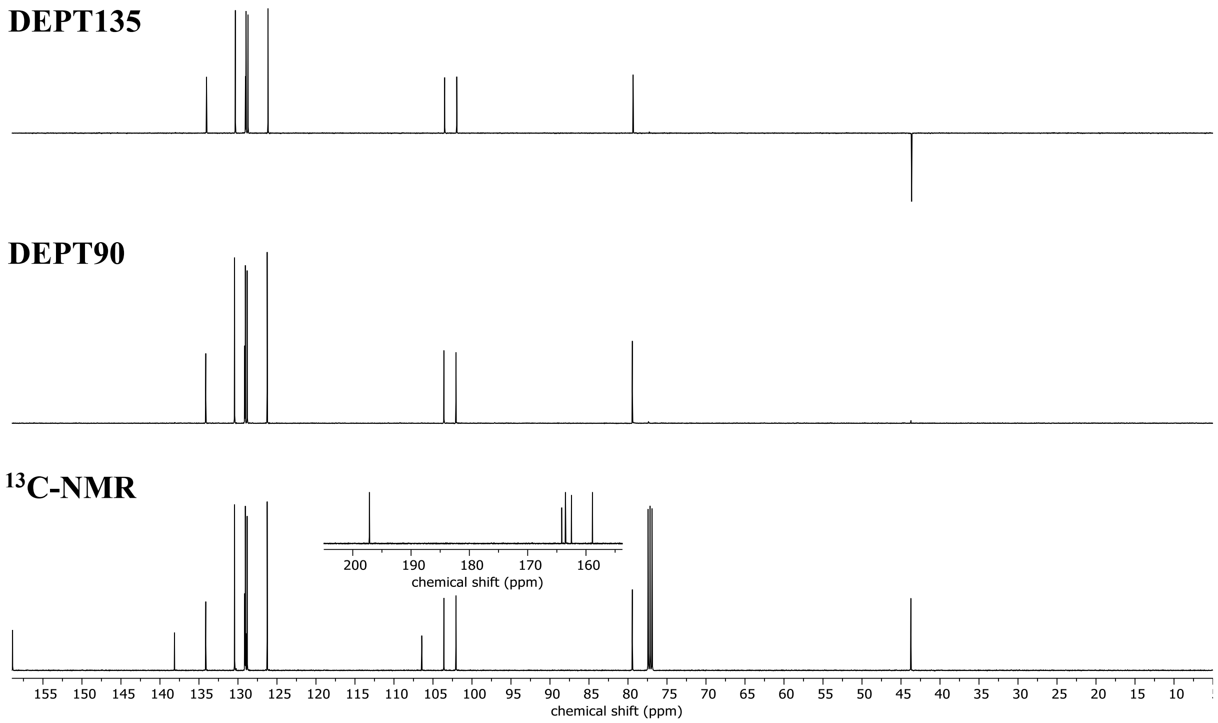

Figure S48. ^{13}C-NMR, DEPT90, and DEPT135 spectra (125 MHz, $CDCl_3$) of 7-*O*-benzoylpinocembrin (**32**).

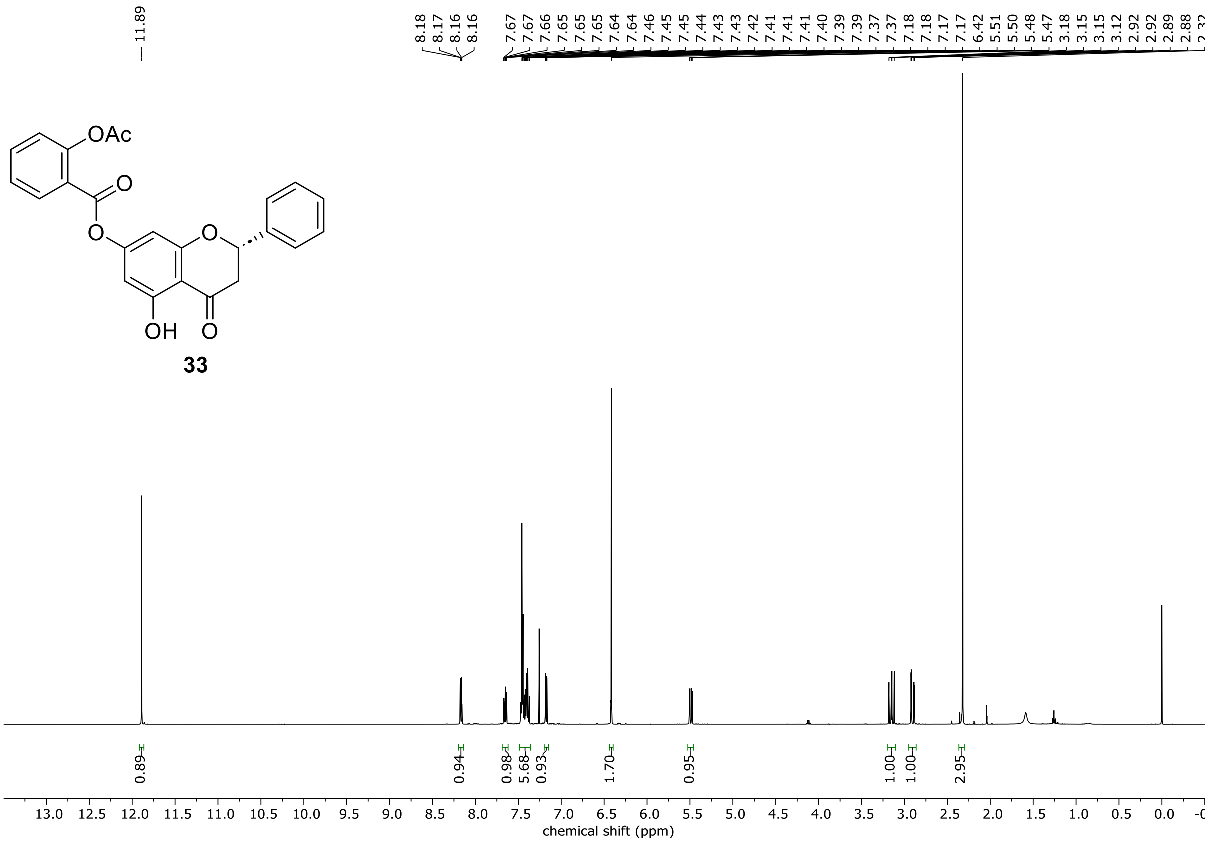

Figure S49. ^{1}H-NMR spectrum (500 MHz, $CDCl_3$) of 7-*O*-(*O*-acetylsalicyloyl)pinocembrin (**33**).

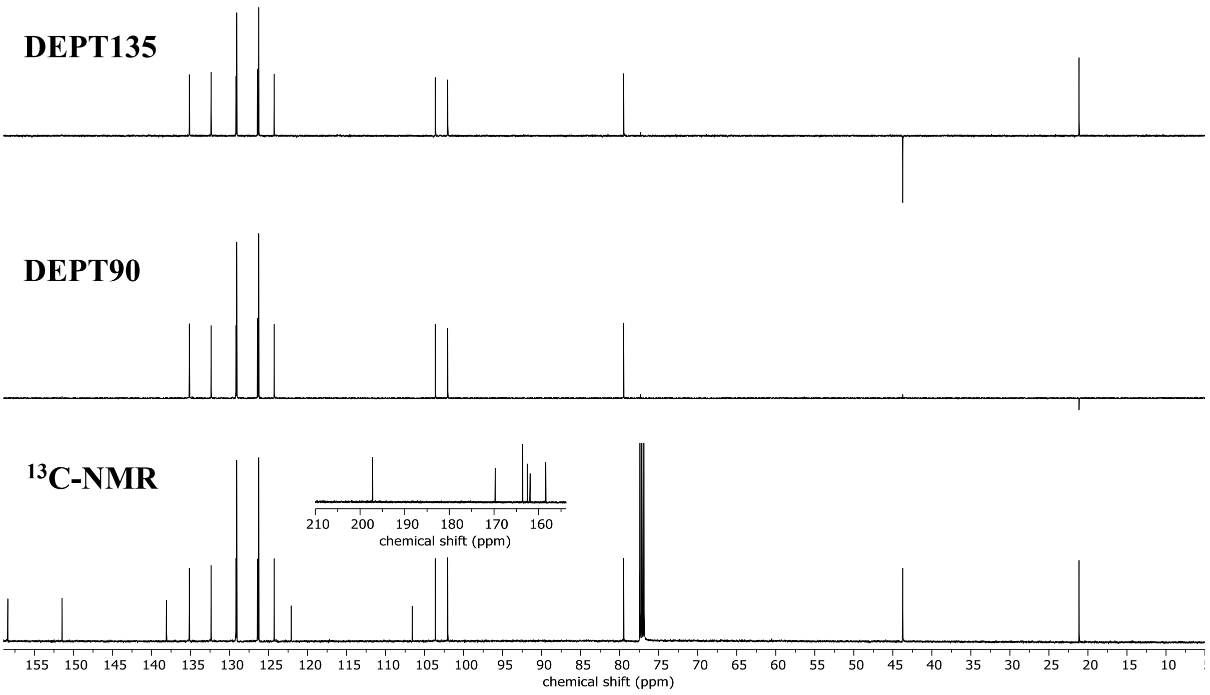

Figure S50. ^{13}C-NMR, DEPT90, and DEPT135 spectra (125 MHz, $CDCl_3$) of 7-*O*-(*O*-acetylsalicyloyl)pinocembrin (**33**).

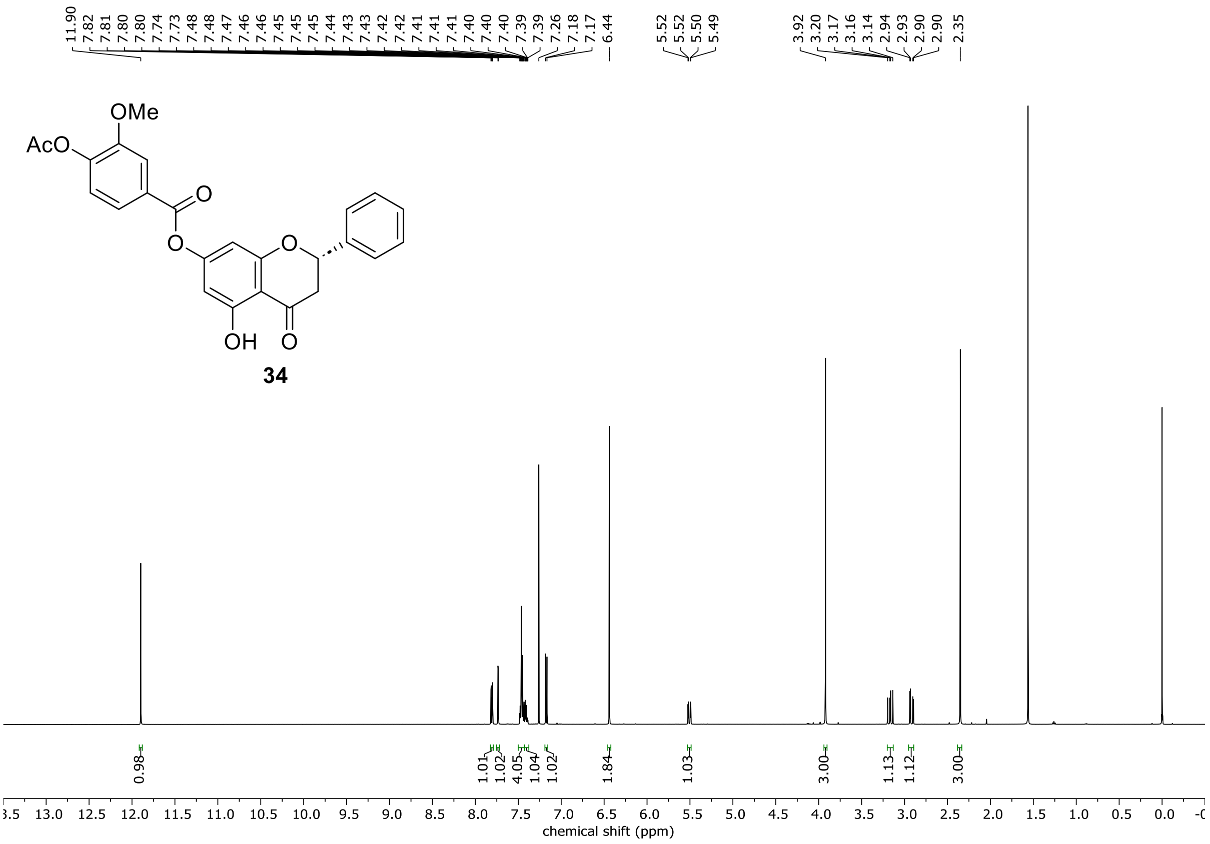

Figure S51. ^{1}H-NMR spectrum (500 MHz, $CDCl_3$) of 7-*O*-(*O*-acetylvanilloyl)pinocembrin (**34**).

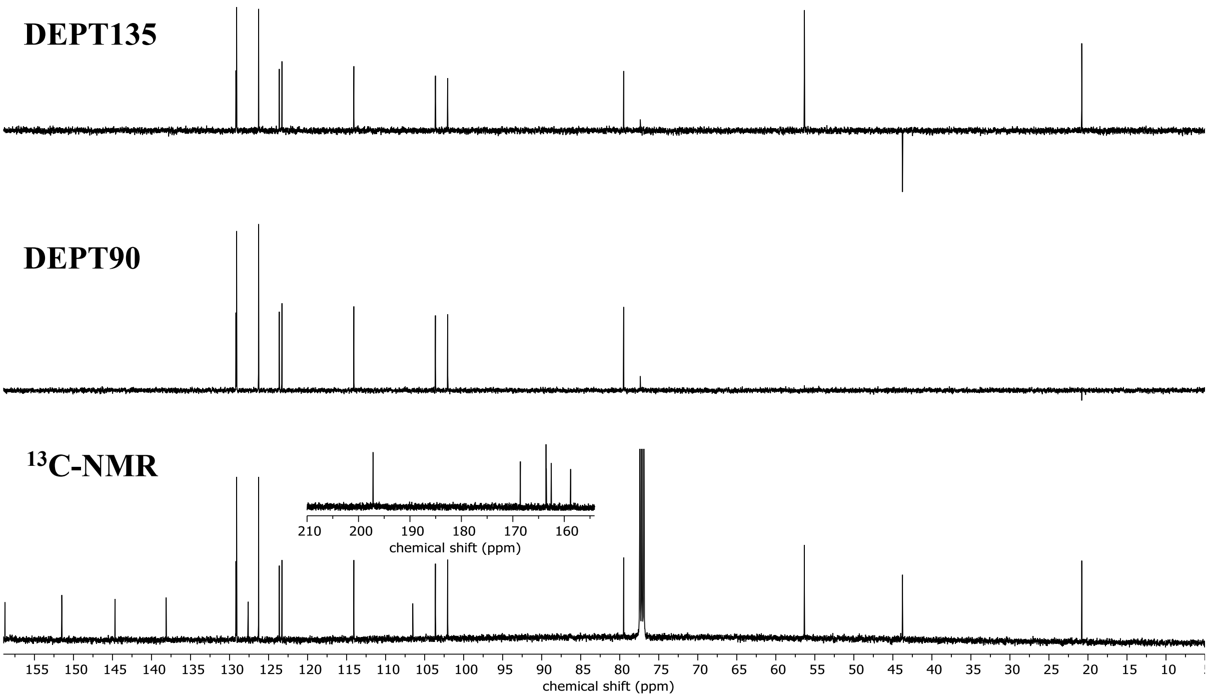

Figure S52. ^{13}C-NMR, DEPT90, and DEPT135 spectra (125 MHz, $CDCl_3$) of 7-*O*-(*O*-acetylvanilloyl)pinocembrin (**34**).

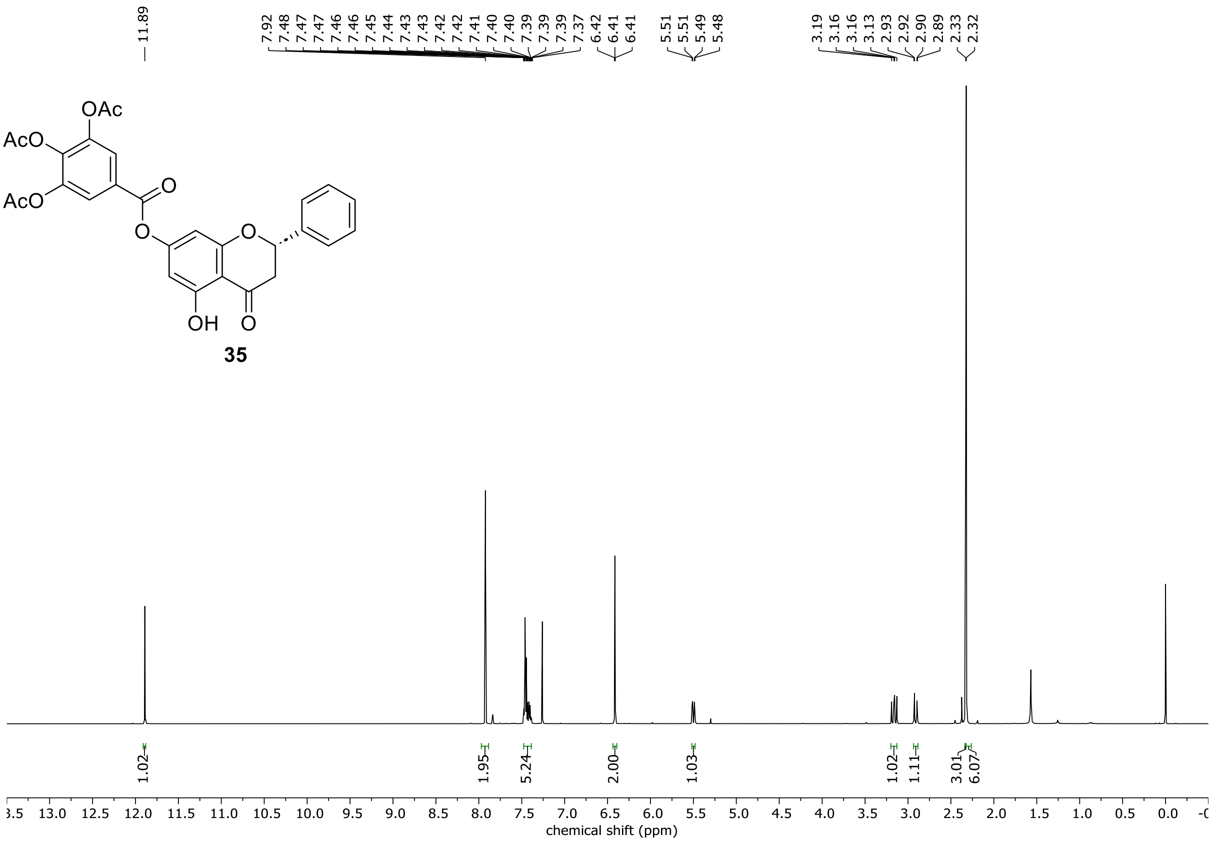

Figure S53. ^{1}H-NMR spectrum (500 MHz, $CDCl_3$) of 7-*O*-(tri-*O*-acetylgalloyl)pinocembrin (**35**).

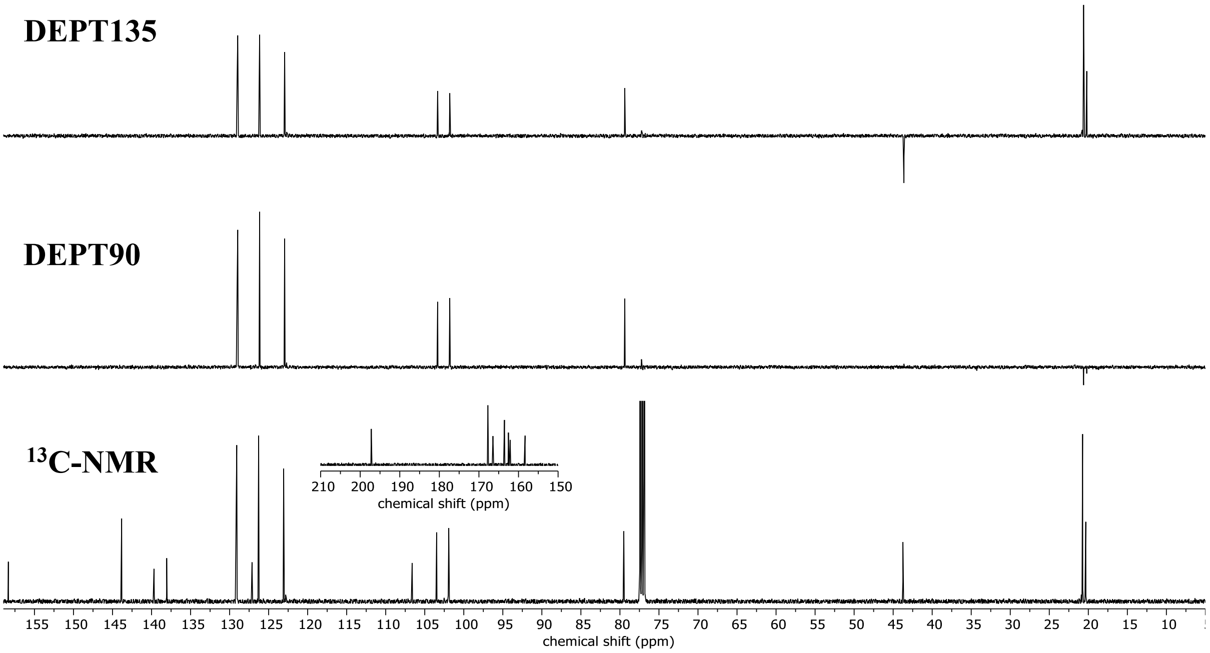

Figure S54. ^{13}C-NMR, DEPT90, and DEPT135 spectra (125 MHz, $CDCl_3$) of 7-*O*-(tri-*O*-acetylgalloyl)pinocembrin (**35**).

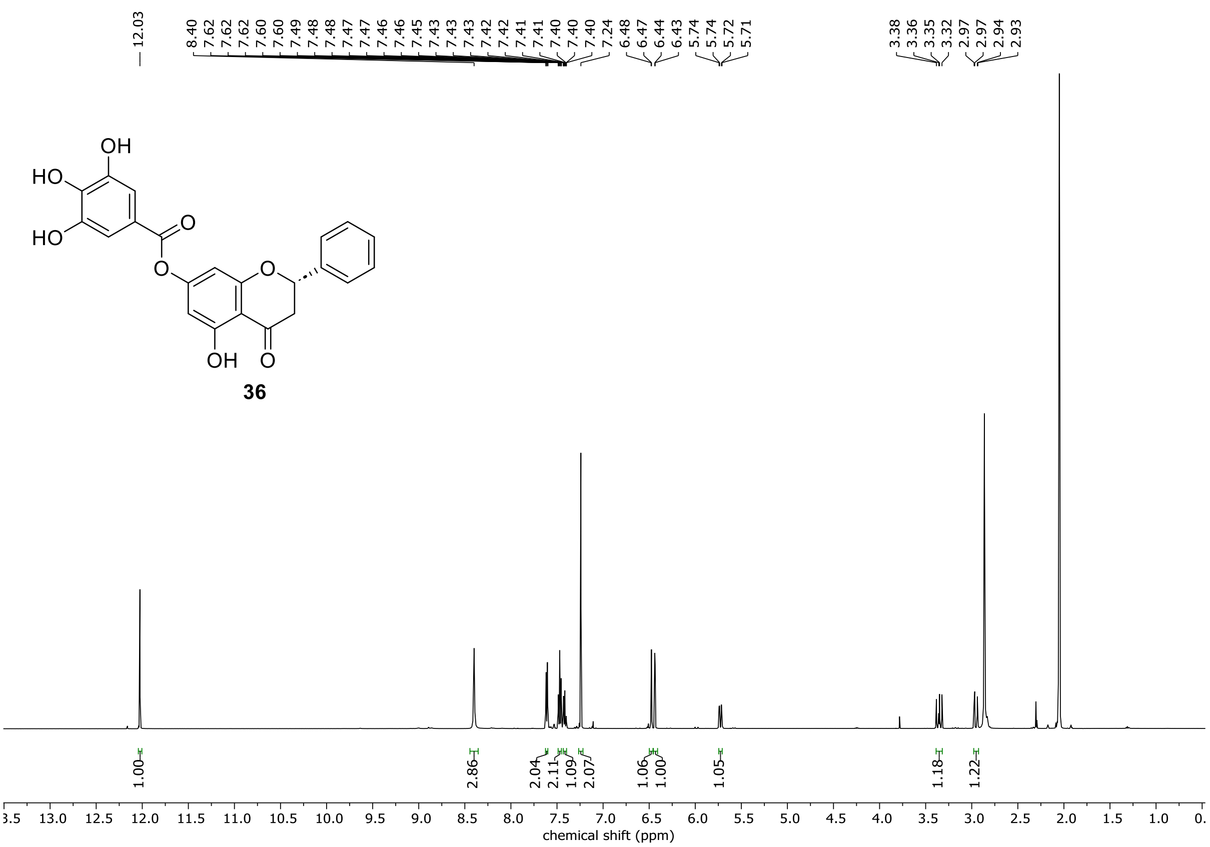

Figure S55. ^{1}H-NMR spectrum (500 MHz, acetone-d_6) of 7-*O*-galloylpinocembrin (**36**).

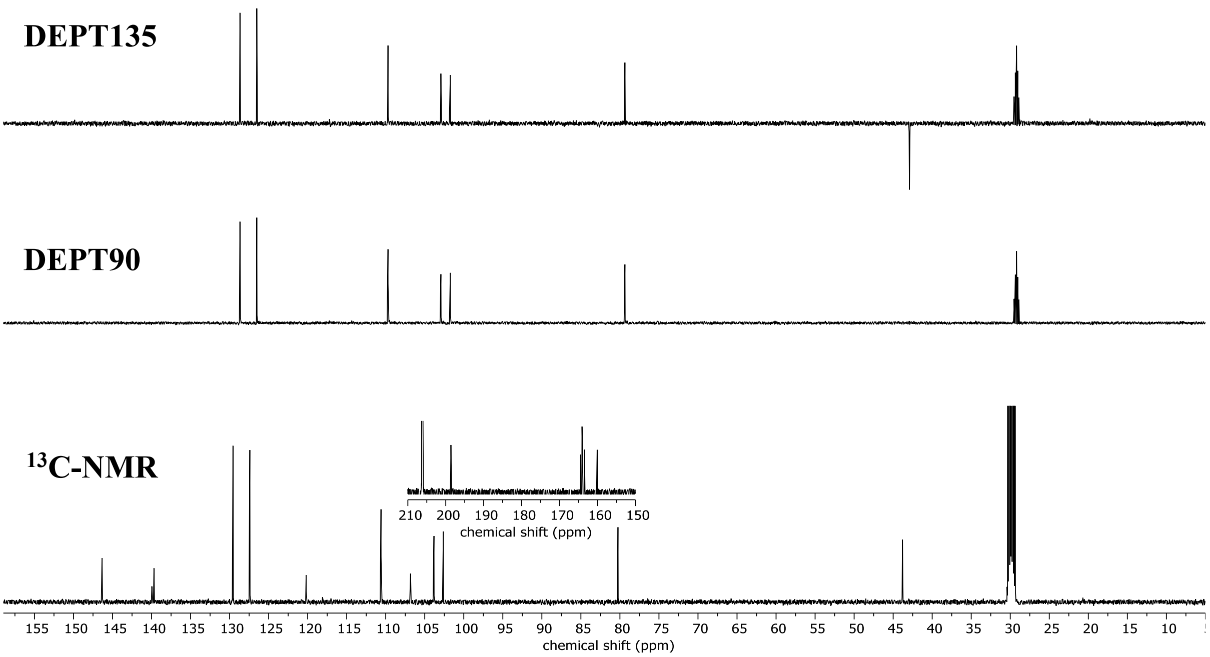

Figure S56. ^{13}C-NMR, DEPT90, and DEPT135 spectra (125 MHz, acetone-d_6) of 7-*O*-galloylpinocembrin (**36**).

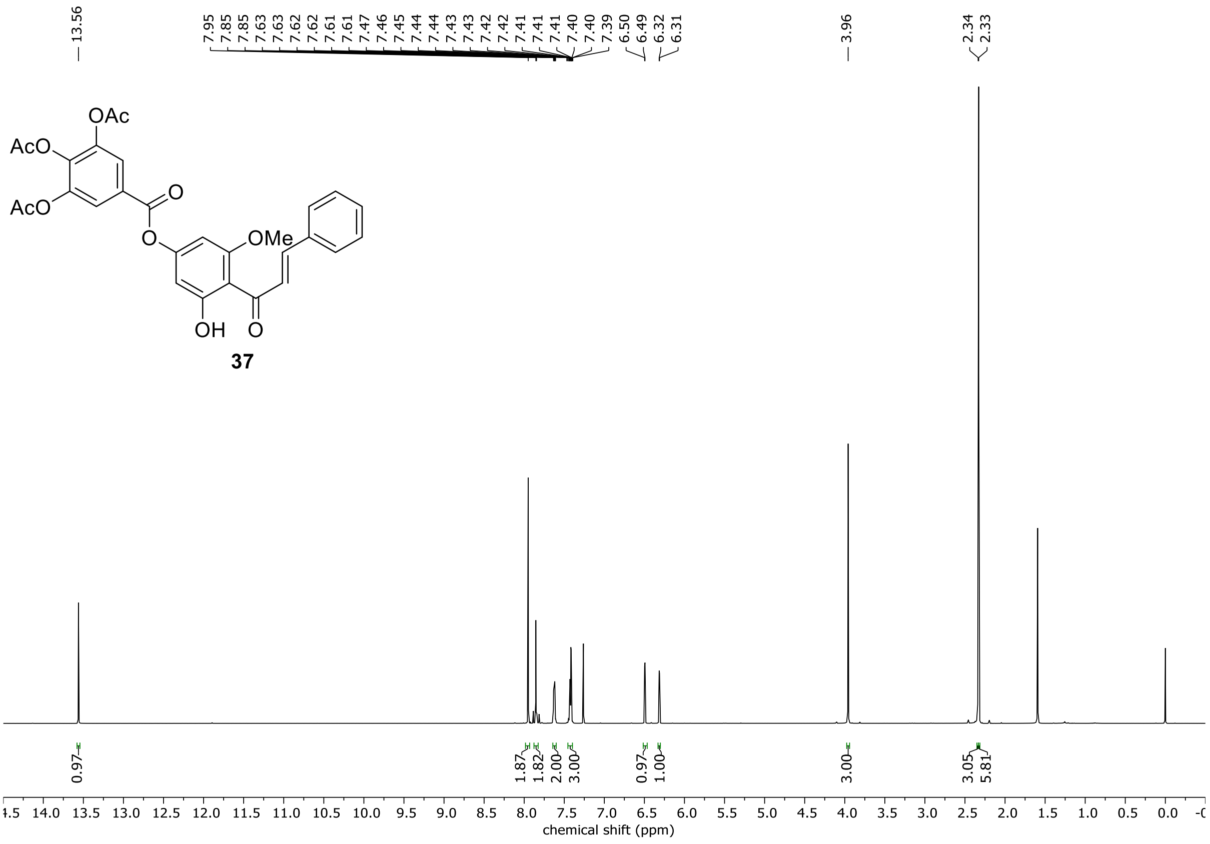

Figure S57. ^{1}H-NMR spectrum (500 MHz, $CDCl_3$) of 4′-*O*-(tri-*O*-acetylgalloyl)cardamonin (**37**).

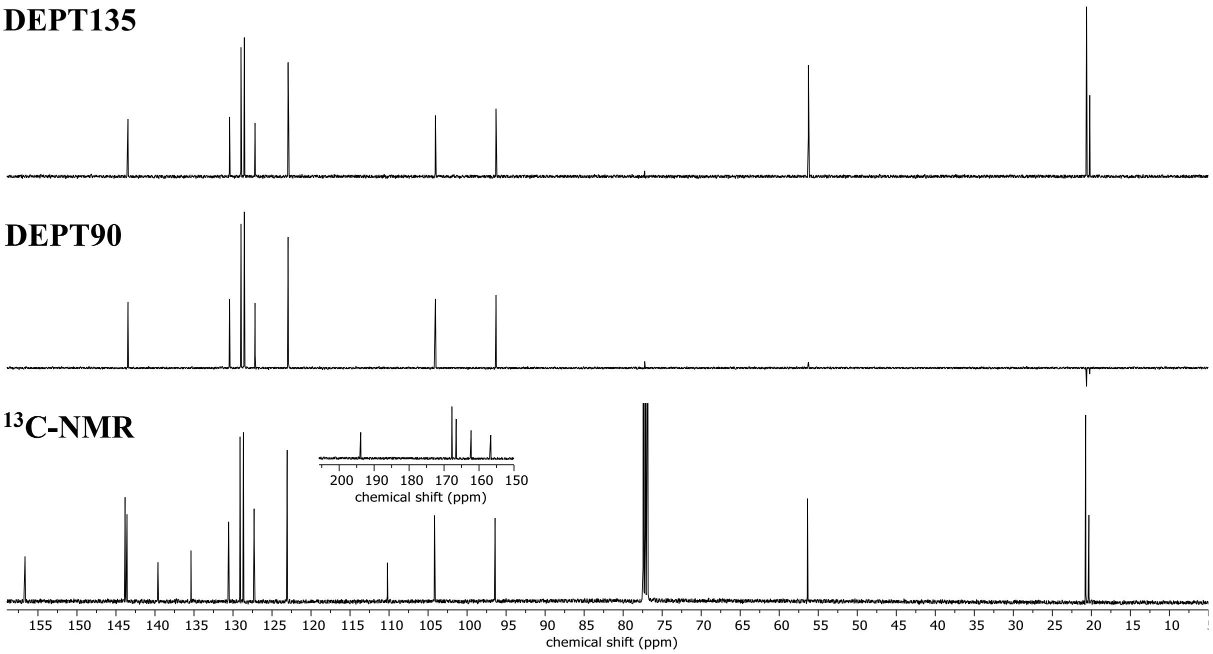

Figure S58. ^{13}C-NMR, DEPT90, and DEPT135 spectra (125 MHz, $CDCl_3$) of 4′-*O*-(tri-*O*-acetylgalloyl)cardamonin (**37**).

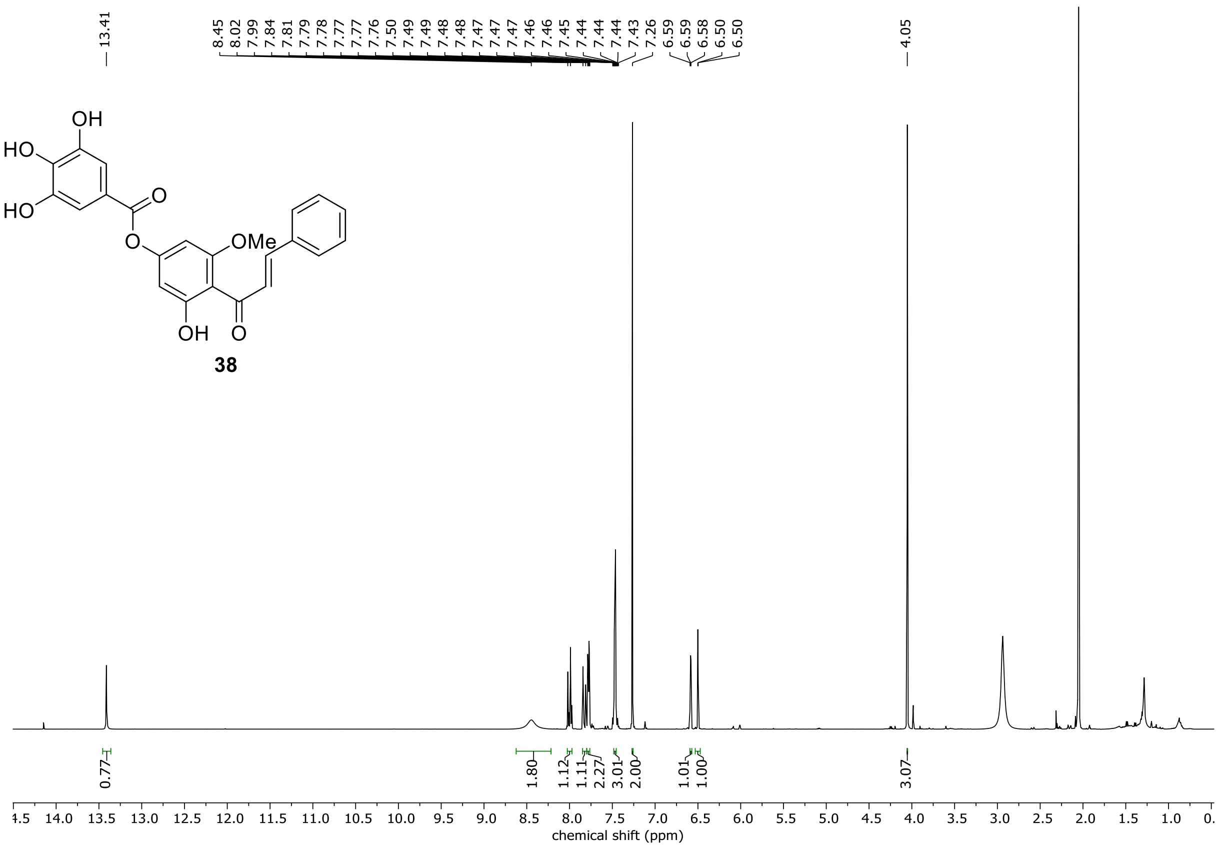

Figure S59. ^{1}H-NMR spectrum (500 MHz, acetone-d_6) of 4′-*O*-galloylcardamonin (**38**).

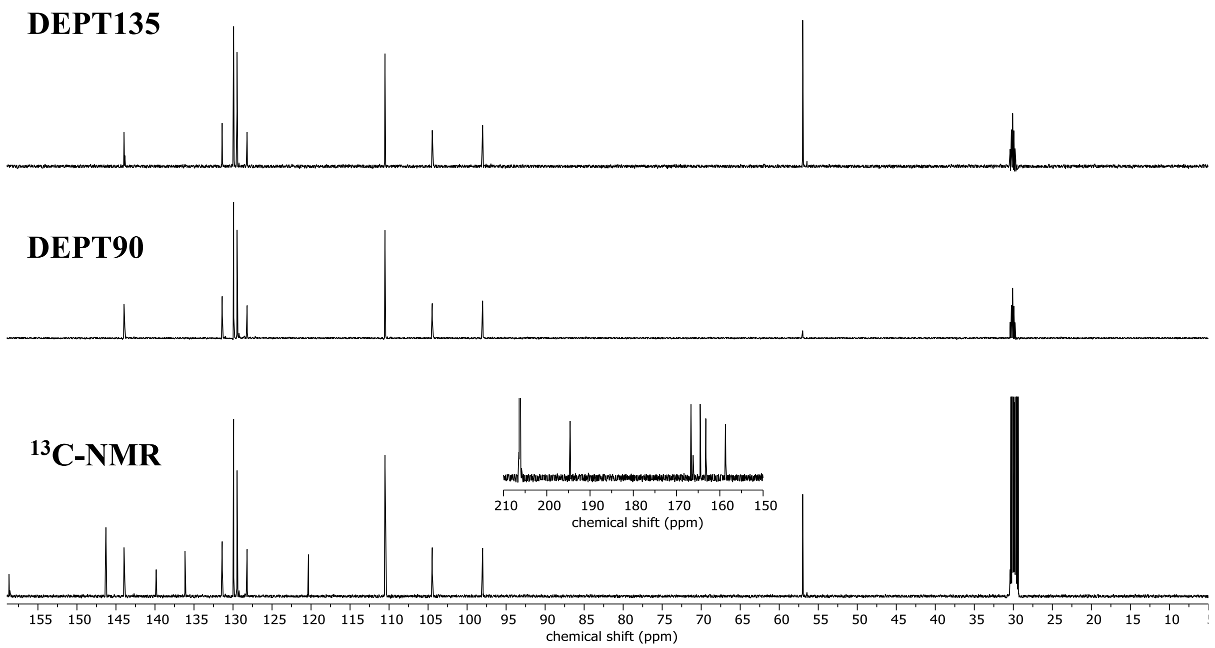

Figure S60. ^{13}C-NMR, DEPT90, and DEPT135 spectra (125 MHz, $CDCl_3$) of 4′-*O*-galloylcardamonin (**38**).

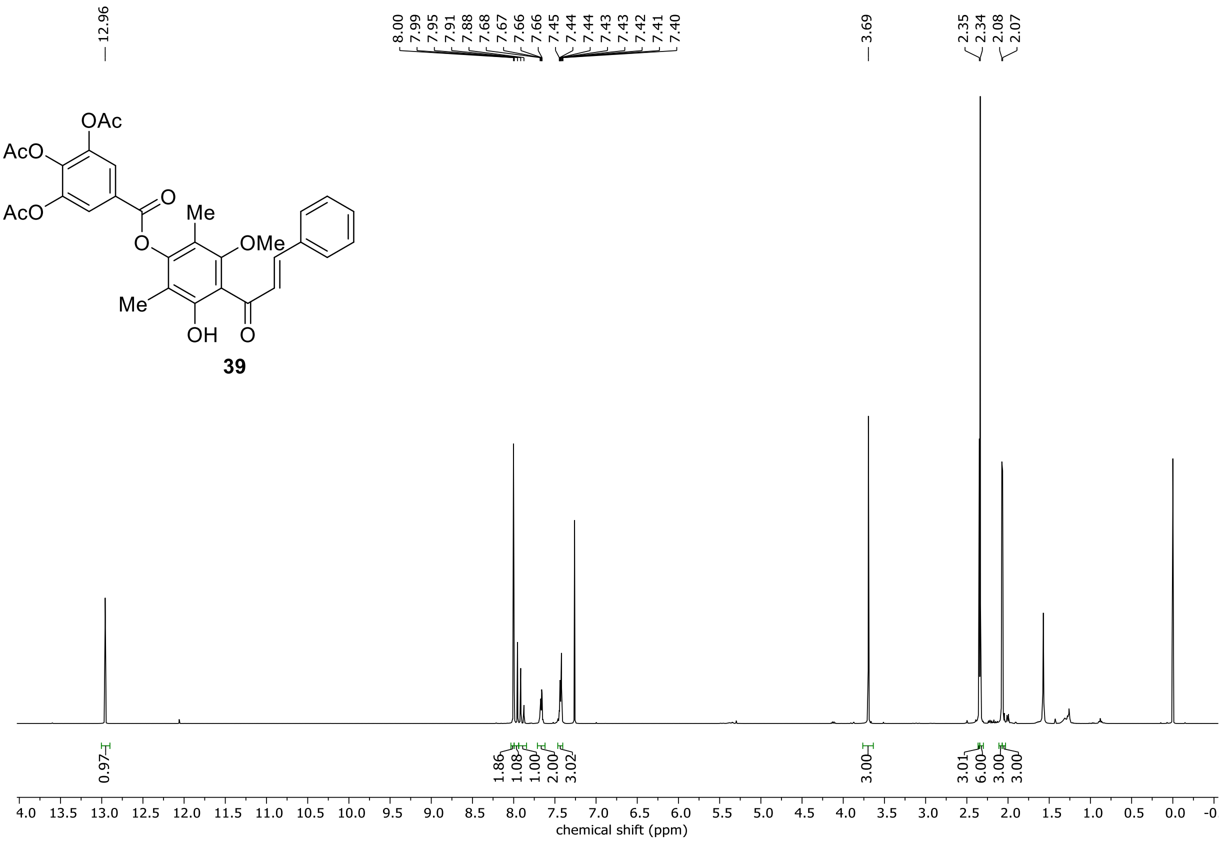

Figure S61. ^{1}H-NMR spectrum (500 MHz, $CDCl_3$) of 4′-*O*-(tri-*O*-acetylgalloyl)-DMC (**39**)

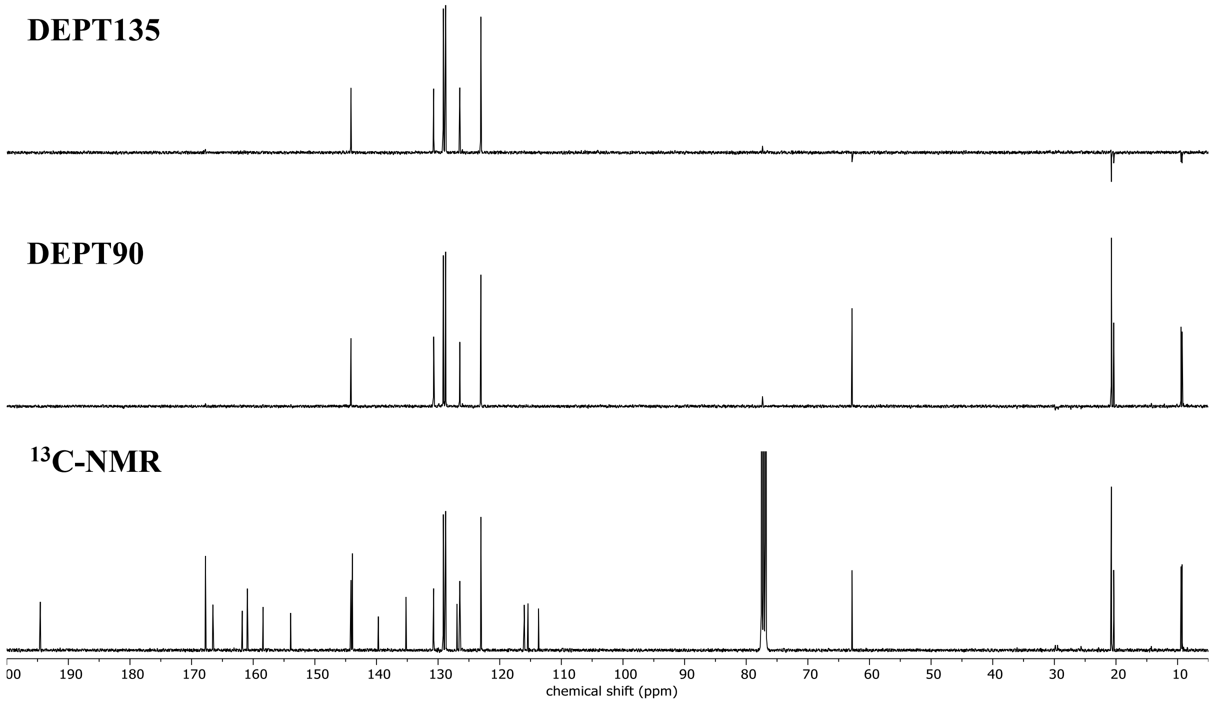

Figure S62. ^{13}C-NMR, DEPT90, and DEPT135 spectra (125 MHz, $CDCl_3$) of 4′-*O*-(tri-*O*-acetylgalloyl)-DMC (**39**)

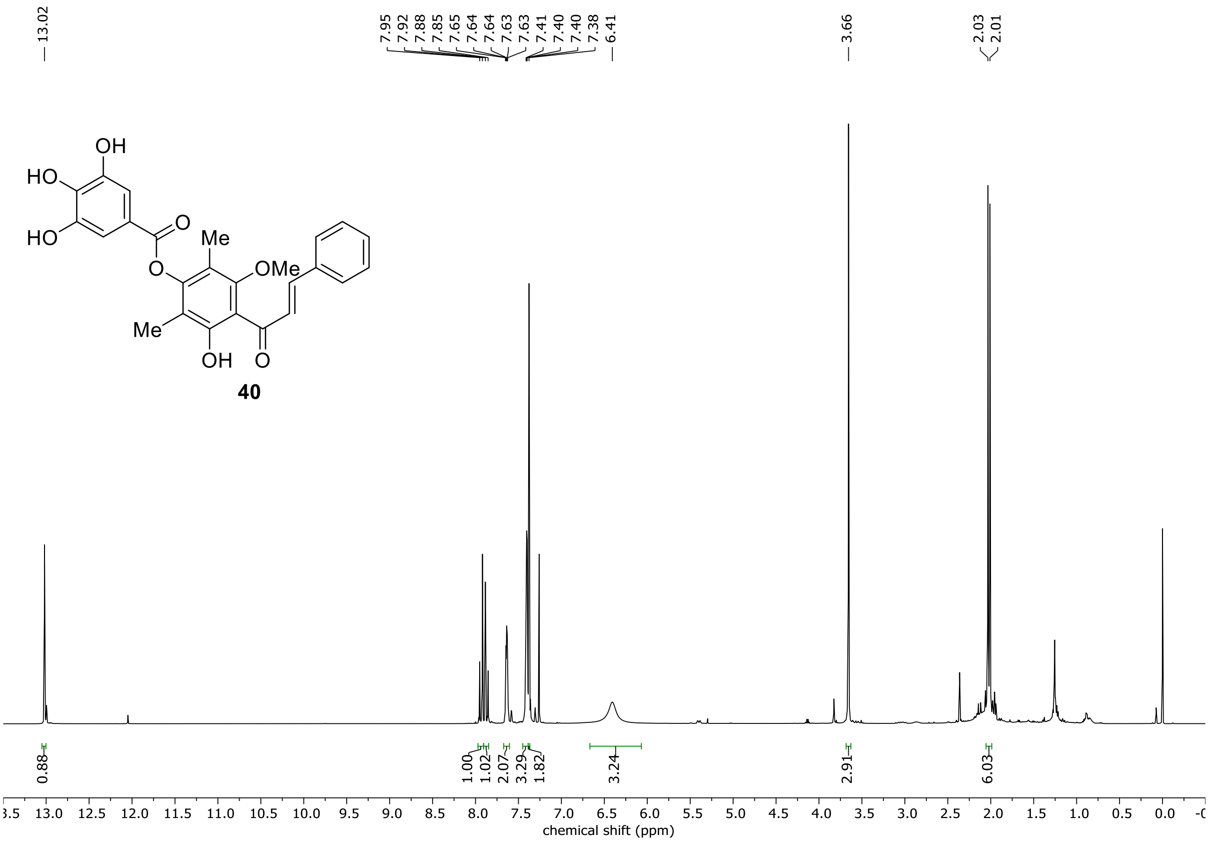

Figure S63. ^{1}H-NMR spectrum (500 MHz, $CDCl_3$) of 4′-*O*-galloyl-DMC (**40**).

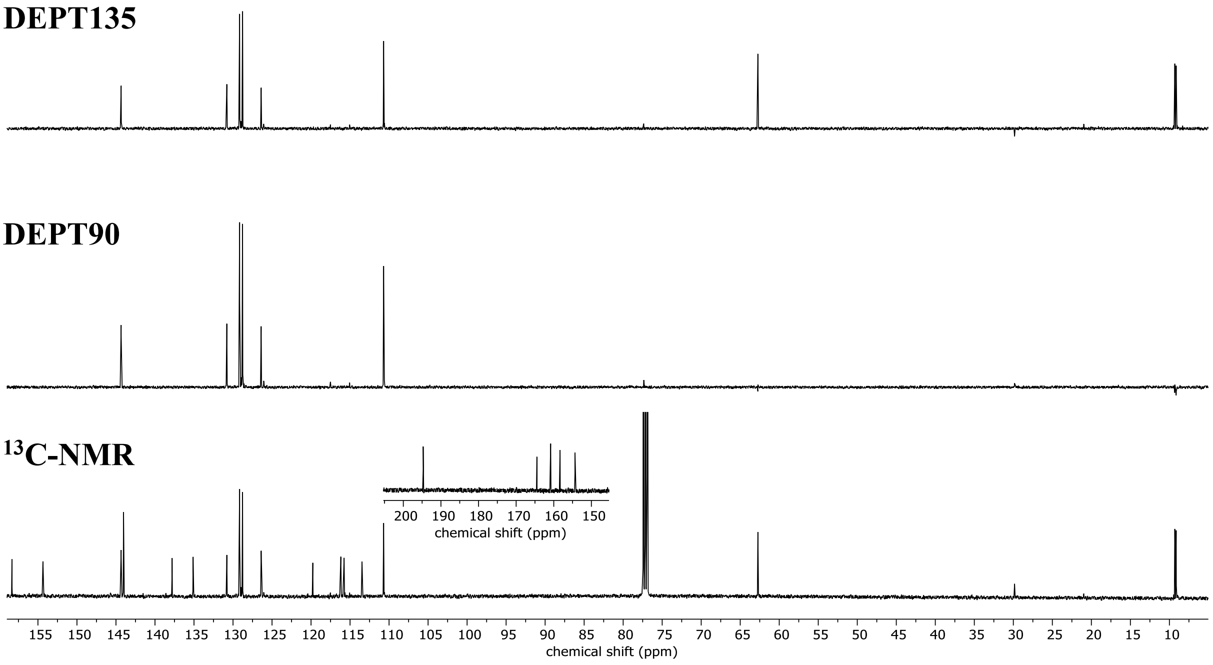

Figure S64. ^{13}C-NMR, DEPT90, and DEPT135 spectra (125 MHz, $CDCl_3$) of 4′-*O*-galloyl-DMC (**40**).

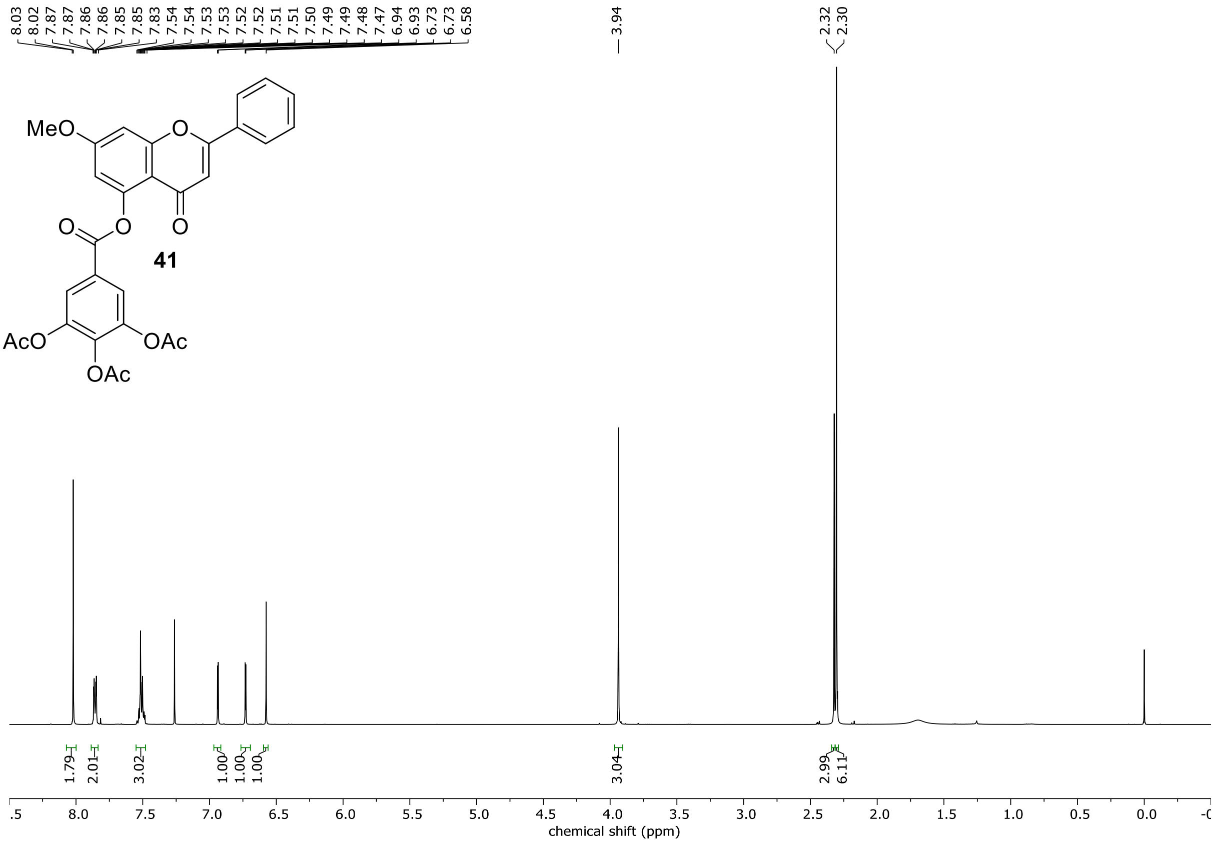

Figure S65. ^{1}H-NMR spectrum (500 MHz, $CDCl_3$) of 5-*O*-(tri-*O*-acetylgalloyl)tectochrysin (**41**).

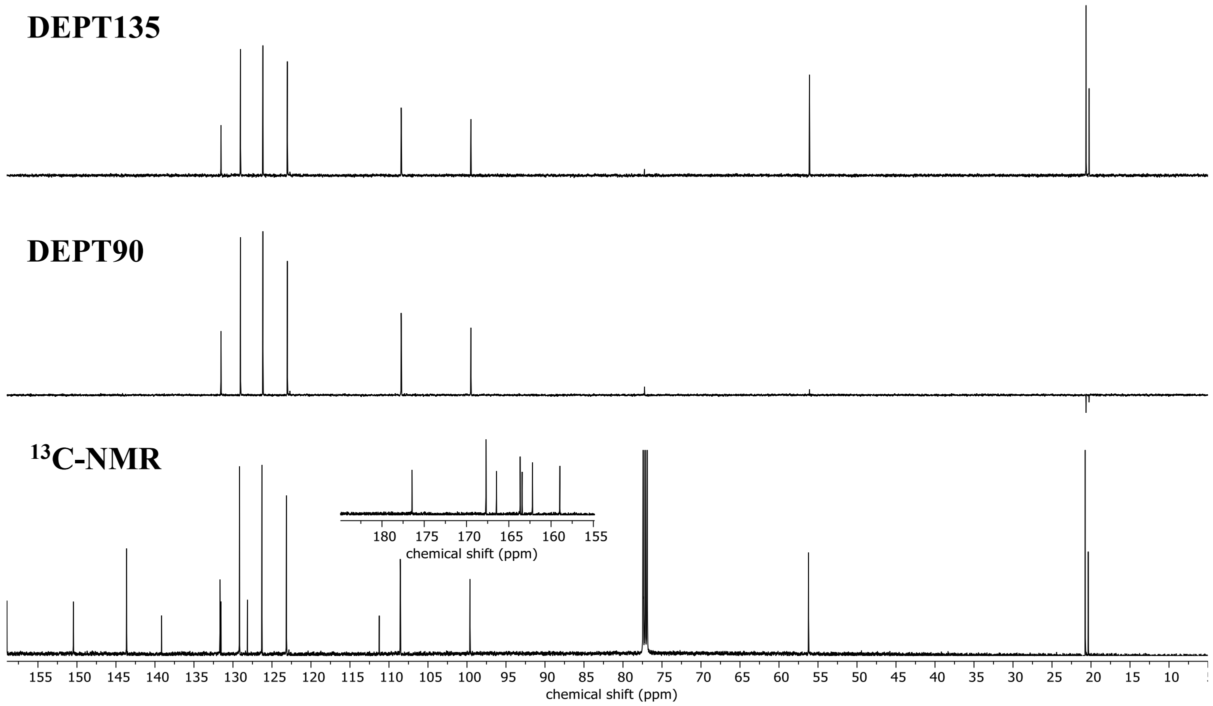

Figure S66. ^{13}C-NMR, DEPT90, and DEPT135 spectra (125 MHz, $CDCl_3$) of 5-*O*-(tri-*O*-acetylgalloyl)tectochrysin (**41**).

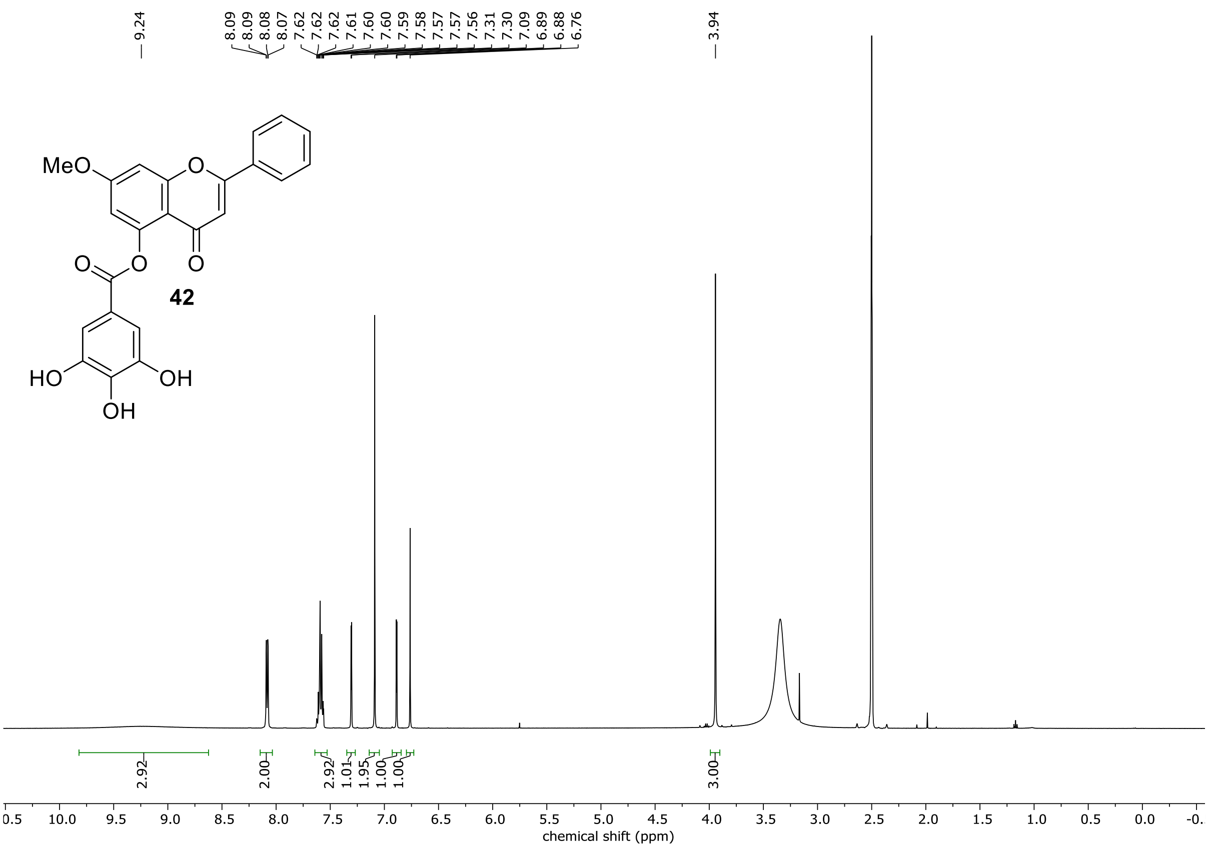

Figure S67. ^{1}H-NMR spectrum (500 MHz, DMSO-d_6) of 5-*O*-galloyltectochrysin (**42**).

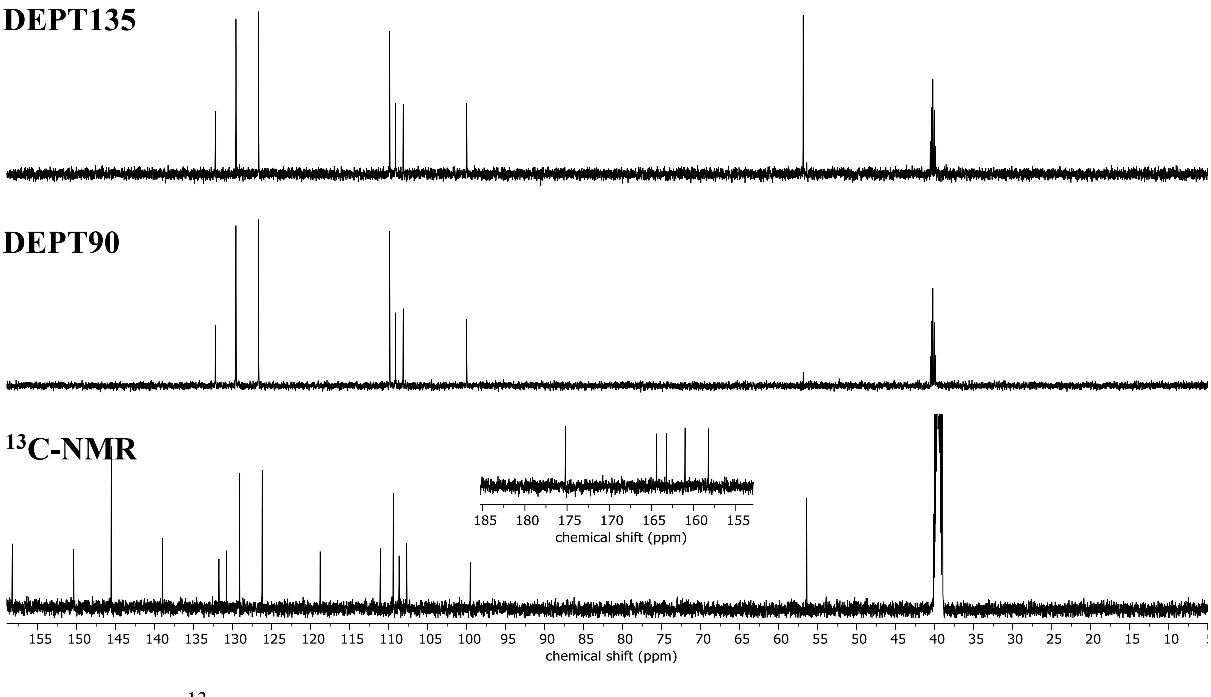

Figure S68. ^{13}C-NMR, DEPT90, and DEPT135 spectra (125 MHz, DMSO-d_6) of 5-*O*-galloyltectochrysin (**42**).

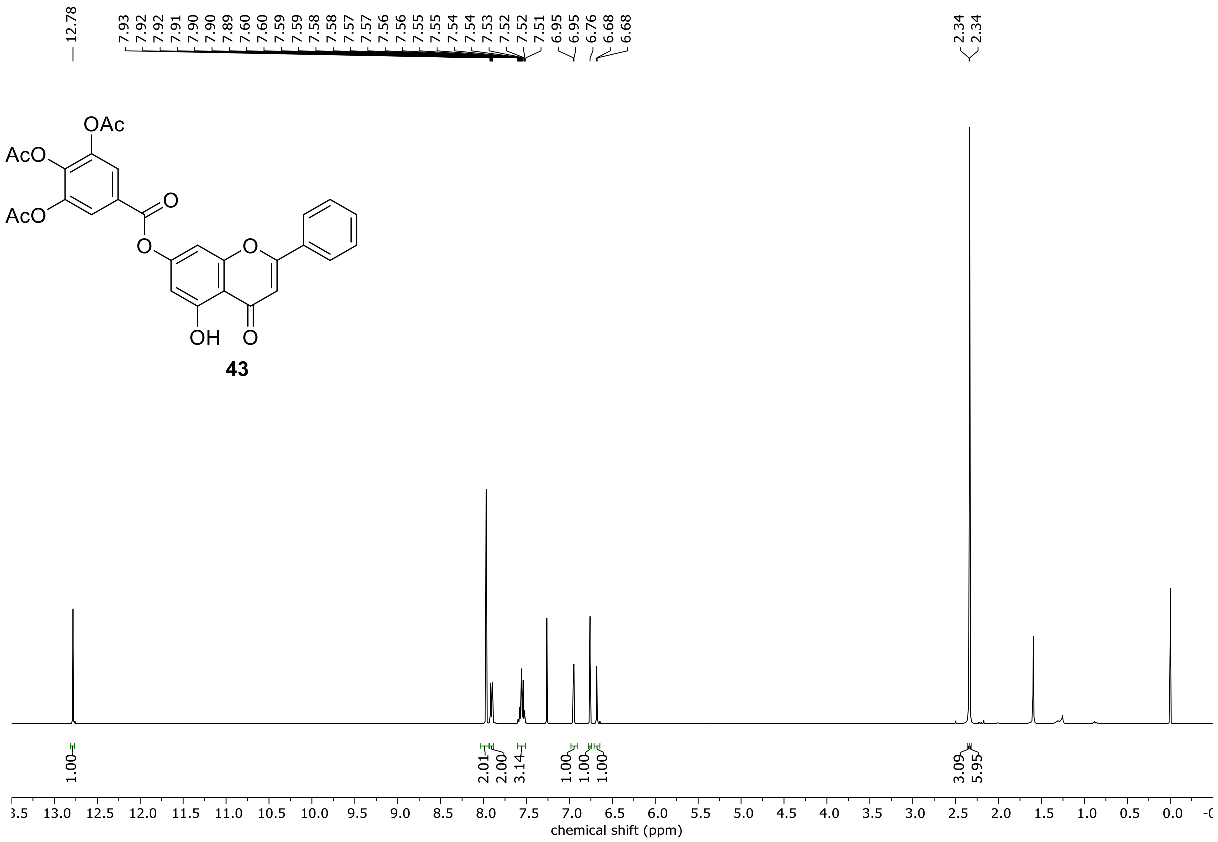

Figure S69. ^{1}H-NMR spectrum (500 MHz, $CDCl_3$) of 7-*O*-(tri-*O*-acetylgalloyl)chrysin (**43**).

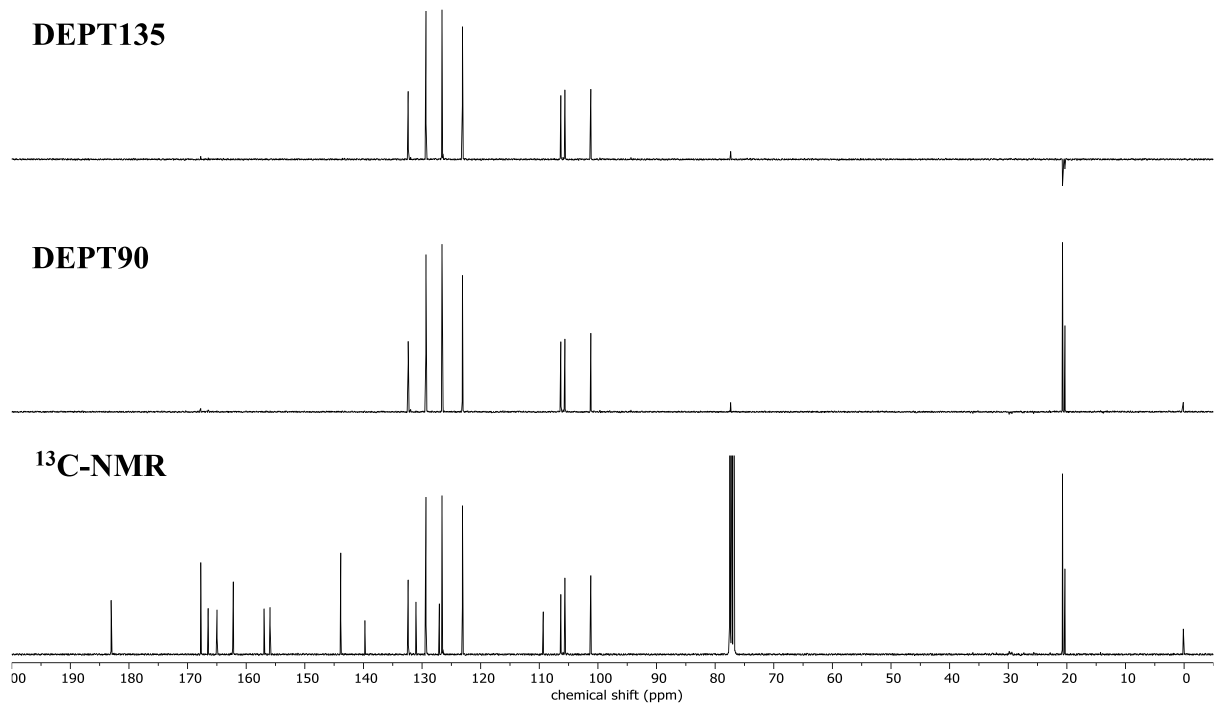

Figure S70. ^{13}C-NMR, DEPT90, and DEPT135 spectra (125 MHz, DMSO-d_6) of 7-*O*-(tri-*O*-acetylgalloyl)chrysin (**43**).

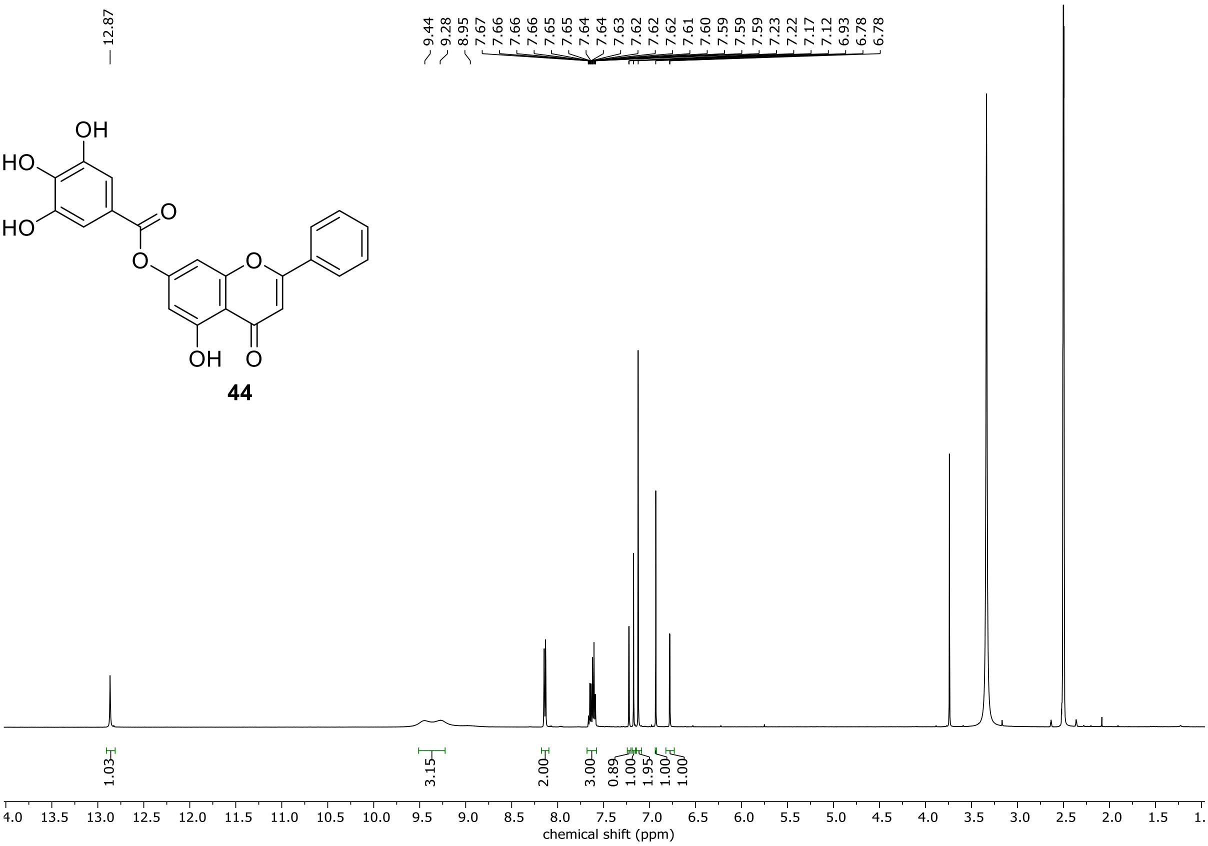

Figure S71. ^{1}H-NMR spectrum (500 MHz, DMSO-d_6) of 7-*O*-galloylchrysin (**44**).

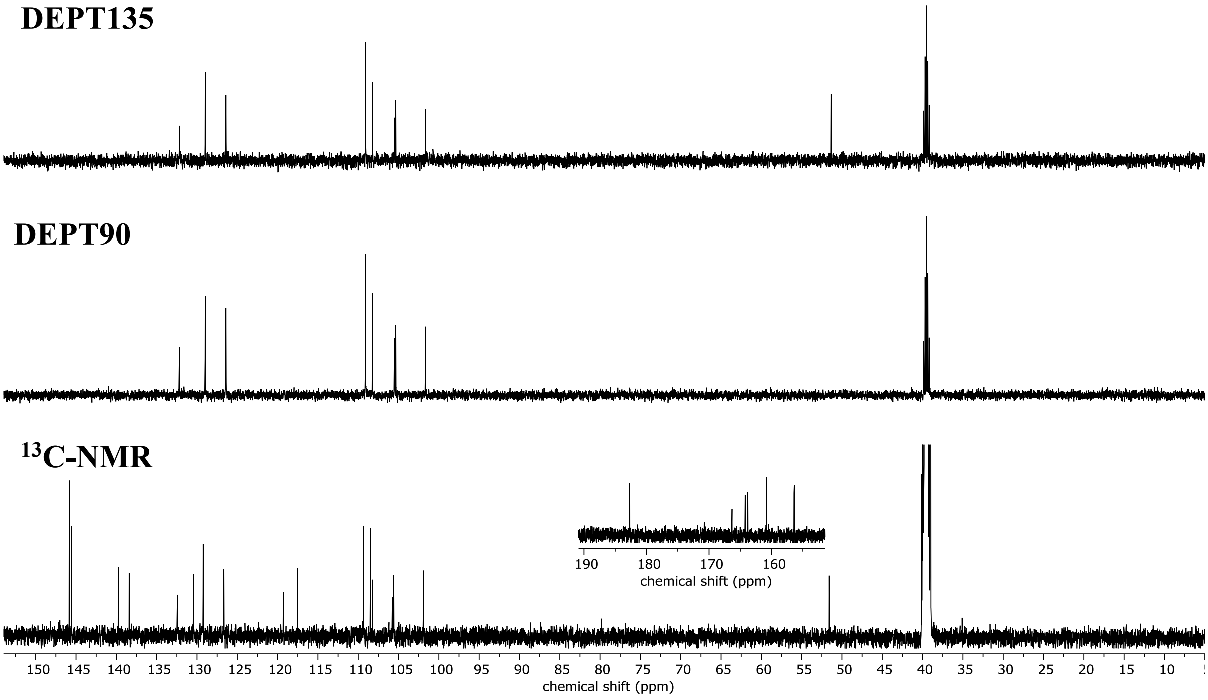

Figure S72. ^{13}C-NMR, DEPT90, and DEPT135 spectra (125 MHz, DMSO-d_6) of 7-*O*-galloylchrysin (**44**).

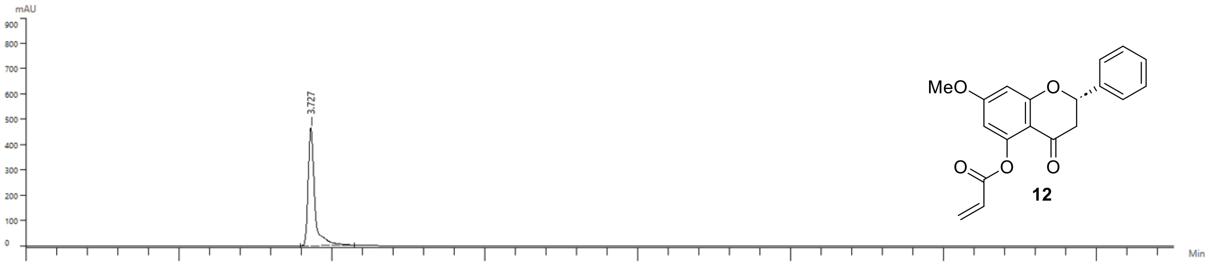

Figure S73. HPLC chromatogram of **12** (Purity >99.9%).

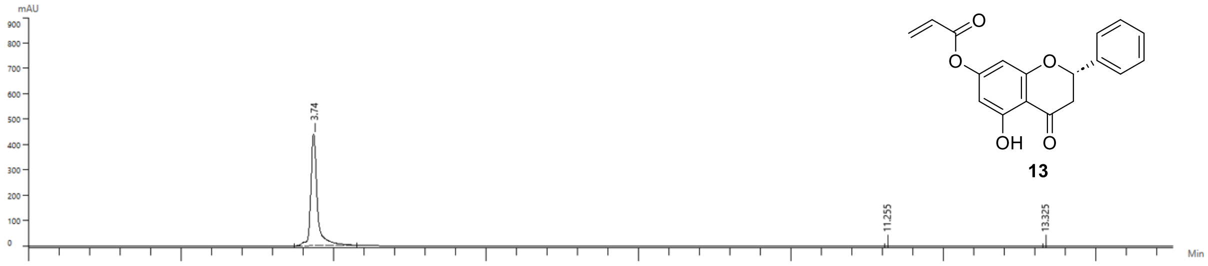

Figure S74. HPLC chromatogram of **13** (Purity >99.9%).

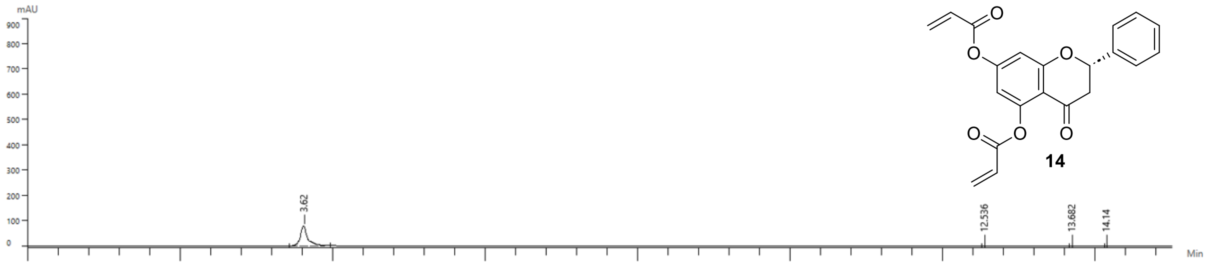

Figure S75. HPLC chromatogram of **14** (Purity 99.8%).

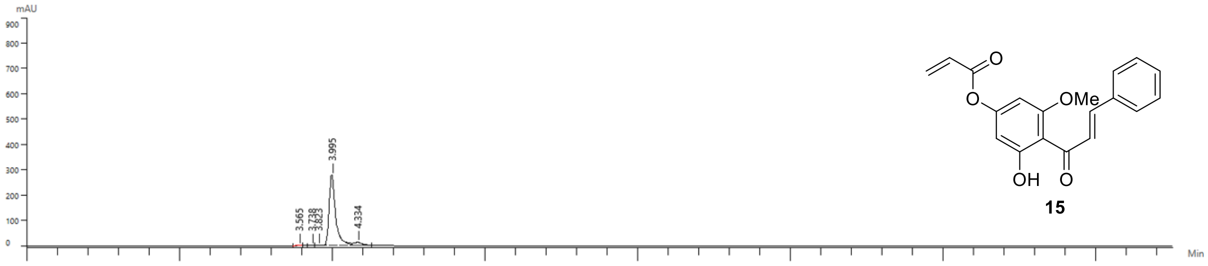

Figure S76. HPLC chromatogram of **15** (Purity 95.0%).

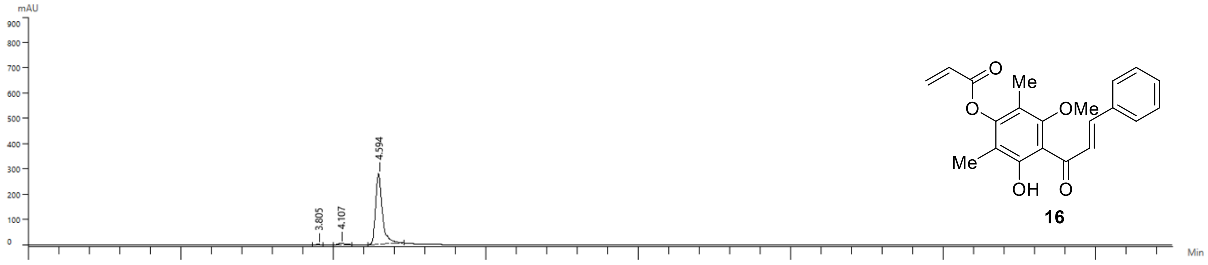

Figure S77. HPLC chromatogram of **16** (Purity 97.5%).

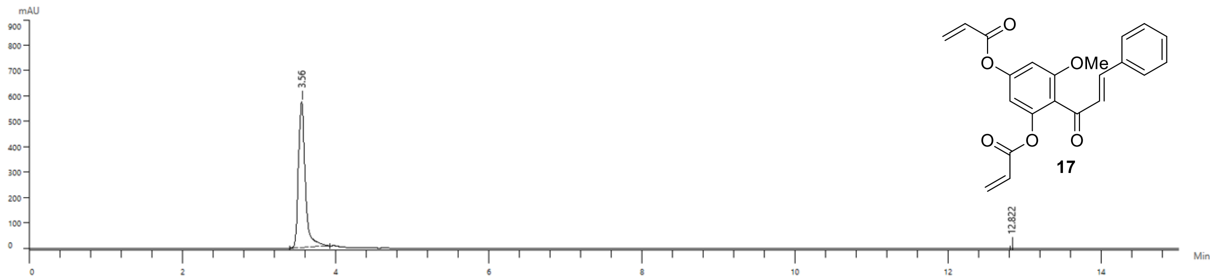

Figure S78. HPLC chromatogram of **17** (Purity >99.9%).

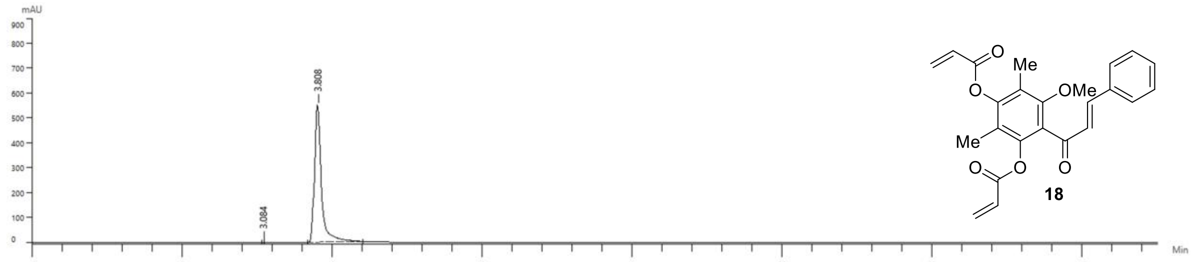

Figure S79. HPLC chromatogram of **18** (Purity >99.9%).

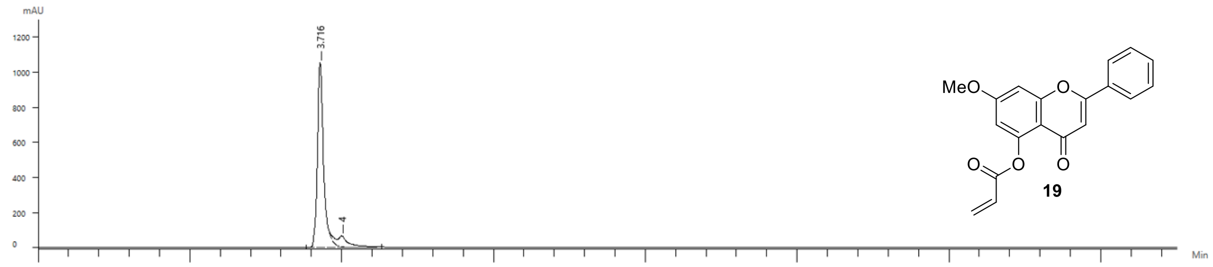

Figure S80. HPLC chromatogram of **19** (Purity 95.0%).

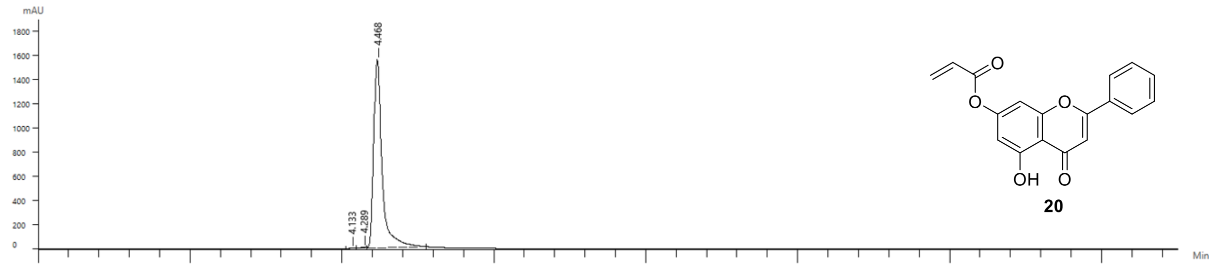

Figure S81. HPLC chromatogram of **20** (Purity 99.5%).

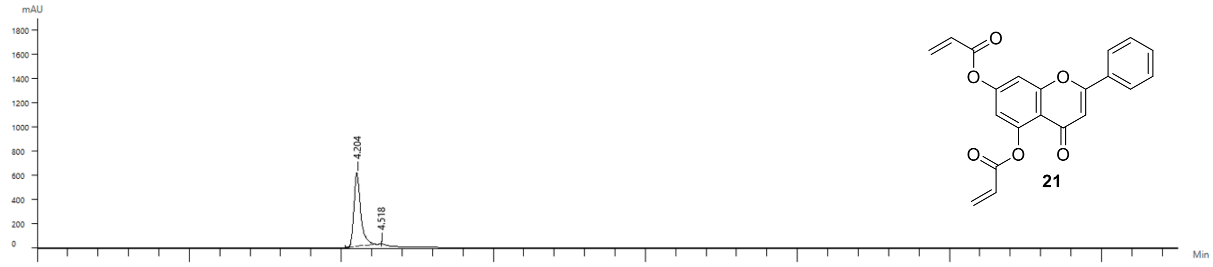

Figure S82. HPLC chromatogram of **21** (Purity >99.9%).

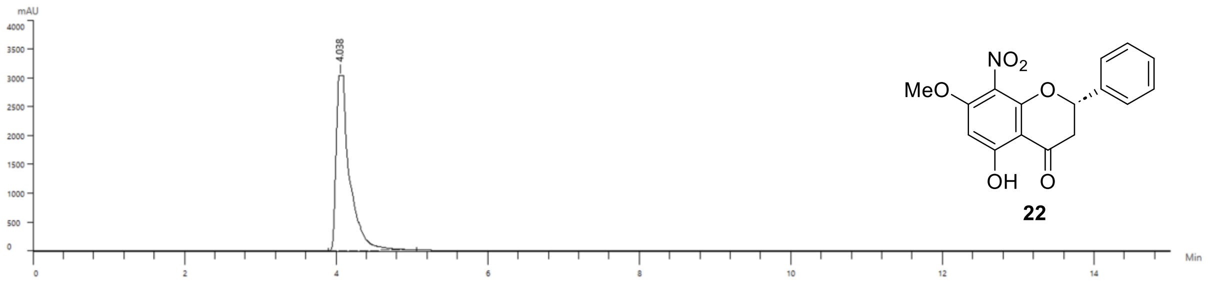

Figure S83. HPLC chromatogram of **22** (Purity >99.9%).

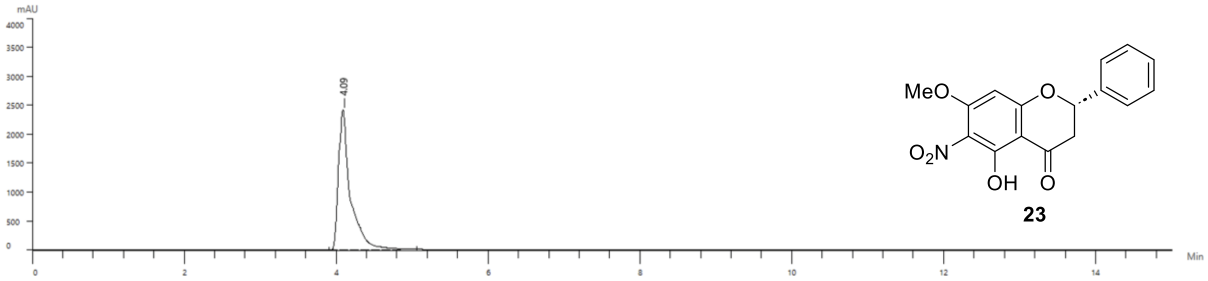

Figure S84. HPLC chromatogram of **23** (Purity >99.9%).

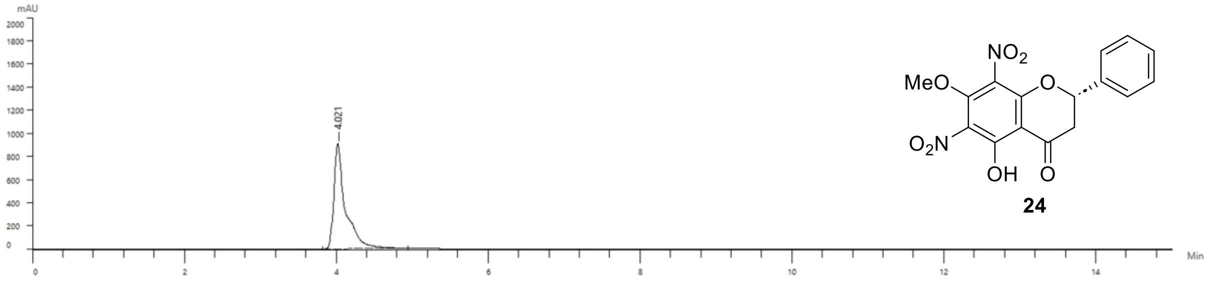

Figure S85. HPLC chromatogram of **24** (Purity >99.9%).

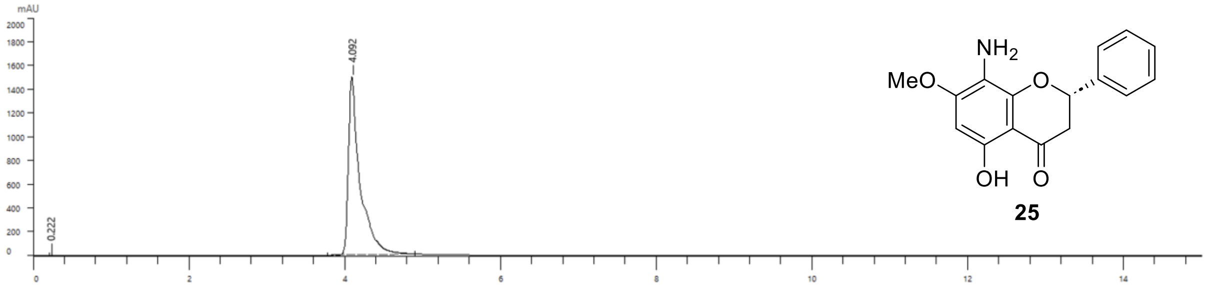

Figure S86. HPLC chromatogram of **25** (Purity >99.9%).

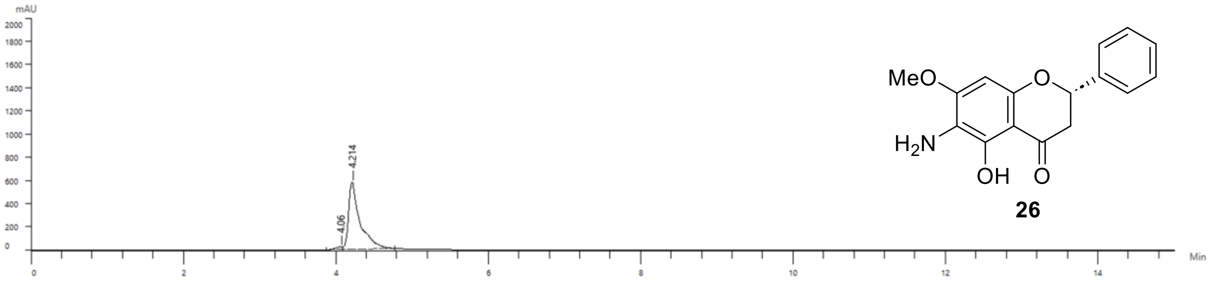

Figure S87. HPLC chromatogram of **26** (Purity 97.8%).

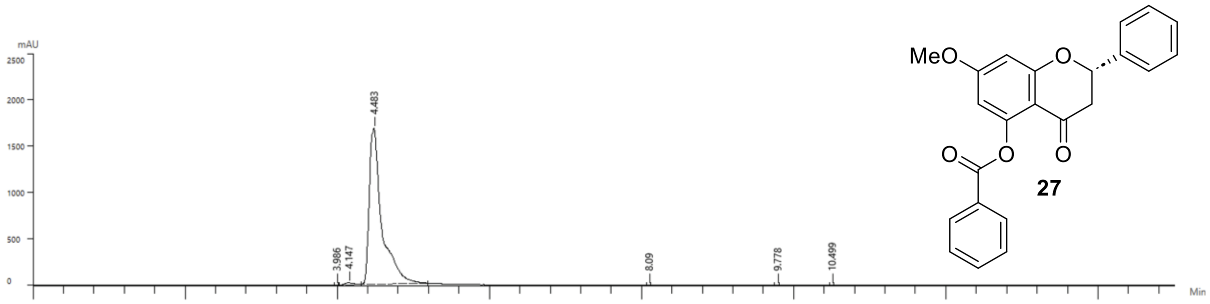

Figure S88. HPLC chromatogram of **27** (Purity 99.2%).

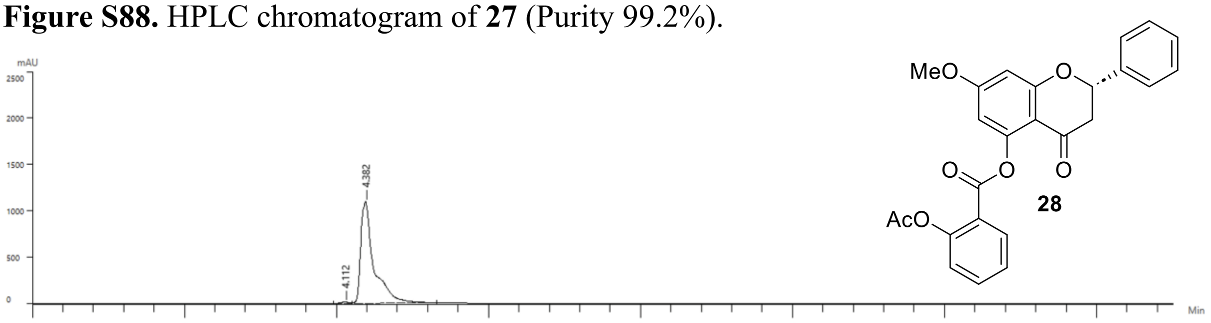

Figure S89. HPLC chromatogram of **28** (Purity 99.2%).

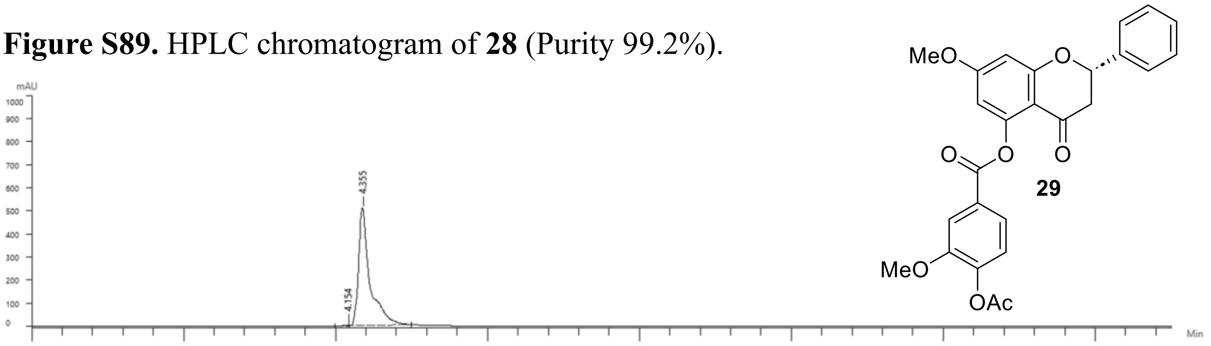

Figure S90. HPLC chromatogram of **29** (Purity 99.8%).

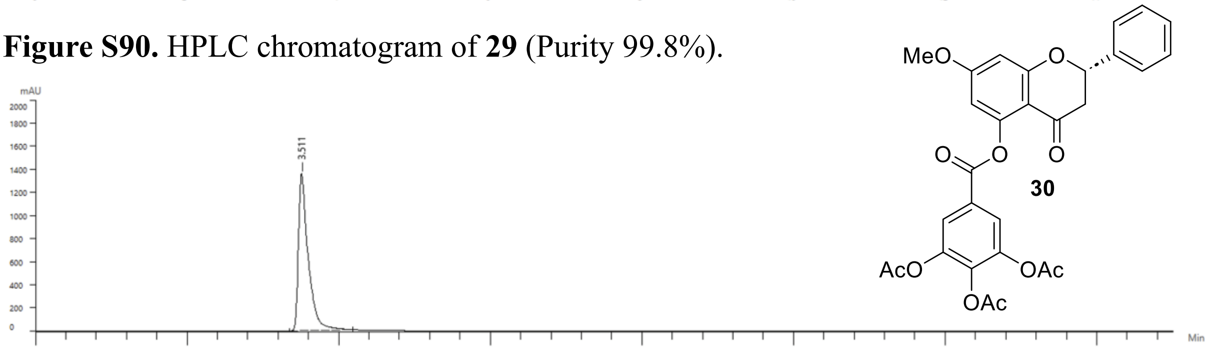

Figure S91. HPLC chromatogram of **30** (Purity >99.9%).

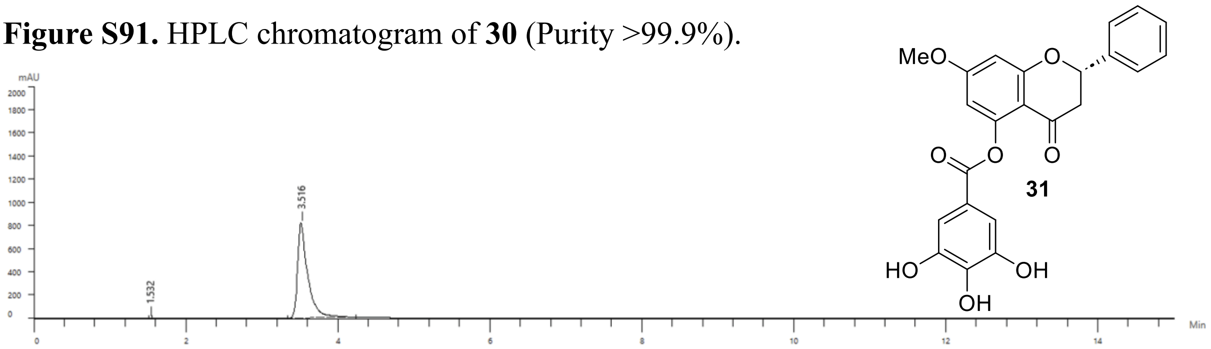

Figure S92. HPLC chromatogram of **31** (Purity >99.9%).

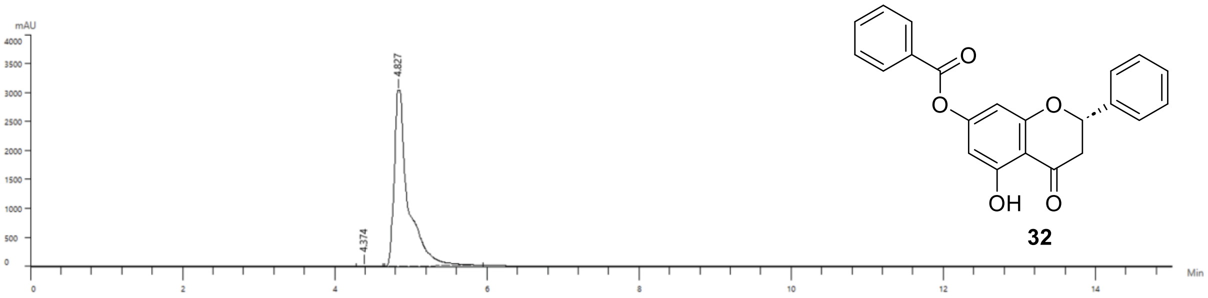

Figure S93. HPLC chromatogram of **32** (Purity >99.9%).

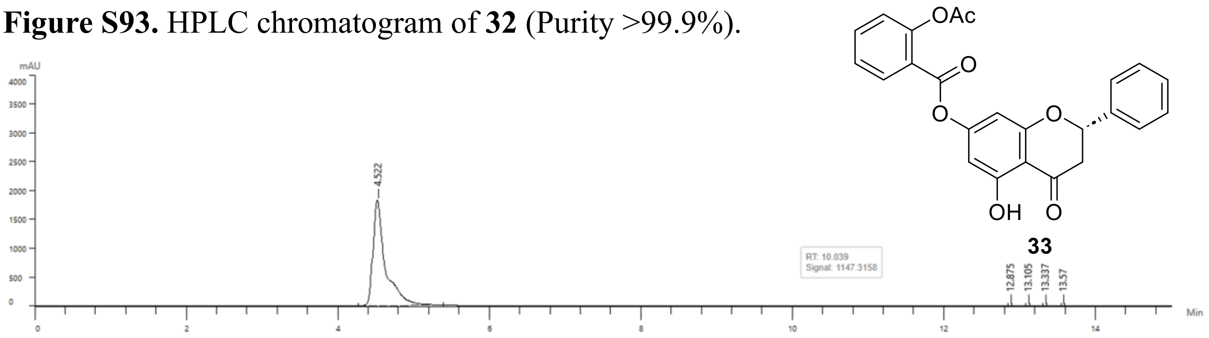

Figure S94. HPLC chromatogram of **33** (Purity >99.9%).

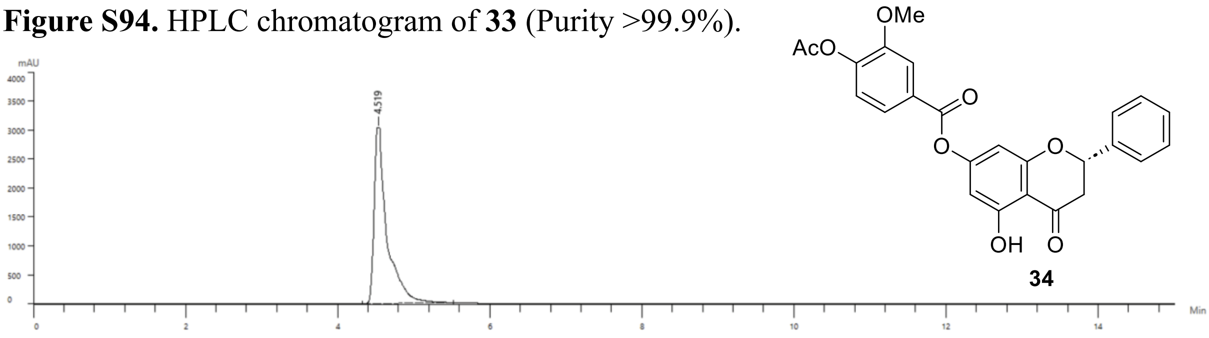

Figure S95. HPLC chromatogram of **34** (Purity >99.9%).

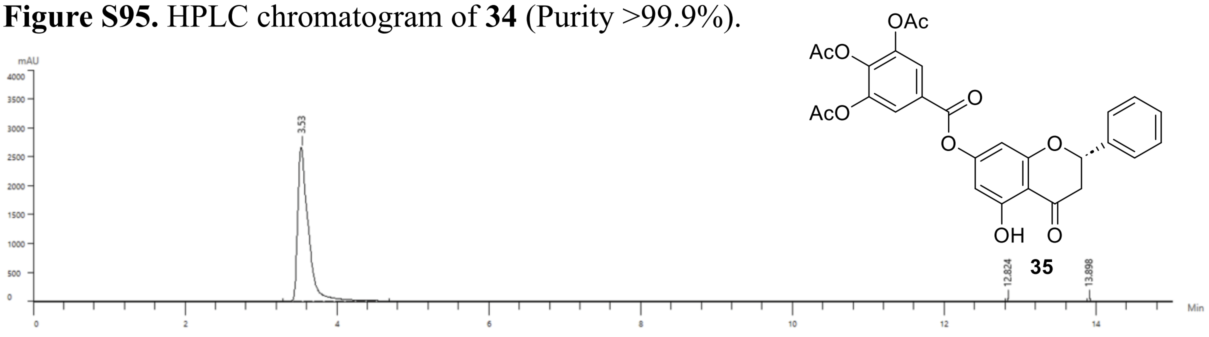

Figure S96. HPLC chromatogram of **35** (Purity >99.9%).

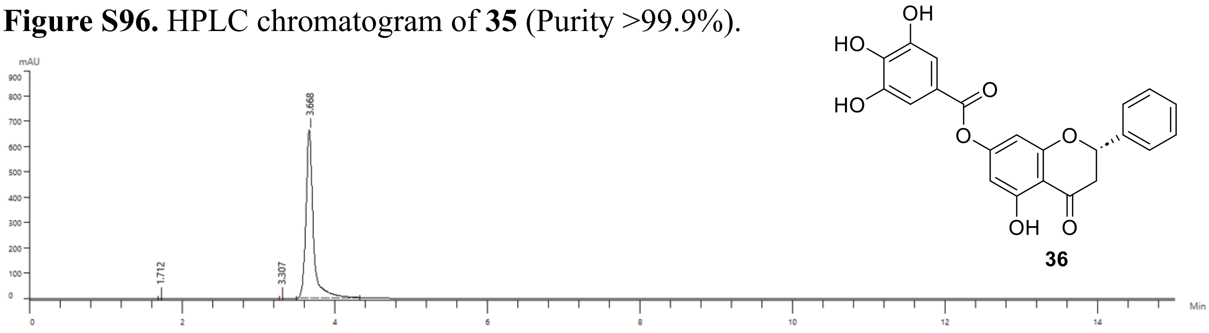

Figure S97. HPLC chromatogram of **35** (Purity >99.9%).

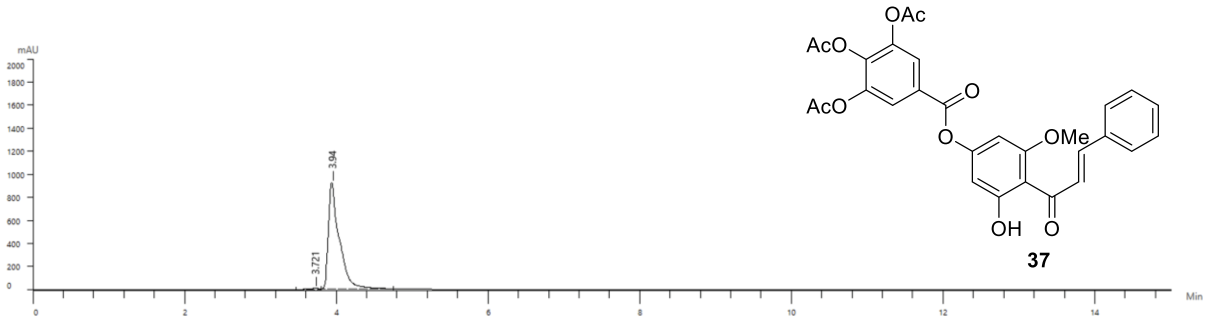

Figure S98. HPLC chromatogram of **37** (Purity 99.2%).

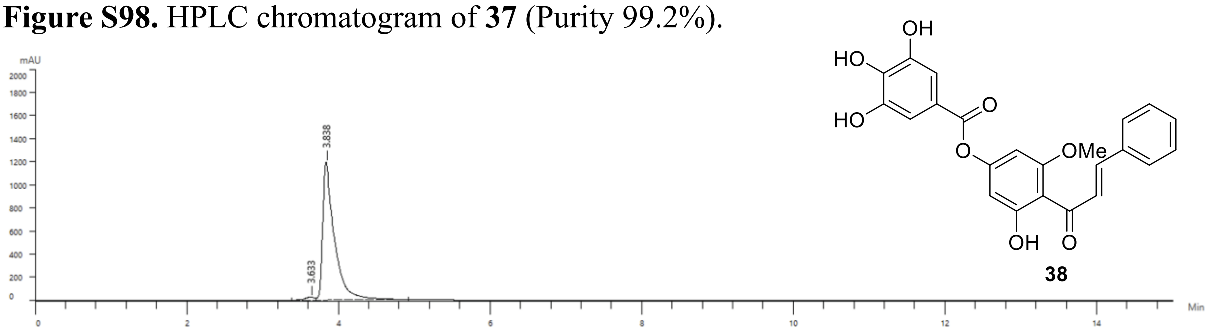

Figure S99. HPLC chromatogram of **38** (Purity 98.3%).

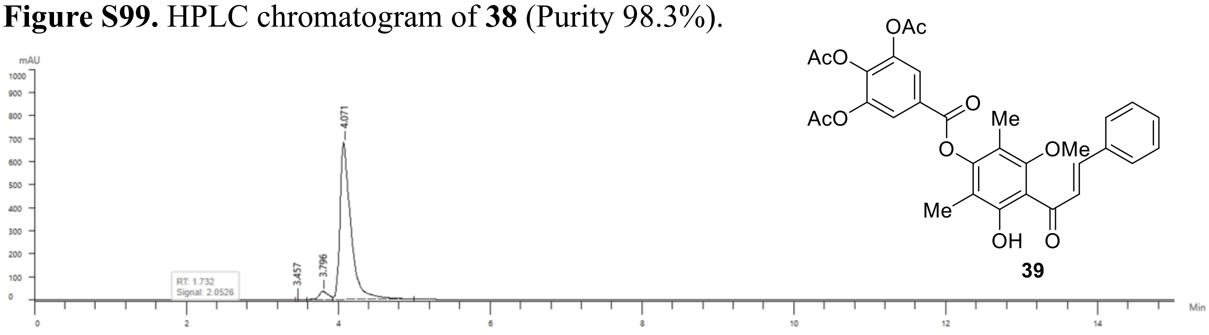

Figure S100. HPLC chromatogram of **39** (Purity 95.7%).

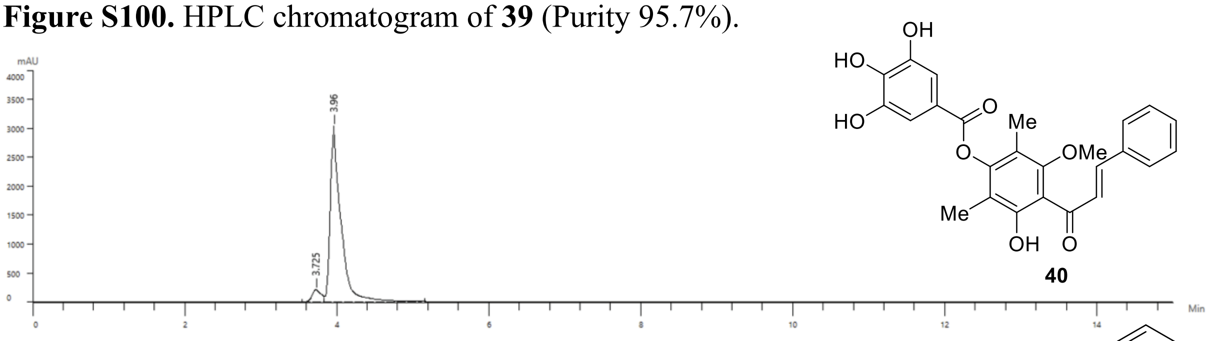

Figure S101. HPLC chromatogram of **40** (Purity 95.0%).

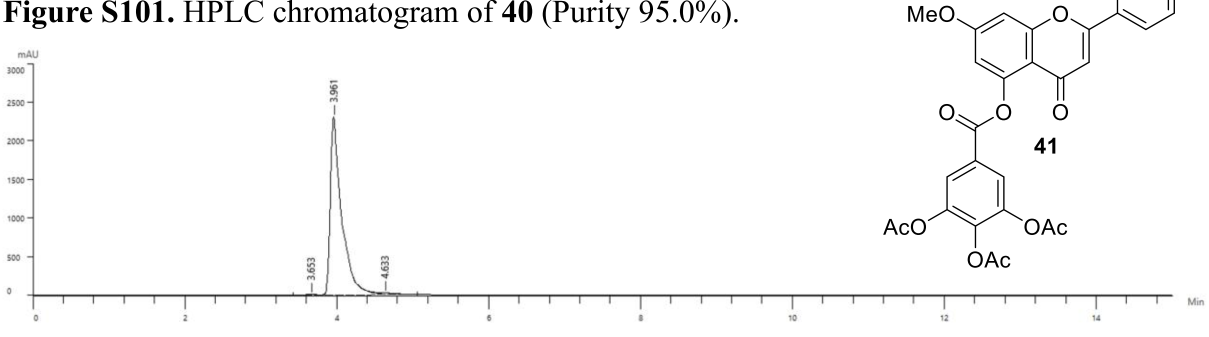

Figure S102. HPLC chromatogram of **41** (Purity 98.2%).

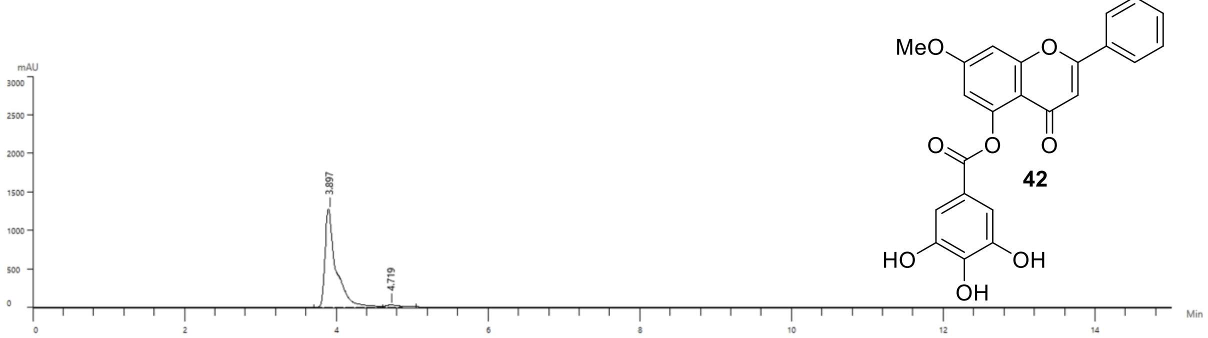

Figure S103. HPLC chromatogram of **42** (Purity 97.7%).

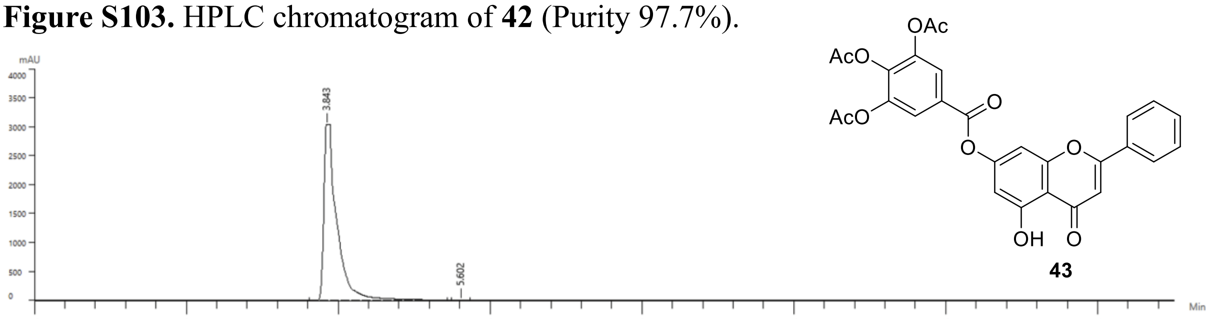

Figure S104. HPLC chromatogram of **43** (Purity >99.9%).

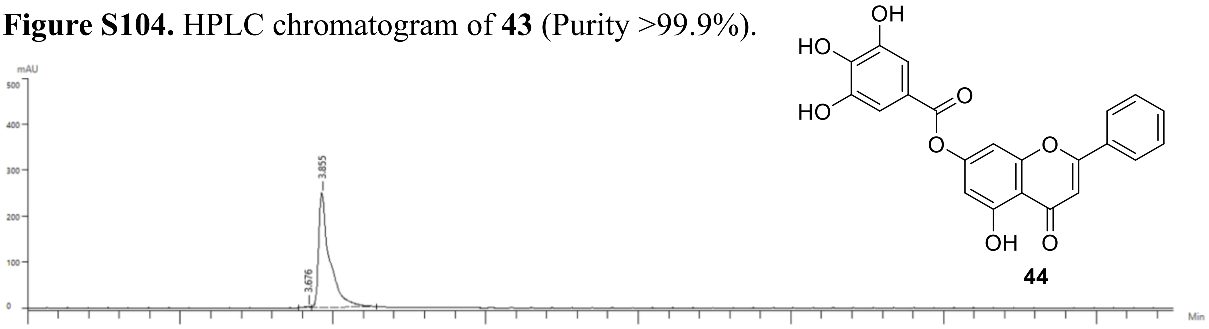

Figure S105. HPLC chromatogram of **44** (Purity 99.3%).

Section II

SMILES Format of Compounds

Table S1. Chemical structures and their SMILES

Cpd.	Chemical structure	SMILES
**6**	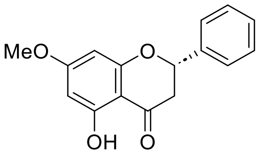	O=C1C[C@@H](C2=CC=CC=C2)OC3=C1C(O)=CC(OC)=C3
**7**	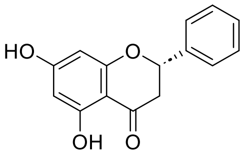	O=C1C[C@@H](C2=CC=CC=C2)OC3=C1C(O)=CC(O)=C3
**8**	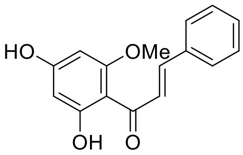	O=C(C1=C(OC)C=C(O)C=C1O)/C=C/C2=CC=CC=C2
**9**	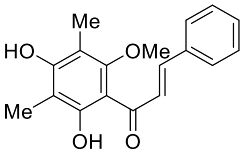	O=C(C1=C(OC)C(C)=C(O)C(C)=C1O)/C=C/C2=CC=CC=C2
**10**	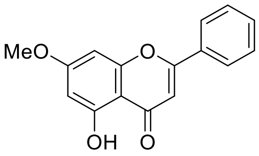	O=C1C=C(C2=CC=CC=C2)OC3=C1C(O)=CC(OC)=C3
**11**	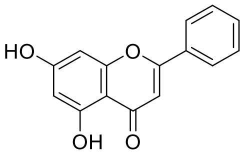	O=C1C=C(C2=CC=CC=C2)OC3=C1C(O)=CC(O)=C3
**12**	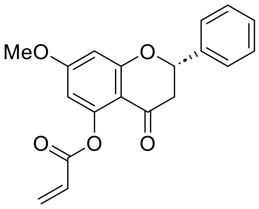	O=C1C[C@@H](C2=CC=CC=C2)OC3=C1C(OC(C=C)=O)=CC(OC)=C3
**13**	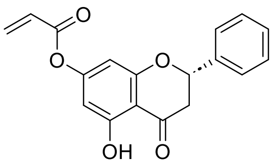	O=C1C[C@@H](C2=CC=CC=C2)OC3=C1C(O)=CC(OC(C=C)=O)=C3
**14**	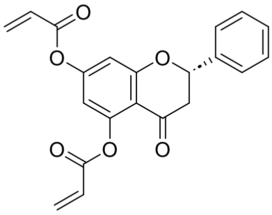	O=C1C[C@@H](C2=CC=CC=C2)OC3=C1C(OC(C=C)=O)=CC(OC(C=C)=O)=C3

Cpd.	Chemical structure	SMILES
**15**	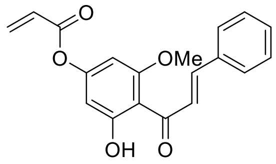	O=C(C1=C(OC)C=C(OC(C=C)=O)C=C1O)/C=C/C2=CC=CC=C2
**16**	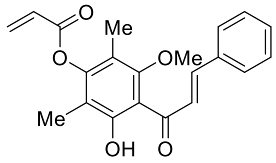	O=C(C1=C(OC)C(C)=C(OC(C=C)=O)C(C)=C1O)/C=C/C2=CC=CC=C2
**17**	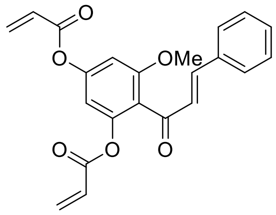	O=C(C1=C(OC)C=C(OC(C=C)=O)C=C1OC(C=C)=O)/C=C/C2=CC=CC=C2
**18**	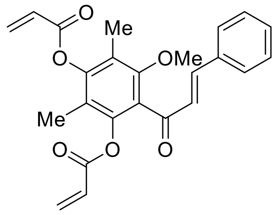	O=C(C1=C(OC)C(C)=C(OC(C=C)=O)C(C)=C1OC(C=C)=O)/C=C/C2=CC=CC=C2
**19**	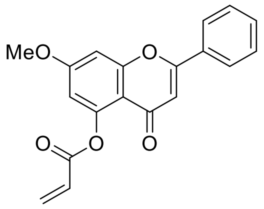	O=C1C=C(C2=CC=CC=C2)OC3=C1C(OC(C=C)=O)=CC(OC)=C3
**20**	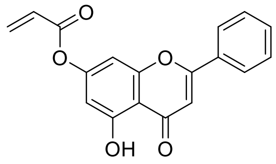	O=C1C=C(C2=CC=CC=C2)OC3=C1C(O)=CC(OC(C=C)=O)=C3
**21**	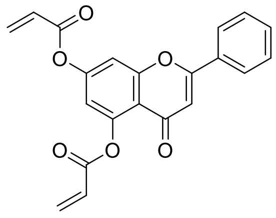	O=C1C=C(C2=CC=CC=C2)OC3=C1C(OC(C=C)=O)=CC(OC(C=C)=O)=C3
**22**	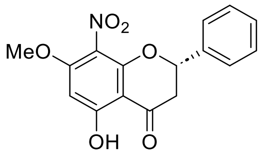	O=C1C[C@@H](C2=CC=CC=C2)OC3=C1C(O)=CC(OC)=C3[N+]([O-])=O

Cpd.	Chemical structure	SMILES
**23**	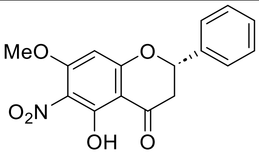	O=C1C[C@@H](C2=CC=CC=C2)OC3=C1C(O)=C([N+]([O-])=O)C(OC)=C3
**24**	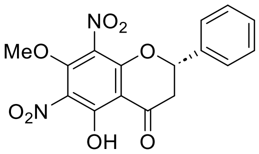	O=C1C[C@@H](C2=CC=CC=C2)OC3=C1C(O)=C([N+]([O-])=O)C(OC)=C3[N+]([O-])=O
**25**	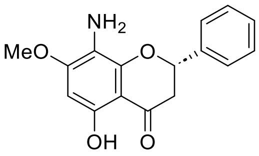	O=C1C[C@@H](C2=CC=CC=C2)OC3=C1C(O)=CC(OC)=C3N
**26**	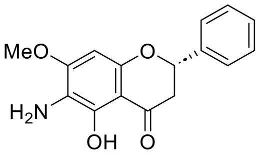	O=C1C[C@@H](C2=CC=CC=C2)OC3=C1C(O)=C(N)C(OC)=C3
**27**	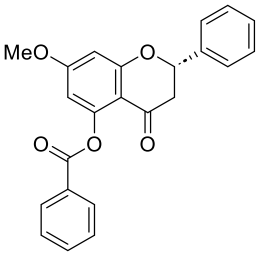	O=C1C[C@@H](C2=CC=CC=C2)OC3=C1C(OC(C4=CC=CC=C4)=O)=CC(OC)=C3
**28**	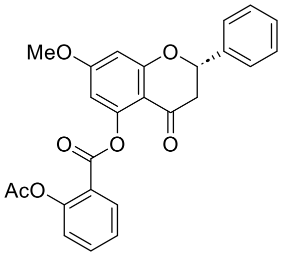	O=C1C[C@@H](C2=CC=CC=C2)OC3=C1C(OC(C4=C(OC(C)=O)C=CC=C4)=O)=CC(OC)=C3
**29**	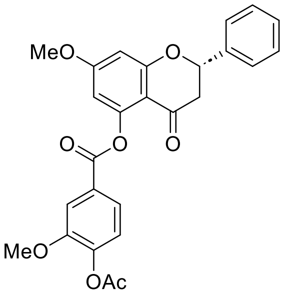	O=C1C[C@@H](C2=CC=CC=C2)OC3=C1C(OC(C4=CC(OC)=C(OC(C)=O)C=C4)=O)=CC(OC)=C3

Cpd.	Chemical structure	SMILES
**30**	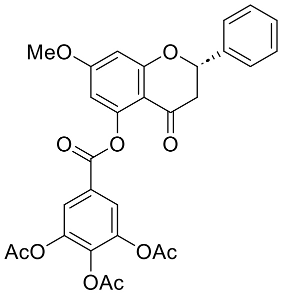	O=C1C[C@@H](C2=CC=CC=C2)OC3=C1C(OC(C4=CC(OC(C)=O)=C(OC(C)=O)C(OC(C)=O)=C4)=O)=CC(OC)=C3
**31**	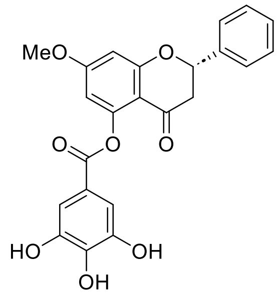	O=C1C[C@@H](C2=CC=CC=C2)OC3=C1C(OC(C4=CC(O)=C(O)C(O)=C4)=O)=CC(OC)=C3
**32**	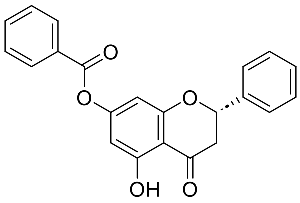	O=C1C[C@@H](C2=CC=CC=C2)OC3=C1C(O)=CC(OC(C4=CC=CC=C4)=O)=C3
**33**	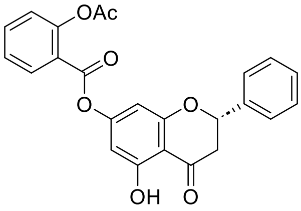	O=C1C[C@@H](C2=CC=CC=C2)OC3=C1C(O)=CC(OC(C4=C(OC(C)=O)C=CC=C4)=O)=C3
**34**	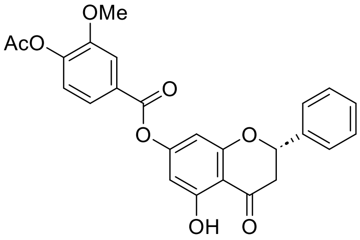	O=C1C[C@@H](C2=CC=CC=C2)OC3=C1C(O)=CC(OC(C4=CC(OC)=C(OC(C)=O)C=C4)=O)=C3
**35**	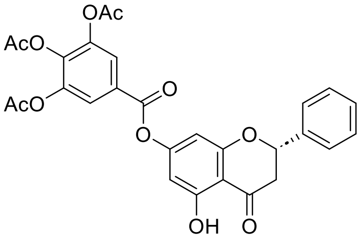	O=C1C[C@@H](C2=CC=CC=C2)OC3=C1C(O)=CC(OC(C4=CC(OC(C)=O)=C(OC(C)=O)C(OC(C)=O)=C4)=O)=C3

Cpd.	Chemical structure	SMILES
**36**	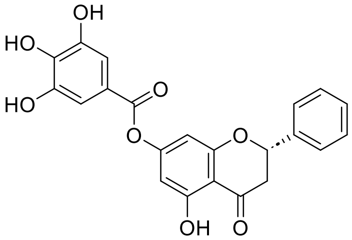	O=C1C[C@@H](C2=CC=CC=C2)OC3=C1C(O)=CC(OC(C4=CC(O)=C(O)C(O)=C4)=O)=C3
**37**	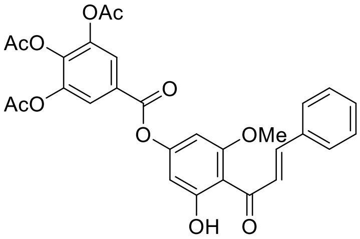	O=C(C1=C(OC)C=C(OC(C2=CC(OC(C)=O)=C(OC(C)=O)C(OC(C)=O)=C2)=O)C=C1O)/C=C/C3=CC=CC=C3
**38**	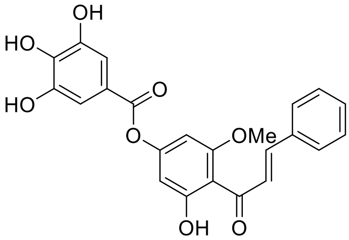	O=C(C1=C(OC)C=C(OC(C2=CC(O)=C(O)C(O)=C2)=O)C=C1O)/C=C/C3=CC=CC=C3
**39**	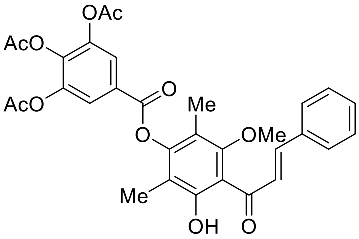	O=C(C1=C(OC)C(C)=C(OC(C2=CC(OC(C)=O)=C(OC(C)=O)C(OC(C)=O)=C2)=O)C(C)=C1O)/C=C/C3=CC=CC=C3
**40**	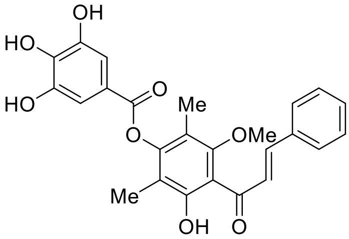	O=C(C1=C(OC)C(C)=C(OC(C2=CC(O)=C(O)C(O)=C2)=O)C(C)=C1O)/C=C/C3=CC=CC=C3
**41**	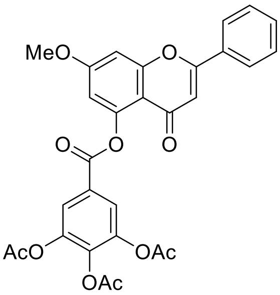	O=C1C=C(C2=CC=CC=C2)OC3=C1C(OC(C4=CC(OC(C)=O)=C(OC(C)=O)C(OC(C)=O)=C4)=O)=CC(OC)=C3

Cpd.	Chemical structure	SMILES
**42**	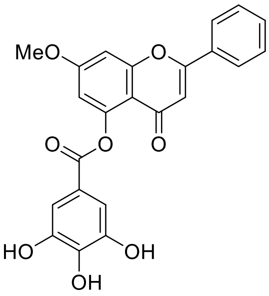	O=C1C=C(C2=CC=CC=C2)OC3=C1C(OC(C4=CC(O)=C(O)C(O)=C4)=O)=CC(OC)=C3
**43**	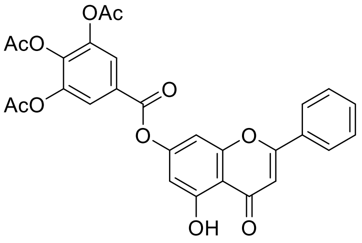	O=C1C=C(C2=CC=CC=C2)OC3=C1C(O)=CC(OC(C4=CC(OC(C)=O)=C(OC(C)=O)C(OC(C)=O)=C4)=O)=C3
**44**	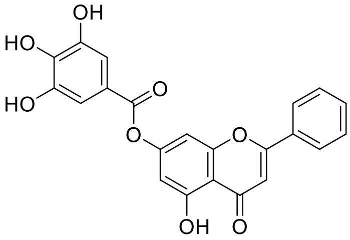	O=C1C=C(C2=CC=CC=C2)OC3=C1C(O)=CC(OC(C4=CC(O)=C(O)C(O)=C4)=O)=C3
gallic acid	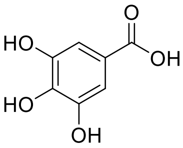	OC1=CC(C(O)=O)=CC(O)=C1O
baicalein	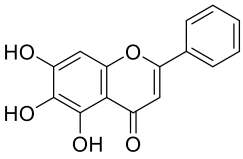	O=C1C=C(C2=CC=CC=C2)OC3=C1C(O)=C(C(O)=C3)O

Section III

Computational Simulations

Molecular Docking, Molecular Dynamics Simulation, and MM-GBSA Calculations

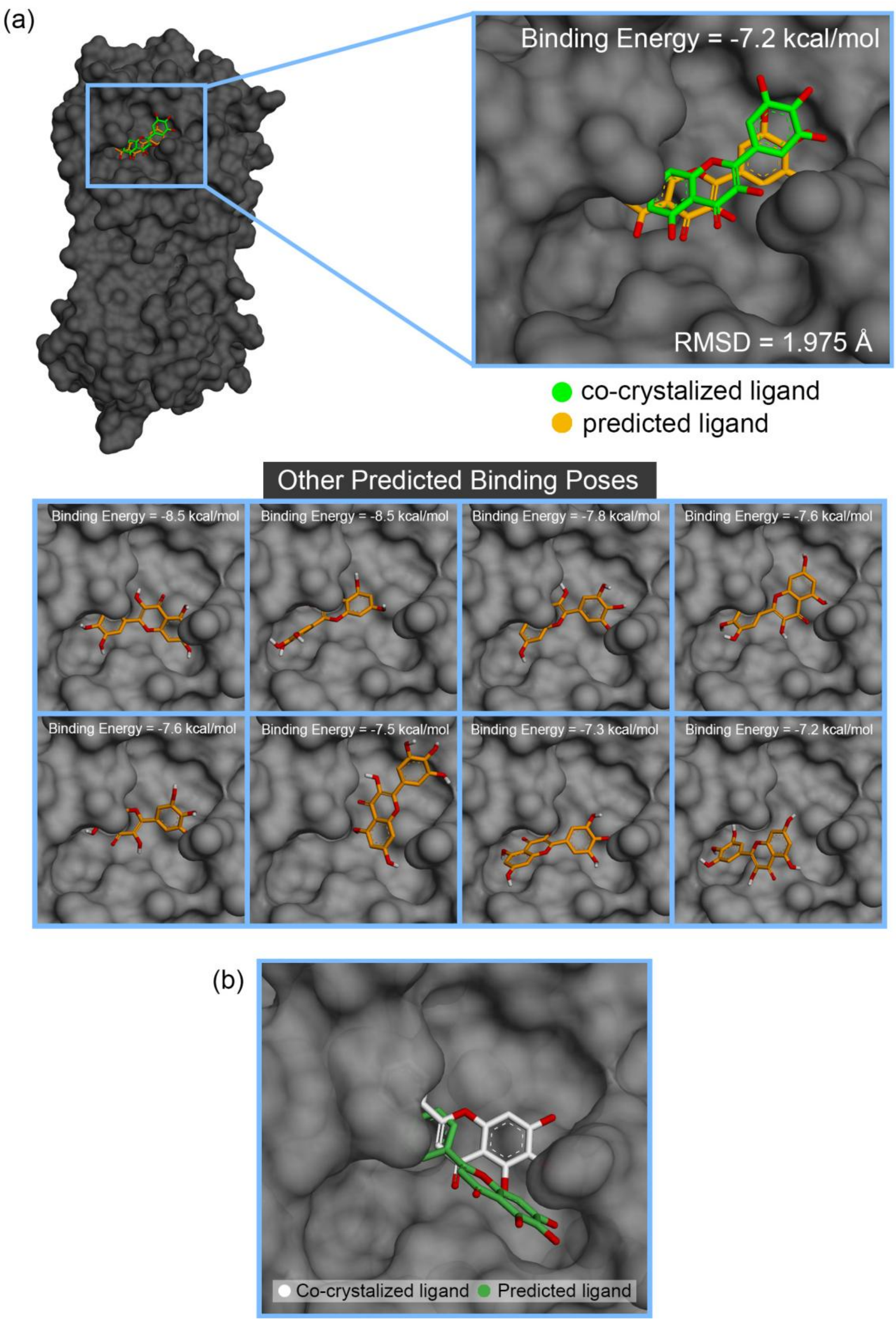

Figure S106. Validation of molecular docking protocol. (a) Redocking of a co-crystalized ligand to substrate binding site of $3CL^{pro}$ (PDB ID: 7B3E). The RMSD value of selected pose was calculated using all atoms, excluding non-polar hydrogens. (b) Docking of a baicalein to substrate binding site of $3CL^{pro}$.

Table S2. The molecular docking results and binding poses for MD simulations at the binding site of SARS-CoV-2 3CL^{pro}

Cpd.	Binding Pose		
	Pose 1	Pose 2	Pose 3
**7**	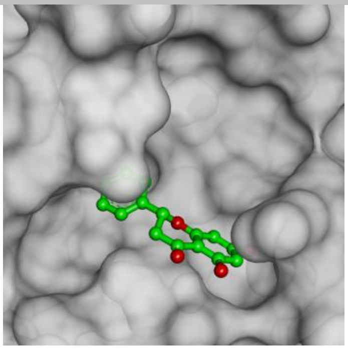<br>(BE = −7.0 kcal/mol)	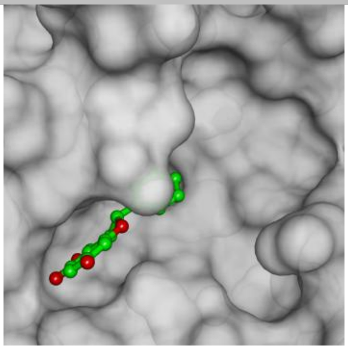<br>(BE = −7.0 kcal/mol)	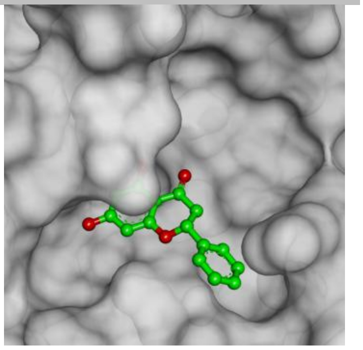<br>(BE = −6.7 kcal/mol)
**9**	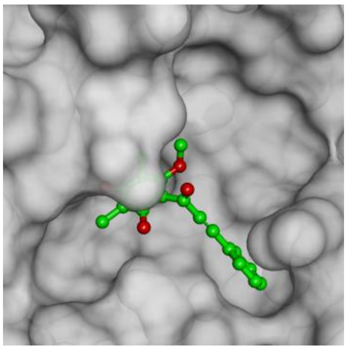<br>(BE = −6.5 kcal/mol)	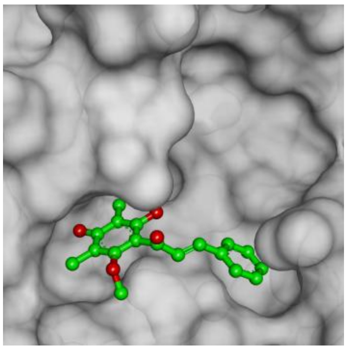<br>(BE = −6.2 kcal/mol)	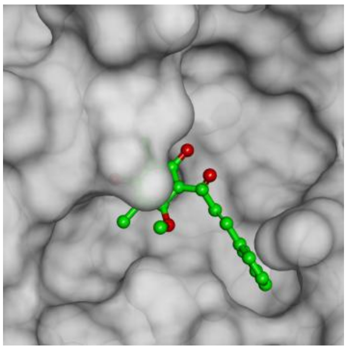<br>(BE = −6.1 kcal/mol)
**31**	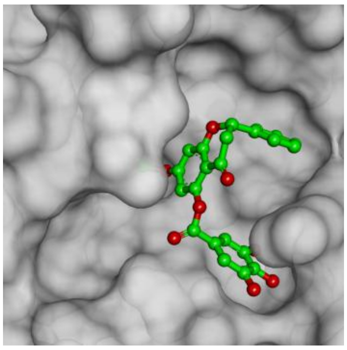<br>(BE = −7.7 kcal/mol)	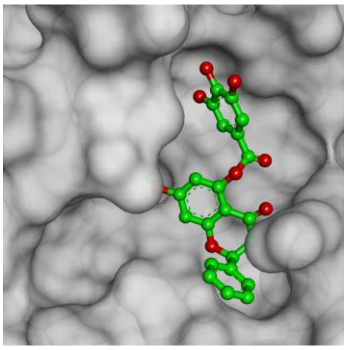<br>(BE = −7.5 kcal/mol)	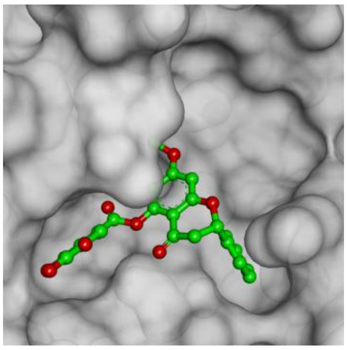<br>(BE = −7.4 kcal/mol)

Cpd.	Binding Pose		
	Pose 1	Pose 2	Pose 3
**35**	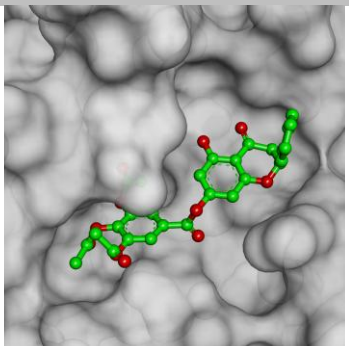<br>(BE = −7.4 kcal/mol)	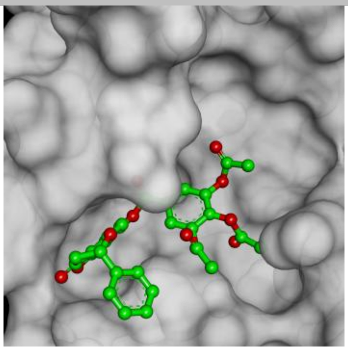<br>(BE = −7.4 kcal/mol)	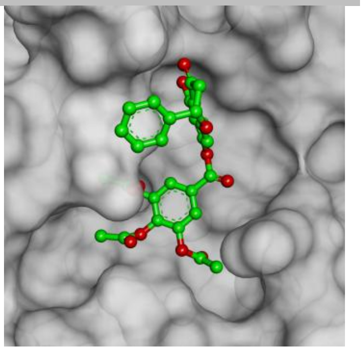<br>(BE = −7.4 kcal/mol)
**36**	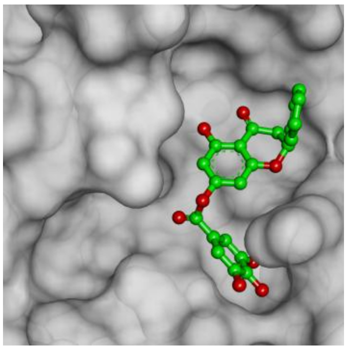<br>(BE = −7.7 kcal/mol)	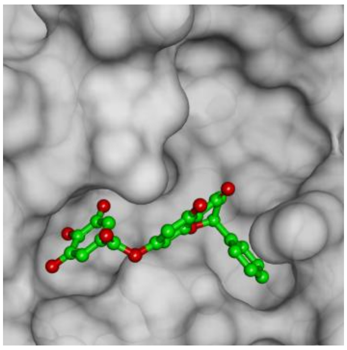<br>(BE = −7.3 kcal/mol)	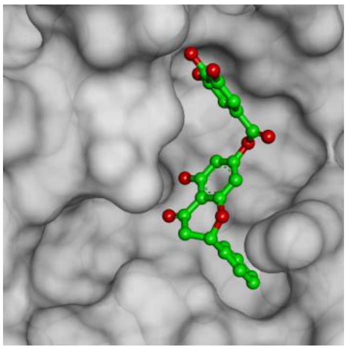<br>(BE = −7.1 kcal/mol)
**40**	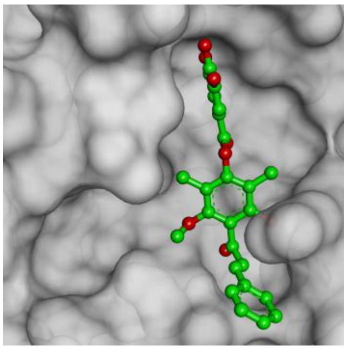<br>(BE = −7.3 kcal/mol)	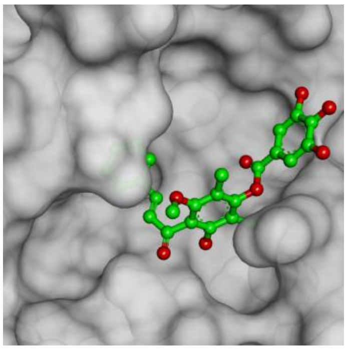<br>(BE = −7.0 kcal/mol)	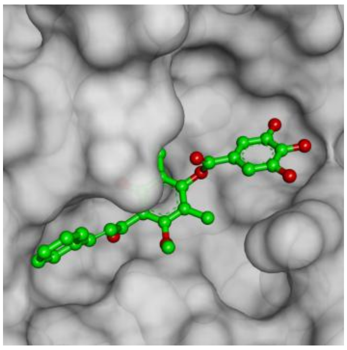<br>(BE = −6.9 kcal/mol)

Cpd.	Binding Pose		
	Pose 1	Pose 2	Pose 3
**42**	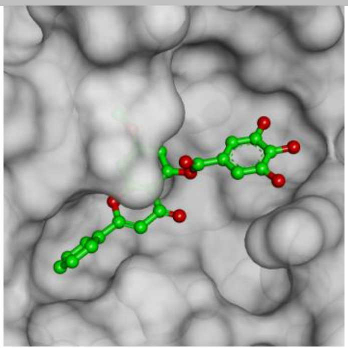 (BE = −8.3 kcal/mol)	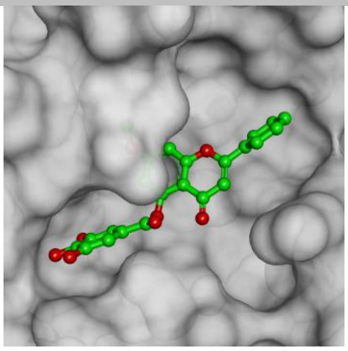 (BE = −7.7 kcal/mol)	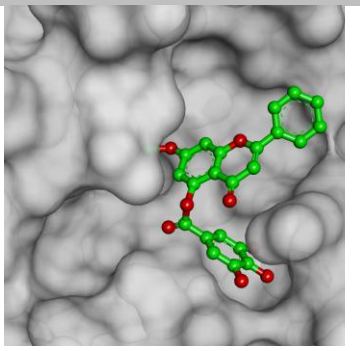 (BE = −7.7 kcal/mol)
**44**	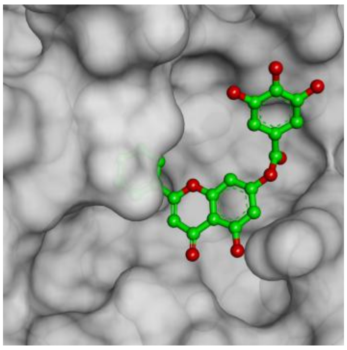 (BE = −8.7 kcal/mol)	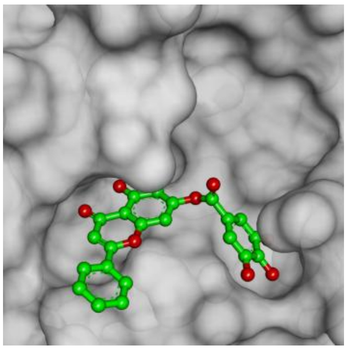 (BE = −7.7 kcal/mol)	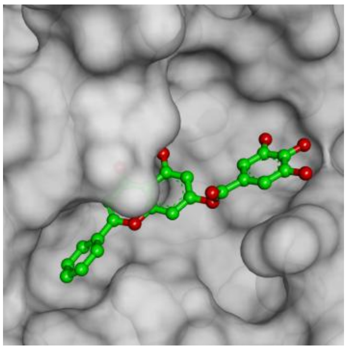 (BE = −7.7 kcal/mol)

Table S3. Seed values for molecular dynamics simulation

Complex	Pose	Seed value
SARS-CoV-2 3CL^{pro} apo form	1	-1082558753
	2	1996446959
	3	-572792908
SARS-CoV-2 3CL^{pro} – **7**	1	-572531661
	2	-352372738
	3	-679649837
SARS-CoV-2 3CL^{pro} – **9**	1	-136624642
	2	977006127
	3	2012733111
SARS-CoV-2 3CL^{pro} - **31**	1	-557850649
	2	-268437892
	3	-1109413073

Complex	Pose	Seed value
SARS-CoV-2 3CL^{pro} - **35**	1	-302391881
	2	-151030529
	3	1851784175
SARS-CoV-2 3CL^{pro} - **36**	1	2147467089
	2	1860662403
	3	-1150291762
SARS-CoV-2 3CL^{pro} - **40**	1	-574648769
	2	1463810047
	3	-3182085
SARS-CoV-2 3CL^{pro} - **42**	1	-277119521
	2	-959485457
	3	-281133071
SARS-CoV-2 3CL^{pro} - **44**	1	-1385168917
	2	1073208830
	3	-69705

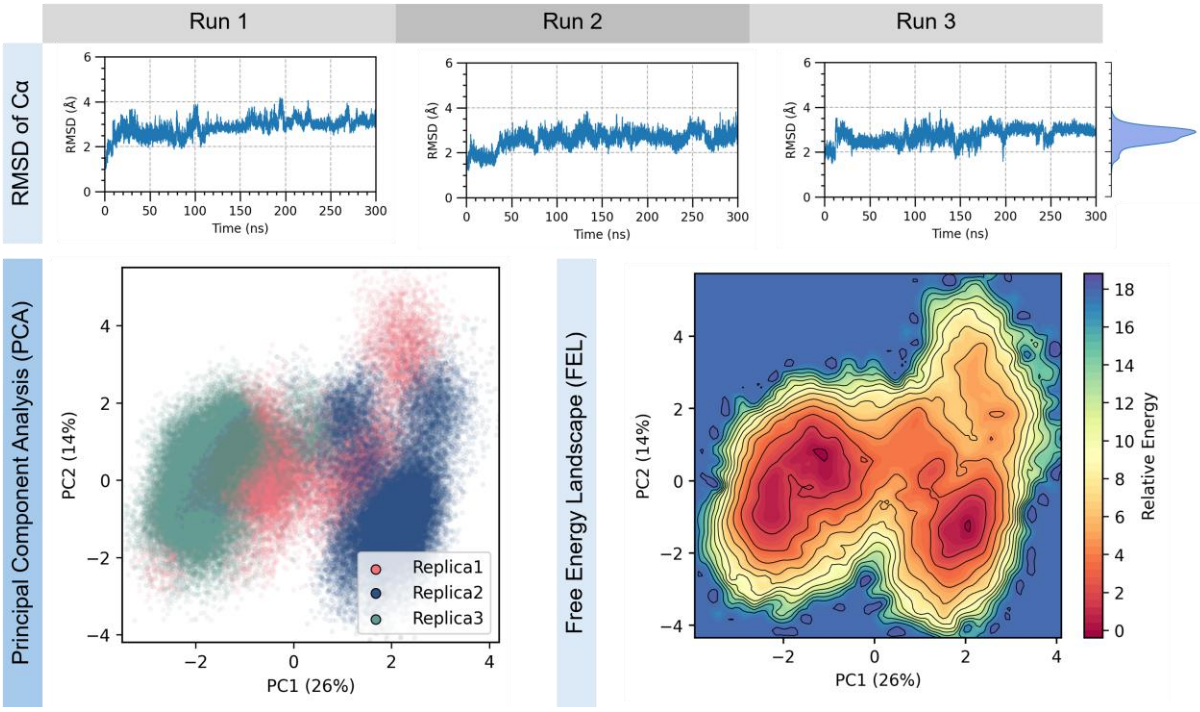

Figure S107. Analysis of three independent simulations of 300 ns molecular dynamics simulations of SARS-CoV-2 3CL^{pro} in apo form. The analysis includes RMSD of protein Cα, principal component analysis (PCA), and free energy landscape (FEL).

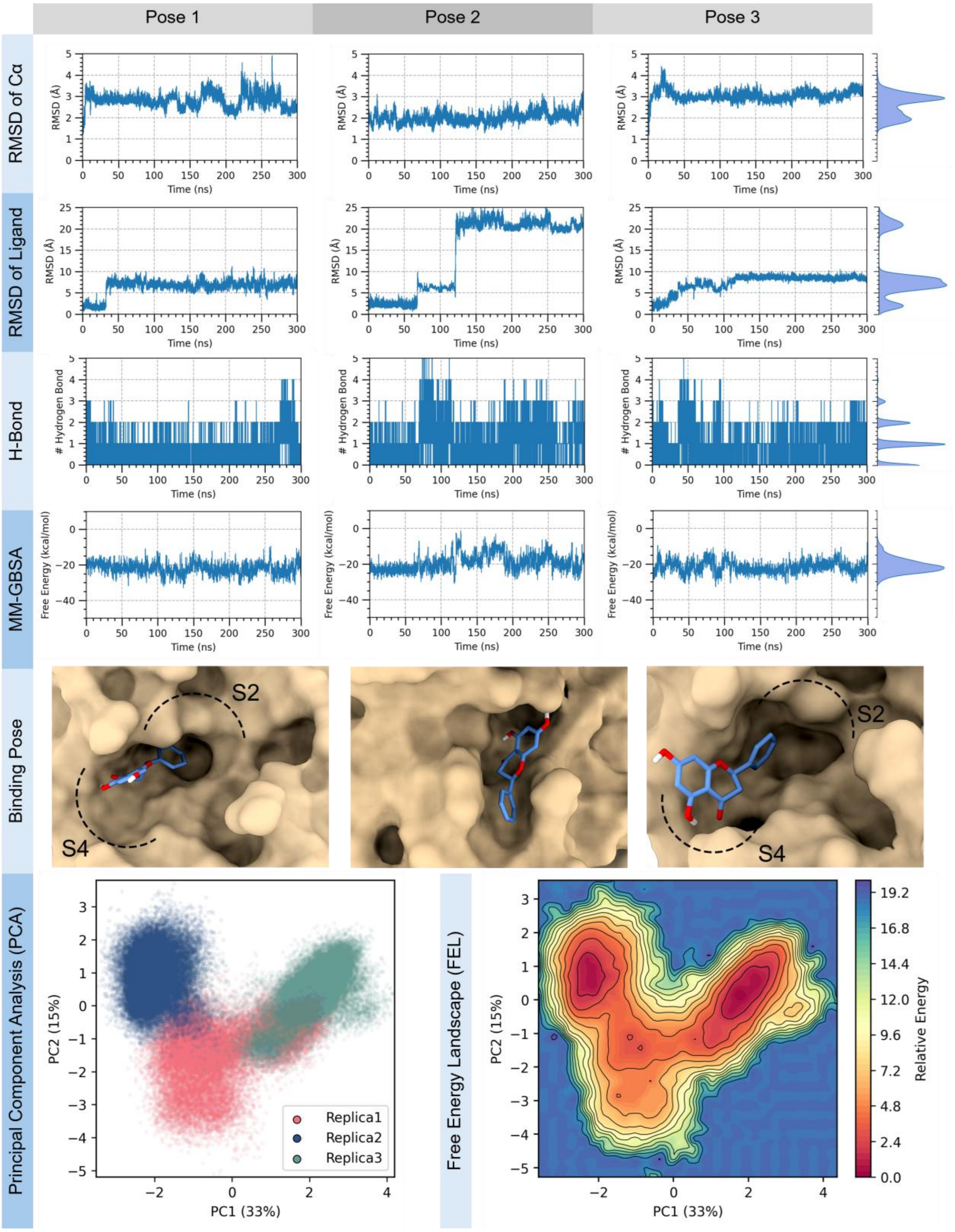

Figure S108. Analysis of three independent simulations of 300 ns molecular dynamics simulations of pinocembrin (**7**). The analysis includes RMSD of protein Cα, RMSD of ligand, number of hydrogen bonds, evolution of binding free energy, principal component analysis (PCA), and free energy landscape (FEL).

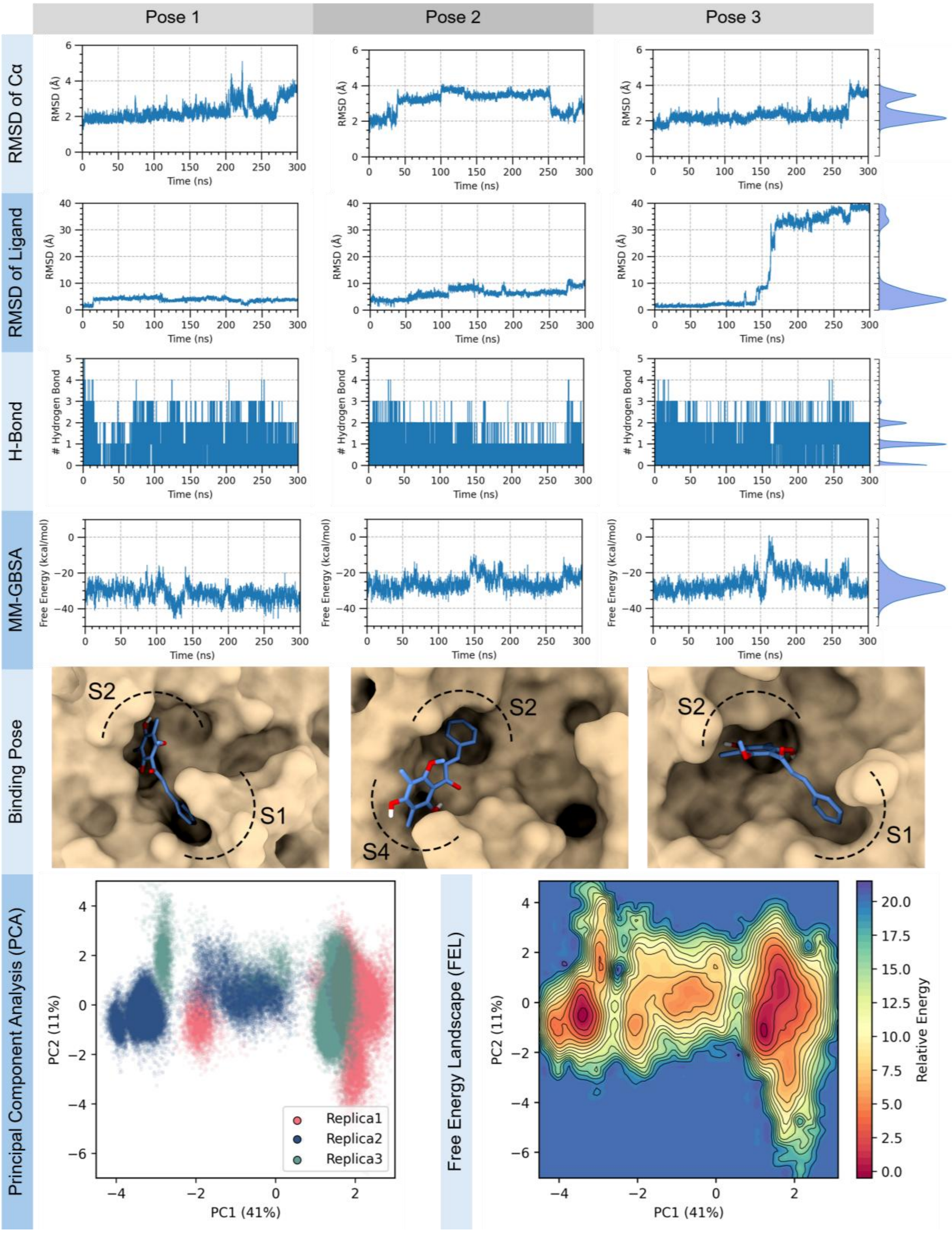

Figure S109. Analysis of three independent simulations of 300 ns molecular dynamics simulations of DMC (**9**). The analysis includes RMSD of protein Cα, RMSD of ligand, number of hydrogen bonds, evolution of binding free energy, principal component analysis (PCA), and free energy landscape (FEL).

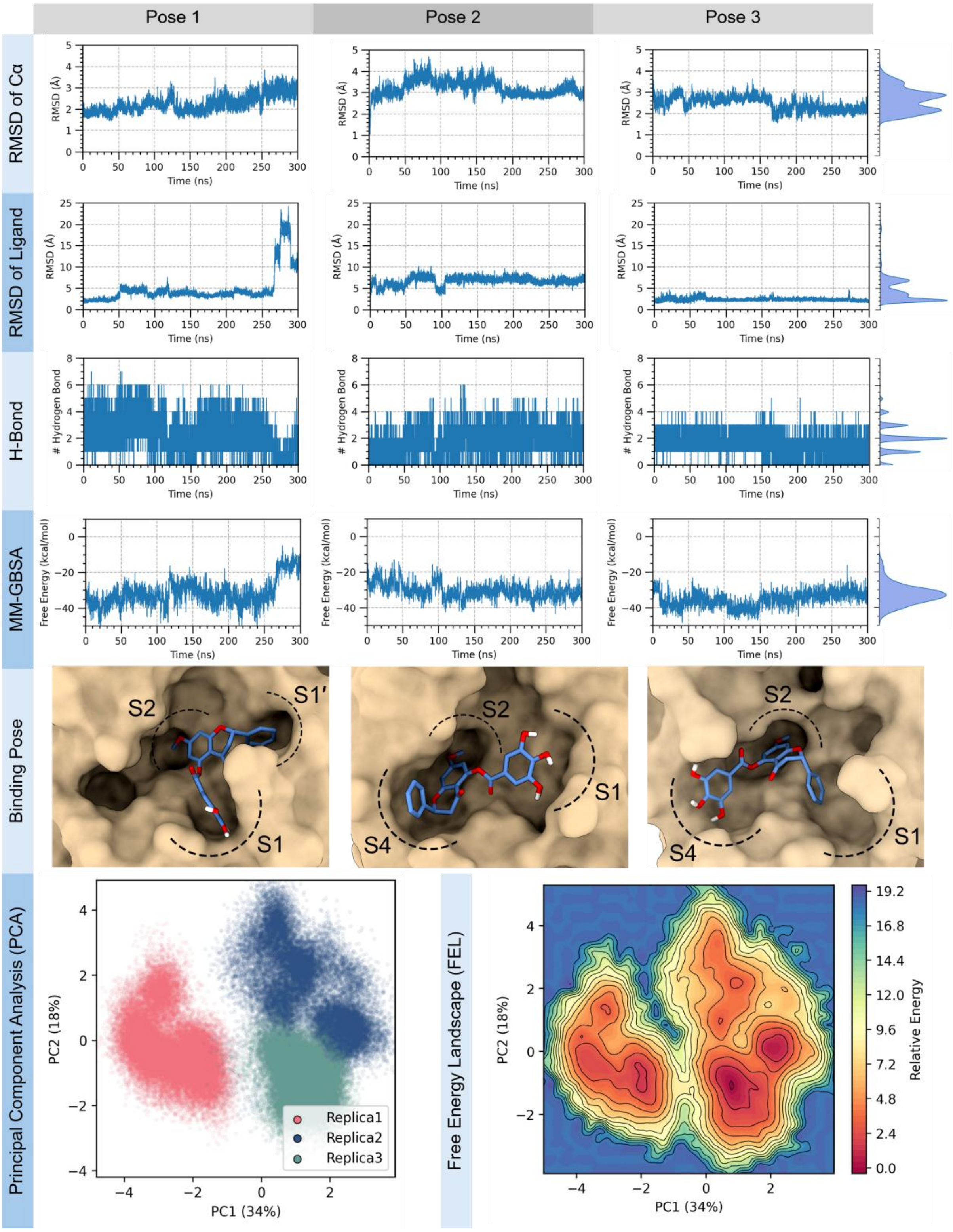

Figure S110. Analysis of three independent simulations of 300 ns molecular dynamics simulations of 5-*O*-galloylpinostrobin (**31**). The analysis includes RMSD of protein Cα, RMSD of ligand, number of hydrogen bonds, evolution of binding free energy, principal component analysis (PCA), and free energy landscape (FEL).

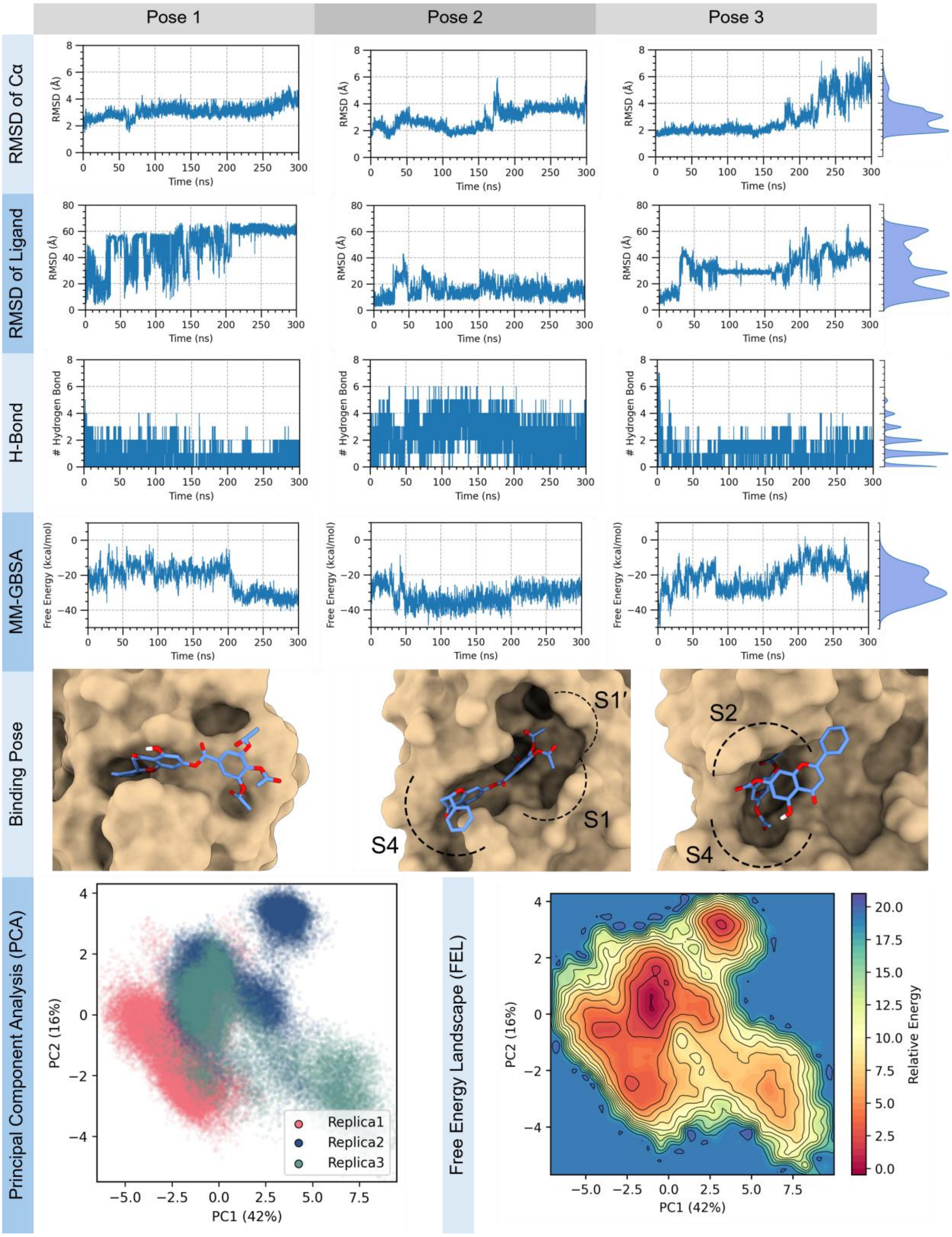

Figure S111. Analysis of three independent simulations of 300 ns molecular dynamics simulations of 7-*O*-(tri-*O*-acetylgalloyl)pinocembrin (**35**). The analysis includes RMSD of protein Cα, RMSD of ligand, number of hydrogen bonds, evolution of binding free energy, principal component analysis (PCA), and free energy landscape (FEL).

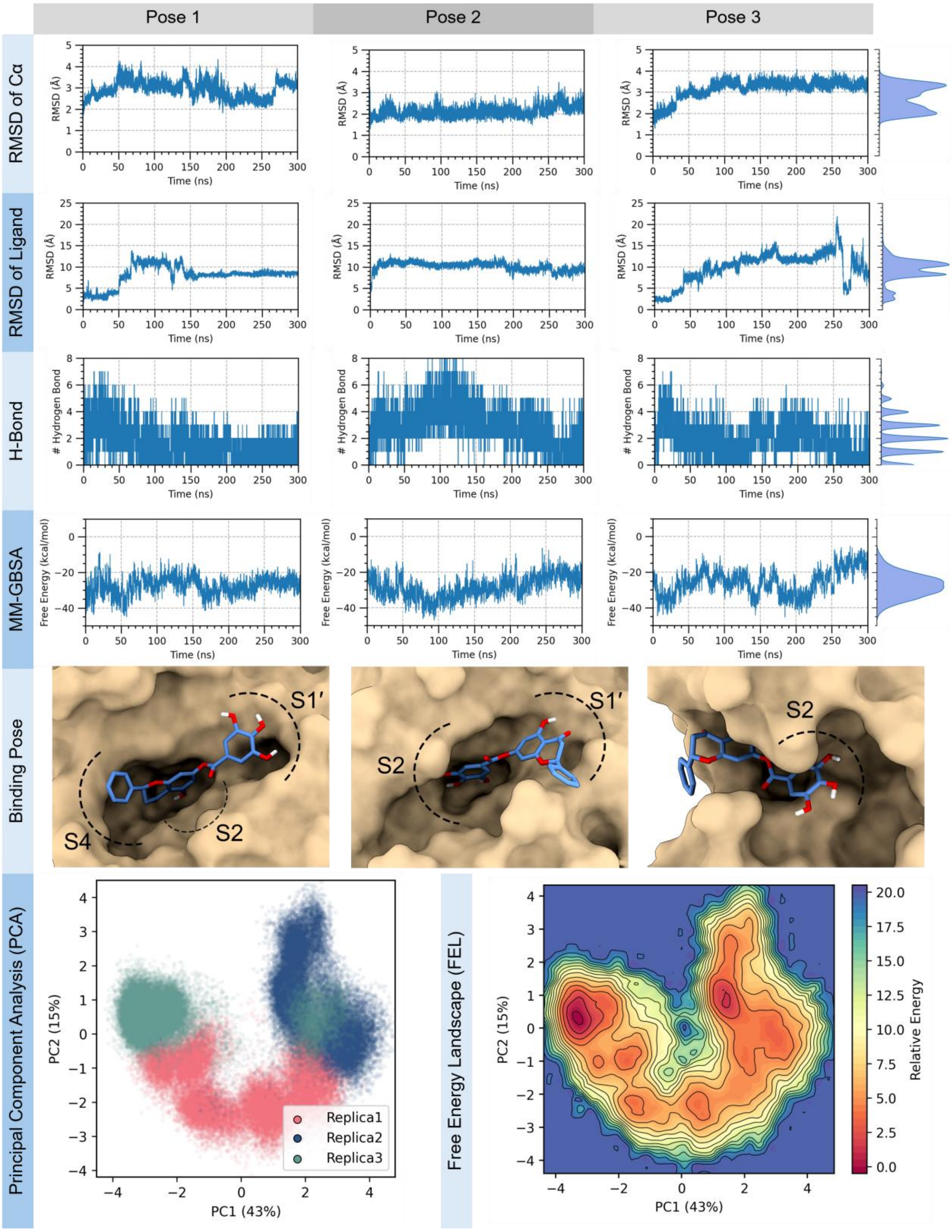

Figure S112. Analysis of three independent simulations of 300 ns molecular dynamics simulations of 7-*O*-galloylpinocembrin (**36**). The analysis includes RMSD of protein Cα, RMSD of ligand, number of hydrogen bonds, evolution of binding free energy, principal component analysis (PCA), and free energy landscape (FEL).

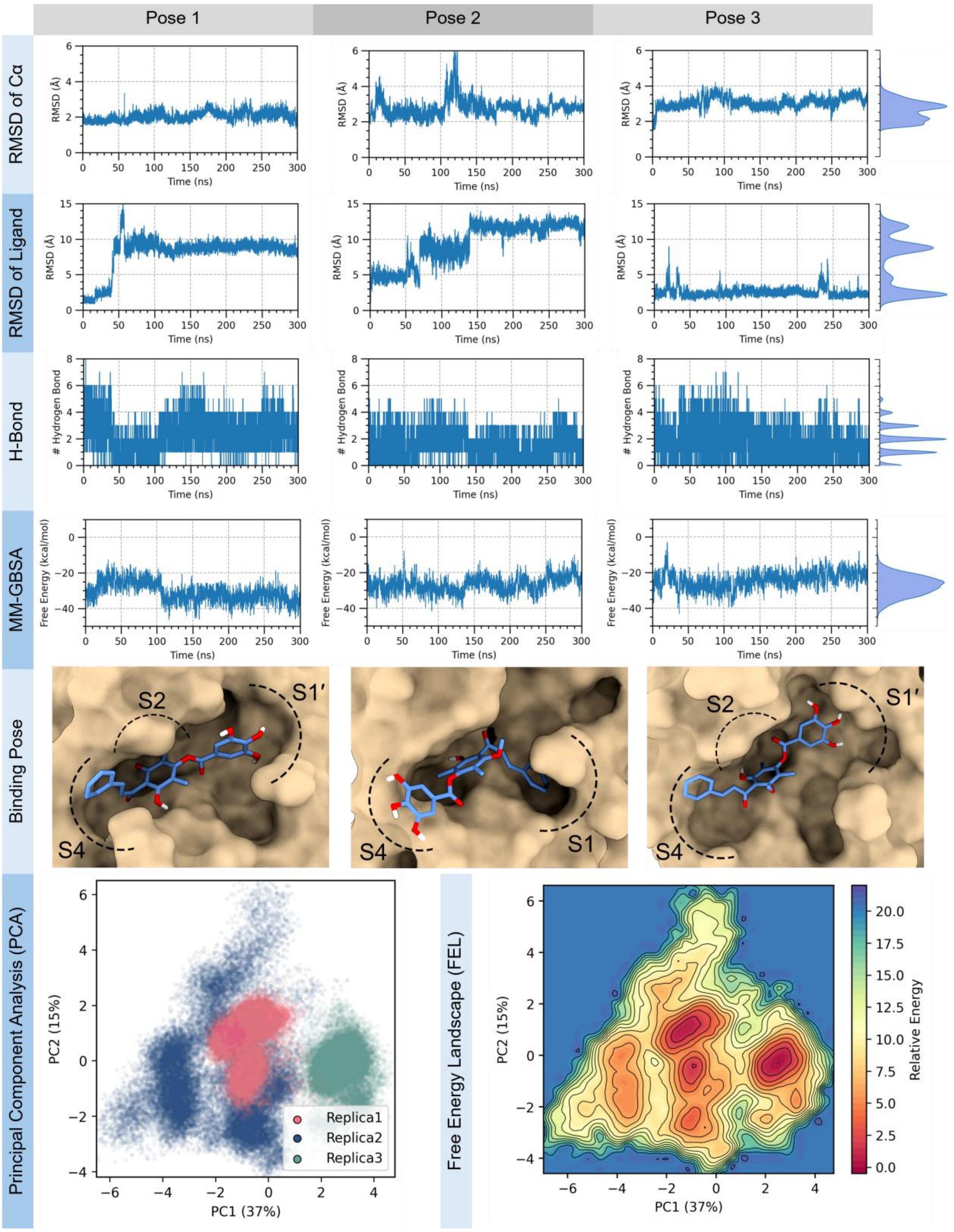

Figure S113. Analysis of three independent simulations of 300 ns molecular dynamics simulations of 4′-*O*-galloyl-DMC (**40**). The analysis includes RMSD of protein Cα, RMSD of ligand, number of hydrogen bonds, evolution of binding free energy, principal component analysis (PCA), and free energy landscape (FEL).

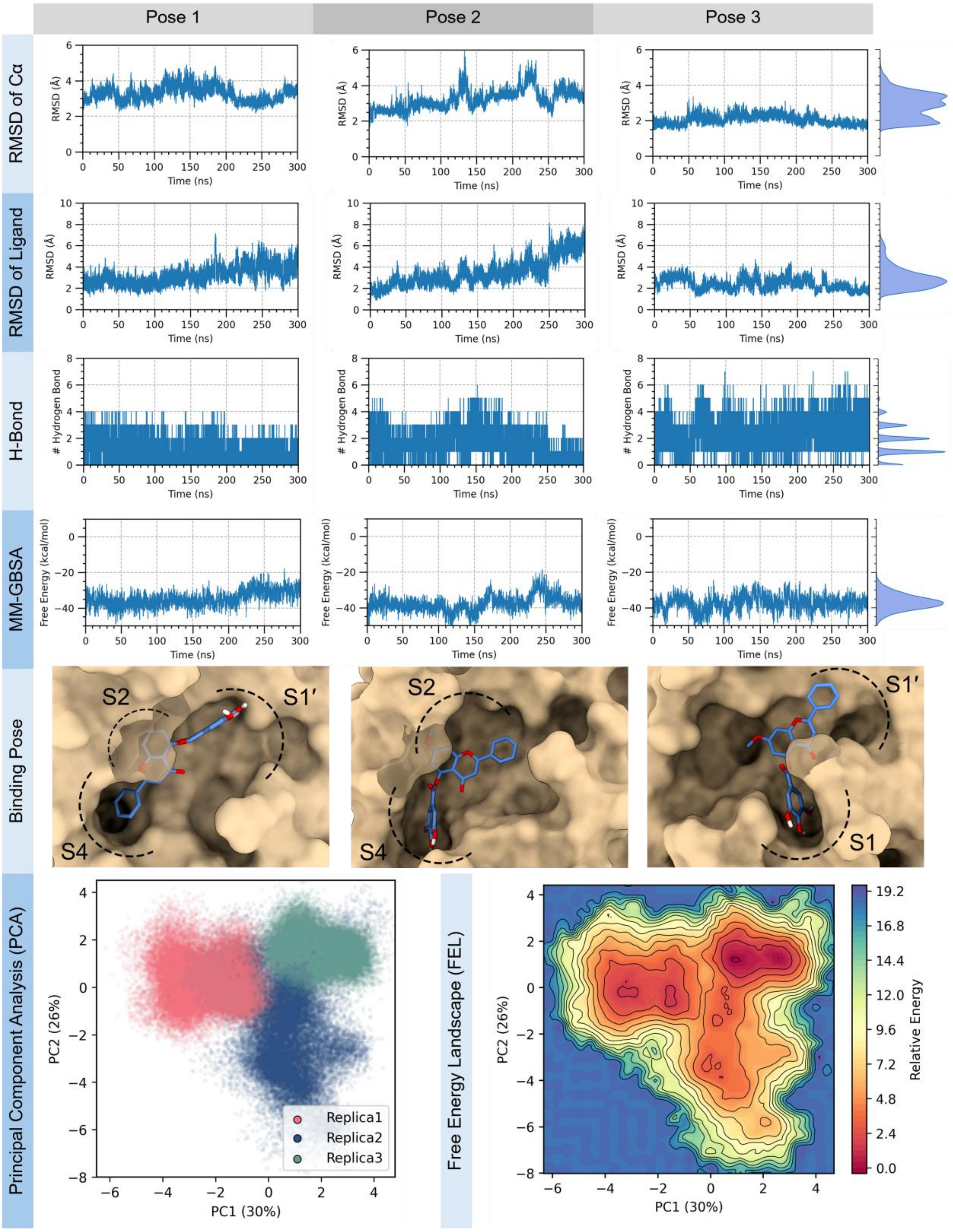

Figure S114. Analysis of three independent simulations of 300 ns molecular dynamics simulations of 7-*O*-galloyltectochrysin (**42**). The analysis includes RMSD of protein Cα, RMSD of ligand, number of hydrogen bonds, evolution of binding free energy, principal component analysis (PCA), and free energy landscape (FEL).

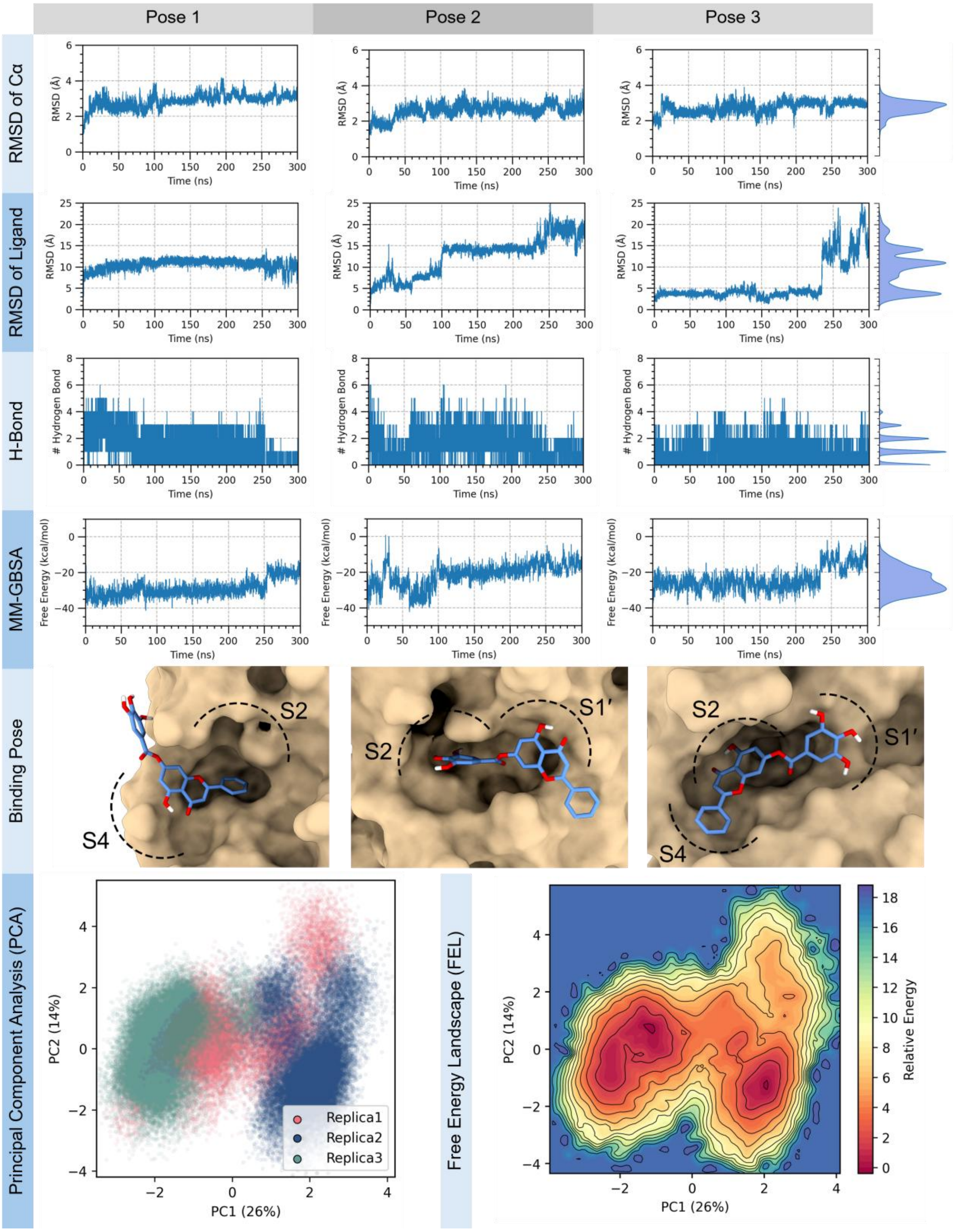

Figure S115. Analysis of three independent simulations of 300 ns molecular dynamics simulations of 5-*O*-galloylchrysin (**44**). The analysis includes RMSD of protein Cα, RMSD of ligand, number of hydrogen bonds, evolution of binding free energy, principal component analysis (PCA), and free energy landscape (FEL).

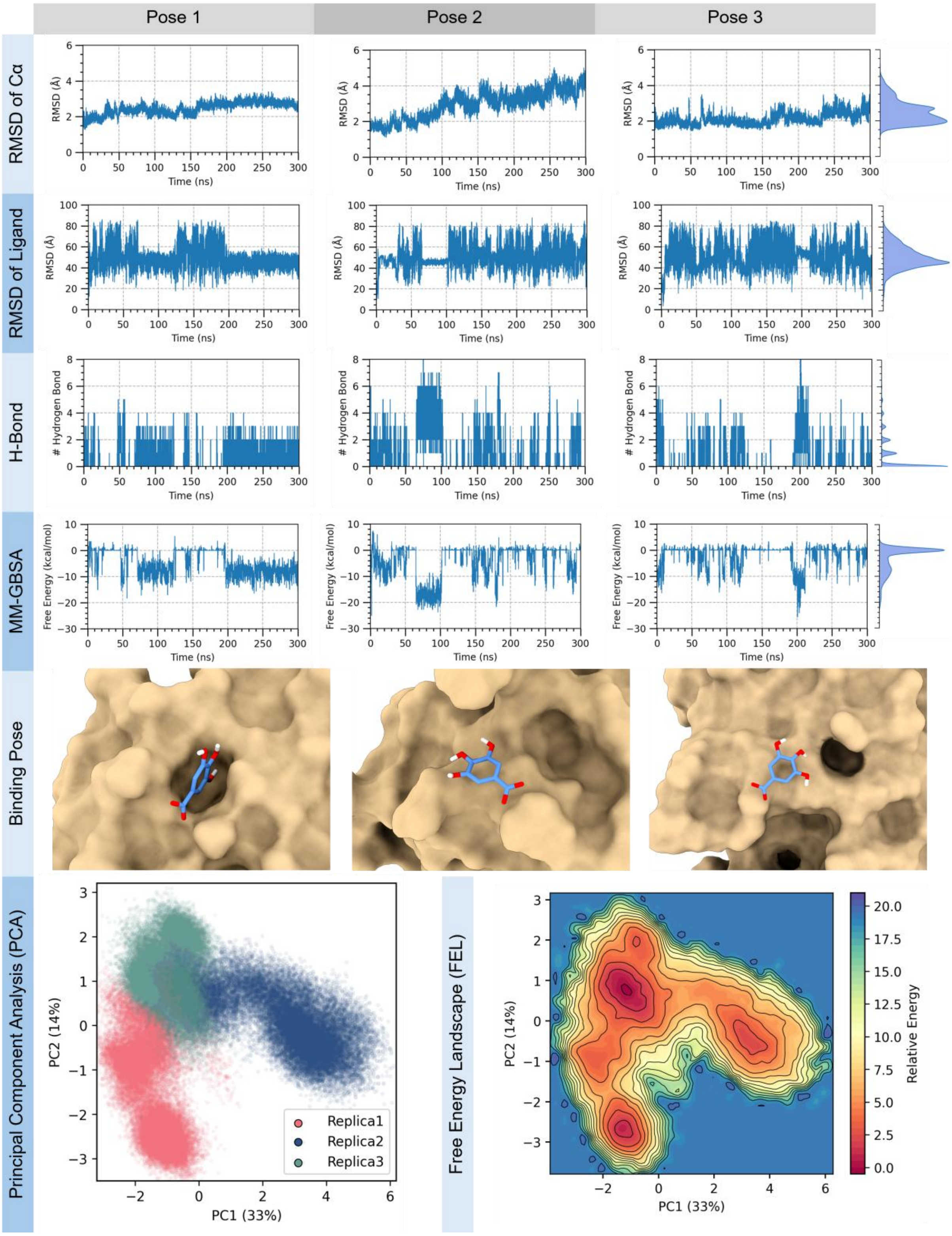

Figure S116. Analysis of three independent simulations of 300 ns molecular dynamics simulations of gallic acid. The analysis includes RMSD of protein Cα, RMSD of ligand, number of hydrogen bonds, evolution of binding free energy, principal component analysis (PCA), and free energy landscape (FEL).

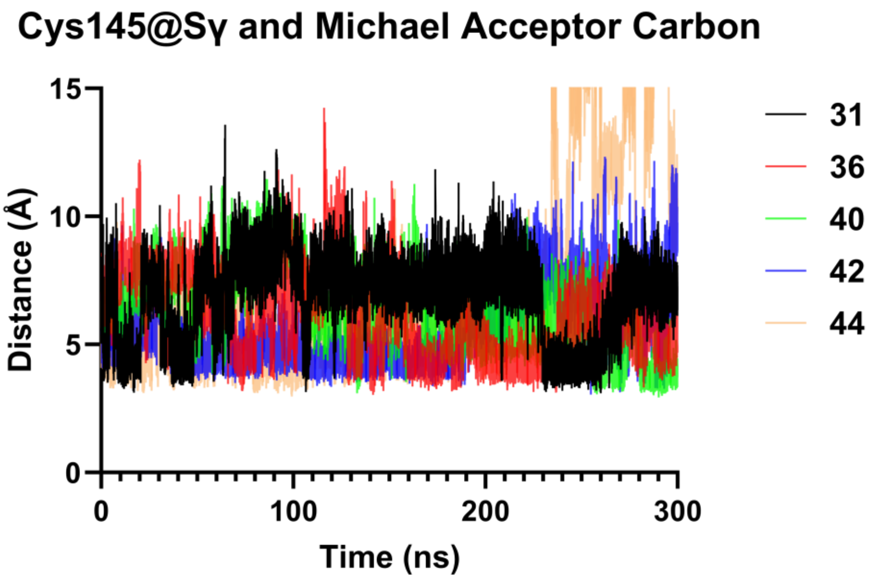

Figure S117. Distance between thiolate nucleophile of Cys145 and reactive Michael acceptor carbon of galloyl group

Section VI

Enzymatic Assay

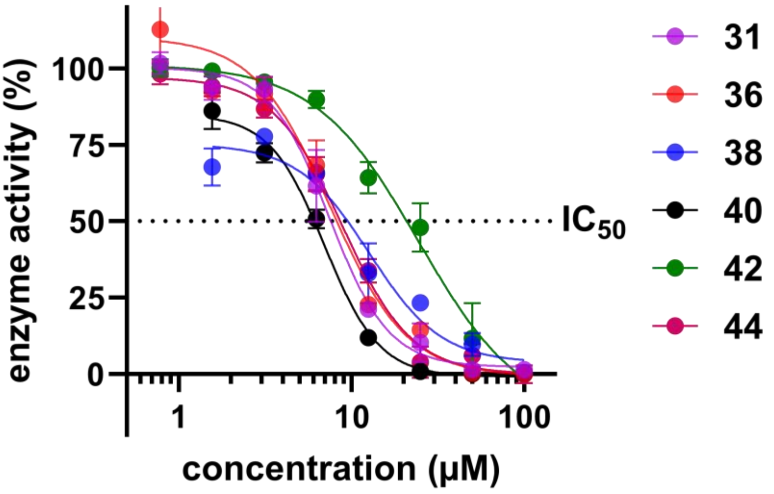

Figure S118. Dose-dependent curves of galloylated flavonoids against SARS-CoV-2 $3CL^{pro}$
